# Atroposelective Synthesis of Biaryl *N*‐Oxides via Cu‐Catalyzed De Novo Heteroaromatic *N*‐Oxide Ring Formation

**DOI:** 10.1002/advs.202405743

**Published:** 2024-08-09

**Authors:** Ke Ma, Ting Qi, Lei Hu, Chen Chen, Wan Wang, Jun‐Long Li, Cheng Peng, Gu Zhan, Bo Han

**Affiliations:** ^1^ State Key Laboratory of Southwestern Chinese Medicine Resources Hospital of Chengdu University of Traditional Chinese Medicine School of Pharmacy Chengdu University of Traditional Chinese Medicine Chengdu 611137 China; ^2^ Anti‐Infective Agent Creation Engineering Research Centre of Sichuan Province Sichuan Industrial Institute of Antibiotics School of Pharmacy Chengdu University Chengdu 610106 China; ^3^ Department of Biotherapy Cancer Center and State Key Laboratory of Biotherapy West China Hospital Sichuan University Chengdu 610041 China

**Keywords:** asymmetric catalysis, atroposelective synthesis, axially chiral, heteroaromatic N‐oxide, lewis base catalyst, pyridine‐N‐oxide, triple‐negative breast cancer

## Abstract

Heteroaromatic *N*‐oxides, renowned for their highly polar N─O bond and robust structure, exhibit significant bioactivities and have played a pivotal role in various drug development projects since the discovery of Minoxidil. Moreover, heteroaromatic *N*‐oxides, featuring axially chiral biaryl frameworks, are indispensable as Lewis base catalysts and ligands in organic synthesis. Despite their importance, synthesizing these chiral compounds is challenging, necessitating chiral starting materials or resolution processes. Catalytic strategies rely on the functionalization of heteroaromatic *N*‐oxide compounds, leading to products with a relatively limited skeletal diversity. This study introduces a Cu‐catalyzed atroposelective method for synthesizing biaryl *N*‐oxides via *de novo* heteroaromatic *N*‐oxide ring formation. This mild and efficient approach achieves excellent stereoselectivities (up to 99:1 er), enabling the production of a wide array of *N*‐oxides with novel heteroaromatic scaffolds. The axially chiral *N*‐oxide product **3f** demonstrates high stereoselectivity and recyclability as a Lewis base catalyst. Additionally, product **3e** exhibits promising therapeutic efficacy against triple‐negative breast cancer, with IC_50_ values of 4.8 and 5.2 µm in MDA‐MB‐231 and MDA‐MB‐468 cells, respectively. This research not only advances the synthesis of challenging chiral heteroaromatic *N*‐oxides but also encourages further exploration of *N*‐oxide entities in the discovery of bioactive small molecules.

## Graphical abstract

This work develops a copper‐catalyzed atroposelective synthesis of axially chiral biaryl *N*‐oxides via *de novo* heteroaromatic *N*‐oxide ring formation. The mild and efficient method delivers diverse *N*‐oxide scaffolds with excellent stereoselectivities. Product **3f** demonstrates high stereoselectivity and recyclability as a Lewis base catalyst. Furthermore, *N*‐oxide **3e** shows promising anticancer activity against triple‐negative breast cancer cell lines.

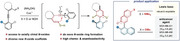

## 1 Introduction

The significance of heteroaromatic *N*‐oxides in pharmaceutical research has surged since the discovery of Minoxidil in the early 1960s. Initially recognized for its antihypertensive properties, Minoxidil later emerged as a groundbreaking treatment for alopecia, a condition characterized by hair loss due to androgenic miniaturization.^[^ ^1^ ^]^ This serendipitous breakthrough not only expanded the therapeutic reach of Minoxidil but also illuminated the extensive potential of heterocyclic *N*‐oxides in drug innovation.

The chemical structure of heterocyclic *N*‐oxides plays a crucial role in enhancing the effectiveness of drug molecules.^[^ ^2^ ^]^ It can serve as a mimic of nitric oxide (NO), a donor of NO, a bioisostere of the carbonyl group, and a hypoxic‐selective cytotoxin. Each of these functionalities provides distinct pathways for engaging with biological systems, offering a diverse range of activities such as anticancer and antibacterial properties, as well as neuroprotection.^[^ ^3^ ^]^ Furthermore, incorporating the *N*‐oxide feature into drug molecules can potentially enhance water solubility, reduce membrane permeability, and mitigate immunogenic responses.^[^ ^4^ ^]^ These attributes are essential for effective drug design and delivery, underscoring the pivotal role of heteroaromatic *N*‐oxides in shaping the future of pharmaceutical treatments.

**Figure 1:**
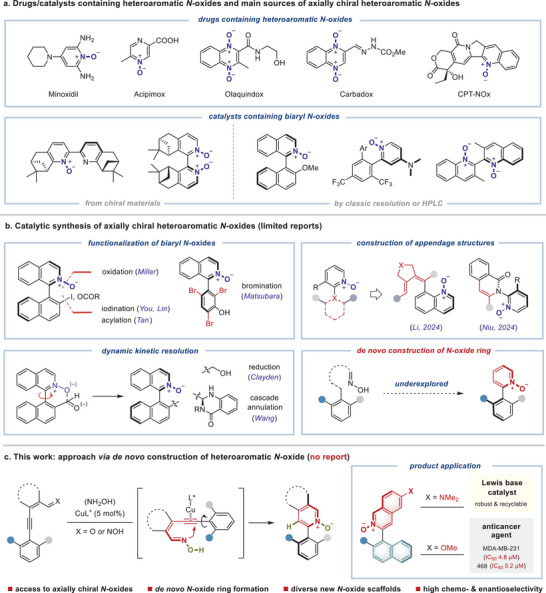
Research background and strategies for heteroaromatic *N*‐oxide synthesis. a) Drugs containing heteroaromatic *N*‐oxides and main sources of axially chiral heteroaromatic *N*‐oxides. b) Catalytic synthesis of axially chiral heteroaromatic *N*‐oxides. c) New approach via *de novo* construction of heteroaromatic *N*‐oxide (this work).

On the other hand, chiral heteroaromatic *N*‐oxides, especially those possessing axially chiral biaryl frameworks, have become indispensable in organic synthesis. Their stable molecular structures and unique Lewis basicity enable them to serve effectively as chiral organocatalysts and ligands.^[^ ^5^ ^]^ Notable examples include QUINOX, bipyridine *N,N*′‐dioxides, Me_2_PINDOX, and biquinoline *N,N*′‐dioxides, utilized in various synthetic processes such as asymmetric allylation, aldol reactions, *meso*‐epoxide opening, and cyanosilylation of aldimines (**Figure** **1**a).^[^ ^6^ ^]^ These developments underscore chiral heteroaromatic *N*‐oxides' increasing significance and utility in contemporary chemistry.^[^ ^7^ ^]^

The synthesis of axially chiral heteroaromatic nitrogen oxides represents a crucial area of research, attracting considerable attention within the scientific community. Despite its importance, challenges persist in this field, primarily due to the reliance on chiral starting materials or tedious resolution methods in existing synthetic approaches (Figure 1a).^[^ ^8^ ^]^ This underscores the need for innovative strategies to streamline the synthesis of these compounds and potentially unlock new avenues for their practical applications. Currently, only limited methods have been reported for the catalytic atroposelective synthesis of heteroaromatic *N*‐oxides (Figure 1b).^[^ ^9^ ^]^ Previous studies by You, Lin, Tan, and Miller explored kinetic resolution (KR) approaches through Pd(II)‐catalyzed C−H bond iodination, NHC‐catalyzed acylation, or peptide‐catalyzed *N*‐oxidation, respectively.^[^ ^9a–e^ ^]^ Notably, Matsubara developed a highly organocatalytic electrophilic bromination reaction for the enantioselective synthesis of axially chiral isoquinoline *N*‐oxides in 2015.^[^ ^9f^ ^]^ Subsequently, Clayden reported an intriguing biocatalytic dynamic kinetic resolution (DKR) method that utilizes bonding interactions between *N*‐oxides and aldehyde groups on the adjacent aryl ring to process biaryl isoquinoline‐*N*‐oxides.^[^ ^9g^ ^]^ Expanding on this approach, the Wang group developed an effective DKR process via a chiral phosphoric acid‐catalyzed cascade reaction.^[^ ^9h^ ^]^ Different from these strategies based on the KR and DKR of existing biaryl *N*‐oxide scaffolds, the Li and Niu groups recently achieved the efficient synthesis of axially chiral *N*‐oxides through asymmetric C−H activation and annulation reaction of heteroaromatic *N*‐oxides to construct appendage structures.^[^ ^9^, ^10^ ^]^

Despite these advances, challenges remain in the atroposelective synthesis through *de novo* construction of heteroaromatic *N*‐oxide rings. This promising approach, which could significantly expand the *N*‐oxide scaffolds, remains underexplored. Our group is dedicated to studying the synthesis and properties of novel axially chiral frameworks.^[^ ^11^ ^]^ Given the substantial research interest and the potential of *N*‐oxides in organic and medicinal chemistry, and considering the significant impact of axial chirality on drug efficacy and chiral ligands/catalysts,^[^ ^12^, ^13^ ^]^ our goal is to develop a strategy for the *de novo* heteroaromatic *N*‐oxide ring formation of biaryl *N*‐oxides via metal‐catalyzed cyclization of *ortho*‐alkynyl aryl oxime (Figure 1c).

The primary challenge in this strategy is achieving the desired reactivity, regioselectivity, and stereocontrol during cyclization. The target product has a 3‐aryl pyridine *N*‐oxide scaffold with a biaryl stereogenic axis on the pyridine ring, flanked by small substituents (a single *H*‐atom and a single *O*‐atom) at both *ortho*‐positions. To achieve an ideal atropisomer with a stable configuration suitable for catalyst and drug development (Δ*G* rotation > 30 kcal mol^−1^), introducing steric hindrance at both *ortho*‐positions of the aryl ring around the stereogenic axis is necessary. Thus, selecting an appropriate catalytic system is essential to ensure reaction activity, regioselectivity, and precise stereochemical recognition (Figure 1c).

## 2 Results

### 2.1 Reaction Development

The study commenced with an exploration of the catalytic system for the atroposelective 6‐*endo‐dig* cyclization reaction of oxime **1a**, which bears a 2‐siloxy group on the naphthalene ring, derived from 2‐(naphthalen‐1‐ylethynyl)benzaldehyde through simple condensation (**Table** **1**). Initial screenings of various catalysts revealed that AgOAc could catalyze the reaction to yield the isoquinoline‐*N*‐oxide product **3a**, while Dixon's cinchona‐alkaloid‐derived ligand **L1** failed to induce stereoselectivity (Table 1, entry 1). Pd(OAc)_2_ and Me_2_SAuCl, with corresponding phosphine ligands **L2** and **L3**, exhibited low activity in forming the heteroaromatic *N*‐oxide ring (entries 2−3). When 5 mol% of Cu(CH_3_CN)_4_PF_6_ was used as the catalyst precursor along with 6 mol% of chiral pyridine‐bis(oxazoline) **L4** in DCM, the target product **3a** was obtained in 33% yield with a 65:35 er (entry 4). Subsequent utilization of bisoxazoline (BOX) ligand **L6** led to enhanced enantioselectivity (entries 6, 14:86 er). Encouraged by this result, additional BOX ligands **L7**−**L13** were screened (entries 7−13). **L7**−**L10** yielded **3a**, but the results were no better than those obtained with **L6**. Further examination of substitutions on the methylene of BOX ligands revealed that **L11**, incorporating 4‐(*tert*‐butyl)benzyl groups, exhibited superior stereocontrol and enhanced efficiency, yielding **3a** in a 70% yield with a 94:6 er (entry 11). Interestingly, yield and enantioselectivity markedly improved when the 4‐phenyl groups of **L11** were substituted with 4‐isopropyl groups (entry 12, **L12**, 99% yield, 95.5:4.5 er). However, employing **L13**, bearing bulkier 3,5‐di‐*tert*‐ butylbenzyl groups, resulted in a dramatic decrease in yield and er of **3a** (entry 13).

**Table 1:** Condition optimization for the atroposelective 6‐*endo*‐*dig* cyclization.^a^ ^)^

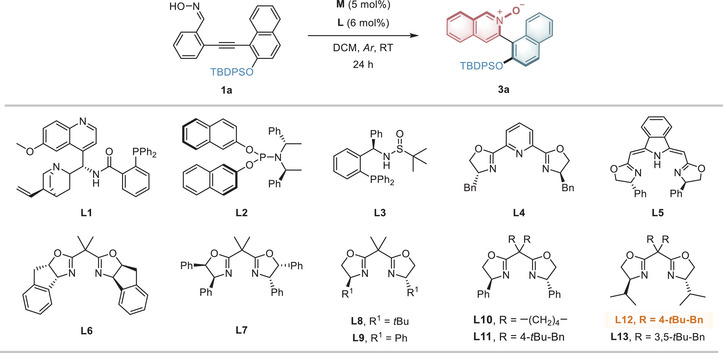				
Entry	M	L	Yield (%)	er
1	AgOAc	L1	52	50:50
2	Pd(OAc)_2_	L2	–	–
3	Me_2_SAuCl	L3	<10%	–
4	Cu(CH_3_CN)_4_PF_6_	L4	33	65:35
5	Cu(CH_3_CN)_4_PF_6_	L5	32	57.5:42.5
6	Cu(CH_3_CN)_4_PF_6_	L6	55	14:86
7	Cu(CH_3_CN)_4_PF_6_	L7	33	67.5:32.5
8	Cu(CH_3_CN)_4_PF_6_	L8	17	73:27
9	Cu(CH_3_CN)_4_PF_6_	L9	12	27:73
10	Cu(CH_3_CN)_4_PF_6_	L10	48	81.5:18.5
11	Cu(CH_3_CN)_4_PF_6_	L11	70	94:6
12	Cu(CH_3_CN)_4_PF_6_	L12	99	95.5:4.5
13	Cu(CH_3_CN)_4_PF_6_	L13	21	51.5:48.5
14	Cu(OTf)_2_	L12	72	76.5:23.5
15	Cu(CH_3_CN)_4_BF_4_	L12	91	90.5:9.5

^a)^ Reaction conditions: **1a** (0.1 mmol), metal salt **M** (5 mol%), chiral ligand **L** (6 mol%) in 1.0 mL of DCM under argon at RT for 24 h; Isolated yield; Er values were determined by chiral high‐performance liquid chromatography (HPLC) analysis.

The utilization of other copper salts, such as CuI, Cu(OTf)_2_, and Cu(CH_3_CN)_4_BF_4_, did not yield better results (see Tables S1 and S2, Supporting Information for details). Upon identifying the optimal catalyst for the atroposelective heteroaromatic *N*‐oxide ring formation, further investigation into other reaction parameters, including solvents, temperature, and concentrations, was conducted. Ultimately, the optimal reaction conditions were determined to be using Cu(CH_3_CN)_4_PF_6_ (5 mol%) as the copper source and **L12** (6 mol%) as the chiral ligand in DCM at room temperature.

### 2.2 Substrate Scope

**Figure 2:**
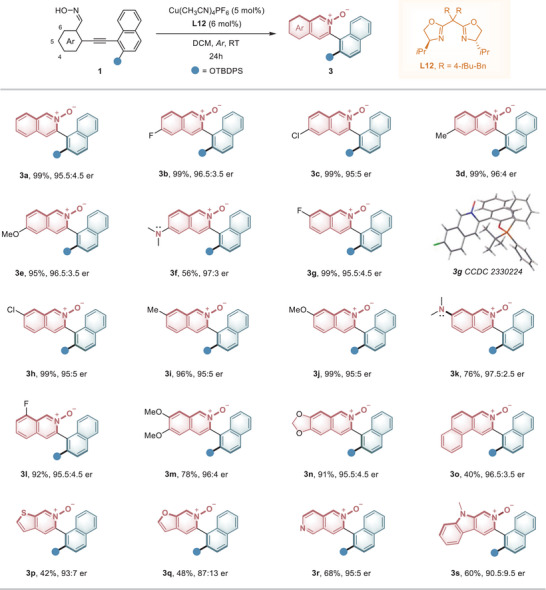
Atroposelective synthesis of heteroaromatic N‐oxides with various heteroaromatic rings. Reaction conditions: 1 (0.1 mmol), Cu(CH_3_CN)_4_PF_6_ (5 mol%), and **L12** (6 mol%) in 1.0 mL of DCM at RT for 24 h; Isolated yield; Er values were determined by HPLC analysis.

With the optimal and mild reaction conditions, we next examined the substrate scope of the atroposelective 6‐*endo*‐*dig* cyclization reaction. Initially, we investigated the applicability of aryl aldehyde oximes **1** with diverse functional groups (**Figure** **2**). Electron‐withdrawing and electron‐donating substituents at position 4 of the benzaldehyde oxime moiety were well tolerated, resulting in the formation of chiral isoquinoline *N*‐Oxides **3b**−**3e** in a highly efficient and enantioselective manner (95%−99% yields, 95:5 to 96.5:3.5 er). Notably, the 4‐dimethylamino group substituted **1f** yielded chiral 6‐(dimethylamino)isoquinoline *N*‐oxides **3f** in 56% yield with a 97:3 er. Oximes **1g**−**1k** with substituents at position 5, including halogens, alkyls, alkoxyl, and dimethylamino groups, were also compatible, yielding **3g**−**3k** with excellent yields and high enantioselectivities (95:5 to 97.5:2.5 er). The structure of the heteroaromatic *N*‐oxide product and the absolute configuration of the stereogenic axis were confirmed by X‐ray crystallography analysis of the atropisomer **3** **g** (CCDC 2330224). Notably, introducing the substituent to the *otho*‐site (position 6) of the oxime did not affect the activity and stereoselectivity of the reaction. Product **3l** bearing an 8‐F‐atom on the isoquinoline 2‐oxide scaffold was obtained in 88% yield, with 99.5:0.5 er. Additionally, disubstituted aldehyde oximes **1m** and **1n** were successfully applied, yielding the expected products in good yields with high er.

To utilize this new strategy for constructing more diverse axially chiral heteroaromatic *N*‐oxides with novel skeletons for potential catalyst and drug development, we further explored oxime substrates with different aromatic and heteroaromatic skeletons. Novel fused pyridine‐*N*‐oxides, including benzo[*f*]isoquinoline 3‐oxide, thiophene‐, and furan‐fused pyridine‐*N*‐oxides (**3o**−**3q**) were smoothly delivered under standard conditions. Remarkably, oximes with pyridine and indole moieties were well tolerated, providing interesting chiral biaryl 2,6‐naphthyridine 2‐oxide **3r** and pyrido[3,4‐*b*]indole 2‐oxide **3s** with uncompromised enantioselectivities.

**Figure 3:**
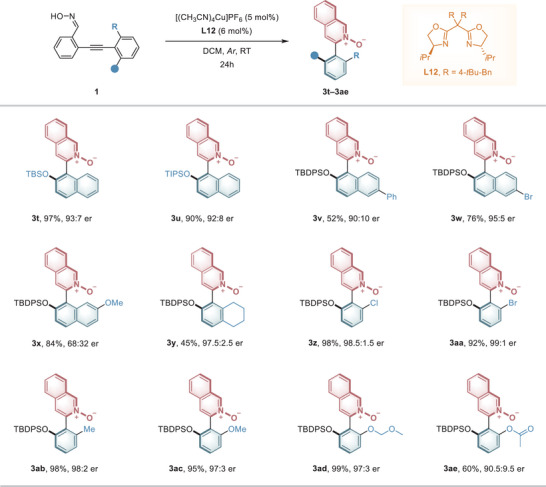
Atroposelective synthesis of heteroaromatic *N*‐oxides with various 3‐aryls. Reaction conditions: **1** (0.1 mmol), Cu(CH_3_CN)_4_PF_6_ (5 mol%), and **L12** (6 mol%) in 1.0 mL of DCM at RT for 24 h; Isolated yield; Er values were determined by HPLC analysis.

After confirming that the new strategy is highly compatible with substrates bearing a variety of oxime moieties, we further investigated the reaction's capability to produce heteroaromatic *N*‐oxides with different 3‐aryl groups (**Figure** **3**). Initially, we examined the different substituents on the naphthalene ring and found that introducing different *ortho*‐silyloxy groups, including *tert*‐butyldimethylsiloxy (TBS) and triisopropylsilyloxy (TIPS), did not significantly affect the reaction efficiency or enantioselectivity (**3t** and **3u**). Different substituents, including halogen and phenyl groups at the 6‐position of the naphthalene, were also well tolerated (**3v** and **3w**). Interestingly, when a methoxy group was introduced at the 7‐position of the naphthalene ring, there was a sharp decline in the reaction's stereoselectivity, although the reactivity remained unaffected (**3x**). To further investigate the substrate scope, we explored the reactions of oximes with different substituted phenyl groups. Replacing the naphthyl moiety with a tetrahydronaphthalene ring afforded the product **3y** with a high er of 97.5:2.5. This observation suggests that the rigid naphthalene ring and its π‐system are not essential for achieving high stereoselectivity.

We subsequently investigated whether substrates with various *ortho*‐substituted phenyl groups would retain high stereoselectivity in the reaction. Intriguingly, the findings revealed that phenyl groups substituted with diverse electron‐donating and electron‐withdrawing groups, such as *ortho*‐chloro, bromo, methyl, and methoxy, did not adversely affect the reaction's efficiency or stereoselectivity (**3z**−**3ac**, 92%−98% yields, 97:3 to 99:1 er). Moreover, functional groups such as the methoxymethoxy group and ester group proved well compatible, yielding the expected products **3ad**−**3ae** with uncompromised results (60%−99% yields, 90.5:9.5 to 97:3 er). These results highlight the exceptional versatility and broad substrate applicability of the reaction. Oxime substrates **1** bearing substituents other than a 2‐siloxy group on the naphthalene ring were also tested (see Supporting Information for details). For example, isoquinoline‐*N*‐oxide product **3af** (bearing a 2‐methoxy group on the naphthalene ring) was obtained in 91% yield, albeit with a 64:36 er. This stereogenic axis proved to be semi‐stable, with a determined rotational barrier of 28.9 kcal mol^−1^. Besides, the oxime substrate bearing an ortho Br‐atom on the naphthalene ring provided product **3ag** in excellent yield with an 84.5:15.5 er.

### 2.3 Synthetic Applications

**Figure 4:**
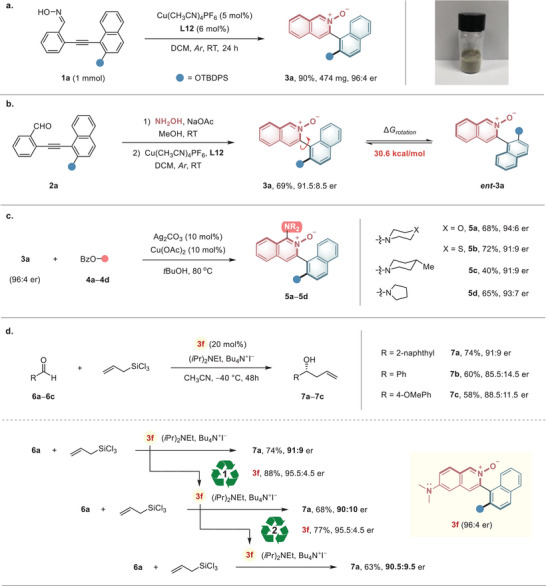
Synthetic practicality, transformations, and applications of the products. a) Scale‐up synthesis of **3a**. b) One‐pot process for heteroaromatic *N*‐oxide synthesis and rotational energy barrier of **3a**. c) Synthetic transformations of **3a**. d) Application of **3f** as a Lewis base catalyst in the asymmetric allylations of aldehydes.

After confirming the strategy's excellent universality, we further explored its practicality and utilization. A scale‐up reaction of **1a** under standard conditions was performed, affording the desired products in 90% yield with 96:4 er (**Figure** **4**a). We also developed a one‐pot condensation‐cyclization process, facilitating the streamlined and proficient synthesis of an extensive array of heteroaromatic *N*‐oxides for diverse applications (Figure 4b). Stirring aldehyde **2a** with hydroxylamine hydrochloride for 2 h cleanly generated the oxime. By simply removing the methanol and subjecting the residue to standard cyclization conditions, product **3a** was obtained with an overall yield of 69%. The conformational stability of the axially chiral *N*‐oxides **3a** was assessed via racemization experiments conducted in toluene at 100 °C. The rotational barrier of the stereogenic axis was determined to be 30.6 kcal mol^−1^, rendering it unlikely to interconvert and a suitable scaffold for catalyst and drug development. Various amino groups could be conveniently introduced to the 1‐position of the chiral isoquinoline *N*‐Oxide **3a** through copper(II)‐catalyzed amination with *O*‐benzoyl hydroxylamines (Figure 4c). The amination products **5a**−**5d** containing morpholine, thiomorpholine, piperidine, and pyrrolidine were afforded with 91:9 to 93:7 er.

Having established access to heteroaromatic *N*‐oxides with varied scaffolds and diverse substitution patterns, we initially explored the use of these novel *N*‐oxides as Lewis base catalysts. Remarkably, the isoquinoline *N*‐oxides **3f** bearing a 6‐dimethylamino group turned out to be an efficient Lewis base catalyst for the asymmetric allylation of aldehydes (Figure 4d). Using **3f** as a catalyst in the reaction of different aldehydes (**6a**−**6c**) with allyltrichlorosilane in acetonitrile, good yields and enantiomeric ratios of up to 91:9 were immediately obtained. Due to the catalyst's excellent structural and configurational stability, we attempted to recycle it in the allylation reaction. We were pleased to find that catalyst **3f**, which had been recycled and reused twice, continued to catalyze the reaction efficiently, yielding compound **7a** with a 63% yield and an er of 90.5:9.5. Notably, the recycled catalyst maintained a consistently high er value. This result underscores the potential of the new framework as a recyclable and highly stereoselective Lewis base catalyst.

### 2.4 Biological Evaluation

**Figure 5:**
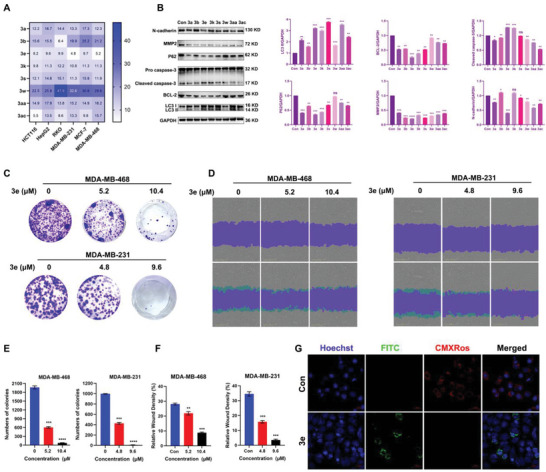
Evaluation of the antitumor activity of heteroaromatic *N*‐oxides. a) The MTT assay was employed to determine the IC_50_ values of the derivatives in tumor cells. b) WB experiment was conducted to evaluate the impact of the derivatives on cell apoptosis‐related proteins and tumor metastasis‐related proteins in MDA‐MB‐231 cells. GAPDH was used as a loading control. Quantitative and representative images of the expression levels of relevant proteins are shown. c) Colony formation assay was utilized to assess **3e**’s effect on proliferation in both MDA‐MB‐231 and MDA‐MB‐468 cells. d) Cell scratch assay was performed to evaluate **3e**’s influence on migration in both MDA‐MB‐231 and MDA‐MB‐468 cells, scale bar, 400 µm. e,f) Quantitative results for c and d are presented. g) **3e** induces apoptosis in MDA‐MB‐231 cells, scale bar, 20 µm. Data are presented as the mean ± SEM. These results are consistent with those of at least three different experiments. ns, not significant, *, *p* < 0.05, **, *p* < 0.01, ***, *p* < 0.001, ****, *p* < 0.0001 Statistical significance was determined relative to the appropriate control groups.

Given the reported remarkable pharmacological activities of heteroaromatic *N*‐oxides, including anticancer and antibacterial properties, we conducted a series of biological assays to evaluate the new heteroaromatic *N*‐oxide products' biological activity and pharmacological potential.^[^ ^2^, ^4^ ^]^ Initially, we assessed the antitumor efficacy of these compounds through MTT (methylthiazolyldiphenyl‐tetrazolium bromide) experiments (**Figure** **5**a and Figure S2a, Supporting Information). These compounds exhibited significant antitumor activity against various tumor types, with methoxy‐substituted isoquinoline‐*N*‐oxide **3e** particularly noteworthy, showing IC_50_ values of 4.8 and 5.2 µm in triple‐negative breast cancer cells MDA‐MB‐231 and MDA‐MB‐468, respectively.

Programmed cell death induction in tumor cells is a common mechanism antitumor drugs employ. To delve into the underlying mechanisms of these heteroaromatic *N*‐ oxides' antitumor effects, we conducted western blot (WB) experiments to detect key proteins involved in apoptotic pathways (Caspase‐3, BCL‐2), autophagy pathways (LC3, P62), and tumor metastasis (*N*‐cadherin, MMP2) in MDA‐MB‐231 and MDA‐MB‐468 cells.^[^ ^14^ ^]^ The results revealed that these compounds, particularly **3e**, significantly enhanced cellular apoptosis and autophagy while inhibiting tumor metastasis (Figure 5b and Figure S1b, Supporting Information). Consequently, **3e** was selected for further validation of its antitumor activity.

We then evaluated the anti‐proliferative capabilities of **3e** against tumors through cloning experiments (Figure 5c). It significantly inhibited tumor proliferation in both cell lines in a dose‐dependent manner. Additionally, the cell scratch assay indicated that *N*‐ oxide **3e** possesses remarkable anti‐metastatic properties (Figure 5d). Changes in mitochondrial membrane potential and phosphatidylserine externalization are key indicators of cellular apoptosis.^[^ ^15^ ^]^ Therefore, we utilized the mitochondrial membrane potential kit to assess **3e**’s ability to induce tumor cell apoptosis. Viable cells were labeled with Mito‐Tracker Red CMXRos, a red fluorescence probe dependent on mitochondrial membrane potential, resulting in red fluorescence‐positive cells. Apoptotic cells were stained with Annexin V‐FITC green fluorescence probe, showing green fluorescence positivity with significantly reduced or absent red fluorescence signal. We found that **3e** effectively promoted cellular apoptosis, consistent with the results from the western blot analysis (Figure 5g). These findings suggest that this series of heteroaromatic *N*‐oxides exhibits significant antitumor activity, with **3e** showing particular promise for development as a therapeutic agent for breast cancer.

### 2.5 Reaction Mechanism

**Figure 6:**
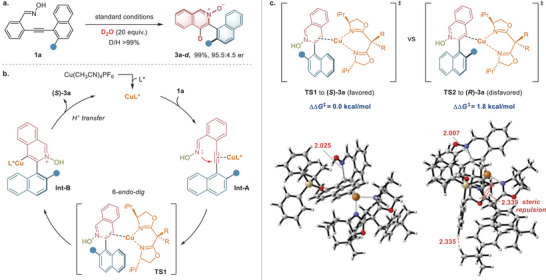
Study of reaction mechanism and stereocontrol models. a) Deuterium‐labeling experiment of the model reaction. b) Proposed mechanism for the Cu‐catalyzed *de novo* heteroaromatic *N*‐oxide ring formation. c) DFT‐optimized structures and relative free Gibbs energies for the enantiomeric transition states **TS1** and **TS2**. Density functional theory (DFT) calculations were carried out at the SMD(DCM)‐M06/def2‐TZVP//SMD(DCM)‐M06/6‐31G(d)/LANL2DZ(Cu) level of theory.

Next, we investigated the reaction mechanism through deuterium‐labeling experiments in the Cu‐catalyzed process. Under standard conditions, adding D_2_O to the reaction did not affect the conversion and led to the generation of deuterated **3a** (**Figure** **6**a). This indicates the formation of alkenyl copper species. Based on the experiment, we proposed the reaction mechanism and possible transition states for the atroposelective heteroaromatic *N*‐oxide ring formation (Figure 6b). In the catalytic cycle, the reaction initiates with the π‐activation of the alkyne by the in situ generated copper catalyst to form intermediate **Int‐A**, increasing electrophilicity and facilitating the subsequent 6‐*endo*‐*dig* cyclization. The ring closure via transition state **TS1** would produce the axially chiral (hydroxyisoquinolinium‐4‐yl)copper intermediate **Int‐B**. Subsequent protonation of the alkenyl copper species **Int‐B** leads to the formation of (*S*)−**3a** and regenerates the catalyst.

To elucidate the origin of enantioselectivity using **L12**‐coordinated Cu(I) complex, we conducted DFT calculations on the cyclization step, which is decisive for the configuration and enantioselectivity of the axially chiral heteroaromatic *N*‐oxide (see Supporting Information for details). As shown in Figure 6c, the relative free Gibbs energy (Δ*G*) of transition state **TS1** (leading to the major enantiomer (*S*)−**3a**) is calculated to be 1.8 kcal mol^−1^ lower than that of **TS2** (leading to the minor enantiomer (*R*)−**3a**). The theoretical er value was 90.9:9.1 based on the Boltzmann distribution,^[^ ^16^ ^]^ which was in accordance with experimental results (95.5:4.5). Inspection of the transition‐state structures shows that the TBDPS group and Ar groups on the **L12** ligand have a very short distance in the structure of **TS2**, which leads to significant steric repulsion and lower stability. Therefore, the observed enantioselectivity is attributed to the stereo‐hindrance effect. In the stereocontrol models of Cu‐catalyzed asymmetric 6‐ *endo*‐*dig* ring closure, **TS1** is favored, avoiding steric repulsion between the bulky siloxy group on the naphthalene and the isopropyl substituent of the BOX ligand.

## 3 Conclusion

In summary, we developed a new strategy for the atroposelective synthesis of biaryl heteroaromatic *N*‐oxides through *de novo* construction of heteroaromatic *N*‐oxide rings. The Cu‐catalyzed *de novo* heteroaromatic *N*‐oxide ring formation allows the efficient construction of various novel *N*‐oxide scaffolds, achieving high yields and excellent enantioselectivity. Axially chiral isoquinoline *N*‐oxides **3f** bearing a 6‐dimethylamino group turned out to be an efficient and recyclable Lewis base catalyst for the asymmetric allylation of aldehydes. Product **3e** exhibited IC_50_ values of 4.8 and 5.2 µm in triple‐negative breast cancer cells MDA‐MB‐231 and MDA‐MB‐468, showing potential for development as a therapeutic agent for breast cancer. This work advances the synthetic capabilities in the field of heteroaromatic *N*‐oxides and lays the groundwork for further exploration and application of these compounds in asymmetric catalysis, drug development, and other chemical areas.

## 4 Experimental Section

### General Procedure for the Synthesis of Isoquinoline *N*‐Oxides 3

To a 10 mL Schlenk tube under an argon atmosphere, Cu(CH_3_CN)_4_PF_6_ (1.9 mg, 5 mol%), **L12** (3.2 mg, 6 mol%), and dry DCM (0.4 mL) were added. The mixture was stirred at room temperature for 30 min. Subsequently, **1** (0.1 mmol) in dry DCM (0.6 mL) was added. The reaction mixture was stirred for 24 h, monitored by TLC, and then directly purified by silica gel column chromatography to yield compound **3**.

### Cell Cultures

The HCT116, HepG2, RKO, MDA‐MB‐231, MCF‐7 and MDA‐MB‐468 cells utilized in this study were procured from American Type Culture Collection (Manassas, VA, USA), and were cultured in GibcoTM Dulbecco's Modified Eagle Medium (DMEM) supplemented with 10% fetal bovine serum (FBS) and incubated in an incubator maintained at 37 °C with 95% air and 5% CO_2_.

### Antibodies

The MTT was purchased from Solarbio (M8180). Mitochondrial membrane potential and apoptosis detection kit (C1071S, Beyotime) and crystal violet staining solution (C0121, Beyotime) were purchased from Beyotime (Shanghai, China). The following antibodies were applied to this study: GAPDH (ab8245, Abcam), Bcl‐2 (ER0602, HuaBio), LC3 (ab192890, Abcam), MMP2 (ab92536, Abcam), N‐cadherin (22018‐1‐AP, Proreintech), P62 (ab109012, Abcam), Caspase‐3 (TA6311, Abmart).

### Cellular Toxicity Assay

The cells were seeded in a 96‐well plate at a density of 5 × 10^3^ cells well^−1^ and incubated in a cell culture incubator for 24 h prior to drug treatment. The concentrations of the compounds utilized were 100, 50, 25, 12.5, 6.25, 3.12, and 1.56 µm. After treatment, 0.5 mg mL^−1^ of MTT was added to each well, and the cells were subsequently incubated at 37 °C for 4 h. Then the medium was removed, and dimethyl sulfoxide (DMSO) was added to dissolve the formazan crystals at 37 °C for 10 min. Finally, the absorbance of the formation dye was measured at 490 nm using a microplate reader (BioTek, USA).

### Colony Formation and Cell‐Migration Assays

For the cloning formation experiment, 1000 cells were seeded in a 6‐well plate and incubated in an incubator for 24 h, and were subjected to continuous treatment with a compound for a duration of 2 weeks. Following this, the culture medium was aspirated and the wells were washed thrice with PBS. Each well was then fixed by adding 1 mL of methanol for a period of 15 min, after which the methanol was discarded and further washing with PBS was performed three times. Finally, a crystal violet solution was utilized to stain the cells. Wound healing assay was performed to determine the cell migration ability; the experiment was conducted using Incucyte Live‐Cell Analysis. Briefly, once the cell confluence in a 96‐well plate reached 90–100%, a 96‐Well WoundmakerTool (Sartorius, 4563) was utilized to generate scratches, followed by treatment of the cells with compounds and imaging every 4 h.

### Western Blotting Analysis

The western blot assay was performed under ice‐cold conditions throughout the entire process. First, cells were lysed using RIPA lysis buffer (EA0002, Shandong Sparkjade Biotechnology Co., Ltd.) supplemented with a proteinase inhibitor (EA0006, Shandong Sparkjade Biotechnology Co., Ltd.), followed by centrifugation of the cell lysate at 12 000 rpm and 4 °C for 15 min. Subsequently, protein quantification was carried out utilizing the BCA assay kit (EC0001‐A, Shandong Sparkjade Biotechnology Co., Ltd.). A total of 30 µg of protein was then separated by SDS‐PAGE and transferred onto PVDF membranes. The membranes were first incubated with 5% skimmed milk, followed by protein‐specific primary antibodies, and finally with HRP‐conjugated secondary antibodies. The membranes were subsequently incubated with the HRP substrate for visualization using enhanced chemiluminescence (ECL). Densitometric analysis of protein bands was conducted using the ImageJ software.

### Cellular Apoptosis Assay

The evaluation of cell apoptosis was conducted using the Mitochondrial Membrane Potential Detection Kit (C1071; Beyotime), following the manufacturer's instructions. Briefly, 188 µL of Annexin V‐FITC binding solution and 5 µL of Annexin V‐FITC were gently mixed with the processed cells. Subsequently, 2 µL of Mito‐Tracker Red CMXRos staining solution and 5 µL of Hoechst 33 342 staining solution were added. Finally, the images were captured utilizing an LSM800 microscope (Zeiss, GER) and analyzed employing ZEN software.

### Statistical Analysis

All the presented data and results were confirmed by at least three independent experiments. The data are expressed as means ± SEM and analyzed with GraphPad Prism 8.0 software. Data preprocessing for western blot quantification involved normalized quantification. Statistical comparisons were made using one‐way ANOVA and Student's *t*‐test. Statistical significance was defined as *p* < 0.05, with “ns” representing non‐significant results.

## Conflict of Interest

The authors declare no conflict of interest.

## Supporting information

Supporting Information

## Data Availability

The data that support the findings of this study are available in the supplementary material of this article.
