# Supplementary material for: Atroposelective Synthesis of Biaryl N‐Oxides via Cu‐Catalyzed De Novo Heteroaromatic N‐Oxide Ring Formation
Source: Adv Sci (Weinh). 2024 Aug 9;11(40):2405743. doi: 10.1002/advs.202405743 (PMC11515923; doi:10.1002/advs.202405743)
Supplement: Supplementary file 1 — Supporting Information [file ADVS-11-2405743-s001.pdf]

ADVANCED SCIENCE
Open Access

Supporting Information

for *Adv. Sci.*, DOI 10.1002/advs.202405743

Atroposelective Synthesis of Biaryl *N*-Oxides via Cu-Catalyzed De Novo Heteroaromatic *N*-Oxide Ring Formation

Ke Ma, Ting Qi, Lei Hu, Chen Chen, Wan Wang, Jun-Long Li, Cheng Peng, Gu Zhan and Bo Han**

ADVANCED SCIENCE
Open Access

Supporting Information

Atroposelective Synthesis of Biaryl *N*-Oxides via Cu-Catalyzed De Novo Heteroaromatic *N*-Oxide Ring Formation

Ke Ma, † *Ting Qi,* † *Lei Hu, Chen Chen, Wan Wang, Jun-Long Li, Cheng Peng, Gu Zhan,* Bo Han**

Supporting Information

Atroposelective synthesis of biaryl *N*-oxides *via* Cu-catalyzed *de novo* heteroaromatic *N*-oxide ring formation

Ke Ma,[1†] Ting Qi,[2†] Lei Hu,[1,3] Chen Chen,[1] Wan Wang,[1] Jun-Long Li,[2] Cheng Peng,[1] Gu Zhan,[1]* Bo Han[1]*

[1]*State Key Laboratory of Southwestern Chinese Medicine Resources, Hospital of Chengdu University of Traditional Chinese Medicine, School of Pharmacy, Chengdu University of Traditional Chinese Medicine, Chengdu 611137, China.*

[2]*Anti-Infective Agent Creation Engineering Research Centre of Sichuan Province, Sichuan Industrial Institute of Antibiotics, School of Pharmacy, Chengdu University, Chengdu, 610106, China.*

[3]*Department of Biotherapy, Cancer Center and State Key Laboratory of Biotherapy, West China Hospital, Sichuan University, Chengdu, 610041, China.*

Email: Gu Zhan: zhangu@cdutcm.edu.cn; Bo Han: hanbo@cdutcm.edu.cn

Contents

1. General information S1

2. Optimization of the model reaction S2

3. General procedure for the synthesis of substrates 1 .... S4

4. General procedure for the synthesis of heteroaromatic *N*-oxides 3 .... S11

5. Scale-up reaction and transformations of the product S24

6. Single crystal X-ray diffraction analysis and crystal data S30

7. Thermal stability experiments S32

8. DFT calculations S35

9. Evaluation of the antitumor activity of heteroaromatic *N*-oxides S44

10. NMR spectra and HPLC chromatograms S45

11. References S148

1. General information

Enantiomeric ratios were determined by HPLC analysis on an Agilent 1260 Infinity II using chiral columns in comparison with authentic racemates. Chiral columns, Daicel Chiralpak AD-H Column (250 × 4.6 mm), Daicel Chiralpak OD-H Column (250 × 4.6 mm), Daicel Chiralpak OJ-H Column (250 × 4.6 mm), Daicel Chiralpak IA Column (250 × 4.6 mm), Daicel Chiralpak IC Column (250 × 4.6 mm), and Daicel Chiralpak ID Column (250 × 4.6 mm) were used. UV detection was performed at 254 nm. Nuclear magnetic resonance (NMR) spectra were recorded in DMSO-d_6 and $CDCl_3$ on Bruker 600, 700 MHz, or JEOL 600 NMR MR instrument for ^{1}H, and at 150 or 175 MHz for ^{13}C. Proton chemical shifts are reported in parts per million (δ scale). The ^{1}H NMR chemical shifts are reported in ppm with the internal TMS signal at 0.0 ppm as standard. The ^{13}C NMR chemical shifts were given by using DMSO-d_6 and $CDCl_3$ as the internal standard (DMSO-d_6: δ = 39.52 ppm, $CDCl_3$: δ = 77.16 ppm). ^{19}F NMR chemical shifts were given by using DMSO-d_6 and $CDCl_3$. Data are reported as follows: chemical shift [multiplicity (s = singlet, d = doublet, t = triplet, q = quartet, m = multiplet, td = triplet of doublets, dt = doublet of triplets), coupling constant(s) (Hz), integration]. High-resolution mass spectra (HRMS) were obtained using Agilent P/N G1969-90010. High-resolution mass spectra were reported for the molecular ion $[M + Na]^+$ or $[M + H]^+$. X-ray diffraction experiment was carried out on an Agilent Gemini and the data obtained were deposited at the Cambridge Crystallographic Data Centre. Column chromatography was performed on silica gel (200-300 mesh) using an eluent of ethyl acetate (EA) and petroleum ether (PE). TLC was performed on glass-backed silica plates; products were visualized using UV light (254 nm). Optical rotation values were measured with instruments operating at λ = 589 nm, corresponding to the sodium D line at 30 °C. Melting points were recorded on BUCHI Melting Point M-565 instrument. Unless otherwise noted, all reagents were obtained commercially and used without further purification. All heating sources are oil baths.

2. Optimization of the model reaction

Table S1. Screening of ligands.[a]

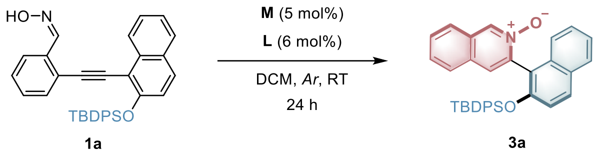

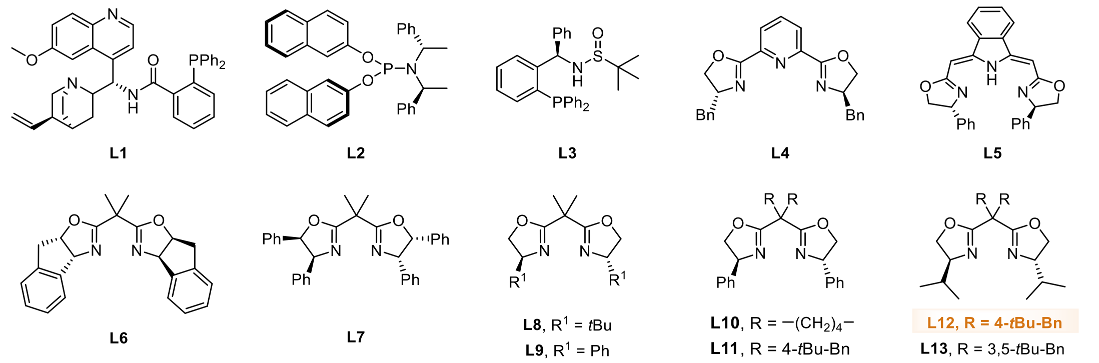

Entry	M	L	Yield (%)	er
1	AgOAc	**L1**	52	50:50
2	$Pd(OAc)_2$	**L2**	-	-
3	Me_2SAuCl	**L3**	<10%	-
4	$Cu(CH_3CN)_4PF_6$	**L4**	33	65:35
5	$Cu(CH_3CN)_4PF_6$	**L5**	32	57.5:42.5
6	$Cu(CH_3CN)_4PF_6$	**L6**	55	14:86
7	$Cu(CH_3CN)_4PF_6$	**L7**	33	67.5:32.5
8	$Cu(CH_3CN)_4PF_6$	**L8**	17	73:27
9	$Cu(CH_3CN)_4PF_6$	**L9**	12	27:73
10	$Cu(CH_3CN)_4PF_6$	**L10**	48	81.5:18.5
11	$Cu(CH_3CN)_4PF_6$	**L11**	70	94:6
**12**	**$Cu(CH_3CN)_4PF_6$**	**L12**	**99**	**95.5:4.5**
13	$Cu(CH_3CN)_4PF_6$	**L13**	21	51.5:48.5

[a)] Reaction conditions: **1a** (0.1 mmol), metal salt **M** (5 mol%), chiral ligand **L** (6 mol%) in 1.0 mL of DCM under argon at RT for 24 h; Isolated yield; Er values were determined by chiral high-performance liquid chromatography (HPLC) analysis.

Table S2. Screening of copper source and other conditions.[a]

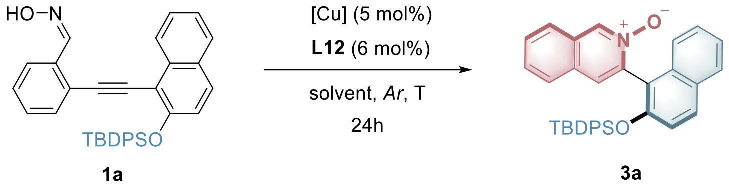

Entry	M	Sol	T	C(mmol/ml)	Yield(%)	er
1	CuI	DCM	RT	0.1 mmol	NR	ND
2	Cu_2O	DCM	RT	0.1 mmol	NR	ND
3	CuTC	DCM	RT	0.1 mmol	NR	ND
4	$Cu(acac)_2$	DCM	RT	0.1 mmol	NR	ND
5	$Cu(OAc)_2$	DCM	RT	0.1 mmol	NR	ND
6	$Cu(OTf)_2$	DCM	RT	0.1 mmol	72	76.5:23.5
7	$Cu(CH_3CN)_4BF_4$	DCM	RT	0.1 mmol	91	90.5:9.5
8	$Cu(CH_3CN)_4PF_6$	DCE	RT	0.1 mmol	93	93.5:6.5
9	$Cu(CH_3CN)_4PF_6$	$CHCl_3$	RT	0.1 mmol	85	93.5:6.5
10	$Cu(CH_3CN)_4PF_6$	Toluene	RT	0.1 mmol	72	89.5:10.5
11	$Cu(CH_3CN)_4PF_6$	CH_3CN	RT	0.1 mmol	34	86.5:13.5
12	$Cu(CH_3CN)_4PF_6$	THF	RT	0.1 mmol	74	96:4
13	$Cu(CH_3CN)_4PF_6$	DCM	0°C	0.1 mmol	72	96.5:3.5
14	$Cu(CH_3CN)_4PF_6$	DCM	40°C	0.1 mmol	81	92:8
15	$Cu(CH_3CN)_4PF_6$	DCM	RT	0.05mmol	91	95.5:4.5
16	$Cu(CH_3CN)_4PF_6$	DCM	RT	0.2mmol	97	95.5:4.5
17[b]	$Cu(CH_3CN)_4PF_6$	DCM	RT	0.1 mmol	99	95:5
18[c]	$Cu(CH_3CN)_4PF_6$	DCM	RT	0.1 mmol	91	96:4
19[d]	$Cu(CH_3CN)_4PF_6$	DCM	RT	0.1 mmol	95	95:5
20[e]	$Cu(CH_3CN)_4PF_6$	DCM	RT	0.1 mmol	93	95:5

[a] Reaction conditions: **1a** (0.1 mmol), Cu salt (5 mol%), **L12** (6 mol%) in solvent under argon for 24 h; Isolated yield; Er values were determined by chiral HPLC analysis. [b] 4Å MS was added. [c] $MgSO_4$ was added. [d] Na_2SO_4 was added. [e] [Cu] (10 mol%) and **L12** (12 mol%) was used.

3. General procedure for the synthesis of substrates 1

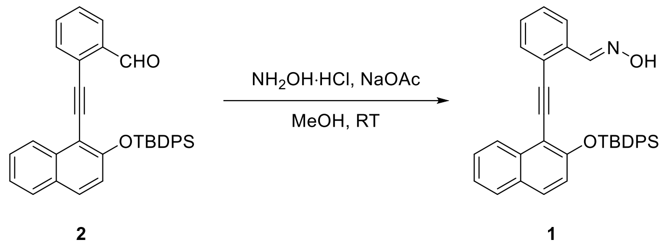

2 (1.96 mmol,1.0 equiv.) was dissolved in MeOH (10 mL), $NH_2OH{\cdot}HCl$ (3.92 mmol, 2.0 equiv.) was added to the stirred solution, followed by NaOAc (3.92 mmol, 2.0 equiv.). The reaction was monitored by TLC. After 1 to 2 h, the reaction mixture was diluted with EA (50 mL) and H_2O (50 mL). Phases were separated and the aqueous phase was extracted twice with EA (50 mL). The combined organic phases were dried over Na_2SO_4, filtered and concentrated under reduced pressure. The crude product was purified by column chromatography on silica gel (PE: EA=30:1-10:1) to obtain the product **1**.[1]

(*E*)-2-((2-((*tert*-butyldiphenylsilyl)oxy)naphthalen-1-yl)ethynyl)benzaldehyde oxime (1a)

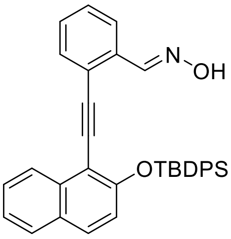

^{1}H NMR (600 MHz, Chloroform-*d*) δ 8.86 (s, 1H), 8.25 (d, *J* = 8.4 Hz, 1H), 7.85 (dd, *J* = 7.8, 1.8 Hz, 1H), 7.73 (dd, *J* = 8.4, 1.8 Hz, 4H), 7.66 (s, 1H), 7.61 (d, *J* = 7.8 Hz, 1H), 7.57 (dd, *J* = 7.8, 1.8 Hz, 1H), 7.51 – 7.47 (m, 1H), 7.37 – 7.32 (m, 3H), 7.32 – 7.25 (m, 7H), 6.68 (d, *J* = 9.0 Hz, 1H), 1.09 (s, 9H). **^{13}C NMR** (150 MHz, Chloroform-*d*) δ 155.7, 149.6, 135.6, 134.8, 133.2, 132.83, 132.76, 130.2, 129.9, 129.7, 128.8, 128.5, 128.3, 128.0, 127.5, 125.45, 125.43, 124.5, 124.1, 120.3, 108.6, 95.4, 90.8, 26.7, 19.9. **HRMS** (ESI-TOF) m/z: $[M + H]^+$ Calcd for $C_{35}H_{32}NO_2Si^+$ 526.2197; Found 526.2202.

(*E*)-2-((2-((*tert*-butyldiphenylsilyl)oxy)naphthalen-1-yl)ethynyl)-4-chlorobenzaldehyde oxime (1c)

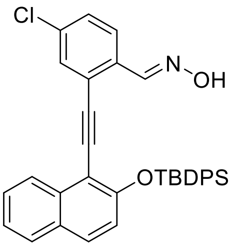

^{1}H NMR (600 MHz, Chloroform-*d*) δ 8.76 (s, 1H), 8.19 (dd, *J* = 8.4, 1.2 Hz, 1H), 7.78 (d, *J* = 8.4 Hz, 1H), 7.74 – 7.70 (m, 4H), 7.64 – 7.60 (m, 2H), 7.52 – 7.47 (m, 2H), 7.39 (d, *J* = 9.0 Hz, 1H), 7.37 – 7.33 (m, 2H), 7.31 – 7.26 (m, 5H), 7.25 (dd, *J* = 8.4, 2.4 Hz, 1H), 6.70 (d, *J* = 9.0 Hz, 1H), 1.09 (s, 9H). **^{13}C NMR** (150 MHz, Chloroform-*d*) δ 156.1, 148.6, 135.6, 134.7, 132.7, 132.3, 131.6, 130.4, 130.3, 128.8, 128.7, 128.3, 128.09, 128.07, 127.6, 126.7, 125.4,

125.3, 124.6, 120.4, 108.0, 94.1, 92.1, 26.7, 19.9. **HRMS** (ESI-TOF) m/z: $[M + Na]^+$ Calcd for $C_{35}H_{30}ClNNaO_2Si^+$ 582.1627; Found 582.1626.

(*E*)-2-((2-((*tert*-butyldiphenylsilyl)oxy)naphthalen-1-yl)ethynyl)-4-methoxybenzaldehyde oxime (1e)

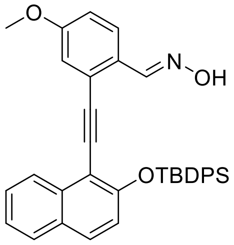

^{1}H NMR (600 MHz, Chloroform-*d*) δ 8.81 (s, 1H), 8.26 (d, *J* = 8.4 Hz, 1H), 7.78 (d, *J* =9.0 Hz, 1H), 7.73 (dd, *J* = 7.8, 1.2 Hz, 4H), 7.61 (d, *J* = 7.8 Hz, 1H), 7.52 – 7.48 (m, 1H), 7.38 – 7.26 (m, 9H), 7.08 (d, *J* = 2.4 Hz, 1H), 6.87 (dd, *J* = 9.0, 3.0 Hz, 1H), 6.67 (d, *J* = 9.0 Hz, 1H), 3.76 (s, 3H), 1.10 (s, 9H). **^{13}C NMR** (150 MHz, Chloroform-*d*) δ 160.6, 155.8, 149.2, 135.6, 134.8, 132.7, 130.2, 130.0, 128.8, 128.3, 128.1, 127.5, 126.9, 126.1, 125.4, 125.3, 124.5, 120.3, 116.3, 116.2, 108.4, 95.4, 90.6, 55.6, 26.7, 19.9. **HRMS** (ESI-TOF) m/z: $[M + Na]^+$ Calcd for $C_{36}H_{33}NNaO_3Si^+$ 578.2122; Found 578.2127.

(*E*)-2-((2-((*tert*-butyldiphenylsilyl)oxy)naphthalen-1-yl)ethynyl)-5-(dimethylamino)benzaldehyde oxime (1k)

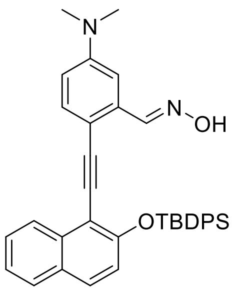

^{1}H NMR (600 MHz, Chloroform-*d*) δ 8.85 (s, 1H), 8.28 (d, *J* = 7.8 Hz, 1H), 7.74 (dd, *J* = 7.8, 1.2 Hz, 4H), 7.61 – 7.52 (m, 2H), 7.49 – 7.44 (m, 2H), 7.36 – 7.32 (m, 2H), 7.31 (d, *J* = 9.0 Hz, 1H), 7.30 – 7.26 (m, 5H), 7.13 (d, *J* = 2.4 Hz, 1H), 6.70 (dd, *J* = 9.0, 3.0 Hz, 1H), 6.66 (d, *J* = 9.0 Hz, 1H), 2.97 (s, 6H), 1.10 (s, 9H). **^{13}C NMR** (150 MHz, Chloroform-*d*) δ 155.1, 150.3, 150.0, 135.7, 134.9, 133.8, 133.7, 133.01, 132.99, 130.1, 129.1, 128.8, 128.1, 128.0, 127.2, 125.7, 124.3, 120.4, 114.2, 111.8, 109.5, 107.6, 96.6, 88.2, 40.4, 26.7, 19.9. **HRMS** (ESI-TOF) m/z: $[M + Na]^+$ Calcd for $C_{37}H_{36}N_2NaO_2Si^+$ 591.2439; Found 591.2431.

(*E*)-6-((2-((*tert*-butyldiphenylsilyl)oxy)naphthalen-1-yl)ethynyl)benzo[d][1,3]dioxole-5-carbaldehyde oxime (1n)

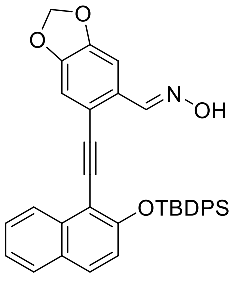

^{1}H NMR (600 MHz, Chloroform-*d*) δ 8.80 (s, 1H), 8.21 (dd, *J* = 8.4, 1.2 Hz, 1H), 7.74 – 7.71 (m, 4H), 7.60 (d, *J* = 8.4 Hz, 1H), 7.55 – 7.46 (m, 2H), 7.37 – 7.33 (m, 3H), 7.31 – 7.25 (m, 6H), 6.94 (s, 1H), 6.68 (d, *J* = 9.0 Hz, 1H), 5.96 (s, 2H), 1.09 (s, 9H). **^{13}C NMR** (150 MHz, Chloroform-*d*) δ 155.7, 149.3, 149.1, 148.5, 135.6, 134.8, 132.8, 130.2, 129.8, 128.8, 128.5, 128.2, 128.0, 127.5, 125.4, 124.5, 120.3, 118.6, 111.6, 108.6, 104.8, 101.9,

95.3, 89.6, 26.7, 19.9. **HRMS** (ESI-TOF) m/z: $[M + Na]^+$ Calcd for $C_{36}H_{31}NNaO_4Si^+$ 592.1915; Found 592.1925.

(*E*)-1-((2-((*tert*-butyldiphenylsilyl)oxy)naphthalen-1-yl)ethynyl)-2-naphthaldehyde oxime (1o)

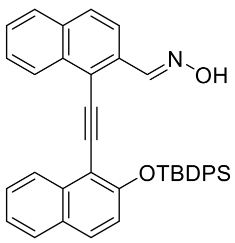

^{1}H NMR (600 MHz, Chloroform-*d*) δ 9.08 (s, 1H), 8.60 (d, *J* = 7.8 Hz, 1H), 8.33 (d, *J* = 8.4 Hz, 1H), 7.97 (d, *J* = 8.4 Hz, 1H), 7.80 (dd, *J* = 7.2, 1.8 Hz, 1H), 7.77 (d, *J* = 8.4 Hz, 1H), 7.74 (dd, *J* = 7.8, 1.2 Hz, 4H), 7.64 (d, *J* = 7.8 Hz, 1H), 7.51 – 7.45 (m, 4H), 7.41 (d, *J* = 9.0 Hz, 1H), 7.35 – 7.29 (m, 3H), 7.24 (t, *J* = 7.8 Hz, 4H), 6.75 (d, *J* = 9.0 Hz, 1H), 1.02 (s, 9H). **^{13}C NMR** (150 MHz, Chloroform-*d*) δ 155.9, 150.3, 135.7, 134.9, 133.9, 133.6, 132.9, 131.9, 130.2, 130.1, 128.81, 128.78, 128.3, 128.0, 127.58, 127.57, 127.5, 127.4, 125.5, 124.5, 122.3, 122.2, 120.5, 108.6, 96.2, 93.2, 26.7, 19.8. **HRMS** (ESI-TOF) m/z: $[M + Na]^+$ Calcd for $C_{39}H_{33}NNaO_2Si^+$ 598.2173; Found 598.2178.

(*E*)-3-((2-((*tert*-butyldiphenylsilyl)oxy)naphthalen-1-yl)ethynyl)furan-2-carbaldehyde oxime (1q)

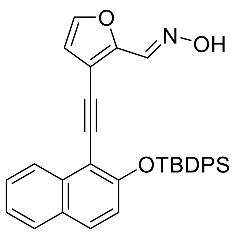

^{1}H NMR (600 MHz, Chloroform-*d*) δ 8.28 (s, 1H), 8.20 (d, *J* = 8.4 Hz, 1H), 7.93 (s, 1H), 7.71 (dd, *J* = 7.8, 1.2 Hz, 4H), 7.61 (d, *J* = 8.4 Hz, 1H), 7.50 – 7.47 (m, 1H), 7.41 (d, *J* = 1.8 Hz, 1H), 7.37 – 7.33 (m, 3H), 7.31 – 7.27 (m, 5H), 6.66 (d, *J* = 9.6 Hz, 1H), 6.54 (d, *J* = 1.8 Hz, 1H), 1.10 (s, 9H). **^{13}C NMR** (150 MHz, Chloroform-*d*) δ 155.8, 148.7, 144.1, 139.3, 135.6, 134.6, 132.7, 130.2, 130.0, 128.7, 128.3, 128.1, 127.5, 125.3, 124.5, 120.3, 114.3, 111.6, 108.4, 91.3, 88.4, 26.6, 19.9. **HRMS** (ESI-TOF) m/z: $[M + Na]^+$ Calcd for $C_{33}H_{29}NNaO_3Si^+$ 538.1809; Found 538.1804.

(*E*)-3-((2-((*tert*-butyldiphenylsilyl)oxy)naphthalen-1-yl)ethynyl)isonicotinaldehyde oxime (1r)

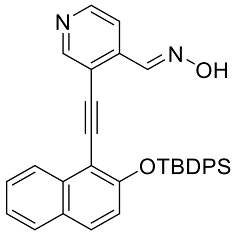

^{1}H NMR (600 MHz, Chloroform-*d*) δ 8.79 (s, 1H), 8.72 (s, 1H), 8.48 (d, *J* = 5.4 Hz, 1H), 8.20 (d, *J* = 9.0 Hz, 1H), 8.08 (s, 1H), 7.74 – 7.70 (m, 5H), 7.64 (d, *J* = 7.8 Hz, 1H), 7.53 – 7.49 (m, 1H), 7.41 (d, *J* = 9.6 Hz, 1H), 7.36 (t, *J* = 7.8 Hz, 2H), 7.32 (dd, *J* = 7.2, 1.2 Hz, 1H), 7.29 (t, *J* = 7.8 Hz, 4H), 6.71 (d, *J* = 9.0 Hz, 1H), 1.09 (s, 9H). **^{13}C NMR** (150 MHz, Chloroform-*d*) δ 156.2, 153.6, 148.3, 147.5, 140.3, 135.6, 134.7, 132.6, 130.6, 130.3, 128.7, 128.4, 128.1,

127.7, 125.2, 124.6, 120.4, 120.0, 118.7, 107.9, 93.7, 92.3, 26.6, 19.9. **HRMS** (ESI-TOF) m/z: $[M + Na]^+$ Calcd for $C_{34}H_{30}N_2NaO_2Si^+$ 527.2150; Found 527.2155.

(*E*)-3-((2-((*tert*-butyldiphenylsilyl)oxy)naphthalen-1-yl)ethynyl)-1-methyl-1*H*-indole-2-carbaldehyde oxime (1s)

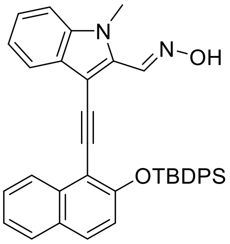

^{1}H NMR (600 MHz, Chloroform-*d*) δ 8.70 (s, 1H), 8.37 (d, *J* = 8.4 Hz, 1H), 7.86 (d, *J* = 7.8 Hz, 1H), 7.75 (dd, *J* = 8.4, 1.8 Hz, 4H), 7.61 (d, *J* = 7.8 Hz, 1H), 7.50 – 7.46 (m, 1H), 7.35 – 7.31 (m, 3H), 7.31 – 7.29 (m, 2H), 7.29 – 7.24 (m, 6H), 7.16 – 7.12 (m, 1H), 6.69 (d, *J* = 9.0 Hz, 1H), 3.99 (s, 3H), 1.09 (s, 9H). **^{13}C NMR** (150 MHz, Chloroform-*d*) δ 155.2, 144.0, 139.0, 135.7, 135.0, 133.2, 133.0, 130.1, 129.2, 128.8, 128.4, 128.2, 128.0, 127.2, 125.7, 124.9, 124.3, 121.2, 120.9, 120.4, 109.9, 109.5, 104.4, 90.9, 90.7, 33.1, 26.8, 19.9. **HRMS** (ESI-TOF) m/z: $[M + Na]^+$ Calcd for $C_{38}H_{34}N_2NaO_2Si^+$ 601.2282; Found 601.2281.

(*E*)-2-((2-((*tert*-butyldimethylsilyl)oxy)naphthalen-1-yl)ethynyl)benzaldehyde oxime (1t)

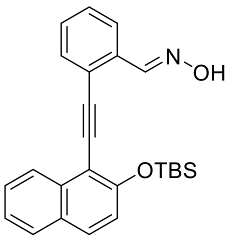

^{1}H NMR (600 MHz, Chloroform-*d*) δ 8.82 (s, 1H), 8.21 (d, *J* = 8.4 Hz, 1H), 7.83 (dd, *J* = 7.8, 1.2 Hz, 1H), 7.71 (d, *J* = 7.8 Hz, 1H), 7.67 (d, *J* = 9.0 Hz, 1H), 7.57 (dd, *J* = 7.8, 1.8 Hz, 1H), 7.51 – 7.47 (m, 1H), 7.34– 7.30 (m, 2H), 7.29 (td, *J* = 7.2, 1.2 Hz, 1H), 7.03 (d, *J* = 9.0 Hz, 1H), 0.98 (s, 9H), 0.23 (s, 6H). **^{13}C NMR** (150 MHz, Chloroform-*d*) δ 155.9, 149.5, 134.9, 133.1, 132.7, 130.3, 129.8, 129.0, 128.5, 128.3, 127.5, 125.5, 125.4, 124.6, 124.0, 121.1, 109.4, 95.0, 90.9, 25.9, 18.5. **HRMS** (ESI-TOF) m/z: $[M + Na]^+$ Calcd for $C_{25}H_{27}NNaO_2Si^+$ 424.1704; Found 424.1705.

(*E*)-2-((2-((triisopropylsilyl)oxy)naphthalen-1-yl)ethynyl)benzaldehyde oxime (1u)

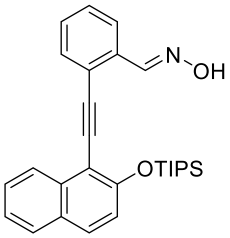

^{1}H NMR (600 MHz, Chloroform-*d*) δ 8.81 (s, 1H), 8.21 (d, *J* = 8.4 Hz, 1H), 7.84 (dd, *J* = 7.8, 1.2 Hz, 1H), 7.71 (d, *J* = 8.4 Hz, 1H), 7.67 – 7.63 (m, 2H), 7.56 (dd, *J* = 7.8, 1.2 Hz, 1H), 7.50 – 7.46 (m, 1H), 7.34 – 7.30 (m, 1H), 7.28 (td, *J* = 7.8, 1.2 Hz, 1H), 7.06 (d, *J* = 9.0 Hz, 1H), 1.32 (p, *J* = 7.8 Hz, 3H), 1.08 (d, *J* = 7.8 Hz, 18H). **^{13}C NMR** (150 MHz, Chloroform-*d*) δ 156.3, 149.5, 135.0, 133.1, 132.7, 130.3, 129.7, 128.8, 128.4, 128.2, 127.5, 125.5, 125.3, 124.4, 124.2, 120.5, 108.7, 95.0, 90.9, 18.2, 13.3. **HRMS** (ESI-TOF) m/z: $[M + H]^+$ Calcd for $C_{28}H_{34}NO_2Si^+$ 444.2354; Found 444.2353.

(*E*)-2-((2-((*tert*-butyldiphenylsilyl)oxy)-6-phenylnaphthalen-1-yl)ethynyl)benzaldehyde oxime (1v)

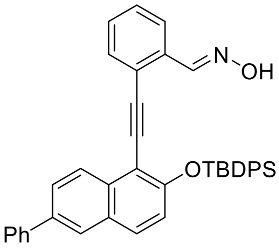

^{1}H NMR (600 MHz, Chloroform-*d*) δ 8.88 (s, 1H), 8.31 (d, *J* = 8.4 Hz, 1H), 7.88 – 7.85 (m, 1H), 7.81 (d, *J* = 2.4 Hz, 1H), 7.76 – 7.73 (m, 4H), 7.62 – 7.60 (m, 2H), 7.58 (dd, *J* = 6.6, 1.8 Hz, 1H), 7.42 (d, *J* = 9.0 Hz, 1H), 7.40 – 7.36(m, 3H), 7.36 – 7.32 (m, 3H), 7.31 – 7.26 (m, 7H), 6.71 (d, *J* = 9.0 Hz, 1H), 1.10 (s, 9H). **^{13}C NMR** (150 MHz, Chloroform-*d*) δ 155.8, 149.6, 141.0, 137.3, 135.6, 134.0, 133.2, 132.80, 132.77, 130.2, 129.8, 129.0, 128.6, 128.1, 127.4, 127.1, 126.1, 126.0, 125.5, 124.0, 120.8, 108.5, 95.5, 90.8, 26.7, 19.9. **HRMS** (ESI-TOF) m/z: [M + Na]$^+$ Calcd for $C_{41}H_{35}NNaO_2Si^+$ 624.2330; Found 624.2325.

(*E*)-2-((6-bromo-2-((*tert*-butyldiphenylsilyl)oxy)naphthalen-1-yl)ethynyl)benzaldehyde oxime (1w)

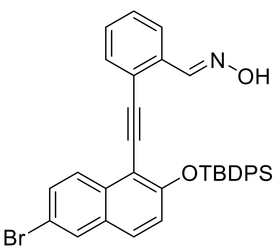

^{1}H NMR (600 MHz, Chloroform-*d*) δ 8.81 (s, 1H), 8.11 (d, *J* = 9.0 Hz, 1H), 7.86 – 7.83 (m, 1H), 7.76 (d, *J* = 1.8 Hz, 1H), 7.71 (dd, *J* = 7.8, 1.2 Hz, 4H), 7.63 (s, 1H), 7.55 (td, *J* = 7.8, 1.8 Hz, 2H), 7.38 – 7.31 (m, 3H), 7.32 – 7.25 (m, 6H), 6.69 (d, *J* = 9.0 Hz, 1H), 1.09 (s, 9H). **^{13}C NMR** (150 MHz, Chloroform-*d*) δ 155.9, 149.4, 135.6, 133.4, 133.2, 132.7, 132.6, 130.7, 130.3, 130.2, 129.85, 129.77, 128.9, 128.7, 128.1, 127.3, 125.5, 123.7, 121.4, 118.3, 108.9, 95.8, 90.2, 26.6, 19.9. **HRMS** (ESI-TOF) m/z: [M + Na]$^+$ Calcd for $C_{35}H_{30}BrNNaO_2Si^+$ 626.1122; Found 626.1118.

(*E*)-2-((2-((*tert*-butyldiphenylsilyl)oxy)-7-methoxynaphthalen-1-yl)ethynyl)benzaldehyde oxime (1x)

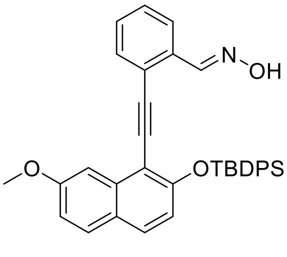

^{1}H NMR (600 MHz, Chloroform-*d*) δ 8.92 (s, 1H), 7.84 (dd, *J* = 7.8, 1.8 Hz, 1H), 7.77 – 7.72 (m, 5H), 7.61 (d, *J* = 2.4 Hz, 1H), 7.58 (dd, *J* = 7.8, 1.2 Hz, 1H), 7.50 (d, *J* = 9.0 Hz, 1H), 7.37 – 7.31 (m, 3H), 7.30 – 7.26 (m, 6H), 6.95 (dd, *J* = 9.0, 2.4 Hz, 1H), 6.51 (d, *J* = 9.0 Hz, 1H), 3.93 (s, 3H), 1.12 (s, 9H). **^{13}C NMR** (150 MHz, Chloroform-*d*) δ 159.3, 156.4, 149.3, 136.5, 135.6, 132.9, 132.8, 132.7, 130.2, 129.84, 129.79, 129.7, 128.4, 128.0, 125.6, 124.14, 124.07, 117.7, 117.1, 107.7, 104.0, 95.5, 91.3, 55.6, 26.7, 19.9. **HRMS** (ESI-TOF) m/z: [M + Na]$^+$ Calcd for $C_{36}H_{33}NNaO_3Si^+$ 578.2122; Found 578.2121.

(*E*)-2-((2-bromo-6-((*tert*-butyldiphenylsilyl)oxy)phenyl)ethynyl)benzaldehyde oxime (1aa)

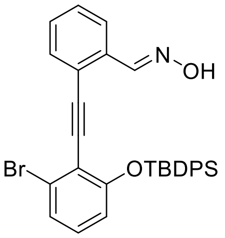

^{1}H NMR (600 MHz, Chloroform-*d*) δ 8.99 (s, 1H), 7.87 – 7.83 (m, 1H), 7.78 (dd, *J* = 8.4, 1.8 Hz, 4H), 7.54 – 7.50 (m, 1H), 7.39 – 7.35 (m, 3H), 7.33 – 7.27 (m, 6H), 7.08 (dd, *J* = 7.8, 1.2 Hz, 1H), 6.68 (t, *J* = 7.8 Hz, 1H), 6.33 (dd, *J* = 8.4, 1.2 Hz, 1H), 1.08 (s, 9H). **^{13}C NMR** (150 MHz, Chloroform-*d*) δ 157.5, 149.9, 135.6, 133.8, 132.5, 132.2, 130.3, 129.7, 128.8, 128.1, 127.0, 125.3, 125.2, 123.5, 117.9, 117.4, 94.7, 91.4, 26.6, 19.8. **HRMS** (ESI-TOF) m/z: $[M + H]^+$ Calcd for $C_{31}H_{29}BrNO_2Si^+$ 554.1146; Found 554.1136.

(*E*)-2-((2-((*tert*-butyldiphenylsilyl)oxy)-6-methylphenyl)ethynyl)benzaldehyde oxime (1ab)

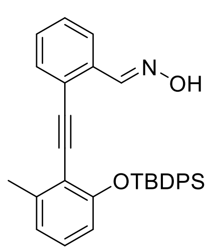

^{1}H NMR (600 MHz, Chloroform-*d*) δ 8.78 (s, 1H), 7.82 (dd, *J* = 7.2, 1.8 Hz, 1H), 7.72 – 7.67 (m, 5H), 7.50 (dd, *J* = 6.6, 1.8 Hz, 1H), 7.36 – 7.32 (m, 2H), 7.30 – 7.25 (m, 6H), 6.74 (t, *J* = 7.8 Hz, 1H), 6.70 (d, *J* = 7.8 Hz, 1H), 6.25 (d, *J* = 7.8 Hz, 1H), 2.47 (s, 3H), 1.06 (s, 9H). **^{13}C NMR** (150 MHz, Chloroform-*d*) δ 156.6, 149.6, 142.4, 135.6, 133.1, 132.8, 132.5, 130.1, 129.7, 128.9, 128.4, 128.0, 125.3, 124.2, 122.3, 116.5, 114.8, 94.4, 91.7, 26.7, 21.5, 19.8. **HRMS** (ESI-TOF) m/z: $[M + Na]^+$ Calcd for $C_{32}H_{31}NNaO_2Si^+$ 512.2017; Found 512.2010.

(*E*)-2-((2-((*tert*-butyldiphenylsilyl)oxy)-6-(methoxymethoxy)phenyl)ethynyl)benzaldehyde oxime (1ad)

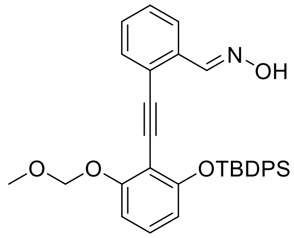

^{1}H NMR (600 MHz, Chloroform-*d*) δ 9.05 (s, 1H), 7.83 (dd, *J* = 7.8, 1.8 Hz, 1H), 7.74 – 7.69 (m, 4H), 7.47 (s, 1H), 7.45 – 7.42 (m, 1H), 7.38 – 7.34 (m, 2H), 7.32 – 7.28 (m, 4H), 7.28 – 7.22 (m, 2H), 6.77 (t, *J* = 8.4 Hz, 1H), 6.64 (dd, *J* = 8.4, 1.2 Hz, 1H), 6.07 (dd, *J* = 8.4, 0.6 Hz, 1H), 5.32 (s, 2H), 3.49 (s, 3H), 1.11 (s, 9H). **^{13}C NMR** (150 MHz, Chloroform-*d*) δ 159.6, 157.1, 150.3, 135.7, 133.5, 132.7, 131.5, 130.2, 129.7, 129.6, 128.2, 128.0, 125.0, 124.4, 112.8, 106.8, 105.6, 94.7, 94.5, 89.6, 56.5, 26.7, 19.9. **HRMS** (ESI-TOF) m/z: $[M + Na]^+$ Calcd for $C_{33}H_{33}NNaO_4Si^+$ 558.2072; Found 558.2073.

(*E*)-3-((*tert*-butyldiphenylsilyl)oxy)-2-((2-((hydroxyimino)methyl)phenyl)ethynyl)phenyl acetate (1ae)

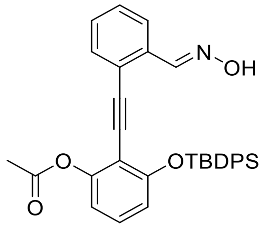

^{1}H NMR (600 MHz, Chloroform-*d*) δ 8.73 (s, 1H), 7.74 – 7.66 (m, 5H), 7.57 (s, 1H), 7.38 – 7.34 (m, 2H), 7.33 – 7.26 (m, 7H), 6.84 (t, *J* = 8.4 Hz, 1H), 6.62 (d, *J* = 9.6 Hz, 1H), 6.29 (d, *J* = 8.4 Hz, 1H), 2.32 (s, 3H), 1.07 (s, 9H). **^{13}C NMR** (150 MHz, Chloroform-*d*) δ 169.3, 157.4, 152.8, 149.2, 135.6, 133.2, 132.5, 132.3, 130.3, 129.7, 129.3, 128.7, 128.1, 125.3, 123.5, 116.8, 115.0, 109.6, 94.7, 87.7, 26.6, 21.1, 19.8. **HRMS** (ESI-TOF) m/z: [M + Na]$^+$ Calcd for $C_{33}H_{31}NNaO_4Si^+$ 556.1915; Found 556.1922.

4. General procedure for the synthesis of heteroaromatic *N*-oxides 3

To a 10 mL Schlenk tube under argon atmosphere, $Cu(CH_3CN)_4PF_6$ (1.9 mg, 5 mol%), **L12** (3.2 mg, 6 mol%) and dry DCM (0.4 ml) were added, and the mixture was stirred at RT for 30 minutes. Subsequently, **1** (0.1 mmol) in dry DCM (0.6 mL) was added. The reaction was stirred for 24 h (monitored by TLC) at RT. Then, the mixture was directly purified by column chromatography on silica gel (PE: EA=5:1 to 2:1) to afford the product **3**.

(*S*)-3-(2-((*tert*-butyldiphenylsilyl)oxy)naphthalen-1-yl)isoquinoline 2-oxide (3a)

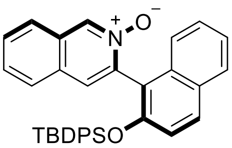

52.2 mg, 99% yield, pale-yellow solid, m.p: 116.4 – 117.5 °C; **HPLC** (Daicel Chiralpak AD-H, *n*-hexane/2-propanol = 70:30, 1.0 mL/min, at 254 nm): t_R = 5.35 min (major), t_R = 13.34 min (minor); er = 95.5:4.5, $[\alpha]D^{20}$ = +306.667 (*c* = 0.12, EA). **^{1}H NMR** (600 MHz, Chloroform-*d*) δ 8.95 (s, 1H), 7.74 – 7.71 (m, 2H), 7.69 (d, *J* = 7.8 Hz, 1H), 7.68 – 7.65 (m, 3H), 7.60 – 7.55 (m, 3H), 7.51 (t, *J* = 7.8 Hz, 2H), 7.35 – 7.27 (m, 4H), 7.26 – 7.22 (m, 5H), 6.77 (d, *J* = 9.0 Hz, 1H), 0.66 (s, 9H). **^{13}C NMR** (150 MHz, Chloroform-*d*) δ 151.8, 144.5, 136.8, 135.7, 135.4, 133.6, 132.8, 132.3, 130.7, 130.11, 130.08, 129.6, 129.1, 129.0, 128.8, 128.6, 128.3, 128.01, 127.97, 127.3, 127.2, 126.7, 124.5, 124.2, 124.0, 120.4, 118.2, 26.1, 19.2. **HRMS** (ESI-TOF) m/z: $[M + H]^+$ Calcd for $C_{35}H_{32}NO_2Si^+$ 526.2197; Found 526.2193.

(*S*)-3-(2-((*tert*-butyldiphenylsilyl)oxy)naphthalen-1-yl)-6-fluoroisoquinoline 2-oxide (3b)

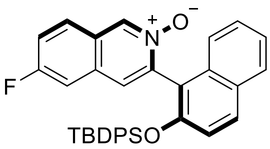

54 mg, 99% yield, pale-yellow solid, m.p: 107.8 – 109.7 °C; **HPLC** (Daicel Chiralpak IA, *n*-hexane/2-propanol = 70:30, 1.0 mL/min, at 254 nm): t_R = 5.34 min (major), t_R = 11.30 min (minor); er =96.5:3.5, $[\alpha]D^{20}$ = -87.333 (*c* = 0.15, EA). **^{1}H NMR** (600 MHz, DMSO-d_6) δ 8.71 (s, 1H), 7.91 – 7.86 (m, 2H), 7.70 – 7.65 (m, 3H), 7.62 (dd, *J* = 8.4 Hz, 1.2Hz, 2H), 7.54 – 7.50 (m, 2H), 7.41 (d, *J* = 8.4 Hz, 1H), 7.34 – 7.30 (m, 2H), 7.30 – 7.26 (m, 2H), 7.27-7.22 (m, 6H), 6.82 (d, *J* = 9.0 Hz, 1H), 3.82 (s, 3H), 0.68 (s,9H). **^{13}C NMR** (150 MHz, DMSO-d_6) δ 161.62 (d, J_{CF} = 246.0 Hz)，151.0, 142.69, 142.67, 135.23 (d, J_{CF} = 6.0 Hz)，135.1, 134.8, 133.1, 131.8, 131.5, 130.69 (d, J_{CF} = 10.5 Hz), 130.30, 130.27, 130.1, 129.98 (d, J_{CF} = 9.0 Hz),128.3, 128.1, 128.0, 127.9, 127.00 (d, J_{CF} = 24.0 Hz), 125.2, 124.0, 119.4, 118.3, 118.3, 118.2, 107.79 (d, J_{CF} = 22.5 Hz), 25.5, 18.5. **^{19}F NMR** (659 MHz, DMSO-d_6) δ -110.0. **HRMS** (ESI-TOF) m/z: $[M + Na]^+$ Calcd for $C_{35}H_{30}FNNaO_2Si^+$ 556.1923; Found 556.1930.

(*S*)-3-(2-((*tert*-butyldiphenylsilyl)oxy)naphthalen-1-yl)-6-chloroisoquinoline 2-oxide (3c)

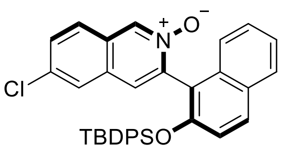

55.8 mg, 99% yield, pale-yellow solid, m.p: 118.5 – 119.9 °C; **HPLC** (Daicel Chiralpak AD-H, *n*-hexane/2-propanol = 70:30, 1.0 mL/min, at 254 nm): t_R = 5.25 min (major), t_R = 12.83 min (minor); er = 95:5, $[\alpha]D^{20}$ = +90.625 (*c* = 0.16, EA). **^{1}H NMR** (600 MHz, Chloroform-*d*) δ 8.91 (s, 1H), 7.68 – 7.65 (m, 5H), 7.63 (s, 1H), 7.58 (d, *J* = 1.2 Hz, 1H), 7.57 (d, *J* = 1.8 Hz, 1H), 7.53 – 7.49 (m, 2H), 7.35 – 7.30 (m, 2H), 7.30 – 7.28 (m, 2H), 7.27 – 7.23 (m, 5H), 6.77 (d, *J* = 9.0 Hz, 1H), 0.68 (s, 9H). **^{13}C NMR** (150 MHz, Chloroform-*d*) δ 151.8, 145.8, 136.6, 135.7, 135.4, 134.5, 133.4, 132.7, 132.1, 131.0, 130.3, 130.2, 128.4, 128.04, 128.01, 127.9, 127.3, 126.3, 126.0, 125.6, 124.1, 123.9, 120.4, 117.7, 26.1, 19.2. **HRMS** (ESI-TOF) m/z: $[M + Na]^+$ Calcd for $C_{35}H_{30}ClNNaO_2Si^+$ 582.1627; Found 582.1632.

(*S*)-3-(2-((*tert*-butyldiphenylsilyl)oxy)naphthalen-1-yl)-6-methylisoquinoline 2-oxide (3d)

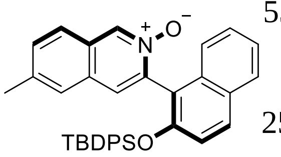

53.8 mg, 99% yield, pale-yellow solid, m.p: 120.6 – 122.1 °C; **HPLC** (Daicel Chiralpak AD-H, *n*-hexane/2-propanol = 70:30, 1.0 mL/min, at 254 nm): t_R = 4.96 min (major), t_R = 9.92 min (minor); er =96:4, $[\alpha]D^{20}$ = +460.286 (*c* = 0.14, EA). **^{1}H NMR** (600 MHz, Chloroform-*d*) δ 8.91 (s, 1H), 7.69 – 7.61 (m, 5H), 7.59 (d, *J* = 7.8 Hz, 2H), 7.51 – 7.46 (m, 2H), 7.41 (d, *J* = 8.4 Hz, 1H), 7.35 – 7.30 (m, 3H), 7.28 (t, *J* = 6.6 Hz, 1H), 7.26 – 7.21 (m, 5H), 6.76 (d, *J* = 9.0 Hz, 1H), 2.48 (s, 3H), 0.67 (s, 9H). **^{13}C NMR** (150 MHz, Chloroform-*d*) δ 151.8, 144.3, 139.1, 136.7, 135.7, 135.4, 133.6, 132.9, 132.3, 131.5, 130.7, 130.09, 130.06, 129.0, 128.3, 128.00, 127.96, 127.2, 126.7, 125.7, 124.5, 124.2, 124.0, 120.4, 118.3, 26.1, 22.1, 19.2. **HRMS** (ESI-TOF) m/z: $[M + Na]^+$ Calcd for $C_{36}H_{33}NNaO_2Si^+$ 562.2173; Found 562.2165.

(*S*)-3-(2-((*tert*-butyldiphenylsilyl)oxy)naphthalen-1-yl)-6-methoxyisoquinoline 2-oxide (3e)

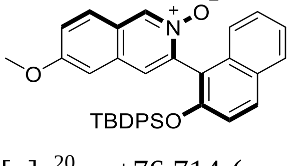

53.1 mg, 95% yield, pale-yellow solid, m.p: 193.6 – 194.5 °C; **HPLC** (Daicel Chiralpak AD-H, *n*-hexane/2-propanol = 70:30, 1.0 mL/min, at 254 nm): t_R = 6.00 min (major), t_R = 12.10 min (minor); er =96.5:3.5, $[\alpha]_D^{20}$ = +76.714 (*c* = 0.14, EA). **^{1}H NMR** (600 MHz, Chloroform-*d*) δ 8.87 (s, 1H), 7.69 – 7.64 (m, 3H), 7.63 (d, *J* = 9.0 Hz, 1H), 7.61 – 7.57 (m, 3H), 7.50 (d, *J* = 9.0 Hz, 1H), 7.35 – 7.30 (m, 3H), 7.28 (d, *J* = 7.8 Hz, 1H), 7.26 – 7.21(m 6H), 6.96 (d, *J* = 2.4 Hz, 1H), 6.77 (d, *J* = 9.0 Hz, 1H), 3.87 (s, 3H), 0.68 (s, 9H). **^{13}C NMR** (150 MHz, Chloroform-*d*) δ 160.0, 151.7, 144.5, 136.7, 135.7, 135.4, 133.6, 132.9, 132.3, 130.7, 130.1, 130.0, 129.0, 128.3, 128.01,

127.95, 127.2, 126.4, 126.2, 125.0, 124.3, 124.0, 122.2, 120.4, 118.4, 104.8, 55.7, 26.1, 19.2. **HRMS** (ESI-TOF) m/z: $[M + Na]^+$ Calcd for $C_{36}H_{33}NNaO_3Si^+$ 578.2122; Found 578.2122.

(*S*)-3-(2-((*tert*-butyldiphenylsilyl)oxy)naphthalen-1-yl)-6-(dimethylamino)isoquinoline 2-oxide (3f)

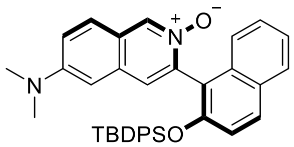

32 mg, 56% yield, pale-yellow solid, m.p: 134.3 – 136.4 °C; **HPLC** (Daicel Chiralpak AD-H, *n*-hexane/2-propanol = 70:30, 1.0 mL/min, at 254 nm): t_R = 6.03 min (major), t_R = 10.80 min (minor); er =97:3, $[\alpha]D^{20}$ = +136.000 (*c* = 0.13, EA). **^{1}H NMR** (600 MHz, Chloroform-*d*) δ 8.79 (s, 1H), 7.69 (dd, *J* =7.8, 1.2 Hz, 2H), 7.65 (d, *J* = 8.4 Hz, 1H), 7.62 – 7.58 (m, 3H), 7.50 – 7.45 (m, 2H), 7.38 (d, *J* = 8.4 Hz, 1H), 7.35 – 7.27 (m, 3H), 7.28 – 7.19 (m, 6H), 6.77 (d, *J* = 9.0 Hz, 1H), 6.65 (d, *J* = 2.4 Hz, 1H), 3.05 (s, 6H), 0.70 (s, 9H). **^{13}C NMR** (150 MHz, Chloroform-*d*) δ 151.6, 150.2, 143.8, 136.7, 135.8, 135.5, 133.7, 133.1, 132.4, 131.4, 130.4, 130.03, 139.99, 129.0, 128.2, 128.0, 127.9, 127.0, 126.0, 125.3, 124.5, 123.9, 122.1, 120.5, 118.9, 118.4, 103.8, 40.5, 26.1, 19.3. **HRMS** (ESI-TOF) m/z: $[M + Na]^+$ Calcd for $C_{37}H_{36}N_2NaO_2Si^+$ 591.2439; Found 491.2449.

(*S*)-3-(2-((*tert*-butyldiphenylsilyl)oxy)naphthalen-1-yl)-7-fluoroisoquinoline 2-oxide (3g)

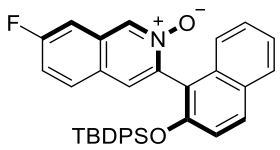

54.1 mg, 99% yield, pale-yellow solid, m.p: 207.8 – 209.7 °C; **HPLC** (Daicel Chiralpak AD-H, *n*-hexane/2-propanol = 70:30, 1.0 mL/min, at 254 nm): t_R = 6.26 min (major), t_R = 24.98 min (minor); er =95.5:4.5, $[\alpha]D^{20}$ = +137.067 (*c* = 0.14, EA). **^{1}H NMR** (600 MHz, DMSO-d_6) δ 9.20 (s, 1H), 8.26 (s, 1H), 8.09 (dd, *J* = 9.0, 6.0 Hz, 1H), 7.83 (d, *J* = 7.2 Hz, 1H), 7.80 (dd, *J* = 9.6, 2.4 Hz, 1H), 7.74 – 7.69 (m, 3H), 7.65 (dd, *J* = 7.8, 1.8 Hz, 2H), 7.57 (td, *J* =9.0, 3.0 Hz, 1H), 7.50 – 7.43 (m, 2H), 7.42 – 7.32 (m, 7H), 6.69 (d, *J* = 9.0 Hz, 1H), 0.66 (s, 9H). **^{13}C NMR** (150 MHz, DMSO-d_6) δ 161.62 (d, J_{CF} = 246.0 Hz),150.9, 142.69, 142.67, 135.23 (d, J_{CF} = 6.0 Hz), 134.8, 133.1, 131.8, 131.5, 130.69 (d, J_{CF} = 10.5 Hz), 130.29, 130.26, 130.1, 129.97 (d, J_{CF} = 10.5 Hz), 128.3, 128.1, 128.0, 127.9, 127.00 (d, J_{CF} = 24.0 Hz), 125.2, 124.0, 119.4, 118.3, 118.3, 118.1, 107.79 (d, *J*CF = 22.5 Hz), 25.5, 18.5. **^{19}F NMR** (659 MHz, DMSO-d_6) δ -109.9. **HRMS** (ESI-TOF) m/z: $[M + Na]^+$ Calcd for $C_{35}H_{30}FNNaO_2Si^+$ 556.1923; Found 556.1931.

(*S*)-3-(2-((*tert*-butyldiphenylsilyl)oxy)naphthalen-1-yl)-7-chloroisoquinoline 2-oxide (3h)

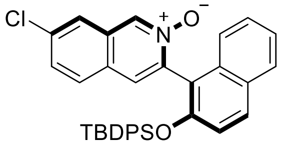

56 mg, 99% yield, pale-yellow solid, m.p: 198.9 – 201.9 °C; **HPLC** (Daicel Chiralpak AD-H, *n*-hexane/2-propanol = 70:30, 1.0 mL/min, at 254 nm): t_R = 6.69 min (major), t_R = 25.96 min (minor); er =95:5, $[\alpha]D^{20}$ = +117.077 (*c* = 0.13, EA). **^{1}H NMR** (600 MHz, Chloroform-*d*) δ 8.85 (s, 1H), 7.71 – 7.68 (m, 2H), 7.68 – 7.65 (m, 3H), 7.63 (d, *J* = 9 Hz, 1H), 7.60 – 7.57 (m, 2H), 7.51 (d, *J* = 9.0 Hz, 1H), 7.44 (dd, *J* =9, 1.8 Hz, 1H), 7.40 – 7.29 (m, 4H), 7.28-7.23 (m, 5H), 6.77 (d, *J* = 9.0 Hz, 1H), 0.67 (s, 9H). **^{13}C NMR** (150 MHz, Chloroform-*d*) δ 151.8, 145.0, 135.8, 135.7, 135.4, 135.2, 133.4, 132.7, 132.1, 130.9, 130.3, 130.15, 130.12, 129.4, 129.0, 128.4, 128.3, 128.27, 128.03, 127.3, 127.1, 126.8, 124.1, 124.0, 123.0, 120.4, 117.7, 26.0, 19.2. **HRMS** (ESI-TOF) m/z: $[M + Na]^+$ Calcd for $C_{35}H_{30}ClNNaO_2Si^+$ 582.1627; Found 582.1633.

(*S*)-3-(2-((*tert*-butyldiphenylsilyl)oxy)naphthalen-1-yl)-7-methylisoquinoline 2-oxide (3i)

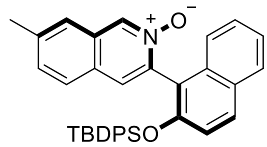

52 mg, 96% yield, pale-yellow solid, m.p: 122.8 – 124.9 °C; **HPLC** (Daicel Chiralpak AD-H, *n*-hexane/2-propanol = 70:30, 1.0 mL/min, at 254 nm): t_R = 5.61 min (major), t_R = 15.73 min (minor); er =95:5, $[\alpha]D^{20}$ = -97.455 (*c* = 0.11, EA). **^{1}H NMR** (600 MHz, Chloroform-*d*) δ 8.95 (s, 1H), 7.74 (td, *J* = 8.4, 1.8 Hz, 4H), 7.68 – 7.64 (m, 3H), 7.57 (d, *J* = 9.0 Hz, 2H), 7.44 – 7.37 (m, 4H), 7.36 (d, *J* = 7.8 Hz, 1H), 7.34 – 7.29 (m, 5H), 6.84 (d, *J* = 9.0 Hz, 1H), 2.58 (s, 3H), 0.73 (s, 9H). **^{13}C NMR** (150 MHz, Chloroform-*d*) δ 151.8, 143.5, 139.4, 136.4, 135.7, 135.4, 133.6, 132.9, 132.3, 131.0, 130.7, 130.09, 130.07, 129.9, 129.0, 128.3, 128.01, 127.97, 127.15, 127.07, 126.5, 124.3, 124.0, 123.4, 120.4, 118.3, 26.1, 22.0, 19.2. **HRMS** (ESI-TOF) m/z: $[M + Na]^+$ Calcd for $C_{36}H_{33}NNaO_2Si^+$ 562.2173; Found 562.2176.

(*S*)-3-(2-((*tert*-butyldiphenylsilyl)oxy)naphthalen-1-yl)-7-methoxyisoquinoline 2-oxide (3j)

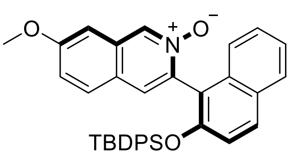

55.3 mg, 99% yield, pale-yellow solid, m.p: 133.5 – 135.9 °C; **HPLC** (Daicel Chiralpak AD-H, *n*-hexane/2-propanol = 70:30, 1.0 mL/min, at 254 nm): t_R = 5.19 min (major), t_R = 35.40 min (minor); er =95:5, $[\alpha]D^{20}$ = –37.500 (*c* = 0.12, EA). **^{1}H NMR** (600 MHz, Chloroform-*d*) δ 8.87 (s, 1H), 7.67 (dd, *J* = 8.4, 1.8 Hz, 2H), 7.65 (d, *J* = 9.0 Hz, 1H), 7.62 (s, 1H), 7.60 – 7.56 (m, 3H), 7.49 (d, *J* = 9.0 Hz, 1H), 7.35 – 7.30 (m, 3H), 7.30 – 7.27 (m, 1H), 7.27 – 7.22 (m, 5H), 7.15 (dd, *J* = 8.4, 2.4 Hz, 1H), 6.95 (d, *J* = 2.4 Hz, 1H), 6.76 (d, *J* = 9.0 Hz, 1H), 3.91 (s, 3H), 0.67 (s, 9H). **^{13}C NMR** (150 MHz, Chloroform-*d*) δ 160.0, 151.8, 142.0, 135.9, 135.7, 135.4, 133.7, 132.9, 132.3, 131.2, 130.6, 130.09, 130.06, 129.0, 128.30, 128.26, 128.00, 127.96, 127.1, 127.0, 124.5, 124.3,

124.0, 121.7, 120.4, 118.3, 102.1, 55.8, 26.1, 19.2. **HRMS** (ESI-TOF) m/z: [M + Na]$^+$ Calcd for $C_{36}H_{33}NNaO_3Si^+$ 578.2122; Found 578.2121.

(*S*)-3-(2-((*tert*-butyldiphenylsilyl)oxy)naphthalen-1-yl)-7-(dimethylamino)isoquinoline 2-oxide (3k)

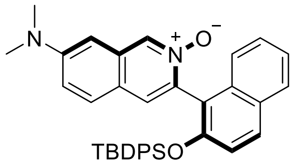

43 mg, 76% yield, pale-yellow solid, m.p: 122.1 – 123.2 °C; **HPLC** (Daicel Chiralpak AD-H, *n*-hexane/2-propanol = 70:30, 1.0 mL/min, at 254 nm): t_R = 5.42 min (major), t_R = 11.87 min (minor); er =97.5:2.5, [α]D^{20} = +61.267 (*c* = 0.3, EA). **^{1}H NMR** (600 MHz, Chloroform-*d*) δ 8.78 (s, 1H), 7.68 (dd, *J* = 8.4, 1.8 Hz, 2H), 7.64 (d, *J* = 7.8 Hz, 1H), 7.60 (dd, *J* = 7.8, 1.2 Hz, 2H), 7.55 – 7.51 (m, 2H), 7.47 (d, *J* = 9.0 Hz, 1H), 7.37 (d, *J* = 7.8 Hz, 1H), 7.34 – 7.29 (m, 2H), 7.28 – 7.21 (m, 6H), 7.13 (dd, *J* = 9.0, 2.4 Hz, 1H), 6.76 (d, *J* = 9.0 Hz, 1H), 6.66 (d, *J* = 2.4 Hz, 1H), 3.07 (s, 6H), 0.69 (s, 9H). **^{13}C NMR** (150 MHz, Chloroform-*d*) δ 151.8, 150.2, 139.7, 135.8, 135.5, 135.4, 133.9, 133.0, 132.4, 131.5, 130.3, 130.03, 130.00, 129.0, 128.2, 128.0, 127.9, 127.5, 127.0, 126.8, 124.5, 123.9, 122.0, 120.5, 118.8, 118.2, 101.4, 40.6, 26.1, 19.2. **HRMS** (ESI-TOF) m/z: [M + Na]$^+$ Calcd for $C_{37}H_{36}N_2NaO_2Si^+$ 591.2439; Found 591.2438.

(*S*)-3-(2-((*tert*-butyldiphenylsilyl)oxy)naphthalen-1-yl)-8-fluoroisoquinoline 2-oxide (3l)

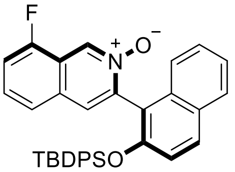

47.9 mg, 88% yield, pale-yellow solid, m.p: 114.3 – 115.8 °C; **HPLC** (Daicel Chiralpak IB, *n*-hexane/2-propanol = 90:10, 1.0 mL/min, at 254 nm): t_R = 9.81 min (major), t_R = 19.86 min (minor); er =99.5:0.5, [α]D^{20} = +61.267 (*c* = 0.3, EA). **^{1}H NMR** (600 MHz, Chloroform-*d*) δ 9.12 (s, 1H), 7.72 (s, 1H), 7.68 – 7.64 (m, 3H), 7.58 (dd, *J* = 7.8, 1.2 Hz, 2H), 7.51 (d, *J* = 9.0 Hz, 1H), 7.48 – 7.40 (m, 2H), 7.34 – 7.28 (m, 4H), 7.27 – 7.19 (m, 6H), 6.78 (d, *J* = 9.0 Hz, 1H), 0.68 (s, 9H). **^{13}C NMR** (150 MHz, Chloroform-*d*) δ 155.96 (d, J_{CF} = 253.5 Hz), 151.8, 145.8, 135.7, 135.4, 133.4, 132.7, 132.1, 131.2, 131.2, 130.9, 130.2, 130.1, 129.7, 129.7, 129.0, 128.59 (d, J_{CF} = 7.5 Hz), 128.4, 128.0, 128.0, 127.3, 127.10 (d, J_{CF} = 3.0 Hz), 124.1, 124.0, 122.48 (d, J_{CF} = 3.0 Hz), 120.51 (d, J_{CF} = 16.5 Hz), 120.4, 117.8, 112.87 (d, J_{CF} = 18.0Hz), 26.0, 19.2. **^{19}F NMR** (565 MHz, Chloroform-*d*) δ -121.8. **HRMS** (ESI-TOF) m/z: [M + Na]$^+$ Calcd for $C_{35}H_{30}FNNaO_2Si^+$ 566.1923; Found 566.1923.

(*S*)-3-(2-((*tert*-butyldiphenylsilyl)oxy)naphthalen-1-yl)-6,7-dimethoxyisoquinoline 2-oxide (3m)

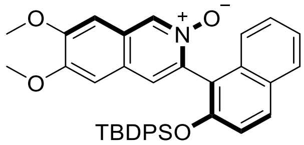

46 mg, 78% yield, pale-yellow solid, m.p: 171.1 – 173.5 °C; **HPLC** (Daicel Chiralpak AD-H, *n*-hexane/2-propanol = 70:30, 1.0 mL/min, at 254 nm): t_R = 6.34 min (major), t_R = 17.80 min (minor); er = 96:4, $[\alpha]D^{20}$ = +170.615 (*c* = 0.13, EA). **^{1}H NMR** (700 MHz, Chloroform-*d*) δ 8.83 (s, 1H), 7.68 (dd, *J* = 7.7, 1.4 Hz, 2H), 7.66 (d, *J* = 8.4 Hz, 1H), 7.59 (dd, *J* = 8.4, 1.4 Hz, 2H), 7.54 (s, 1H), 7.49 (d, *J* = 8.4 Hz, 1H), 7.35 – 7.30 (m, 3H), 7.30 – 7.27 (m, 1H), 7.27 – 7.22 (m, 5H), 6.95 (d, *J* = 20.3 Hz, 2H), 6.77 (d, *J* = 9.1 Hz, 1H), 4.00 (s, 3H), 3.94 (s, 3H), 0.69 (s, 9H). **^{13}C NMR** (175 MHz, Chloroform-*d*) δ 152.1, 151.9, 151.7, 142.3, 135.7, 135.6, 135.4, 133.6, 133.0, 132.3, 130.6, 130.1, 130.0, 129.0, 128.3, 128.0, 127.9, 127.1, 125.83, 125.78, 125.5, 124.4, 124.0, 120.4, 118.5, 105.1, 102.8, 56.4, 56.3, 26.1, 19.2. **HRMS** (ESI-TOF) m/z: $[M + Na]^+$ Calcd for $C_{37}H_{35}NNaO_4Si^+$ 608.2228; Found 608.2225.

(*S*)-7-(2-((*tert*-butyldiphenylsilyl)oxy)naphthalen-1-yl)-[1,3]dioxolo[4,5-g]isoquinoline 6-oxide (3n)

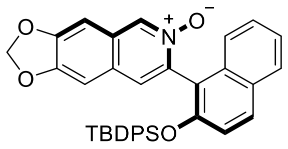

52 mg, 91% yield, pale-yellow solid, m.p: 180.2 – 182.9 °C; **HPLC** (Daicel Chiralpak AD-H, *n*-hexane/2-propanol = 70:30, 1.0 mL/min, at 254 nm): t_R = 6.68 min (major), t_R = 16.72 min (minor); er = 95.5:4.5, $[\alpha]D^{20}$ = +188.286 (*c* = 0.14, EA). **^{1}H NMR** (600 MHz, Chloroform-*d*) δ 8.78 (s, 1H), 7.70 – 7.64 (m, 3H), 7.60 (d, *J* = 6.6 Hz, 2H), 7.52 – 7.47 (m, 2H), 7.35 – 7.28 (m, 4H), 7.28 – 7.23 (m, 5H), 6.97 (d, *J* = 22.2 Hz, 2H), 6.76 (d, *J* = 9.0 Hz, 1H), 6.07 (d, *J* = 6.6 Hz, 2H), 0.69 (s, 9H). **^{13}C NMR** (150 MHz, Chloroform-*d*) δ 151.7, 150.2, 150.1, 142.6, 136.2, 135.7, 135.4, 133.6, 132.9, 130.6, 130.11, 130.08, 129.1, 128.3, 128.02, 127.98, 127.14, 127.12, 126.9, 126.3, 124.3, 124.0, 120.4, 118.3, 103.0, 102.1, 100.6, 26.1, 19.2. **HRMS** (ESI-TOF) m/z: $[M + Na]^+$ Calcd for $C_{36}H_{31}NNaO_4Si^+$ 592.1915; Found 592.1912.

(*S*)-2-(2-((*tert*-butyldiphenylsilyl)oxy)naphthalen-1-yl)benzo[f]isoquinoline 3-oxide (3o)

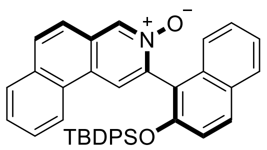

26 mg, 40% yield, pale-yellow solid, m.p: 235.7 – 237.2 °C; **HPLC** (Daicel Chiralpak AD-H, *n*-hexane/2-propanol = 70:30, 1.0 mL/min, at 254 nm): t_R = 6.37 min (major), t_R = 8.99 min (minor); er = 96.5:3.5, $[\alpha]D^{20}$ = +215.333 (*c* = 0.12, EA). **^{1}H NMR** (600 MHz, Chloroform-*d*) δ 8.97 (s, 1H), 8.49 (s, 1H), 8.29 (dd, *J* = 7.2, 2.4 Hz, 1H), 7.87 (dd, *J* = 6.6, 1.8 Hz, 1H), 7.83 (d, *J* = 9.0 Hz, 1H), 7.69 (d, *J* = 7.8 Hz, 1H), 7.66 (dd, *J* = 8.4, 1.8 Hz, 2H), 7.62 – 7.58 (m, 4H), 7.55 (d, *J* = 9.0 Hz, 1H), 7.35 (d, *J* = 7.8 Hz, 1H), 7.33 – 7.29 (m, 3H), 7.28 (dd, *J* = 6, 1.8 Hz, 1H), 7.26 – 7.20 (m, 5H), 6.83 (d, *J* = 9.0 Hz, 1H), 0.64 (s, 9H). **^{13}C NMR** (150 MHz, Chloroform-*d*) δ 151.9,

144.7, 137.5, 135.7, 135.4, 133.4, 132.9, 132.7, 132.3, 130.9, 130.8, 130.11, 130.08, 129.2, 129.1, 128.8, 128.5, 128.4, 128.3, 128.1, 128.02, 128.00, 127.3, 124.2, 124.1, 123.2, 123.0, 122.3, 120.5, 118.4, 26.1, 19.2. **HRMS** (ESI-TOF) m/z: $[M + Na]^+$ Calcd for $C_{39}H_{33}NNaO_2Si^+$ 598.2173; Found 598.2173.

(*S*)-5-(2-((*tert*-butyldiphenylsilyl)oxy)naphthalen-1-yl)thieno[2,3-c]pyridine 6-oxide (3p)

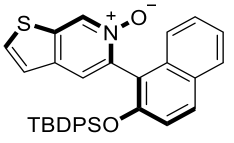

22.6 mg, 42% yield, pale-yellow solid, m.p: 130.1 – 132.5 °C; **HPLC** (Daicel Chiralpak AD-H, *n*-hexane/2-propanol = 70:30, 1.0 mL/min, at 254 nm): t_R = 5.67 min (major), t_R = 27.6 min (minor); er = 93:7, $[\alpha]D^{20}$ = +125.667 (*c* = 0.18, EA). **^{1}H NMR** (600 MHz, Chloroform-*d*) δ 8.94 (s, 1H), 7.68 – 7.64 (m, 4H), 7.60 – 7.56 (m, 3H), 7.50 (d, *J* = 9.0 Hz, 1H), 7.35 – 7.28 (m, 4H), 7.27 – 7.22 (m, 6H), 6.77 (d, *J* = 9.0 Hz, 1H), 0.70 (s, 9H). **^{13}C NMR** (150 MHz, Chloroform-*d*) δ 151.7, 143.6, 137.2, 136.6, 135.7, 135.4, 133.7, 133.4, 132.9, 132.2, 130.8, 130.7, 130.13, 130.09, 129.1, 128.4, 128.01, 127.97, 127.2, 124.2, 124.1, 123.0, 122.7, 120.4, 118.4, 26.1, 19.2. **HRMS** (ESI-TOF) m/z: $[M + H]^+$ Calcd for $C_{33}H_{30}NO_2SSi^+$ 532.1762; Found 532.1766.

(*S*)-5-(2-((*tert*-butyldiphenylsilyl)oxy)naphthalen-1-yl)furo[2,3-c]pyridine 6-oxide (3q)

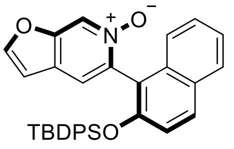

24.8 mg, 48% yield, pale-yellow solid, m.p: 160.3 – 162.8 °C; **HPLC** (Daicel Chiralpak AD-H, *n*-hexane/2-propanol = 70:30, 1.0 mL/min, at 254 nm): t_R = 5.57 min (major), t_R = 13.06 min (minor); er = 87:13, $[\alpha]D^{20}$ = +149.667 (*c* = 0.12, EA). **^{1}H NMR** (600 MHz, Chloroform-*d*) δ 8.79 (s, 1H), 7.74 (d, *J* = 1.8 Hz, 1H), 7.68 (dd, *J* = 7.8, 1.2 Hz, 2H), 7.65 (d, *J* = 7.8 Hz, 1H), 7.59 (dd, *J* = 7.8, 1.2 Hz, 2H), 7.50 – 7.47 (m, 2H), 7.35 – 7.30 (m, 2H), 7.30 – 7.23 (m, 7H), 6.76 – 6.73 (m, 2H), 0.71 (s, 9H). **^{13}C NMR** (150 MHz, Chloroform-*d*) δ 152.3, 151.6, 149.0, 143.0, 135.7, 135.4, 133.4, 132.9, 132.1, 130.6, 130.13, 130.10, 129.1, 128.3, 128.03, 127.98, 127.2, 125.9, 125.7, 124.1, 124.0, 120.4, 120.2, 118.5, 106.5, 26.0, 19.2. **HRMS** (ESI-TOF) m/z: $[M + Na]^+$ Calcd for $C_{33}H_{29}NNaO_3Si^+$ 538.1809; Found 538.1818.

(*S*)-3-(2-((*tert*-butyldiphenylsilyl)oxy)naphthalen-1-yl)-2,6-naphthyridine 2-oxide (3r)

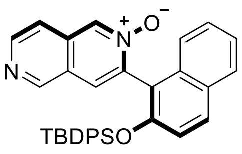

36 mg, 68% yield, pale-yellow solid, m.p: 107.9 – 109.8 °C; **HPLC** (Daicel Chiralpak AD-H, *n*-hexane/2-propanol = 70:30, 1.0 mL/min, at 254 nm): t_R = 6.52 min (major), t_R = 23.37 min (minor); er = 95:5, $[\alpha]D^{20}$ = +203.167 (*c* = 0.12, EA). **^{1}H NMR** (600 MHz, Chloroform-*d*) δ 9.10 (s, 1H), 8.86 (s, 1H), 8.60 (d, *J* = 5.4 Hz, 1H), 7.80 (s, 1H), 7.68 (dd, *J* = 8.4, 1.8 Hz, 1H), 7.56 (dd, *J* = 7.8, 1.2 Hz,

2H), 7.65 (dd, *J* = 7.8, 1.2 Hz, 2H), 7.54 (d, *J* = 9.0 Hz, 1H), 7.50 (d, *J* =6 Hz, 1H), 7.40 – 7.27 (m, 4H), 7.27 – 7.22 (m, 5H), 6.79 (d, *J* = 9.0 Hz, 1H), 0.67 (s, 9H). **^{13}C NMR** (150 MHz, Chloroform-*d*) δ 152.0, 150.9, 147.0, 146.2, 135.6, 135.43, 135.36, 135.3, 133.3, 132.5, 132.2, 132.0, 131.2, 130.2, 129.0, 128.5, 128.0, 127.4, 124.2, 123.7, 122.9, 120.4, 117.2, 116.2, 26.0, 19.2. **HRMS** (ESI-TOF) m/z: $[M + Na]^+$ Calcd for $C_{34}H_{30}N_2NaO_2Si^+$ 527.2150; Found 527.2151.

(*S*)-3-(2-((*tert*-butyldiphenylsilyl)oxy)naphthalen-1-yl)-9-methyl-9*H*-pyrido[3,4-b]indole 2-oxide (3s)

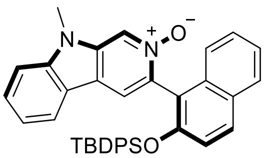

35.1 mg, 60% yield, yellow solid, m.p: 209.1 – 212.0 °C; **HPLC** (Daicel Chiralpak AD-H, *n*-hexane/2-propanol = 70:30, 1.0 mL/min, at 254 nm): t_R = 5.10 min (major), t_R = 6.34 min (minor); er = 90.5:9.5, $[\alpha]D^{20}$ = +169.222 (*c* = 0.18, EA). **^{1}H NMR** (600 MHz, Chloroform-*d*) δ 8.71 (s, 1H), 7.91 – 7.86 (m, 2H), 7.70 – 7.65 (m, 3H), 7.62 (dd, *J* = 8.4 Hz, 1.2Hz, 2H), 7.54 – 7.50 (m, 2H), 7.41 (d, *J* = 8.4 Hz, 1H), 7.34 – 7.30 (m, 2H), 7.30 – 7.26 (m, 2H), 7.27-7.22 (m, 6H), 6.82 (d, *J* = 9.0 Hz, 1H), 3.82 (s, 3H), 0.68 (s,9H). **^{13}C NMR** (150 MHz, Chloroform-*d*) δ 151.8, 143.1, 138.4, 138.3, 135.8, 135.5, 133.7, 133.0, 132.5, 130.5, 130.04, 130.01, 129.2, 128.3, 128.0, 127.98, 127.94, 127.0, 124.5, 124.0, 122.9, 121.3, 120.7, 120.6, 119.8, 109.4, 29.8, 26.1, 19.2. **HRMS** (ESI-TOF) m/z: $[M + Na]^+$ Calcd for $C_{38}H_{34}N_2NaO_2Si^+$ 601.2282; Found 601.2287.

(*S*)-3-(2-((*tert*-butyldimethylsilyl)oxy)naphthalen-1-yl)isoquinoline 2-oxide (3t)

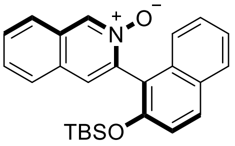

39 mg, 97% yield, pale-yellow solid, m.p: 150.3 – 153.1 °C; **HPLC** (Daicel Chiralpak AD-H, *n*-hexane/2-propanol = 70:30, 1.0 mL/min, at 254 nm): t_R = 8.15 min (major), t_R = 9.31 min (minor); er = 93:7, $[\alpha]D^{20}$ = +74.500 (*c* = 0.2, EA). **^{1}H NMR** (600 MHz, Chloroform-*d*) δ 8.92 (s, 1H), 7.81 (d, *J* = 9.0 Hz, 1H), 7.78 – 7.72(m, 2H), 7.70 (t, *J* = 7.2 Hz, 2H), 7.55 (t, *J* = 7.8 Hz, 1H), 7.51 (t, *J* = 7.8 Hz, 1H), 7.36 – 7.25 (m, 3H), 7.13 (d, *J* = 9.0 Hz, 1H), 0.62 (s, 9H), 0.09 (s, 3H), -0.03 (s, 3H). **^{13}C NMR** (150 MHz, Chloroform-*d*) δ 151.9, 144.2, 136.7, 133.5, 131.3, 129.5, 129.24, 129.16, 128.8, 128.7, 128.3, 127.7, 127.2, 126.6, 124.6, 124.5, 124.1, 120.9, 119.0, 25.5, 17.9 , 1.2, -4.1, -4.5. **HRMS** (ESI-TOF) m/z: $[M + Na]^+$ Calcd for $C_{25}H_{27}NNaO_2Si^+$ 424.1704; Found 424.1700.

(*S*)-3-(2-((triisopropylsilyl)oxy)naphthalen-1-yl)isoquinoline 2-oxide (3u)

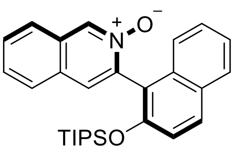

40.2 mg, 90% yield, pale-yellow solid, m.p: 140.2 – 143.3 °C; **HPLC** (Daicel Chiralpak OD-H, *n*-hexane/2-propanol = 90:10, 1.0 mL/min, at 254 nm): t_R = 7.53 min (major), t_R = 10.31 min (minor); er = 92:8, $[\alpha]D^{20}$ = +105.700 (*c* = 0.2, EA). **^{1}H NMR** (600 MHz, Chloroform-*d*) δ 8.92 (s, 1H), 7.79 (dd, *J* =9.0, 3.6 Hz, 1H), 7.76 – 7.72 (m, 2H), 7.70 (t, *J* = 7.8 Hz, 2H), 7.54 (t, *J* = 7.2 Hz, 1H), 7.50 (t, *J* = 7.2 Hz, 1H), 7.30 – 7.23 (m, 3H), 7.14 (dd, *J* = 9.0, 3.6 Hz, 1H), 1.14 – 1.07 (m, 3H), 0.88 – 0.82 (m, 18H). **^{13}C NMR** (150 MHz, Chloroform-*d*) δ 152.2, 144.5, 136.7, 133.7, 131.1, 129.5, 129.1, 128.91, 128.86, 128.6, 128.3, 127.4, 127.2, 126.5, 124.5, 124.3, 123.9, 120.2, 118.0, 17.95, 17.91, 13.0. **HRMS** (ESI-TOF) m/z: $[M + H]^+$ Calcd for $C_{28}H_{34}NO_2Si^+$ 444.2354; Found 444.2351.

(*S*)-3-(2-((*tert*-butyldiphenylsilyl)oxy)-6-phenylnaphthalen-1-yl)isoquinoline 2-oxide (3v)

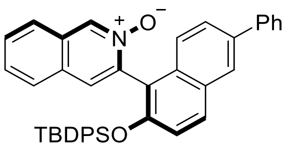

31.4 mg, 52% yield, pale-yellow solid, m.p: 183.4 – 185.1 °C; **HPLC** (Daicel Chiralpak AD-H, *n*-hexane/2-propanol = 70:30, 1.0 mL/min, at 254 nm): t_R = 7.62 min (major), t_R = 14.09 min (minor); er = 90:10, $[\alpha]D^{20}$ = +302.00 (*c* = 0.12, EA). **^{1}H NMR** (600 MHz, Chloroform-*d*) δ 8.97 (s, 1H), 7.86 (d, *J* = 1.8 Hz, 1H), 7.75 (s, 1H), 7.73 (d, *J* = 8.4 Hz, 1H), 7.71 (d, *J* = 9.0 Hz, 1H), 7.68 – 7.66 (m, 2H), 7.61 – 7.59 (m, 2H), 7.58 – 7.54 (m, 5H), 7.54 – 7.50 (m, 1H), 7.41 (d, *J* = 8.4 Hz, 1H), 7.38 – 7.32 (m, 3H), 7.32 – 7.30 (m, 1H), 7.27 – 7.22 (m, 5H), 6.80 (d, *J* = 9.0 Hz, 1H), 0.67 (s, 9H). **^{13}C NMR** (150 MHz, Chloroform-*d*) δ 151.9, 144.3, 141.2, 136.9, 136.8, 135.7, 135.4, 132.82, 132.76, 132.2, 131.0, 130.13, 130.11, 129.6, 129.3, 129.2, 128.9, 128.8, 128.6, 128.1, 128.05, 128.03, 127.4, 127.3, 126.9, 126.7, 126.3, 124.8, 124.6, 120.9, 118.1, 26.1, 19.2. **HRMS** (ESI-TOF) m/z: $[M + Na]^+$ Calcd for $C_{41}H_{35}NNaO_2Si^+$ 624.2330; Found 624.2336.

(*S*)-3-(6-bromo-2-((*tert*-butyldiphenylsilyl)oxy)naphthalen-1-yl)isoquinoline 2-oxide (3w)

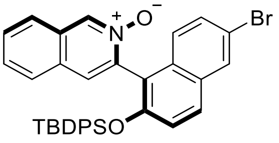

46 mg, 76% yield, pale-yellow solid, m.p: 169.3 – 171.8 °C; **HPLC** (Daicel Chiralpak IA, *n*-hexane/2-propanol = 70:30, 1.0 mL/min, at 254 nm): t_R = 7.21 min (major), t_R = 21.49 min (minor); er =95:5, $[\alpha]D^{20}$ = +200.133 (*c* = 0.15, EA). **^{1}H NMR** (600 MHz, Chloroform-*d*) δ 8.94 (s, 1H), 7.82 (d, *J* = 1.8 Hz, 1H), 7.73 (d, *J* = 8.4 Hz, 1H), 7.70 (t, *J* = 4.2 Hz, 2H), 7.64 (dd, *J* = 8.4, 1.8 Hz, 2H) , 7.60 – 7.56 (m, 3H), 7.54 (td, *J* = 8.4, 1.2 Hz, 1H), 7.41 (d, *J* = 9.0 Hz, 1H), 7.36 – 7.30(m, 3H), 7.24 (t, *J* = 7.8 Hz, 4H), 7.20 (d, *J* = 9.0 Hz, 1H), 6.79 (d, *J* = 9.0 Hz, 1H), 0.66 (s, 9H). **^{13}C NMR** (150 MHz, Chloroform-*d*) δ 152.1, 143.8, 136.8, 135.7, 135.4, 132.6, 132.1, 132.0, 130.4, 130.21, 130.19, 130.1, 129.8, 129.7, 129.3, 128.8, 128.1, 128.0, 127.4, 126.7, 126.1, 124.6,

121.5, 118.5, 117.8, 26.0, 19.2. **HRMS** (ESI-TOF) m/z: $[M + Na]^+$ Calcd for $C_{35}H_{30}BrNNaO_2Si^+$ 626.1122; Found 628.1113.

(*S*)-3-(2-((*tert*-butyldiphenylsilyl)oxy)-7-methoxynaphthalen-1-yl)isoquinoline 2-oxide (3x)

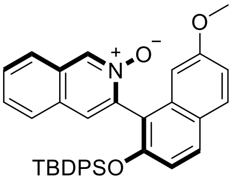

48 mg, 84% yield, pale-yellow solid, m.p: 238.0 – 241.9 °C; **HPLC** (Daicel Chiralpak AD-H, *n*-hexane/2-propanol = 70:30, 1.0 mL/min, at 254 nm): t_R = 5.96 min (major), t_R = 21.26 min (minor); er =68:32, $[\alpha]D^{20}$ = +77.920 (*c* = 0.25, EA). **^{1}H NMR** (600 MHz, Chloroform-*d*) δ 8.96 (s, 1H), 7.74 – 7.69 (m, 3H), 7.66 (dd, *J* = 7.8, 1.2 Hz, 2H), 7.59 – 7.55 (m, 4H), 7.52 (t, *J* = 8.4 Hz, 1H), 7.42 (d, *J* = 8.4 Hz, 1H), 7.34 – 7.29 (m, 2H), 7.26 – 7.21 (m, 4H), 6.91 (dd, *J* = 9.0, 2.4 Hz, 1H), 6.63 – 6.59 (m, 2H), 3.62 (s, 3H), 0.65 (s, 9H). **^{13}C NMR** (150 MHz, Chloroform-*d*) δ 158.8, 152.5, 144.8, 136.8, 135.7, 135.4, 135.0, 132.9, 132.3, 130.4, 130.1, 130.06, 130.05, 129.6, 129.1, 128.9, 128.6, 128.0, 127.9, 127.2, 126.7, 124.6, 124.6, 117.9, 117.4, 116.3, 103.1, 55.4, 26.1, 19.2. **HRMS** (ESI-TOF) m/z: $[M + Na]^+$ Calcd for $C_{36}H_{33}NNaO_3Si^+$ 578.2122; Found 578.2116.

(*S*)-3-(2-((*tert*-butyldiphenylsilyl)oxy)-5,6,7,8-tetrahydronaphthalen-1-yl)isoquinoline 2-oxide (3y)

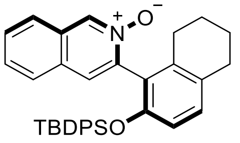

24.1 mg, 45% yield, pale-yellow solid, m.p: 115.3 – 117.5 °C; **HPLC** (Daicel Chiralpak AD-H, *n*-hexane/2-propanol = 70:30, 1.0 mL/min, at 254 nm): t_R = 4.23 min (major), t_R = 7.54 min (minor); er = 97.5:2.5, $[\alpha]D^{20}$ = +199.167 (*c* = 0.12, EA). **^{1}H NMR** (600 MHz, Chloroform-*d*) δ 8.90 (s, 1H), 7.67 (d, *J* = 8.4 Hz, 2H), 7.62 – 7.58 (m, 3H), 7.55 (dd, *J* = 7.8, 1.8 Hz, 2H), 7.52(dd, *J* = 8.4, 1.8 Hz, 1H), 7.49 (td, *J* = 8.4, 1.8 Hz, 1H), 7.33 – 7.28 (m, 2H), 7.23 (td, *J* = 7.8, 3.6Hz, 4H), 6.70 (d, *J* = 8.4 Hz, 1H), 6.26 (d, *J* = 8.4 Hz, 1H), 2.68 – 2.52 (m, 3H), 2.42 – 2.34 (m, 1H), 1.76 – 1.60 (m, 4H), 0.59 (s, 9H). **^{13}C NMR** (150 MHz, Chloroform-*d*) δ 151.2, 145.6, 137.8, 136.6, 135.7, 135.4, 133.1, 132.4, 130.8, 129.91, 129.90, 129.8, 129.4, 129.0, 128.9, 128.5, 127.9, 127.8, 126.5, 126.2, 124.5, 123.8, 116.4, 29.2, 27.2, 26.1, 23.0, 22.9, 19.2. **HRMS** (ESI-TOF) m/z: $[M + Na]^+$ Calcd for $C_{35}H_{35}NNaO_2Si^+$ 552.2330; Found 552.2332.

(*R*)-3-(2-((*tert*-butyldiphenylsilyl)oxy)-6-chlorophenyl)isoquinoline 2-oxide (3z)

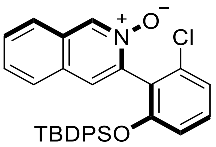

50 mg, 98% yield, pale-yellow solid, m.p: 122.1 – 124.8 °C; **HPLC** (Daicel Chiralpak AD-H, *n*-hexane/2-propanol = 70:30, 1.0 mL/min, at 254 nm): t_R = 4.55 min (major), t_R = 5.35 min (minor); er =98.5:1.5, $[\alpha]D^{20}$ = +207.167 (*c* = 0.12, EA). **^{1}H NMR** (700 MHz, Chloroform-*d*) δ 8.87 (s, 1H), 7.71 (d, *J* = 8.4 Hz, 1H), 7.68 (d, *J* = 8.4 Hz, 1H), 7.66 (s, 1H), 7.62 (dd, *J* = 7.7, 1.4 Hz, 2H), 7.56 – 7.53 (m, 3H), 7.52 – 7.49 (m, 1H), 7.36 – 7.30 (m, 2H), 7.25 (td, *J* = 7.7, 2.8 Hz, 4H), 6.99 (dd, *J* = 8.4, 1.4 Hz, 1H), 6.90 (t, *J* = 8.4 Hz, 1H), 6.39 (dd, *J* = 8.4, 1.4 Hz, 1H), 0.61 (s, 9H). **^{13}C NMR** (175 MHz, Chloroform-*d*) δ 155.0, 143.5, 136.4, 135.7, 135.4, 135.1, 132.3, 131.5, 130.6, 130.2, 129.6, 129.3, 128.6, 128.0, 126.8, 126.6, 124.6, 124.4, 122.1, 117.6, 25.9, 19.1. **HRMS** (ESI-TOF) m/z: $[M + Na]^+$ Calcd for $C_{31}H_{28}ClNNaO_2Si^+$ 532.1471; Found 532.1472.

(*R*)-3-(2-bromo-6-((*tert*-butyldiphenylsilyl)oxy)phenyl)isoquinoline 2-oxide (3aa)

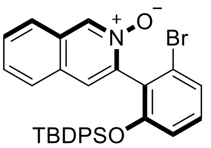

51 mg, 92% yield, pale-yellow solid, m.p: 136.1 – 139.0 °C; **HPLC** (Daicel Chiralpak AD-H, *n*-hexane/2-propanol = 70:30, 1.0 mL/min, at 254 nm): t_R = 4.59 min (major), t_R = 5.56 min (minor); er =99:1, $[\alpha]D^{20}$ = +209.538 (*c* = 0.13, EA). **^{1}H NMR** (700 MHz, Chloroform-*d*) δ 8.87 (s, 1H), 7.72 (d, *J* = 8.4 Hz, 1H), 7.69 (d, *J* = 7.7 Hz, 1H), 7.65 (s, 1H), 7.62 (dd, *J* = 6.3, 1.4 Hz, 2H), 7.57 – 7.53 (m, 3H), 7.51 (td, *J* = 7.0, 1.4 Hz, 1H), 7.36 – 7.30 (m, 2H), 7.25 (td, *J* = 7.7, 2.1 Hz, 4H), 7.16 (dt, *J* = 8.4, 1.4 Hz, 1H), 6.84 (td, *J* = 8.4, 1.4 Hz, 1H), 6.42 (dt, *J* = 7.2, 0.7 Hz, 1H), 0.60 (s, 9H). **^{13}C NMR** (175 MHz, Chloroform-*d*) δ 154.9, 145.0, 136.4, 135.7, 135.4, 132.2, 131.5, 131.0, 130.20, 130.18, 129.6, 129.3, 128.7, 128.6, 128.03, 128.01, 126.8, 126.4, 126.4, 125.2, 124.8, 124.6, 118.2, 25.9, 19.1. **HRMS** (ESI-TOF) m/z: $[M + H]^+$ Calcd for $C_{31}H_{29}BrNO_2Si^+$ 554.1146; Found 554.1138.

(*S*)-3-(2-((*tert*-butyldiphenylsilyl)oxy)-6-methylphenyl)isoquinoline 2-oxide (3ab)

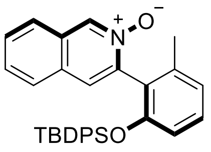

48 mg, 98% yield, pale-yellow solid, m.p: 212.9 – 215.3 °C; **HPLC** (Daicel Chiralpak AD-H, *n*-hexane/2-propanol = 70:30, 1.0 mL/min, at 254 nm): t_R = 4.01 min (major), t_R = 5.10 min (minor); er =98:2, $[\alpha]D^{20}$ = +91.625 (*c* = 0.16, EA). **^{1}H NMR** (600 MHz, Chloroform-*d*) δ 8.89 (s, 1H), 7.79 – 7.67 (m, 2H), 7.63 (s, 1H), 7.60 (dd, *J* = 8.4, 1.2 Hz, 2H), 7.57 – 7.52 (m, 3H), 7.50 (t, *J* = 7.8 Hz, 1H), 7.34 – 7.28 (m, 2H), 7.24 (td, *J* = 7.8, 2.4, 4H), 6.88 (t, *J* = 7.8 Hz, 1H), 6.80 (d, *J* = 7.8 Hz, 1H), 6.33 (d, *J* = 8.4 Hz, 1H), 2.13 (s, 3H), 0.60 (s, 9H). **^{13}C NMR** (150 MHz, Chloroform-*d*) δ 153.6, 145.4, 139.3, 136.5, 135.7, 135.4, 132.9, 132.1, 130.0, 129.8, 129.4, 129.0, 128.9, 128.6, 127.91,

127.89, 126.6, 126.2, 124.5, 122.7, 116.6, 26.0, 19.9, 19.2. **HRMS** (ESI-TOF) m/z: [M + Na]$^+$ Calcd for $C_{32}H_{31}NNaO_2Si^+$ 512.2017; Found 512.2016.

(*S*)-3-(2-((*tert*-butyldiphenylsilyl)oxy)-6-methoxyphenyl)isoquinoline 2-oxide (3ac)

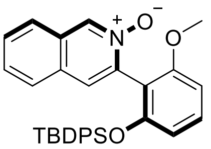

48 mg, 95% yield, pale-yellow solid, m.p: 170.3 – 173.1 °C; **HPLC** (Daicel Chiralpak AD-H, *n*-hexane/2-propanol = 70:30, 1.0 mL/min, at 254 nm): t_R = 5.39 min (major), t_R = 6.56 min (minor); er =3:97, $[\alpha]D^{20}$ = +238.500 (*c* = 0.12, EA). **^{1}H NMR** (600 MHz, Chloroform-*d*) δ 8.88 (s, 1H), 7.69 – 7.65 (m, 2H), 7.65 – 7.61 (m, 3H), 7.56 (dd, *J* = 7.8, 1.2 Hz, 2H), 7.51 (t, *J* = 7.2 Hz, 1H), 7.48 (t, *J* = 7.2 Hz, 1H), 7.34 – 7.29 (m, 2H), 7.24 (t, *J* = 7.2 Hz, 4H), 6.94 (t, *J* = 7.8 Hz, 1H), 6.51 (d, *J* = 8.4 Hz, 1H), 6.13 (d, *J* = 8.4 Hz, 1H), 3.69 (s, 3H), 0.62 (s, 9H). **^{13}C NMR** (150 MHz, Chloroform-*d*) δ 158.9, 154.7, 143.1, 136.5, 135.7, 135.44, 135.39, 132.9, 132.1, 130.5, 130.0, 129.4, 128.9, 128.8, 128.4, 127.90, 127.88, 126.9, 126.6, 124.6, 113.7, 112.2, 104.0, 56.1, 26.1, 19.2. **HRMS** (ESI-TOF) m/z: [M + Na]$^+$ Calcd for $C_{33}H_{31}NNaO_3Si^+$ 528.1966; Found 528.1969.

(*S*)-3-(2-((*tert*-butyldiphenylsilyl)oxy)-6-(methoxymethoxy)phenyl)isoquinoline 2-oxide (3ad)

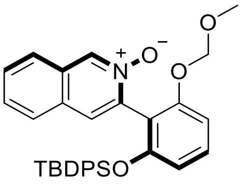

53.2 mg, 99% yield, pale-yellow solid, m.p: 232.3 – 233.8 °C; **HPLC** (Daicel Chiralpak IA, *n*-hexane/2-propanol = 90:10, 1.0 mL/min, at 254 nm): t_R = 16.06 min (major), t_R = 17.54 min (minor); er = 3:97, $[\alpha]D^{20}$ = +91.857 (*c* = 0.14, EA). **^{1}H NMR** (600 MHz, Chloroform-*d*) δ 8.84 (s, 1H), 7.68 – 7.63 (m, 3H), 7.63 – 7.60 (m, 2H), 7.57 – 7.54 (m, 2H), 7.53 – 7.49 (m, 1H), 7.48 – 7.45 (m, 1H), 7.34 – 7.28 (m, 2H), 7.24 (t, *J* = 7.8 Hz, 4H), 6.91 (t, *J* = 8.4 Hz, 1H), 6.72 (dd, *J* = 8.4, 0.6 Hz, 1H), 6.18 (dd, *J* = 8.4, 0.6 Hz, 1H), 5.14 (d, *J* = 6.6 Hz, 1H), 4.94 (d, *J* = 6.6 Hz, 1H), 3.29 (s, 3H), 0.63 (s, 9H). **^{13}C NMR** (150 MHz, Chloroform-*d*) δ 156.7, 154.6, 143.1, 136.2, 135.7, 135.4, 132.8, 132.1, 130.5, 130.0, 129.5, 128.8, 128.7, 128.3, 127.89, 127.88, 126.7, 126.6, 124.4, 113.2, 107.7, 95.0, 56.3, 26.1, 19.2. **HRMS** (ESI-TOF) m/z: [M + Na]$^+$ Calcd for $C_{33}H_{33}NNaO_4Si^+$ 558.2072; Found 558.2080.

(*S*)-3-(2-acetoxy-6-((*tert*-butyldiphenylsilyl)oxy)phenyl)isoquinoline 2-oxide (3ae)

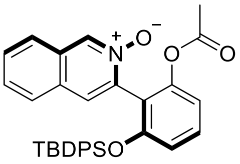

32.3 mg, 60% yield, pale-yellow solid, m.p: 115.3 – 118.6 °C; **HPLC** (Daicel Chiralpak AD-H, *n*-hexane/2-propanol = 70:30, 1.0 mL/min, at 254 nm): t_R = 5.45 min (major), t_R = 11.33 min (minor); er =90.5:9.5, $[\alpha]D^{20}$ = −388.769 (*c* = 0.13, EA). **^{1}H NMR** (600 MHz, Chloroform-*d*) δ 8.88 (s, 1H), 7.69 (dd,

J =7.8, 3.0 Hz, 2H), 7.65 – 7.60 (m, 3H), 7.58 – 7.54 (m, 3H), 7.51 (t, *J* = 8.4 Hz, 1H), 7.32 (dd, *J* = 16.8, 7.2 Hz, 2H), 7.25 (q, *J* = 7.8 Hz, 4H), 7.00 (t, *J* = 8.4 Hz, 1H), 6.72 (dd, *J* = 8.4, 1.2 Hz, 1H), 6.38 (dd, *J* = 8.4, 0.6 Hz, 1H), 1.94 (s, 3H), 0.62 (s, 9H). **^{13}C NMR** (150 MHz, Chloroform-*d*) δ 169.7, 154.8, 150.1, 141.8, 136.5, 135.7, 135.4, 132.3, 131.7, 130.4, 130.2, 130.1, 129.5, 129.3, 128.8, 128.6, 128.01, 127.98, 126.8, 126.5, 124.6, 118.4, 116.9, 115.3, 26.0, 20.9, 19.2. **HRMS** (ESI-TOF) m/z: [M + Na]$^+$ Calcd for $C_{33}H_{31}NNaO_4Si^+$ 556.1915; Found 556.1923.

(*S*)-3-(2-methoxynaphthalen-1-yl)isoquinoline 2-oxide (3af)

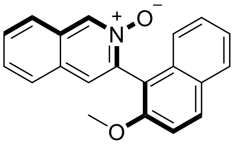

27.4 mg, 91% yield, pale-yellow solid, m.p: 153.4 – 155.2 °C; **HPLC** (Daicel Chiralpak AD-H, *n*-hexane/2-propanol = 70:30, 1.0 mL/min, at 254 nm): t_R = 11.58 min (minor), t_R = 21.57 min (major); er = 64:36, [α]D^{20} = -114.300 (c = 0.13, EA). **^{1}H NMR** (600 MHz, Chloroform-*d*) δ 8.95 (s, 1H), 7.93 (d, *J* = 9.0 Hz, 1H), 7.80 – 7.75 (m, 1H), 7.74 – 7.70 (m, 3H), 7.58 – 7.54 (m, 1H), 7.53 – 7.50 (m, 1H), 7.33 (d, *J* = 9.0 Hz, 1H), 7.31 – 7.27 (m, 1H), 7.27 – 7.27 (m, 1H), 3.82 (s, 3H). **^{13}C NMR** (150 MHz, Chloroform-*d*) δ 155.6, 143.9, 137.1, 133.3, 131.6, 129.6, 129.2, 129.1, 129.0, 128.8, 128.4, 127.4, 127.2, 126.8, 124.8, 124.00, 123.96, 116.1, 113.5, 56.8. **HRMS** (ESI-TOF) m/z: [M + Na]$^+$ Calcd for $C_{20}H_{15}NNaO_2^+$ 324.0995; Found 324.1000.

(*S*)-3-(2-bromonaphthalen-1-yl)isoquinoline 2-oxide (3ag)

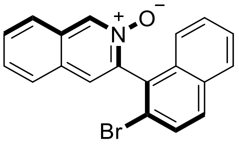

36.3mg, 99% yield, pale-yellow solid, m.p: 123.4 – 127.5 °C; **HPLC** (Daicel Chiralpak AD-H, *n*-hexane/2-propanol = 70:30, 1.0 mL/min, at 254 nm): t_R = 14.65 min (minor), t_R = 18.69 min (major); er = 84.5:15.5, [α]D^{20} = -179.000 (c = 0.12, EA). **^{1}H NMR** (600 MHz, Chloroform-*d*) δ 8.95 (s, 1H), 7.82 (d, *J* = 8.4 Hz, 1H), 7.79 (d, *J* = 9.0 Hz, 1H), 7.76 (dd, *J* = 8.4, 0.6 Hz, 2H), 7.73 – 7.68 (m, 2H), 7.60 (td, *J* = 6.6, 0.6 Hz, 1H), 7.56 (td, *J* = 7.8, 1.2 Hz, 1H), 7.43 (td, *J* = 7.2, 1.2 Hz, 1H), 7.34 (td, *J* = 8.4, 1.2 Hz, 1H), 7.29 (dd, *J* = 8.4, 1.2 Hz, 1H). **^{13}C NMR** (150 MHz, Chloroform-*d*) δ 145.7, 137.1, 133.6, 132.4, 131.5, 131.1, 129.9, 129.8, 129.7, 129.1, 128.9, 128.5, 127.9, 127.0, 126.9, 126.6, 125.2, 124.8, 123.2. **HRMS** (ESI-TOF) m/z: [M + H]$^+$ Calcd for $C_{19}H_{13}BrNO^+$ 350.0176; Found 350.0179.

5. Scale-up reaction and transformations of the product

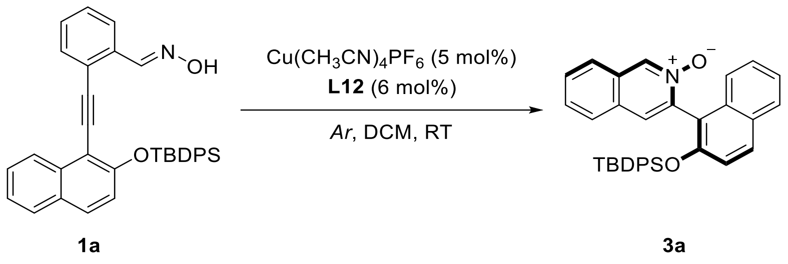

To a 25 mL Schlenk tube under argon atmosphere, $Cu(CH_3CN)_4PF_6$ (18.7 mg, 5 mol%), **L12** (31.9 mg, 6 mol%) and dry DCM (4.0 ml) were added, and the mixture was stirred at RT for 30 minutes. Subsequently, **1a** (525.8 mg, 1.0 mmol) in dry DCM (6.0 mL) were added. The reaction was stirred for 24 h (monitored by TLC). Then, the mixture was directly purified by column chromatography on silica gel (PE: EA=2:1) to afford the product **3a** (474.0 mg, 90% yield, 92% *ee*) as a pale-yellow solid.

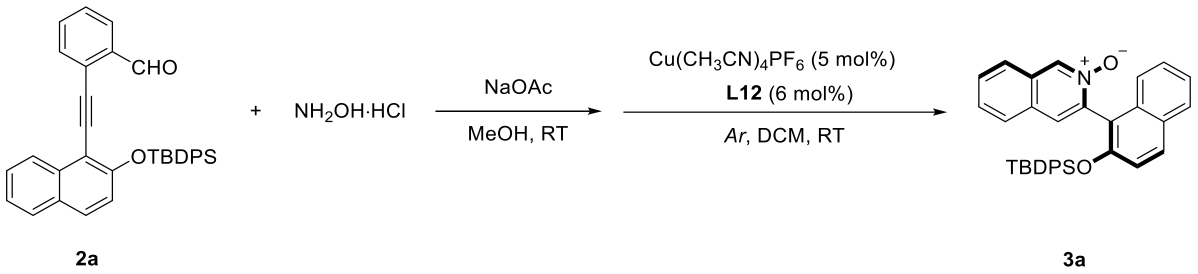

$NH_2OH{\cdot}HCl$ (13.9 mg, 0.2 mmol) and NaOAc (16.4 mg, 0.2 mmol) was added to a mixture of **2a** (51.1mg, 0.1 mmol) in MeOH (2 mL). The mixture was stirred at RT for 2h, then concentrated under reduced pressure. After that, to a 10 mL Schlenk tube under argon atmosphere, $Cu(CH_3CN)_4PF_6$ (1.9 mg, 5 mol%), **L12** (3.2 mg, 6 mol%) and dry DCM (1.0 mL) were added. The reaction was stirred for 24 h until completion (monitored by TLC), and the crude product was purified by column chromatography on silica gel (PE: EA=2:1) to afford **3a** (36.4 mg, 69% yield, 91.5: 8.5 er).

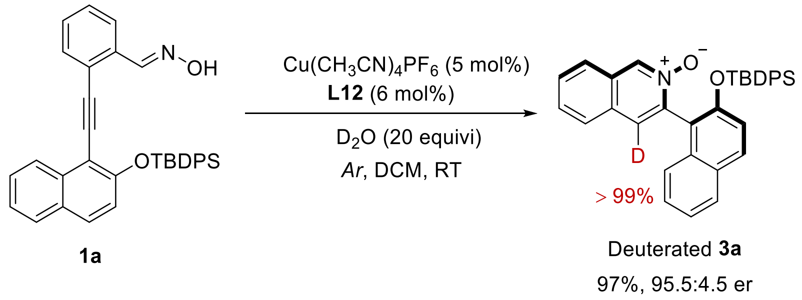

To a 10 mL Schlenk tube under argon atmosphere, $Cu(CH_3CN)_4PF_6$ (1.9 mg, 5 mol%), **L12** (3.2 mg, 6 mol%) and dry DCM (0.4 ml) were added, and the mixture was stirred at RT for 30 minutes. Subsequently, **1** (0.1 mmol) in dry DCM (0.6 mL) and D_2O (20 equiv.) were

added. The reaction was stirred for 24 h (monitored by TLC), and the mixture was directly purified by column chromatography on silica gel (PE: EA=2:1) to yield completely deuterated **3a** (D/H > 99%) at the C4 position of the isoquinoline ring, indicating that protonation had occurred during the reaction process.

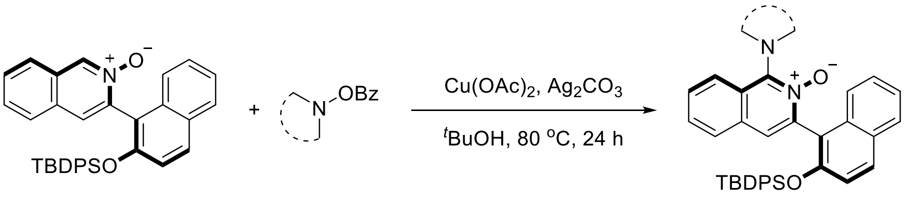

To a solution of Substates **3a** (52.6 mg, 0.1 mmol, 96:4 er) in *t*BuOH (1.0 ml) was added **4** (0.3mmol), $Cu(OAc)_2$ (1.9 mg, 10 mol%) and Ag_2CO_3 (2.8 mg, 10 mol%). The reaction mixture was stirred at 80 °C for 24 h (monitored by TLC) and then diluted with EA (3 mL) and H_2O (3 mL). Phases were separated and the aqueous phase was extracted twice with EA (3 mL). The combined organic phases were dried over $MgSO_4$, filtered and concentrated under reduced pressure. Then the mixture was directly purified by silica gel column chromatography (PE: EA=10:1 to 8:1) to afford the pure product.[2]

(*S*)-3-(2-((*tert*-butyldiphenylsilyl)oxy)naphthalen-1-yl)-1-morpholinoisoquinoline 2-oxide (5a)

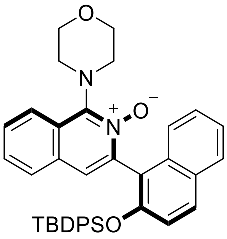

42 mg, 68% yield, pale-yellow solid, m.p: 213.9 – 216.3 °C; **HPLC** (Daicel Chiralpak IC, *n*-hexane/2-propanol = 80:20, 1.0 mL/min, at 254 nm): t_R = 8.84 min (major), t_R = 25.07 min (minor); er = 94:6, $[\alpha]D^{20}$ = +278.363 (*c* = 0.11, EA). **^{1}H NMR** (600 MHz, Chloroform-*d*) δ 8.19 (d, *J* = 9.0 Hz, 1H), 7.68 – 7.63 (m, 2H), 7.63 – 7.56 (m, 5H), 7.53 – 7.48 (m, 3H), 7.35 – 7.29 (m, 3H), 7.26 (td, *J* = 6.6, 1.8 Hz, 1H), 7.25 – 7.19 (m, 5H), 6.78 (d, *J* = 9.0 Hz, 1H), 4.29 – 2.49 (m, 8H), 0.66 (s, 9H). **^{13}C NMR** (150 MHz, Chloroform-*d*) δ 151.6, 149.5, 144.8, 135.7, 135.5, 133.7, 132.9, 132.7, 130.3, 130.1, 130.0, 129.8, 129.1, 128.5, 128.3, 127.95, 127.88, 127.6, 127.12, 127.08, 124.1, 123.9, 123.7, 120.4, 119.2, 67.8, 48.5, 26.2, 19.3. **HRMS** (ESI-TOF) m/z: [M + H]$^+$ Calcd for $C_{39}H_{39}N_2O_3Si^+$ 611.2725; Found 611.2723.

(*S*)-3-(2-((*tert*-butyldiphenylsilyl)oxy)naphthalen-1-yl)-1-thiomorpholinoisoquinoline 2-oxide (5b)

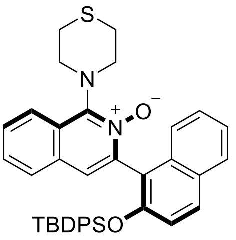

45.3 mg, 72% yield, pale-yellow solid, m.p: 258.7 – 259.9 °C; **HPLC** (Daicel Chiralpak IC, *n*-hexane/2-propanol = 80:20, 1.0 mL/min, at 254 nm): t_R = 6.01 min (major), t_R = 8.58 min (minor); er = 91:9, $[\alpha]D^{20}$ = +256.890 (*c* = 0.27, EA). **^{1}H NMR** (600 MHz, Chloroform-*d*) δ 8.13 (d, *J* = 8.4 Hz, 1H), 7.66 (t, *J* = 9.0 Hz, 2H), 7.61 – 7.55 (m, 5H), 7.52 – 7.47 (m, 3H), 7.35 – 7.24 (m, 4H), 7.24 – 7.19 (m, 5H), 6.78 (d, *J* = 9.0 Hz, 1H), 4.04 – 2.15 (m, 4H), 0.67 (s, 9H). **^{13}C NMR** (150 MHz, Chloroform-*d*) δ 151.6, 150.6, 144.7, 135.7, 135.6, 133.7, 132.9, 132.7, 130.3, 130.1, 130.0, 129.8, 129.1, 128.5, 128.3, 128.0, 127.90, 127.88, 127.6, 127.14, 127.08, 124.1, 123.9, 123.69, 123.67, 120.4, 119.2, 50.8, 28.5, 26.3, 19.3. **HRMS** (ESI-TOF) m/z: $[M + H]^+$ Calcd for $C_{39}H_{39}N_2O_2SSi^+$ 627.2497; Found 627.2500.

(*S*)-3-(2-((*tert*-butyldiphenylsilyl)oxy)naphthalen-1-yl)-1-(4-methylpiperidin-1-yl)isoquinoline 2-oxide (5c)

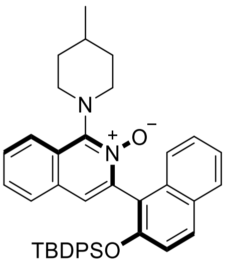

24.9 mg, 40% yield, orange oil; **HPLC** (Daicel Chiralpak ID, *n*-hexane/2-propanol = 95:5, 1.0 mL/min, at 254 nm): t_R = 9.21 min (major), t_R = 11.88 min (minor); er = 91:9, $[\alpha]D^{20}$ = +148.100 (*c* = 0.15, EA). **^{1}H NMR** (600 MHz, Chloroform-*d*) δ 8.17 (s, 1H), 7.65 – 7.60 (m, 4H), 7.59 – 7.56 (m, 2H), 7.54 (t, *J* = 7.8 Hz, 1H), 7.48 – 7.41 (m, 3H), 7.33 – 7.27 (m, 3H), 7.26 – 7.23 (m, 1H), 7.23 – 7.19 (m, 5H), 6.78 (d, *J* = 9.0 Hz, 1H), 3.85 – 2.61 (m, 3H), 1.71 – 1.53 (m, 4H), 1.38 – 1.31 (m, 2H), 0.95 (d, *J* = 6.6 Hz, 3H), 0.66 (s, 9H). **^{13}C NMR** (150 MHz, Chloroform-*d*) δ 151.5, 144.7, 135.8, 135.5, 133.8, 133.1, 132.7, 130.1, 130.02, 129.95, 129.8, 129.1, 128.1, 127.9, 127.84, 127.76, 127.0, 126.9, 124.3, 124.1, 123.9, 120.4, 119.6, 48.6, 35.2, 30.9, 26.2, 22.6, 19.2. **HRMS** (ESI-TOF) m/z: $[M + H]^+$ Calcd for $C_{41}H_{43}N_2O_2Si^+$ 623.3089; Found 623.3087.

(*S*)-3-(2-((*tert*-butyldiphenylsilyl)oxy)naphthalen-1-yl)-1-(pyrrolidin-1-yl)isoquinoline 2-oxide (5d)

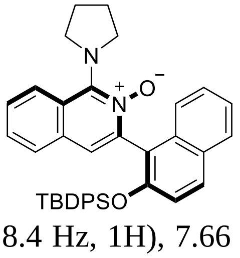

38.7 mg, 65% yield, pale-yellow solid, m.p: 180.0 – 181.7 °C; **HPLC** (Daicel Chiralpak ID, *n*-hexane/2-propanol = 95:5, 1.0 mL/min, at 254 nm): t_R = 7.32 min (major), t_R = 9.75 min (minor); er = 93:7, $[\alpha]D^{20}$ = -195.920 (*c* = 0.16, EA). **^{1}H NMR** (600 MHz, Chloroform-*d*) δ 8.20 (d, *J* = 8.4 Hz, 1H), 7.66 – 7.61 (m, 4H), 7.58 – 7.52 (m, 3H), 7.49 – 7.45 (m, 3H), 7.33 – 7.28 (m, 3H), 7.25 – 7.19 (m, 6H), 6.79 (d, *J* = 9.0 Hz, 1H), 3.55 – 3.49 (m, 2H), 3.37 – 3.32 (m, 2H), 2.00 – 1.94 (m, 4H), 0.64 (s, 9H). **^{13}C NMR** (150 MHz, Chloroform-*d*) δ 151.5, 149.7, 144.8,

135.8, 135.5, 133.8, 133.1, 132.7, 130.1, 130.02, 129.97, 129.5, 129.4, 129.2, 128.2, 128.1, 127.90, 127.86, 127.7, 127.0, 126.7, 124.6, 124.4, 123.9, 123.3, 120.4, 119.7, 48.6, 26.7, 26.2, 19.2. **HRMS** (ESI-TOF) m/z: $[M + H]^+$ Calcd for $C_{39}H_{39}N_2O_2Si^+$ 595.2776; Found 595.2779.

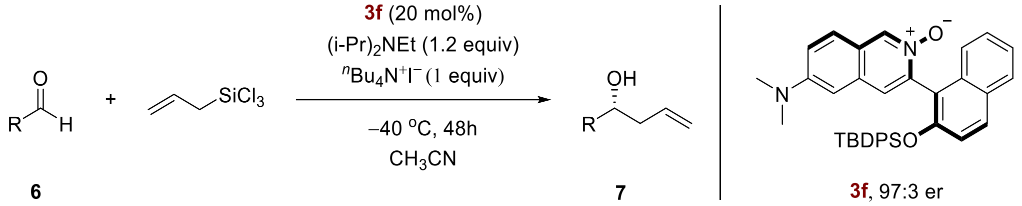

To a 25 mL Schlenk tube under argon atmosphere, **6** (21.3 mg, 0.2 mmol.), **3f** (22.8 mg, 20 mol%), DIPEA (73.9 mg, 0.2 mmol), TBAI (31.1 mg, 0.24 mmol.), allyltrichlorosilane (42.3 mg, 0.24 mmol) and acetonitrile (2.0 ml) were sequentially added to. The reaction mixture was stirred at -40 °C for 48 h, and then warmed to room temperature. The reaction was quenched with saturated aqueous $NaHCO_3$ (2.0 mL), and the aqueous layer was extracted with DCM (3×3.0 mL). The combined organic layers were washed with brine and dried over Na_2SO_4. The mixture was directly purified by silica gel column chromatography (PE: EA= 40:1 to 20:1) to afford the product **7**.[3] The absolute configuration of the chiral product **7a** was identified by comparison with the literature product.[4]

(*R*)-1-(naphthalen-2-yl)but-3-en-1-ol (7a)

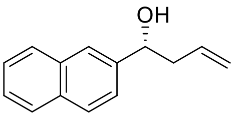

29.4 mg, 74% yield, pale-yellow oli; **HPLC** (Daicel Chiralpak OD-H, *n*-hexane/2-propanol = 95:5, 0.5 mL/min, at 254 nm): t_R = 30.50 min (major), t_R = 33.94 min (minor); er = 9:91, $[\alpha]D^{20}$ = +92.375 (*c* = 0.16, EA). **^{1}H NMR** (600 MHz, Chloroform-*d*) δ 7.78 – 7.71 (m, 4H), 7.43 – 7.36 (m, 3H), 5.79 – 5.70 (m, 1H), 5.10 (dd, *J* = 17.4, 1.8 Hz, 1H), 5.07 (dd, *J* = 10.2, 1.8 Hz, 1H), 4.82 (t, *J* = 6.6 Hz, 1H), 2.57 – 2.46 (m, 2H), 2.12 (d, *J* = 2.4 Hz, 1H). **^{13}C NMR** (150 MHz, Chloroform-*d*) δ 141.4, 134.5, 133.4, 133.1, 128.3, 128.1, 127.8, 126.3, 126.0, 124.6, 124.1, 118.7, 73.5, 43.9. **HRMS** (ESI-TOF) m/z: $[M + Na]^+$ Calcd for $C_{14}H_{14}NaO^+$ 221.0937.; Found 221.0946.

(*R*)-1-phenylbut-3-en-1-ol (7b)

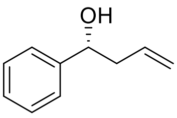

17.8 mg, 60% yield, pale-yellow oli; **HPLC** (Daicel Chiralpak OD-H, *n*-hexane/2-propanol = 99:1, 0.8 mL/min, at 254 nm): t_R = 15.86 min (major), t_R = 17.85 min (minor); er = 85.5:14.5, $[\alpha]D^{20}$ = +101.200 (*c* = 0.18, EA). **^{1}H NMR** (600 MHz, Chloroform-*d*) δ 7.28 – 7.16 (m, 5H), 5.78 – 5.68 (m, 1H), 5.12 – 5.04 (m, 2H), 4.70 – 4.60 (m, 1H), 2.49 – 2.38 (m, 2H), 2.02 (s, 1H). **^{13}C NMR** (150 MHz, Chloroform-

d) δ 144.0, 134.6, 128.5, 127.7, 125.9, 118.6, 73.4, 44.0. **HRMS** (ESI-TOF) m/z: $[M + H]^+$ Calcd for $C_{10}H_{13}O^+$ 149.0961.; Found 149.0963.

(*R*)-1-(4-methoxyphenyl)but-3-en-1-ol (7c)

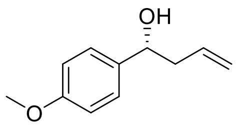

20.8 mg, 58% yield, pale-yellow oli; **HPLC** (Daicel Chiralpak OD-H, *n*-hexane/2-propanol = 95:5, 0.8 mL/min, at 254 nm): t_R = 12.66 min (major), t_R = 14.49 min (minor); er = 88.5:11.5, $[\alpha]D^{20}$ = +53.108 (*c* = 0.14, EA). **^{1}H NMR** (600 MHz, Chloroform-*d*) δ 7.23 – 7.19 (m, 2H), 6.83 – 6.79 (m, 2H), 5.77 – 5.68 (m, 1H), 5.11 – 5.03 (m, 2H), 4.61 (t, *J* = 6.6 Hz, 1H), 3.73 (s, 3H), 2.45 – 2.40 (m, 2H), 1.93 (s, 1H). **^{13}C NMR** (150 MHz, Chloroform-*d*) δ 159.2, 136.2, 134.8, 127.2, 118.4, 113.9, 73.1, 55.4, 43.9. **HRMS** (ESI-TOF) m/z: $[M + Na]^+$ Calcd for $C_{11}H_{14}NaO_2$ 201.0886.; Found 201.0888.

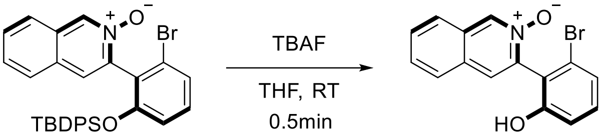

3aa **8aa**

To a stirred solution of **3aa** (55.5 mg, 0.1 mmol) in THF (1 mL) was added TBAF (0.5 equiv.). The resulting mixture was stirred at room temperature for 0.5 minute (monitored by TLC) and extracted with DCM. The organic phase was separated, dried over Na_2SO_4, and concentrated under vacuum. The crude product was purified by silica gel column chromatography (PE: EA = 1:1) to afford **8aa** as a pale-yellow solid (29.8 mg, 94% yield, 12:88 er). (Note: product **8aa** was easy to racemize in the condition, it is better to quench it as soon as possible)

(*R*)-3-(2-bromo-6-hydroxyphenyl)isoquinoline 2-oxide (8aa)

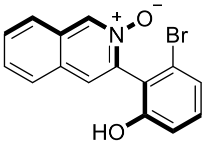

29.8 mg, 94% yield, pale-yellow solid, m.p: 167.4 – 170.5 °C; **HPLC** (Daicel Chiralpak AD-H, *n*-hexane/2-propanol = 70:30, 1.0 mL/min, at 254 nm): t_R = 12.35 min (major), t_R = 19.24 min (minor); er = 12:88, $[\alpha]D^{20}$ = +102.677 (c = 0.14, EA). **^{1}H NMR** (600 MHz, DMSO-d_6) δ 10.08 (s, 1H), 9.06 (s, 1H), 7.98 (s, 1H), 7.95 (d, *J* = 7.8 Hz, 1H), 7.90 (d, *J* = 8.4 Hz, 1H), 7.69 – 7.65 (m, 1H), 7.64 – 7.60 (m, 1H), 7.23 (t, *J* = 8.4 Hz, 1H), 7.17 (dd, *J* = 7.8, 0.6 Hz, 1H), 6.96 (dd, *J* = 7.8, 0.6 Hz, 1H). **^{13}C NMR** (150 MHz, DMSO-d_6) δ 157.2, 143.9, 135.4, 131.3, 129.3, 129.2, 128.3, 127.9, 126.7, 126.3, 124.42,

124.38, 122.52, 122.47, 115.0. **HRMS** (ESI-TOF) m/z: $[M + H]^+$ Calcd for $C_{15}H_{11}BrNO_2^+$ 315.9968; Found 315.9961.

6. Single crystal X-ray diffraction analysis and crystal data

To a 5 mL tube containing **3g** (20 mg) was added a 1:3 mixture of DCM and PE (4 mL). A clear solution was obtained through ultrasound treatment and was kept at room temperature for 2 day to get crystal of **3g**, which were characterized by single crystal X-ray diffraction. The data were collected by an Agilent Gemini. **3g** contains the supplementary crystallographic data for this paper. These data can be obtained free of charge *via* www.ccdc.cam.ac.uk/data_request/cif.

(Ellipsoid contour probability 50%)

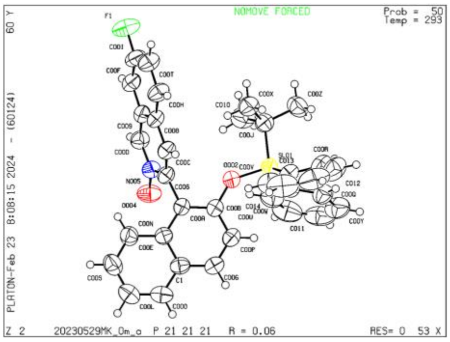

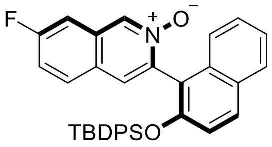

3g

Identification code	**3g**	
Chemical formula	$C_{35}H_{30}FNO_2Si$	
Formula weight	543.69 g/mol	
Temperature	293(2) K	
Wavelength	1.54178 Å	
Crystal system	orthorhombic	
Space group	P 21 21 21	
Unit cell dimensions	a = 10.7384(3) Å	α = 90°
	b = 11.9202(3) Å	β = 90°
	c = 22.9670(6) Å	γ = 90°
Volume	2939.87(13) Å^3	
Z	4	
Density (calculated)	1.228 g/cm^3	
Absorption coefficient	1.009 mm^{-1}	
F(000)	1144	
Theta range for data collection	3.85 to 68.44°	
Index ranges	-9<=h<=12, -13<=k<=14, -25<=l<=27	
Reflections collected	15539	
Independent reflections	5231 [R(int) = 0.0611]	
Coverage of independent reflections	99.0%	
Absorption correction	Multi-Scan	

Structure solution technique	direct methods	
Structure solution program	SHELXT 2014/5 (Sheldrick, 2014)	
Refinement method	Full-matrix least-squares on F^2	
Refinement program	SHELXL-2016/6 (Sheldrick, 2016)	
Function minimized	Σ w(F_o^2- F_c^2)^2	
Data / restraints / parameters	5231 / 0 / 364	
Goodness-of-fit on F2	1.034	
Final R indices	3962 data; I>2σ(I)	R1 = 0.0637, wR2 = 0.1574
	all data	R1 = 0.0826, wR2 = 0.1823
Weighting scheme	w=1/[σ^2(F_o^2) + (0.1003P)^2 + 0.3128P]<br>where P= (F_o^2+2F_c^2)/3	
Absolute structure parameter	0.01(3)	
Largest diff. peak and hole	0.217 and -0.435 eÅ^{-3}	
R.M.S. deviation from mean	0.071 eÅ^{-3}	

7. Thermal stability experiments

Enantiomerically enriched (*S*)-**3a** (1 mg, 96:4 er) and (*S*)-**3af** (1 mg, 36:64 er) dissolved in Toluene (1.0 mL) for **3a** and Mesitylene for **3af** (**3a** in Ar). The vial was fitted with a puncturable sealed cap and placed into an aluminum heating block. The experiment was performed at 100 ^{o}C (**3a**) and 90 ^{o}C (**3af**). At given time, a small aliquot (one microliter) was removed from the vial and concentrated in an HPLC vial. The residue was dissolved in a 30% iso-propanol/hexanes solution (1.0 mL) and injected into the HPLC system equipped with a Chiralpak AD-H column (1.0 ml/min, 70:30 *n*-hexanes: 2-propanol, 25 ^{o}C). The time points and corresponding enantiomeric excess of the entire system were plotted to determine an observed rate constant. The rotational barrier ($\Delta G^{\neq}_{ent}$), rate constants for enantiomerization (k_{ent}) and racemization (k_{rac}), and half-life for racemization ($t_{1/2rac}$) were calculated based on the following Eyring equations:[5]

$$t_{1/2rac} = \ln 2/k_{rac}$$

$$\Delta G^{\neq}_{ent} = RT\ln(k_{ent}h/k_BT)$$

where the transmission coefficient κ is set as 1, Boltzmann constant $k_B = 1.3806503\times10^{-23}$ J/K, Planck constant $h = 6.62606876 \times 10^{-34}$ J·s, idea gas constant $R = 8.314472$ J/(mol·K).

Table S3. | Change of enantiomer ratio with time for **3a** (100 ^{o}C in Toluene)

Time (s)	ee	First order racemization $\ln(ee_0/ee_t)$
0	92	0
3600	88	0.04445
7200	84	0.09097
10800	82	0.11507
18000	76	0.19106
25200	69	0.28768
32400	58	0.46135
43200	49	0.62997
54000	40	0.83291
64800	32	1.05605
75600	28	1.18958
86400	24	1.34373
97200	19	1.57735
108000	14	1.88273
118800	12	2.03688
129600	10	2.2192

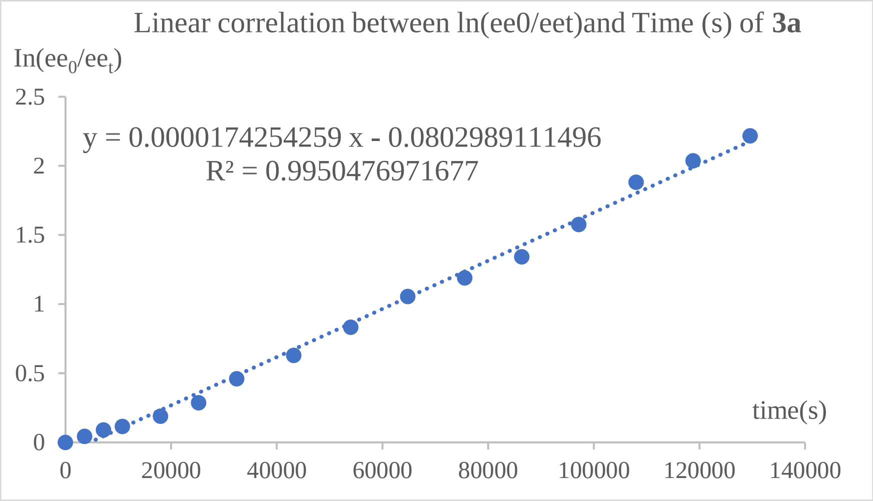

$k_{racemization}$ (100 °C) = 0.0000174254259 s^{-1}

$k_{enantiomerization}$ (100 °C) = 0.00000871271295 s^{-1}

Employing the Eyring equation:

$$\Delta G^{\ddagger} = RT \ln\left(\frac{k_B \times T}{k_{ent} \times h}\right)$$

$$\Delta G^{\ddagger} = 8.314\ J \bullet mol^{-1} \bullet K^{-1} \times 373.15\ K \times \ln\left(\frac{1.381\times10^{-23} J\bullet K^{-1}\times 373.15 K}{8.71271295\times10^{-6} S^{-1}\times 6.626\times10^{-34} J\bullet S}\right)$$

$$\Delta G^{\ddagger} = 128.230\ kJ \bullet mol^{-1} = 30.6\ kcal \bullet mol^{-1}$$

$$T_{1/2} = \frac{\ln 2}{kracemization} = 11.0\ \text{h}$$

Table S4. | Change of enantiomer ratio with time for **3af** (90 °C in mesitylene)

Time (s)	ee	First order racemization ln(ee_0/ee_t)
0	26	0
3600	22	0.16705
10800	16	0.48551
18000	11	0.8602
25200	7	1.31219
32400	4	1.8718
43200	2	2.56495

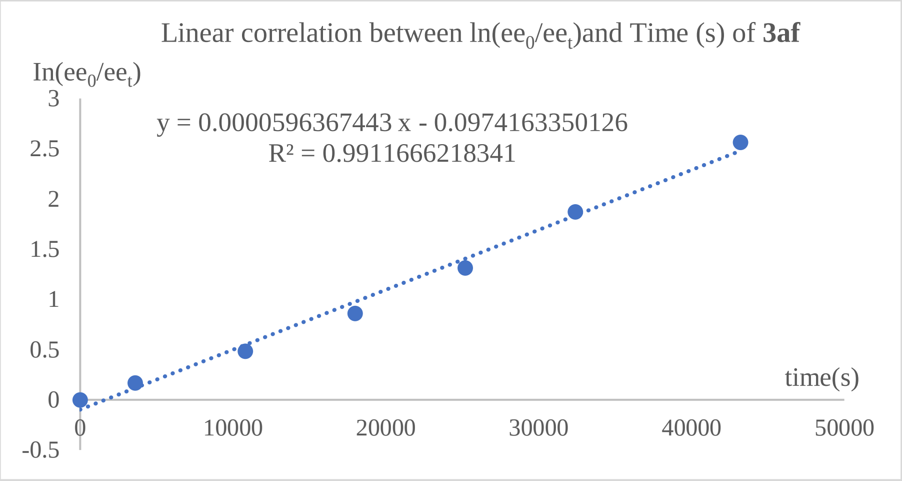

$k_{racemization}$ (90 °C) = 0.0000596367443 s^{-1}

$k_{enantiomerization}$ (90 °C) = 0.00002981837215 s^{-1}

Employing the Eyring equation:

$$\Delta G^{\ddagger} = RT \ln\left(\frac{k_B \times T}{k_{ent} \times h}\right)$$

$$\Delta G^{\ddagger} = 8.314\ J \bullet mol^{-1} \bullet K^{-1} \times 363.15\ K \times \ln\left(\frac{1.381\times10^{-23} J\bullet K^{-1} \times 363.15K}{2.981837215\times10^{-5} S^{-1} \times 6.626\times10^{-34} J\bullet S}\right)$$

$$\Delta G^{\ddagger} = 120.997\ kJ \bullet mol^{-1} = 28.9\ kcal \bullet mol^{-1}$$

$$T_{1/2} = \frac{\ln 2}{kracemization} = 3.2\ \text{h}$$

8. DFT calculations

Computational methods

All density functional theory (DFT) calculations were performed with Gaussian 16 program software.[6] A SMD solvation model was utilized to simulate the solvent effect of dichloromethane (DCM) solution.[7] Geometry optimizations were operated to locate all of the stationary points in DCM solution, using M06 density functional theory method with a mixed basis set of LANL2DZ for Cu and 6-31G(d) for other atoms.[8] In the meantime, the stability of the DFT wave-function of the auxiliary Kohn–Sham determinant was examined.[9] Harmonic vibrational frequency calculations were conducted at the same level of theory used for the geometry optimizations to characterize all stationary points. Herein, minima have zero imaginary frequencies, and transition states (TS) have one imaginary vibrational frequency. For every transition state, intrinsic reaction coordinate (IRC) analysis was performed to confirm that it indeed connects two minima on the potential energy surface.[10] Single point energies were calculated with the M06 functional in combination with def2-TZVP basis set for all atoms, along with the SMD solvation model in DCM solution.[11] The Gibbs energy corrections from the frequency calculations were added to the single-point energies to obtain the Gibbs free energies (ΔG) with larger basis sets.

Computational results

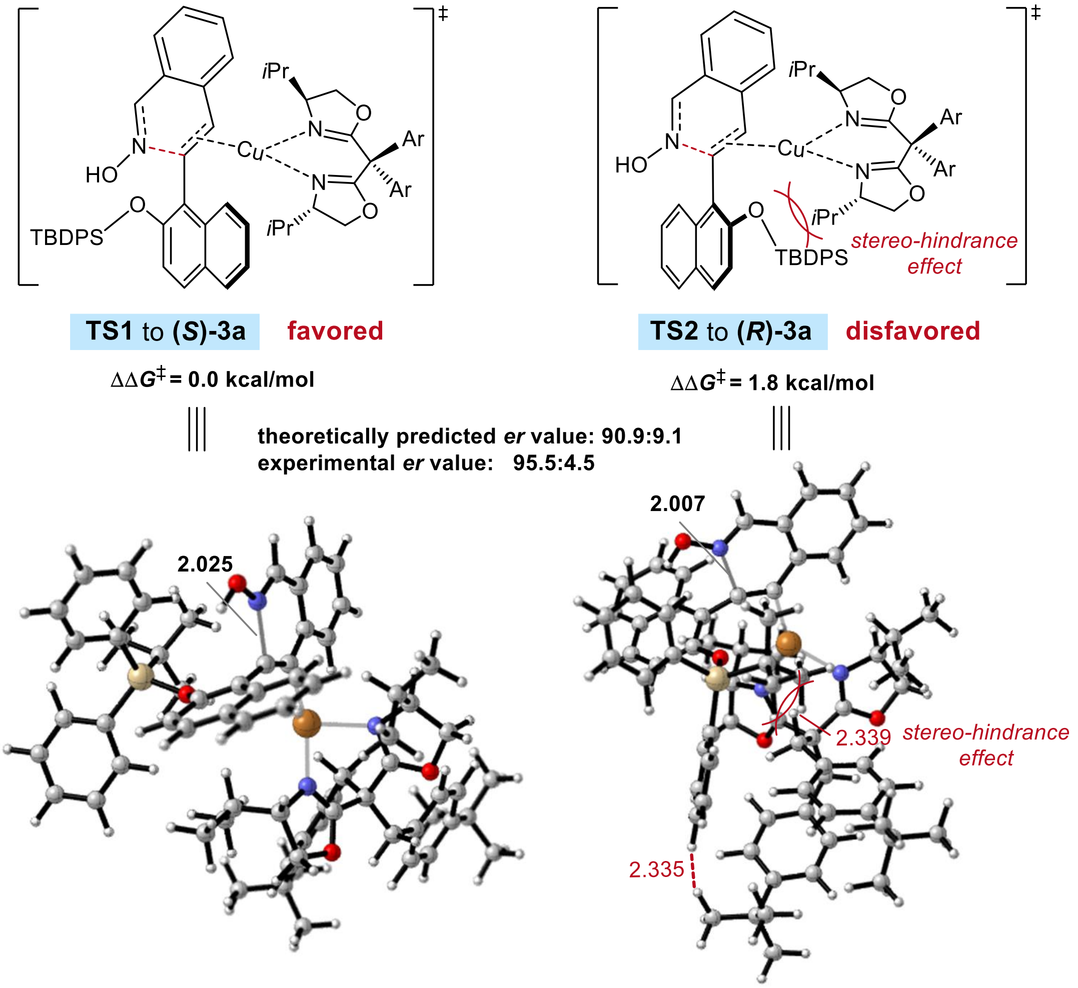

Figure S1. DFT-optimized structures and relative free Gibbs energies for the enantiomeric transition states **TS1** and **TS2**. density functional theory (DFT) calculations were carried out at the SMD(DCM)-M06/def2-TZVP//SMD(DCM)-M06/6-31G(d)/LANL2DZ(Cu) level of theory.

Energies and cartesian coordinates of all stationary points
TS1

Zero-point correction= 1.351241 (Hartree/Particle)
Thermal correction to Energy= 1.429083
Thermal correction to Enthalpy= 1.430027
Thermal correction to Gibbs Free Energy= 1.235011
Sum of electronic and zero-point Energies= -3663.283140
Sum of electronic and thermal Energies= -3663.205298
Sum of electronic and thermal Enthalpies= -3663.204354
Sum of electronic and thermal Free Energies= -3663.399370
SCF Done = -5110.13788568
Imaginary frequency = 383.37*i*

Standard orientation:

Center Number	Atomic Number	Atomic Type	Coordinates (Angstroms) X	Y	Z
1	14	0	2.572150	7.543925	0.048508
2	6	0	0.198261	5.452619	-4.401100
3	8	0	1.558867	6.327626	-0.566063
4	8	0	-1.925336	8.438920	-2.123016
5	7	0	-2.008240	7.577151	-1.063799
6	6	0	-1.102837	5.777119	-0.863645
7	6	0	-2.492530	5.842507	1.201233
8	6	0	-3.103561	7.106007	1.004398
9	6	0	-0.224387	5.763959	-1.993878
10	6	0	1.122926	6.085529	-1.808345
11	6	0	-1.576230	5.284735	0.225346
12	6	0	-2.863204	7.918115	-0.168713
13	1	0	-3.423795	8.850671	-0.291986
14	6	0	-0.712846	5.447780	-3.305927
15	6	0	-3.984352	7.610659	1.971010
16	1	0	-4.448688	8.582772	1.803043
17	6	0	1.556606	5.783332	-4.164508
18	1	0	2.245277	5.788017	-5.009567
19	6	0	-2.787654	5.134725	2.374314
20	1	0	-2.318840	4.161393	2.520687
21	6	0	-4.257038	6.894463	3.125070
22	6	0	1.988091	7.674335	1.844739
23	6	0	2.297579	9.066873	-1.008738
24	6	0	-1.593395	4.837028	-5.913278
25	1	0	-1.947623	4.598870	-6.914406
26	6	0	4.342556	6.941930	-0.040283
27	6	0	-2.070624	5.139985	-3.556614
28	1	0	-2.780220	5.150020	-2.727942
29	6	0	-0.270331	5.138166	-5.698269
30	1	0	0.442576	5.144677	-6.523046
31	6	0	2.011563	6.093722	-2.910280
32	1	0	3.061009	6.334147	-2.741231
33	6	0	3.345221	9.731451	-1.661677

34	1	0	4.370399	9.377856	-1.545921
35	6	0	5.429011	7.769231	0.285659
36	1	0	5.260137	8.812922	0.559439
37	6	0	-2.497681	4.842827	-4.831072
38	1	0	-3.548046	4.612201	-5.005985
39	6	0	-3.653649	5.651819	3.327326
40	1	0	-3.862578	5.085871	4.233424
41	6	0	0.991276	9.539060	-1.218563
42	1	0	0.147585	9.038958	-0.739380
43	6	0	4.612467	5.606521	-0.369802
44	1	0	3.784098	4.937903	-0.612243
45	6	0	0.741473	10.636670	-2.036021
46	1	0	-0.281697	10.979817	-2.185035
47	6	0	2.121890	6.293586	2.494295
48	1	0	1.478137	5.547059	2.003617
49	1	0	1.818721	6.347670	3.553071
50	1	0	3.156293	5.919076	2.467667
51	6	0	3.102444	10.836026	-2.474522
52	1	0	3.932683	11.338209	-2.969082
53	6	0	2.856711	8.681582	2.598455
54	1	0	3.907690	8.361893	2.651803
55	1	0	2.495243	8.789699	3.634277
56	1	0	2.827659	9.680648	2.135475
57	6	0	0.524999	8.113971	1.906843
58	1	0	0.387180	9.143293	1.544161
59	1	0	0.167233	8.083857	2.949932
60	1	0	-0.122646	7.450840	1.314505
61	6	0	1.800555	11.291043	-2.661318
62	1	0	1.609118	12.152534	-3.299518
63	6	0	6.733640	7.283528	0.267160
64	1	0	7.562141	7.943759	0.519487
65	6	0	6.978180	5.954789	-0.071401
66	1	0	7.998490	5.574419	-0.085426
67	6	0	5.914524	5.113723	-0.387227
68	1	0	6.099327	4.070826	-0.645058
69	1	0	-4.938691	7.300957	3.869532
70	1	0	-1.169526	8.104012	-2.646733
71	6	0	1.338353	0.196244	-1.490856
72	8	0	0.534124	-0.391868	-0.435847
73	6	0	-0.158443	0.597432	0.122584
74	6	0	1.040608	1.703155	-1.407090
75	1	0	1.025867	-0.261605	-2.434074
76	7	0	0.029833	1.777327	-0.341703
77	1	0	2.381664	-0.071339	-1.290547
78	6	0	-1.111419	0.256864	1.262340
79	6	0	-2.457484	0.814118	0.827190
80	8	0	-3.401782	-0.072534	0.483339
81	6	0	-4.600705	0.702932	0.242120
82	6	0	-4.097313	2.151238	0.169261
83	1	0	-5.064164	0.325015	-0.675004
84	1	0	-5.279145	0.526495	1.085891

85	7	0	-2.737682	2.056615	0.731456
86	6	0	0.426033	0.445801	3.362654
87	6	0	1.621283	-0.032090	2.816351
88	6	0	0.268591	0.353339	4.744968
89	6	0	2.598372	-0.601915	3.622744
90	1	0	1.791642	0.012497	1.740220
91	6	0	1.250695	-0.212152	5.553160
92	1	0	-0.659985	0.703473	5.199976
93	6	0	2.435238	-0.715664	5.010191
94	1	0	3.507773	-0.976770	3.151979
95	1	0	1.067121	-0.271313	6.624398
96	6	0	-1.693174	-1.728376	2.831777
97	6	0	-2.910868	-1.316659	3.374759
98	6	0	-0.916511	-2.606690	3.594814
99	6	0	-3.309909	-1.727353	4.646273
100	1	0	-3.567186	-0.654020	2.810400
101	6	0	-1.318441	-3.022493	4.855106
102	1	0	0.043666	-2.946937	3.202639
103	6	0	-2.518913	-2.576715	5.423117
104	1	0	-4.262016	-1.364082	5.029142
105	1	0	-0.666648	-3.691088	5.418816
106	6	0	3.512767	-1.396671	5.849239
107	6	0	4.846855	-0.663444	5.676870
108	6	0	3.166250	-1.411917	7.335733
109	6	0	3.664896	-2.848288	5.380460
110	1	0	5.182324	-0.654824	4.631135
111	1	0	4.769675	0.379877	6.015164
112	1	0	5.629650	-1.155398	6.272688
113	1	0	2.238267	-1.966789	7.535450
114	1	0	3.972729	-1.905864	7.895384
115	1	0	3.054323	-0.396734	7.742367
116	1	0	4.421256	-3.368117	5.986855
117	1	0	2.714592	-3.393437	5.481168
118	1	0	3.979299	-2.910903	4.329815
119	6	0	-2.887620	-2.997059	6.842597
120	6	0	-1.813667	-2.477155	7.805796
121	6	0	-4.235952	-2.433785	7.283455
122	6	0	-2.954830	-4.524872	6.932321
123	1	0	-0.821205	-2.888237	7.574304
124	1	0	-1.742945	-1.380196	7.758356
125	1	0	-2.059139	-2.757591	8.840811
126	1	0	-5.056319	-2.781462	6.639457
127	1	0	-4.456016	-2.765470	8.307773
128	1	0	-4.241822	-1.334429	7.284684
129	1	0	-3.212589	-4.834190	7.955911
130	1	0	-3.721681	-4.926070	6.254145
131	1	0	-1.997151	-4.996465	6.674512
132	29	0	-1.002257	3.405456	0.136643
133	6	0	2.245901	2.599541	-1.109669
134	6	0	3.221558	2.553397	-2.277674
135	6	0	2.922868	2.266700	0.211438

136	1	0	1.842592	3.626678	-1.034380
137	1	0	2.725758	2.807310	-3.225739
138	1	0	4.046924	3.263938	-2.131078
139	1	0	3.669545	1.554280	-2.389398
140	1	0	2.224956	2.347564	1.057225
141	1	0	3.348696	1.251422	0.214046
142	1	0	3.753343	2.959594	0.404532
143	1	0	0.577108	2.061315	-2.340764
144	6	0	-4.067921	2.765748	-1.234703
145	6	0	-5.480489	3.014346	-1.743709
146	6	0	-3.262722	1.942150	-2.230691
147	1	0	-3.569826	3.744254	-1.114504
148	1	0	-6.067542	3.615653	-1.035639
149	1	0	-5.455393	3.552400	-2.701752
150	1	0	-6.021770	2.071811	-1.915278
151	1	0	-3.267263	2.425386	-3.217408
152	1	0	-2.211914	1.834393	-1.922864
153	1	0	-3.684691	0.934289	-2.362736
154	1	0	-4.702646	2.805391	0.813276
155	6	0	-1.164608	-1.272060	1.493503
156	1	0	-1.734965	-1.725373	0.671311
157	1	0	-0.145007	-1.658789	1.393562
158	6	0	-0.680195	1.047771	2.539140
159	1	0	-1.567977	1.146530	3.177099
160	1	0	-0.418966	2.073515	2.231871

TS2

Zero-point correction= 1.352332 (Hartree/Particle)
Thermal correction to Energy= 1.429270
Thermal correction to Enthalpy= 1.430214
Thermal correction to Gibbs Free Energy= 1.241033
Sum of electronic and zero-point Energies= -3663.286296
Sum of electronic and thermal Energies= -3663.209358
Sum of electronic and thermal Enthalpies= -3663.208414
Sum of electronic and thermal Free Energies= -3663.397596
SCF Done = -5110.14104670
Imaginary frequency = 394.67*i*

Standard orientation:

Center Number	Atomic Number	Atomic Type	Coordinates (Angstroms) X	Y	Z
1	14	0	-1.629755	1.362014	-2.425895
2	6	0	2.149026	5.237934	-3.304916
3	8	0	-0.998091	2.864722	-1.961007
4	8	0	-1.763834	5.850673	-4.730669
5	7	0	-2.176401	6.129189	-3.457554
6	6	0	-1.277967	5.569416	-1.752174
7	6	0	-3.251520	6.774218	-0.858831

8	6	0	-3.862590	7.110026	-2.093453
9	6	0	-0.078424	4.992327	-2.282850
10	6	0	0.011078	3.607974	-2.429716
11	6	0	-2.035438	5.983323	-0.797927
12	6	0	-3.305143	6.732490	-3.374048
13	1	0	-3.850762	6.988222	-4.288303
14	6	0	0.995951	5.837101	-2.721143
15	6	0	-5.048896	7.856723	-2.105115
16	1	0	-5.508203	8.095766	-3.064371
17	6	0	2.192679	3.827938	-3.444103
18	1	0	3.072992	3.377525	-3.902786
19	6	0	-3.841863	7.251898	0.318563
20	1	0	-3.349677	7.026707	1.264600
21	6	0	-5.623224	8.297810	-0.924384
22	6	0	-3.411637	1.440144	-1.788031
23	6	0	-0.570699	0.034370	-1.628991
24	6	0	3.150604	7.424443	-3.607056
25	1	0	3.975121	8.050122	-3.943576
26	6	0	-1.523312	1.300477	-4.295553
27	6	0	0.950246	7.246927	-2.606033
28	1	0	0.068708	7.716711	-2.170102
29	6	0	3.217600	6.058313	-3.737752
30	1	0	4.091882	5.580582	-4.180494
31	6	0	1.160509	3.031004	-3.024303
32	1	0	1.218418	1.950195	-3.142926
33	6	0	-0.874538	-1.330945	-1.760253
34	1	0	-1.764010	-1.643153	-2.310819
35	6	0	-1.113274	0.163795	-5.003818
36	1	0	-0.840366	-0.745276	-4.465064
37	6	0	2.004972	8.018552	-3.037185
38	1	0	1.954518	9.101675	-2.937308
39	6	0	-5.008450	8.002648	0.293334
40	1	0	-5.442823	8.361438	1.225110
41	6	0	0.585018	0.378953	-0.910327
42	1	0	0.843953	1.430487	-0.764700
43	6	0	-1.841271	2.455110	-5.029652
44	1	0	-2.160989	3.360072	-4.508929
45	6	0	1.417088	-0.596882	-0.367336
46	1	0	2.310690	-0.304211	0.184774
47	6	0	-4.183655	2.522302	-2.545360
48	1	0	-3.714687	3.513155	-2.436351
49	1	0	-5.207317	2.595899	-2.142499
50	1	0	-4.269113	2.300099	-3.618710
51	6	0	-0.053550	-2.310990	-1.207932
52	1	0	-0.309398	-3.363071	-1.325305
53	6	0	-4.092762	0.087010	-2.001009
54	1	0	-4.059220	-0.237545	-3.053331
55	1	0	-5.154797	0.152265	-1.713255
56	1	0	-3.636747	-0.701039	-1.383323
57	6	0	-3.422346	1.780245	-0.297670
58	1	0	-2.885193	1.029432	0.302727

59	1	0	-4.462515	1.808572	0.069022
60	1	0	-2.973652	2.768504	-0.100400
61	6	0	1.099566	-1.944421	-0.517327
62	1	0	1.750755	-2.710515	-0.095553
63	6	0	-1.033117	0.175191	-6.394839
64	1	0	-0.714656	-0.719828	-6.927516
65	6	0	-1.350190	1.331424	-7.102171
66	1	0	-1.280869	1.341932	-8.189001
67	6	0	-1.752277	2.476484	-6.416977
68	1	0	-1.997510	3.387780	-6.961910
69	1	0	-6.542328	8.879628	-0.948932
70	1	0	-0.902314	5.400375	-4.608685
71	6	0	2.951107	4.308445	1.784637
72	8	0	2.158547	3.632229	2.791845
73	6	0	0.902264	3.622756	2.338530
74	6	0	1.908829	4.940624	0.853601
75	1	0	3.566504	3.547426	1.290352
76	7	0	0.660079	4.273990	1.262921
77	1	0	3.604903	5.019882	2.299332
78	6	0	-0.113432	2.809694	3.127316
79	6	0	-1.256421	3.753436	3.481806
80	8	0	-1.608655	3.843158	4.766458
81	6	0	-2.729912	4.759142	4.834418
82	6	0	-2.983819	5.157818	3.371377
83	1	0	-3.560887	4.224044	5.308473
84	1	0	-2.432901	5.595564	5.474205
85	7	0	-1.928242	4.442843	2.636860
86	6	0	1.127789	0.800519	4.215034
87	6	0	2.174366	0.526416	3.334155
88	6	0	0.600619	-0.276307	4.936105
89	6	0	2.629910	-0.776912	3.137993
90	1	0	2.638338	1.335813	2.770579
91	6	0	1.056682	-1.571941	4.743586
92	1	0	-0.213009	-0.097200	5.640704
93	6	0	2.068584	-1.859726	3.818486
94	1	0	3.440159	-0.936850	2.428108
95	1	0	0.592070	-2.379441	5.310587
96	6	0	-1.504387	0.628609	2.651080
97	6	0	-1.357977	-0.636950	2.070524
98	6	0	-2.463874	0.766714	3.651581
99	6	0	-2.134213	-1.712287	2.474177
100	1	0	-0.598484	-0.781092	1.300915
101	6	0	-3.239056	-0.318249	4.062885
102	1	0	-2.611677	1.722372	4.153488
103	6	0	-3.093471	-1.582531	3.488921
104	1	0	-1.974980	-2.678713	1.992361
105	1	0	-3.966913	-0.156100	4.856099
106	6	0	2.476595	-3.307320	3.564187
107	6	0	2.925425	-3.965837	4.872449
108	6	0	1.266514	-4.063980	3.002679
109	6	0	3.620017	-3.419498	2.559503

110	1	0	3.793470	-3.443237	5.299386
111	1	0	2.129035	-3.970121	5.628480
112	1	0	3.215212	-5.011207	4.690831
113	1	0	0.921098	-3.607351	2.062079
114	1	0	1.530798	-5.111900	2.796598
115	1	0	0.422168	-4.062170	3.706294
116	1	0	3.870851	-4.477847	2.402485
117	1	0	3.351358	-2.994671	1.580887
118	1	0	4.528550	-2.911633	2.913713
119	6	0	-3.913311	-2.795239	3.919306
120	6	0	-4.875684	-2.469949	5.058481
121	6	0	-4.733585	-3.302186	2.728206
122	6	0	-2.968814	-3.905965	4.392734
123	1	0	-4.345519	-2.125815	5.958033
124	1	0	-5.605035	-1.698669	4.772768
125	1	0	-5.440169	-3.372286	5.332051
126	1	0	-4.093939	-3.594193	1.884273
127	1	0	-5.324945	-4.182747	3.019767
128	1	0	-5.429550	-2.529017	2.371953
129	1	0	-3.546775	-4.777332	4.734218
130	1	0	-2.299737	-4.246108	3.590510
131	1	0	-2.344688	-3.562122	5.230977
132	29	0	-1.249548	4.961738	0.723024
133	6	0	1.776826	6.464533	0.947858
134	6	0	3.038542	7.138584	0.427148
135	6	0	1.426643	6.949065	2.347976
136	1	0	0.943011	6.729300	0.272938
137	1	0	3.302547	6.783524	-0.579838
138	1	0	2.901511	8.227002	0.372620
139	1	0	3.898271	6.951382	1.088071
140	1	0	0.481627	6.517384	2.709082
141	1	0	2.214369	6.703303	3.076145
142	1	0	1.313327	8.041475	2.353508
143	1	0	2.115053	4.683985	-0.197103
144	1	0	-2.815138	6.238906	3.238180
145	6	0	-4.376087	4.830722	2.822567
146	6	0	-5.417889	5.718810	3.488039
147	6	0	-4.746948	3.360095	2.931118
148	1	0	-4.336875	5.083681	1.748565
149	1	0	-5.160897	6.784647	3.400613
150	1	0	-6.405772	5.570743	3.032096
151	1	0	-5.514087	5.487404	4.560082
152	1	0	-3.997815	2.713219	2.454165
153	1	0	-4.860411	3.034831	3.976745
154	1	0	-5.709111	3.178257	2.433179
155	6	0	-0.648344	1.747839	2.120491
156	1	0	-1.182286	2.292701	1.325548
157	1	0	0.225170	1.297587	1.630482
158	6	0	0.514435	2.168985	4.397127
159	1	0	-0.275933	2.072826	5.147586
160	1	0	1.241168	2.875194	4.819981

9. Evaluation of the antitumor activity of heteroaromatic *N*-oxides

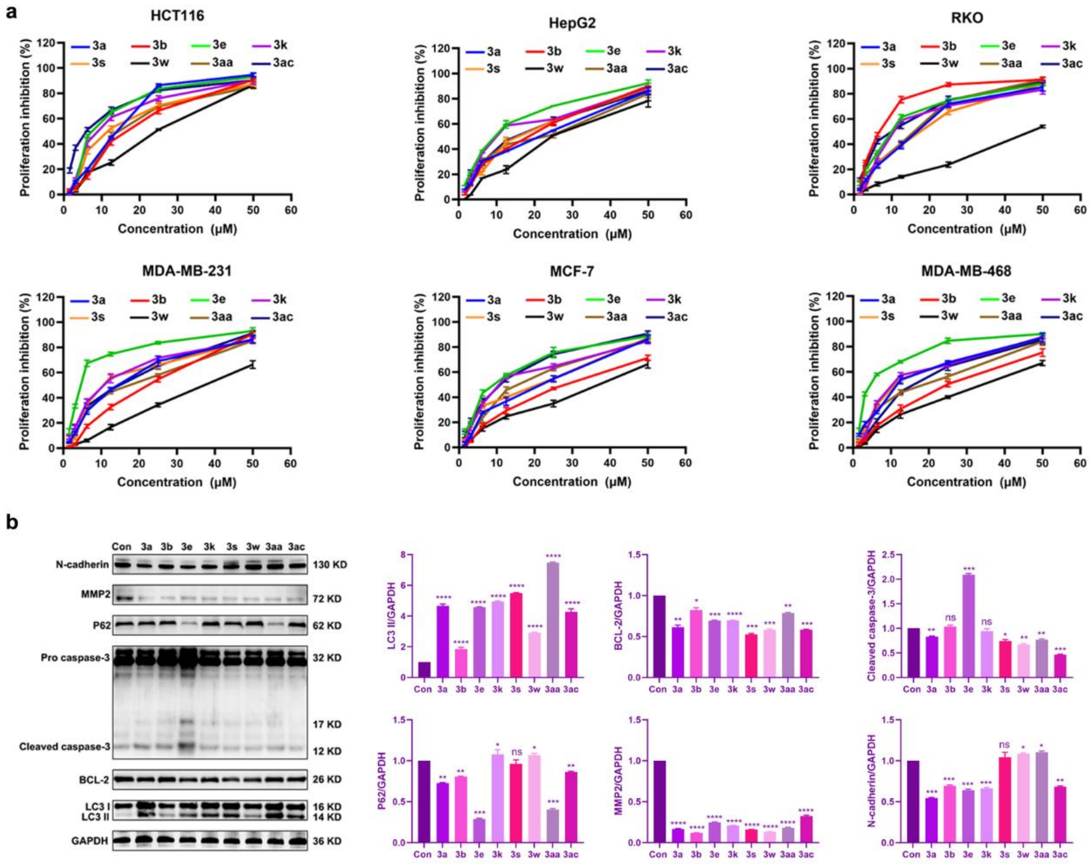

Figure S2. a) The toxicity of heteroaromatic *N*-oxides was examined by the MTT assay in tumor cells. b) WB experiment was conducted to evaluate the impact of the derivatives on cell apoptosis-related proteins and tumor metastasis-related proteins in MDA-MB-468 cells. GAPDH was used as a loading control. Quantitative and representative images of the expression levels of relevant proteins are shown. These results are consistent with those of at least three different experiments. ns, not significant, *, $P < 0.05$, **, $P < 0.01$, ***, $P < 0.001$, ****, $P < 0.0001$ Statistical significance was determined relative to the appropriate control groups.

10. NMR spectra and HPLC chromatograms

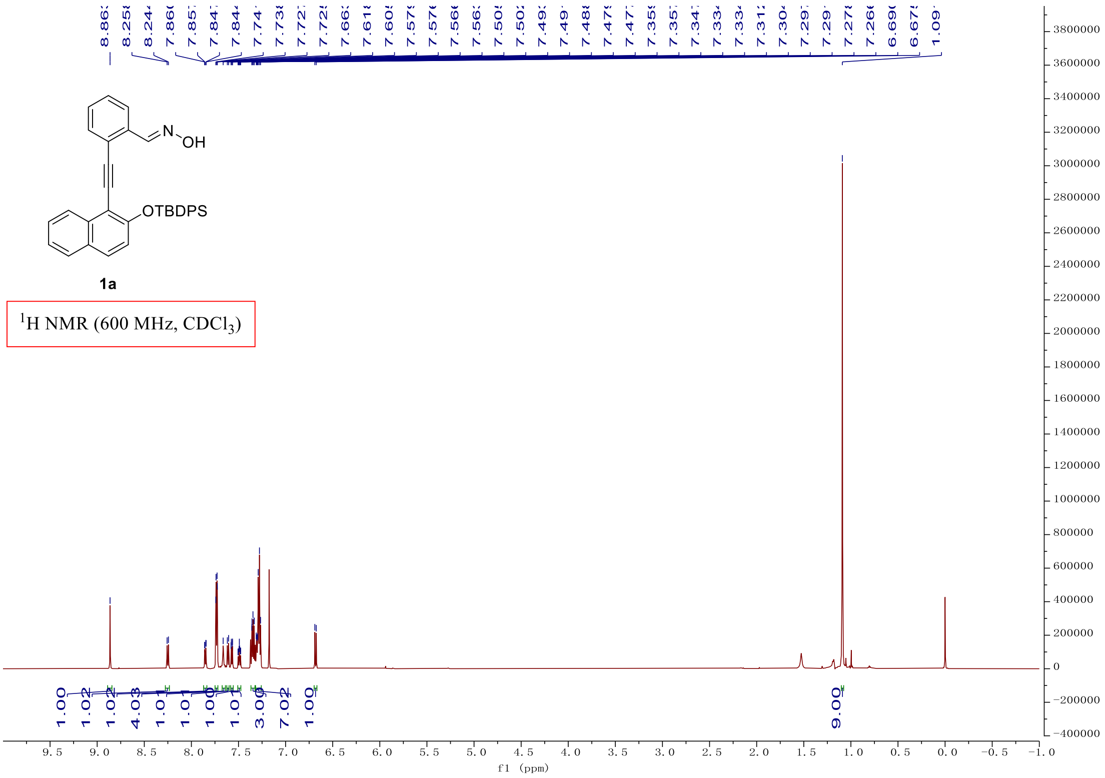

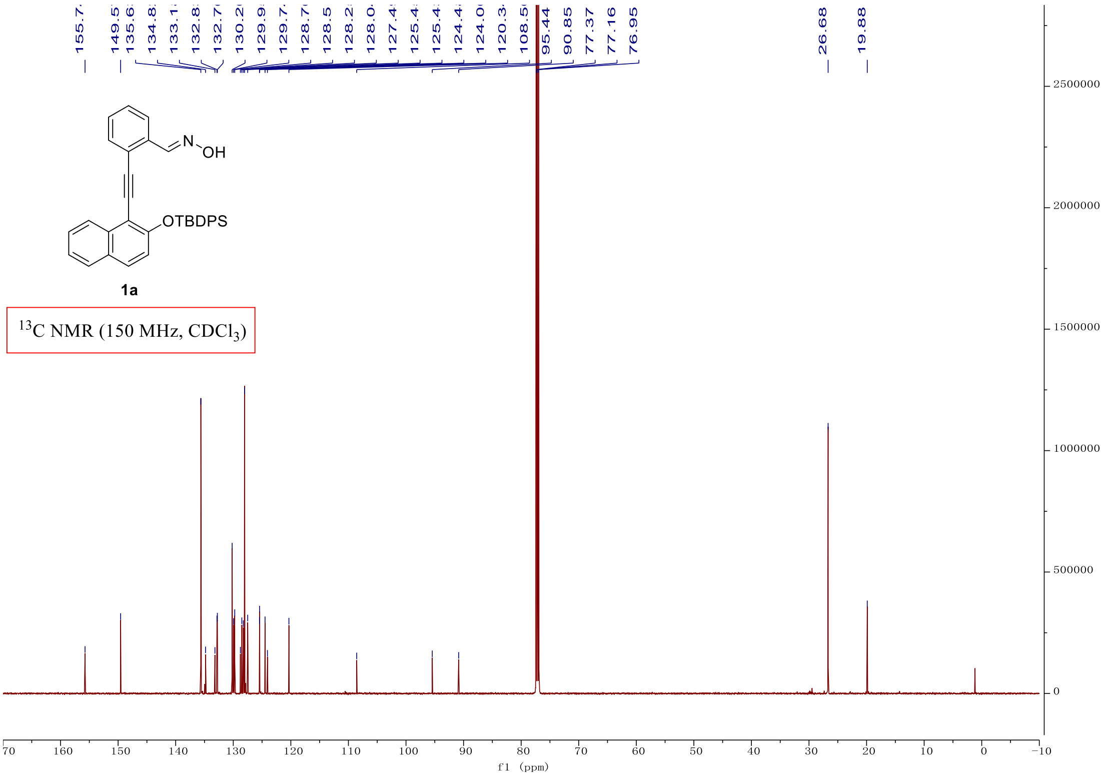

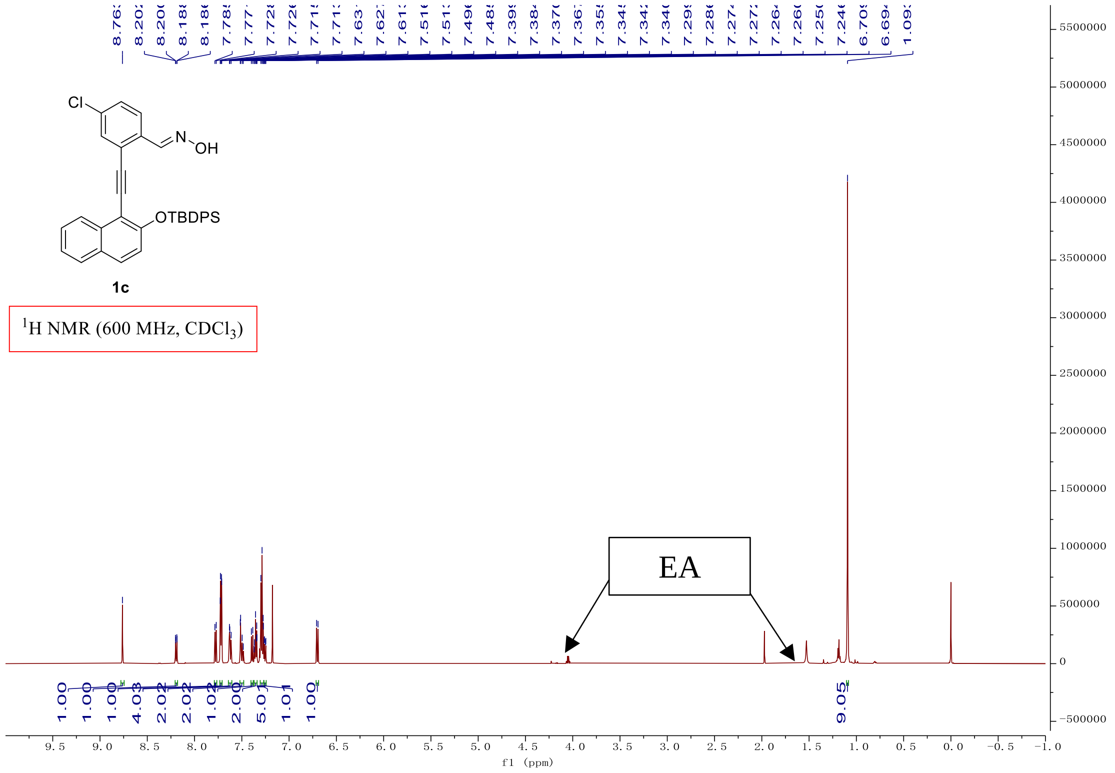

Cl
N
OH
OTBDPS
1c
1H NMR (600 MHz, CDCl3)
EA
f1 (ppm)

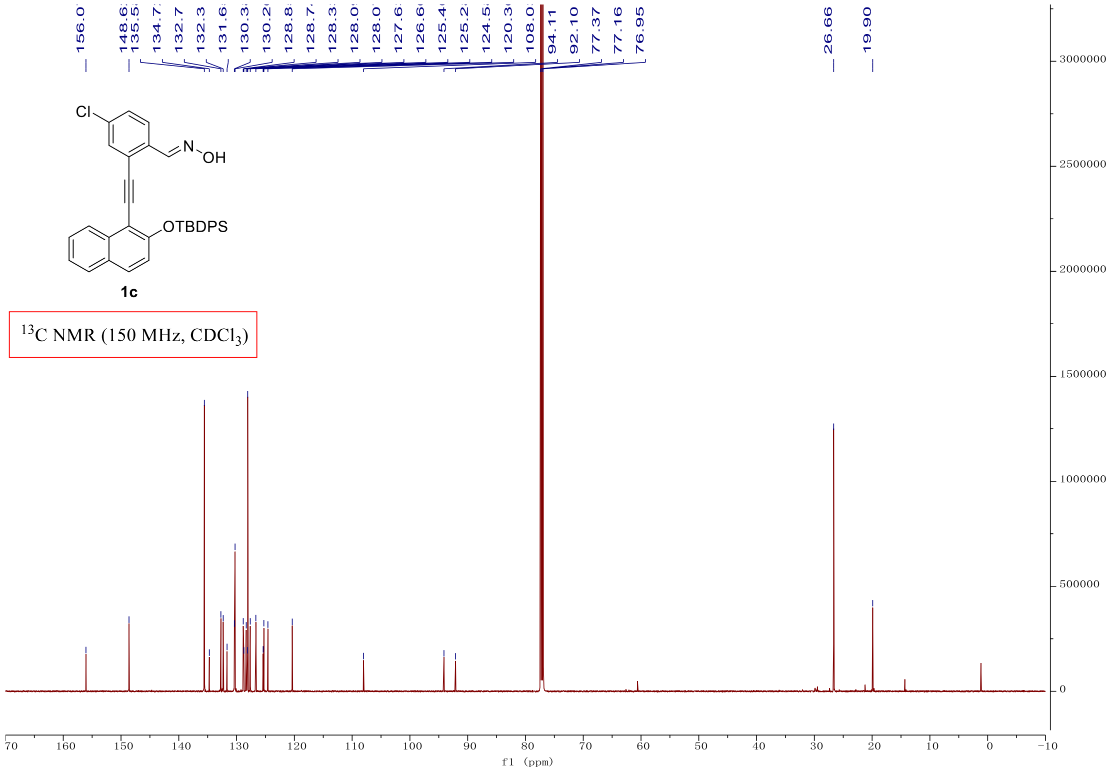

Cl
N
OH
OTBDPS
1c
13C NMR (150 MHz, CDCl3)
f1 (ppm)

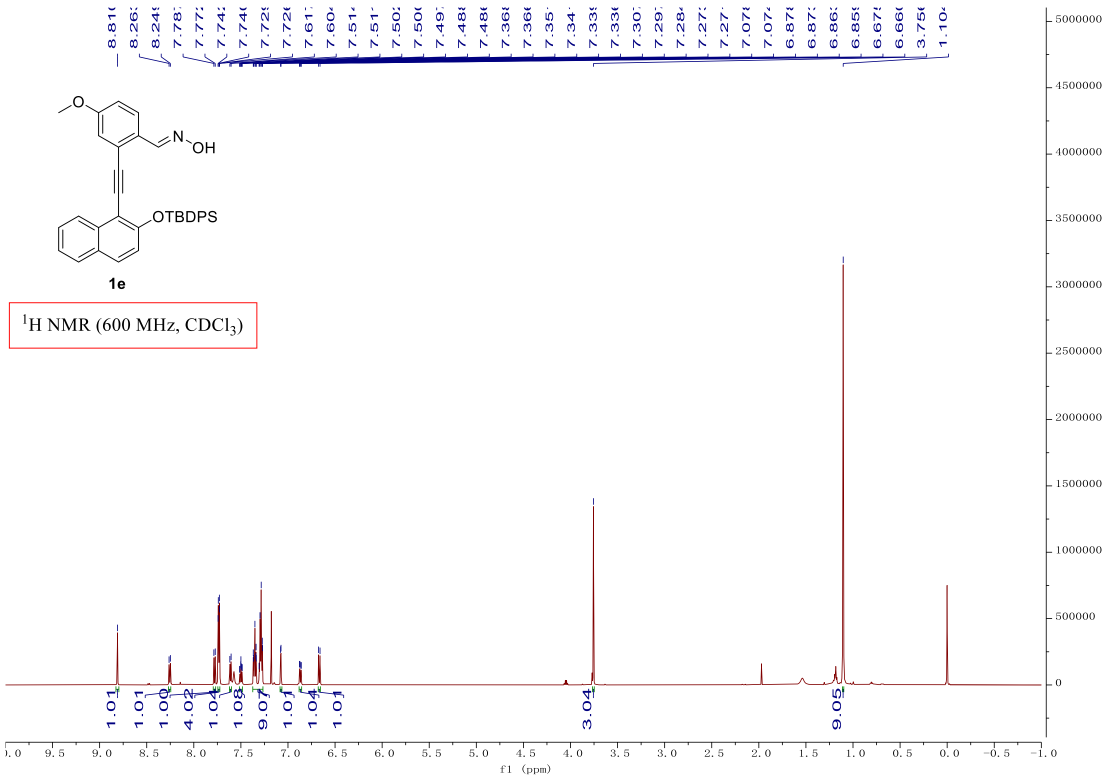
1e
^{1}H NMR (600 MHz, CDCl$_3$)
f1 (ppm)

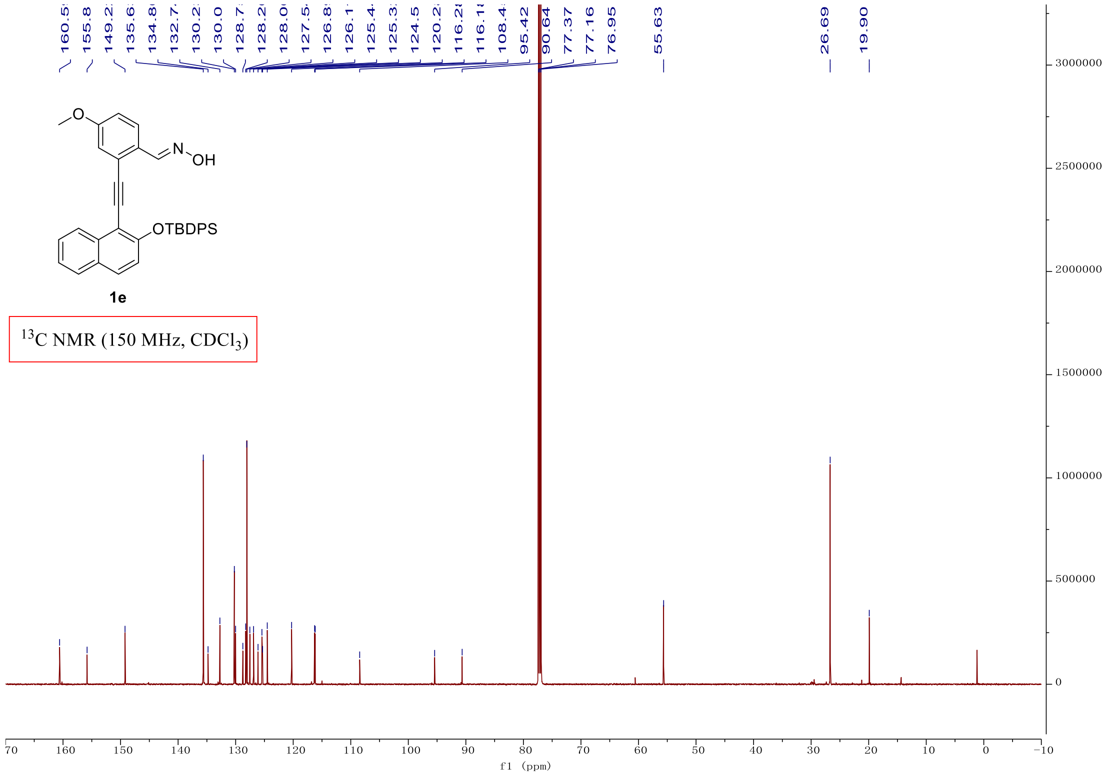
1e
^{13}C NMR (150 MHz, CDCl$_3$)
f1 (ppm)

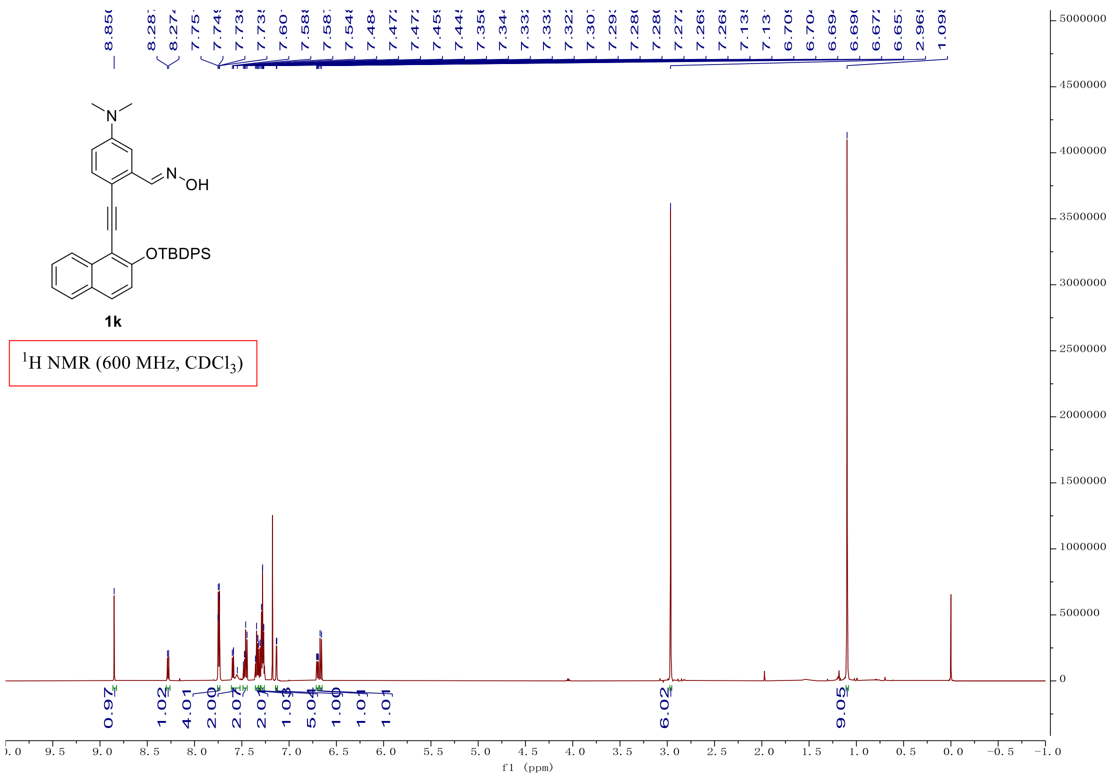

1k

^{1}H NMR (600 MHz, $CDCl_3$)

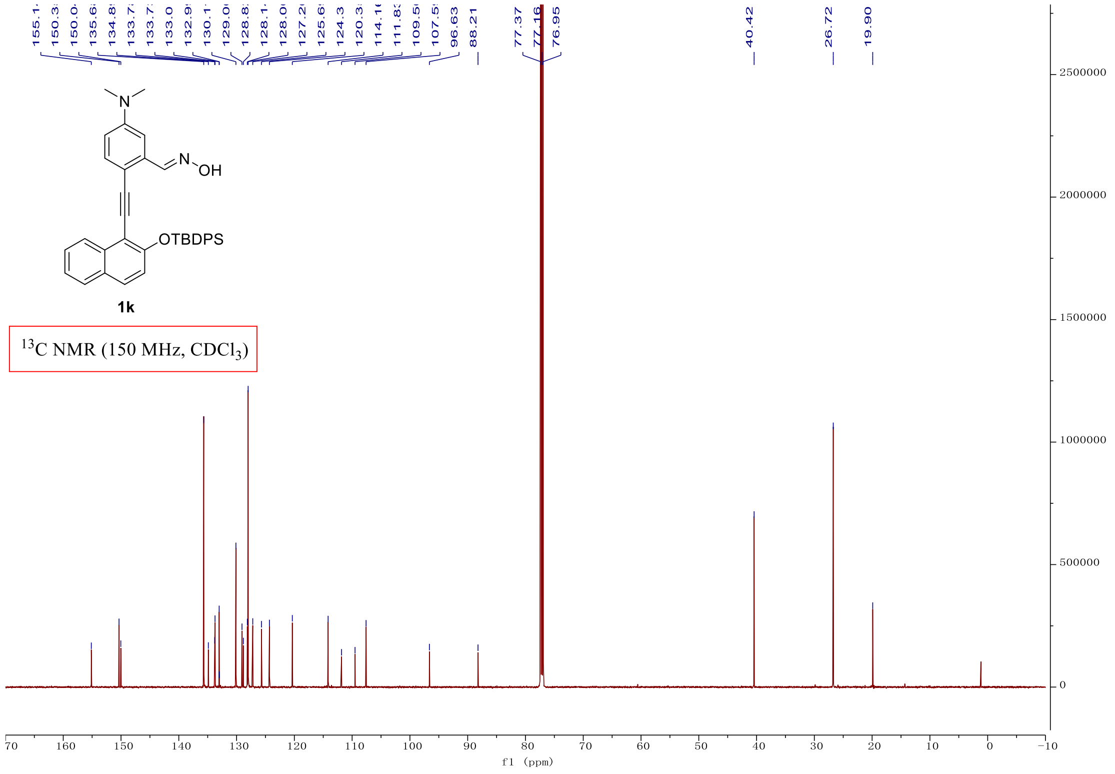

1k

^{13}C NMR (150 MHz, $CDCl_3$)

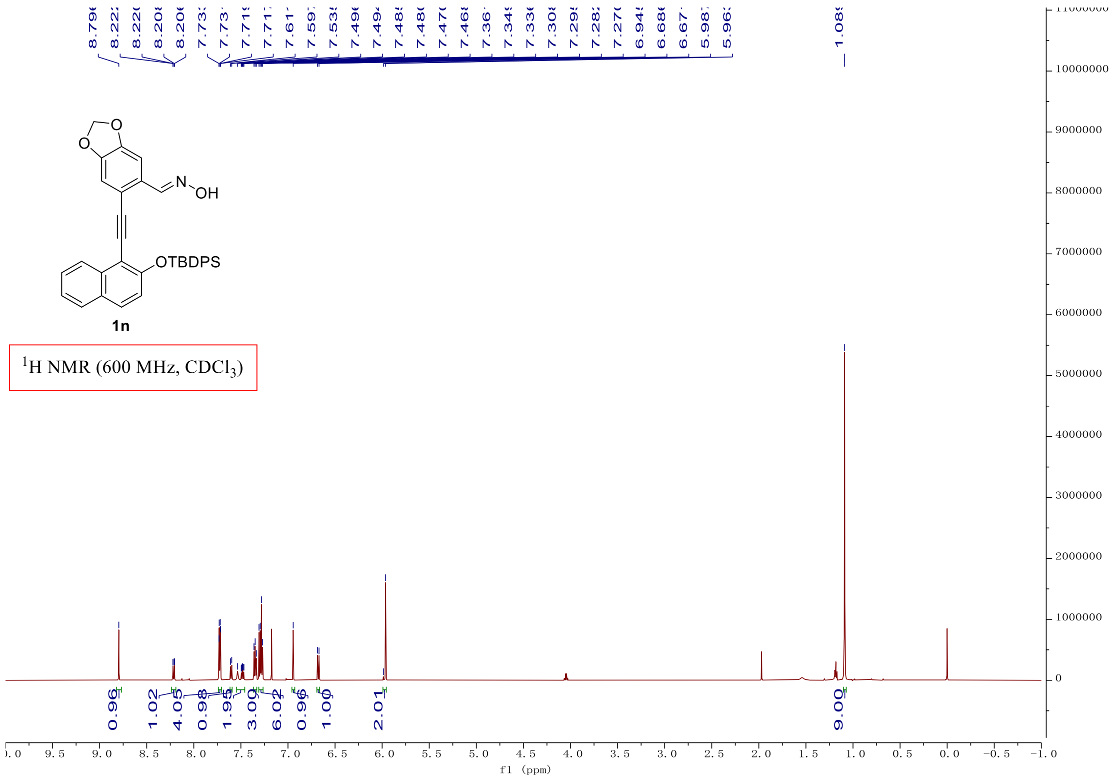
OTBDPS
OH
N
1n
^{1}H NMR (600 MHz, CDCl$_3$)
f1 (ppm)

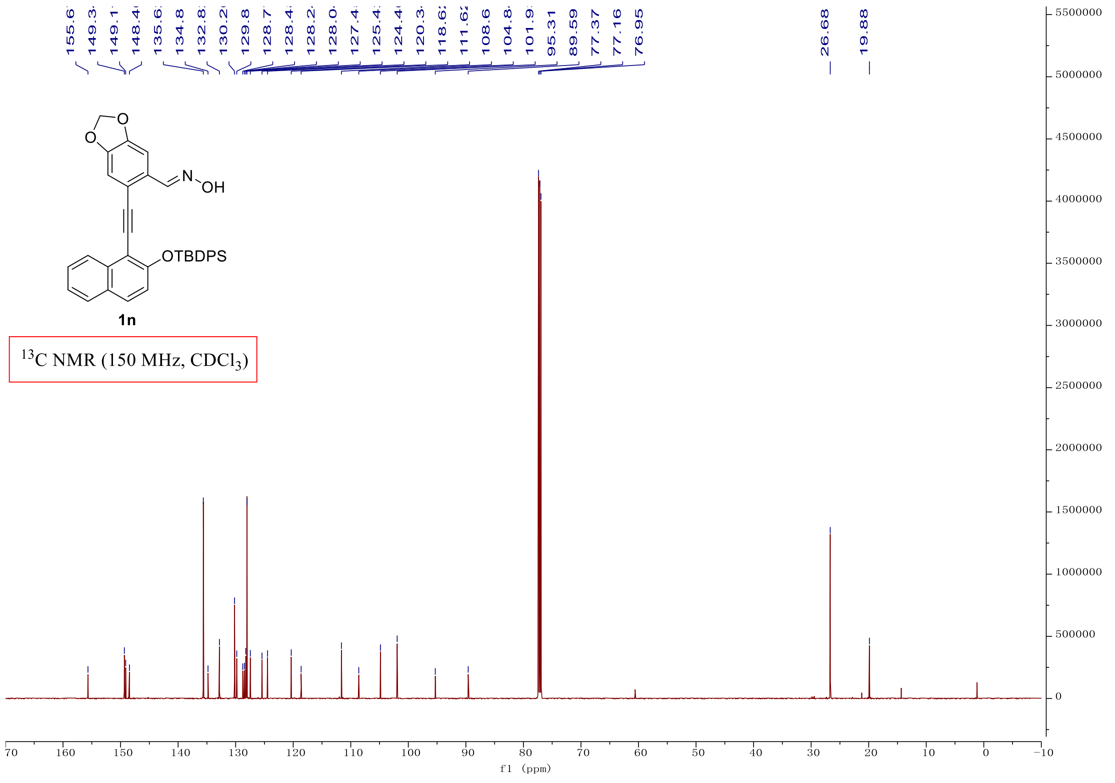
OTBDPS
OH
N
1n
^{13}C NMR (150 MHz, CDCl$_3$)
f1 (ppm)

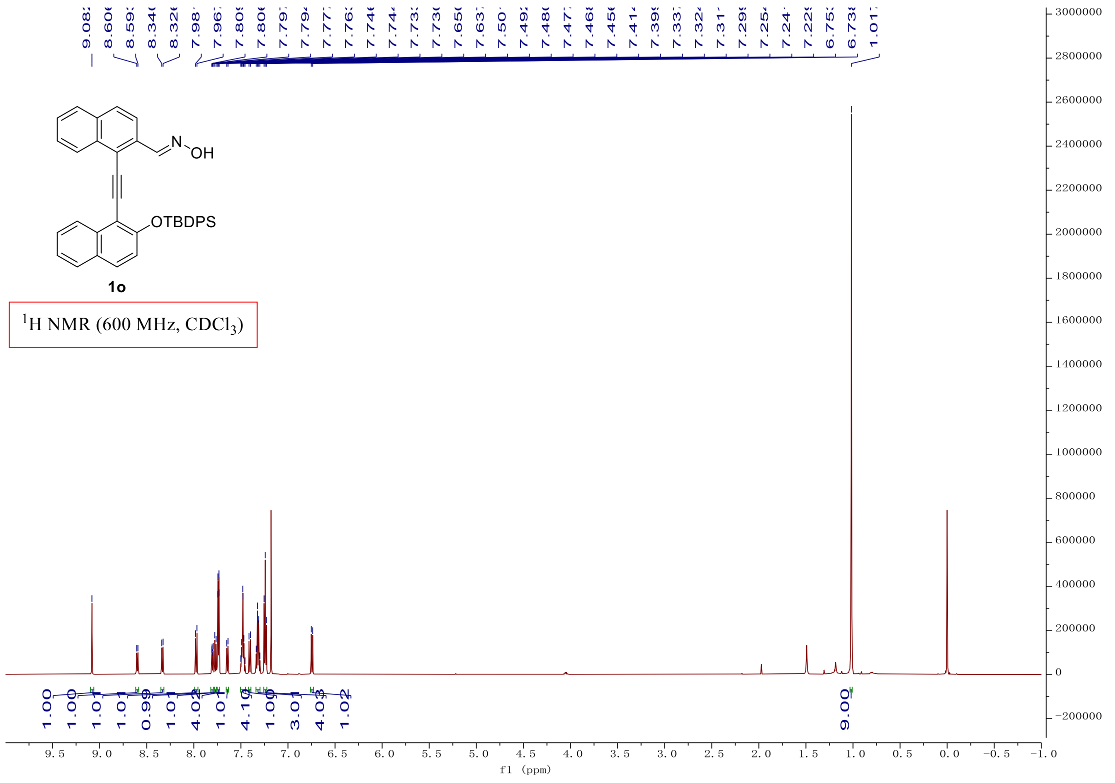

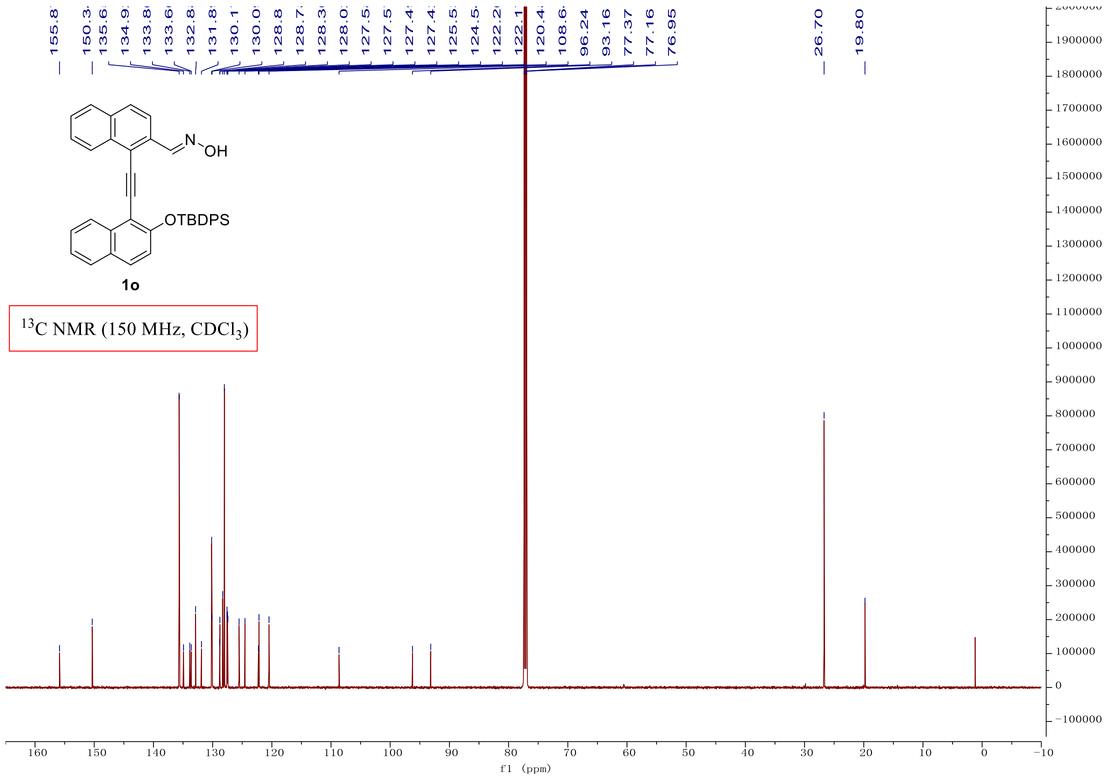

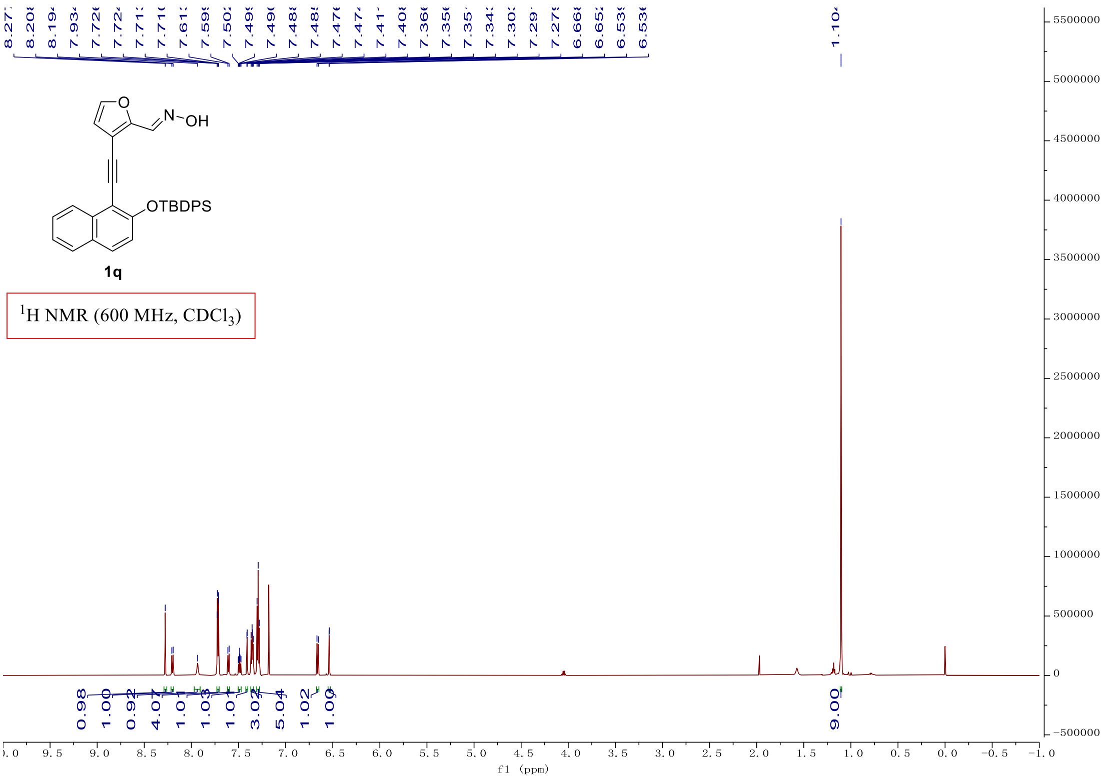
N~OH
OTBDPS
1q
^{1}H NMR (600 MHz, CDCl$_3$)
f1 (ppm)

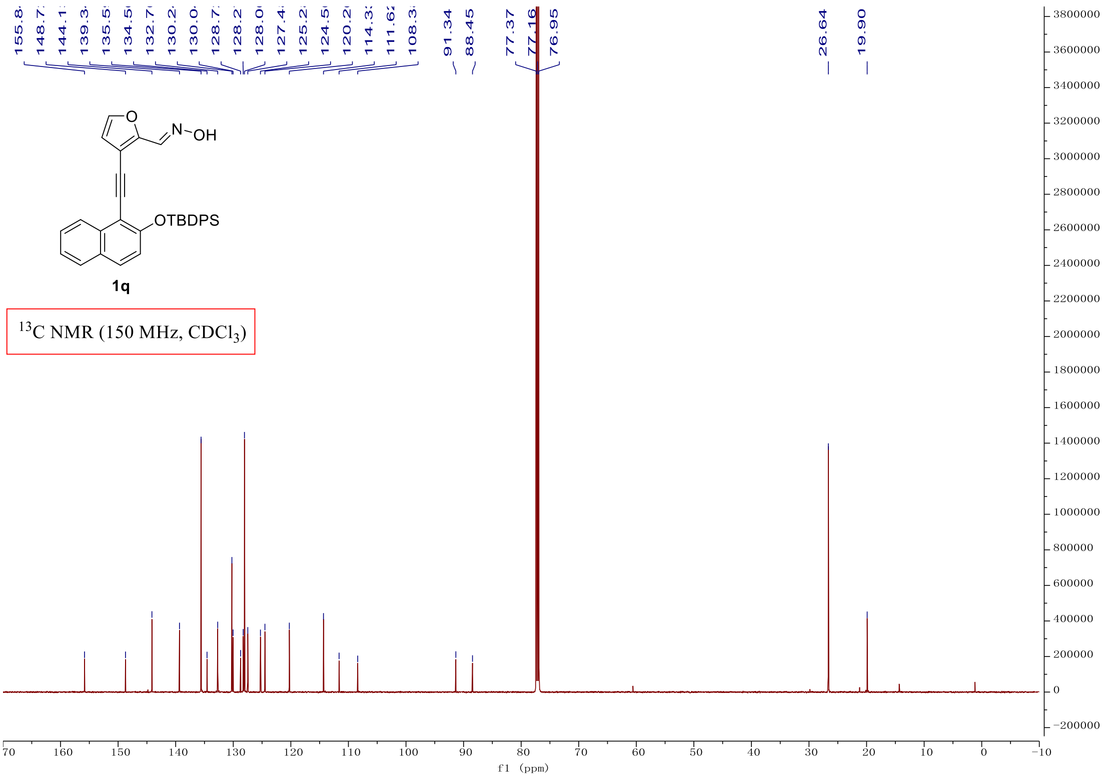
N~OH
OTBDPS
1q
^{13}C NMR (150 MHz, CDCl$_3$)
f1 (ppm)

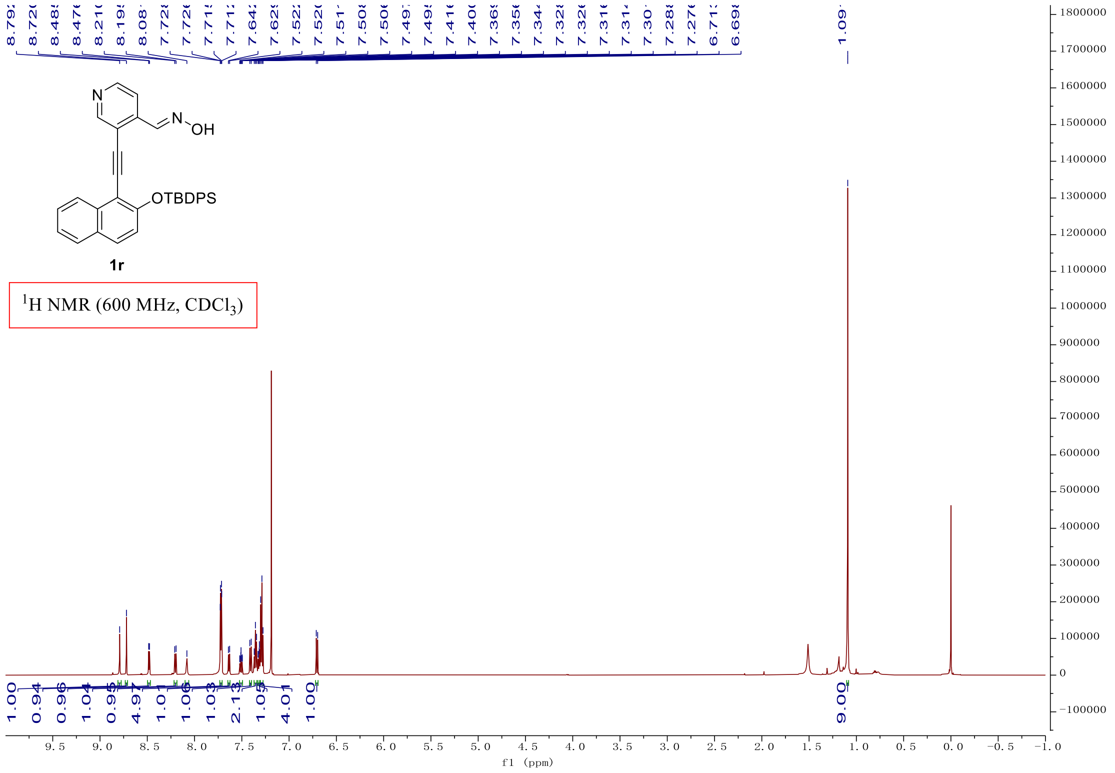
OTBDPS
OH
1r
1H NMR (600 MHz, CDCl3)
f1 (ppm)

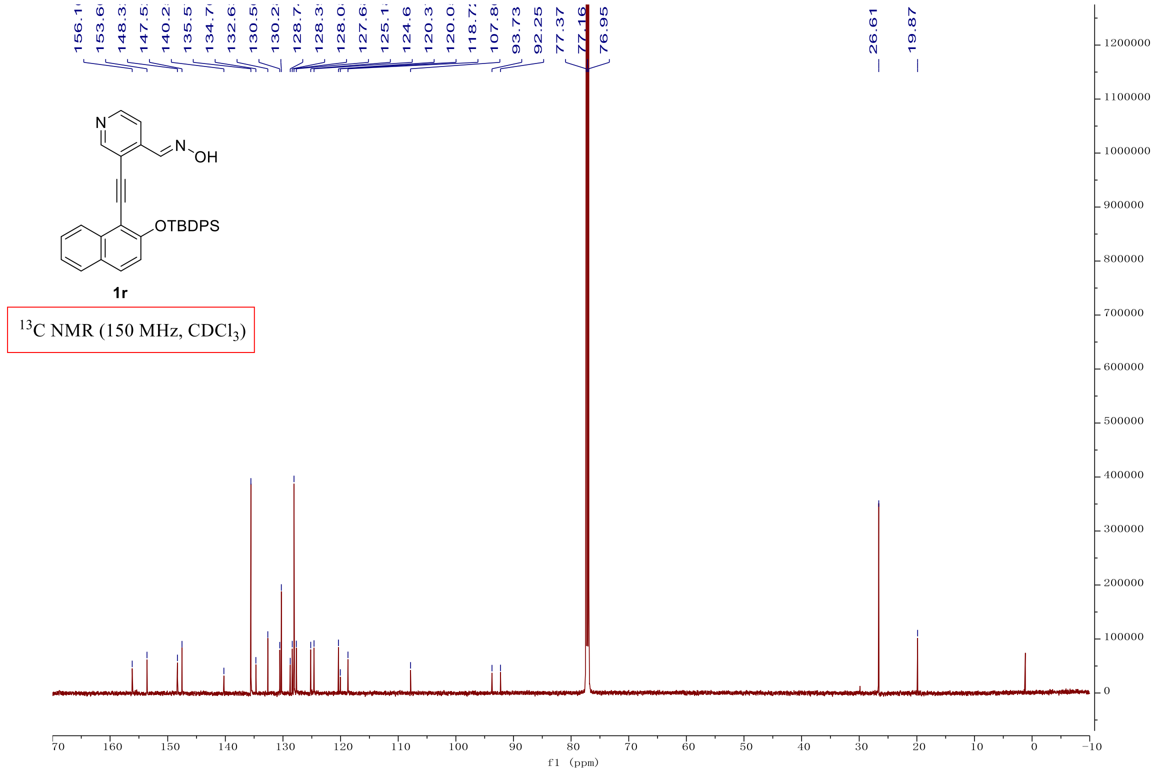
OTBDPS
OH
1r
13C NMR (150 MHz, CDCl3)
f1 (ppm)

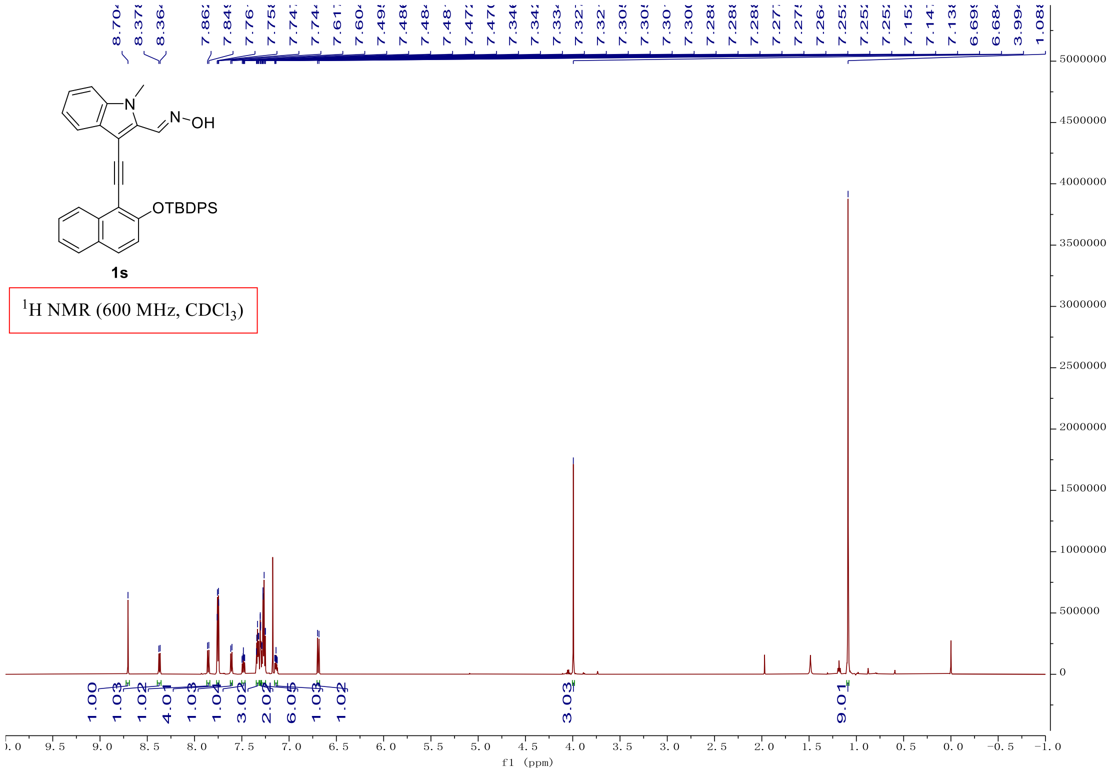
N
N
OH
OTBDPS
1s
¹H NMR (600 MHz, CDCl₃)
f1 (ppm)

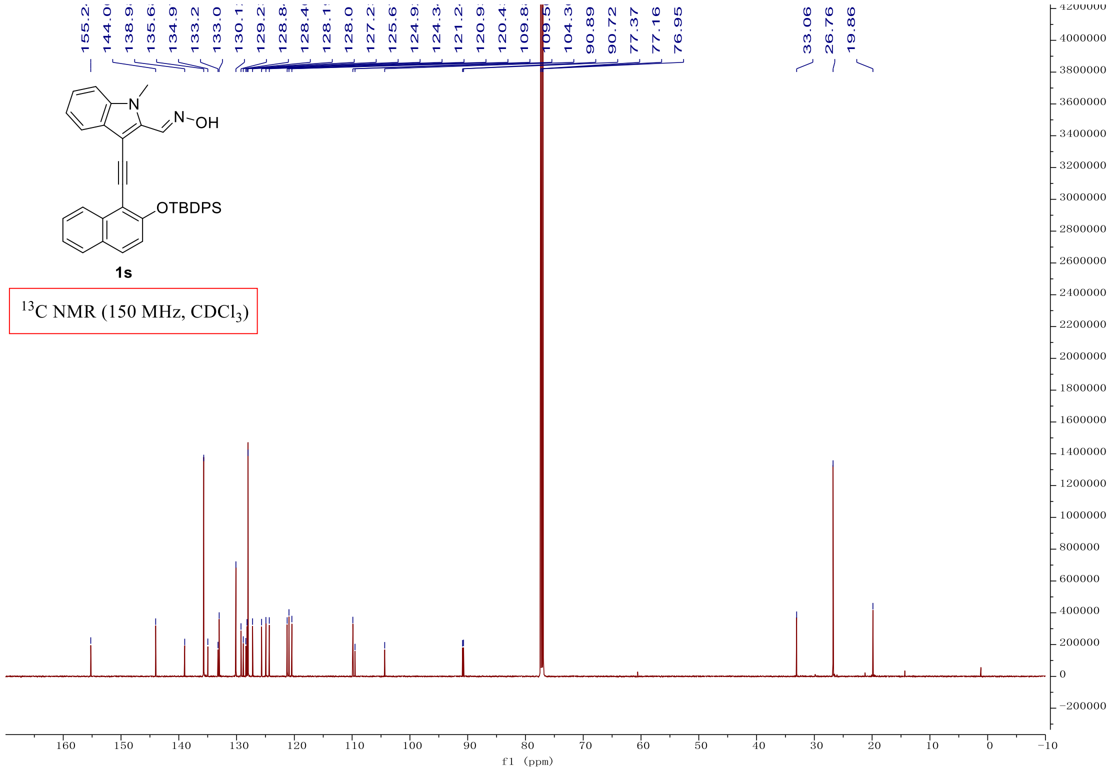
N
N
OH
OTBDPS
1s
¹³C NMR (150 MHz, CDCl₃)
f1 (ppm)

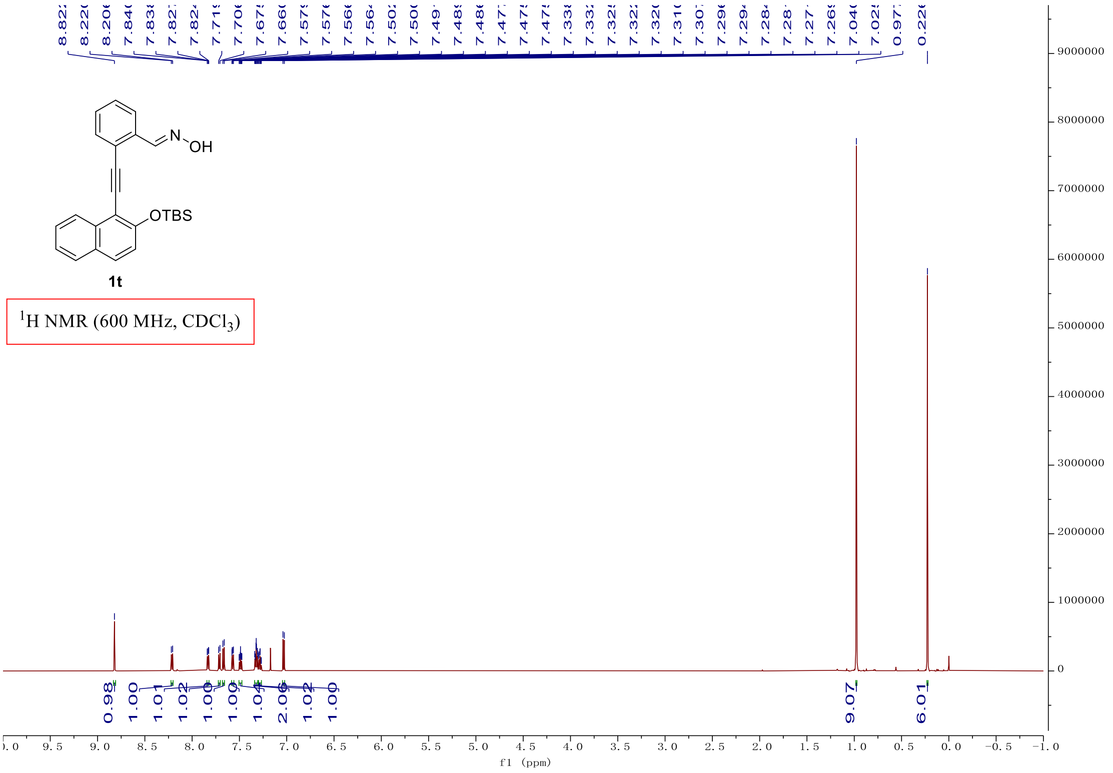

1t

^{1}H NMR (600 MHz, $CDCl_3$)

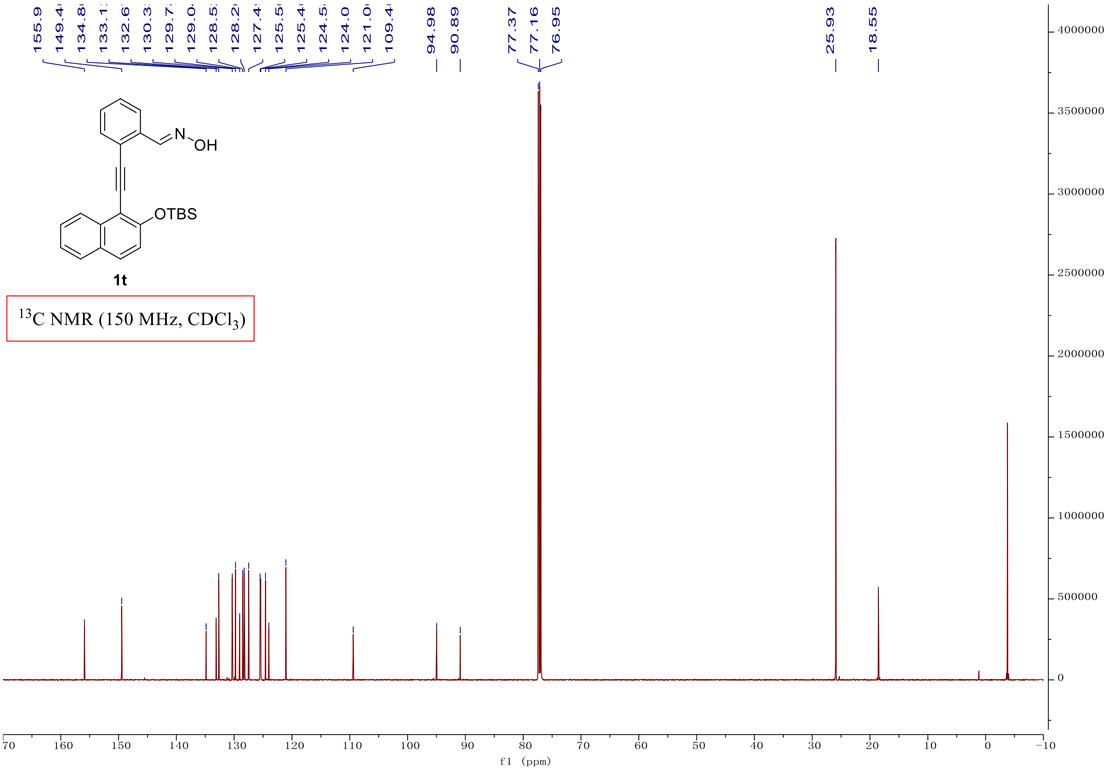

1t

^{13}C NMR (150 MHz, $CDCl_3$)

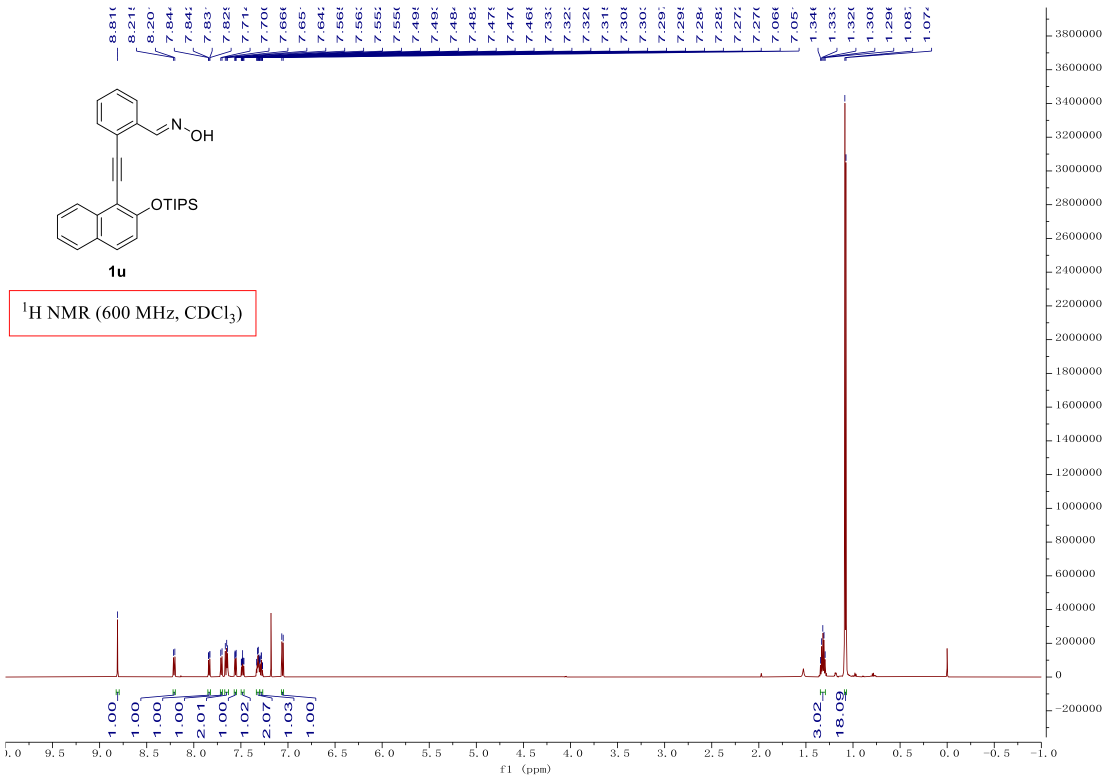

OTIPS
N
OH
1u
1H NMR (600 MHz, CDCl3)
f1 (ppm)

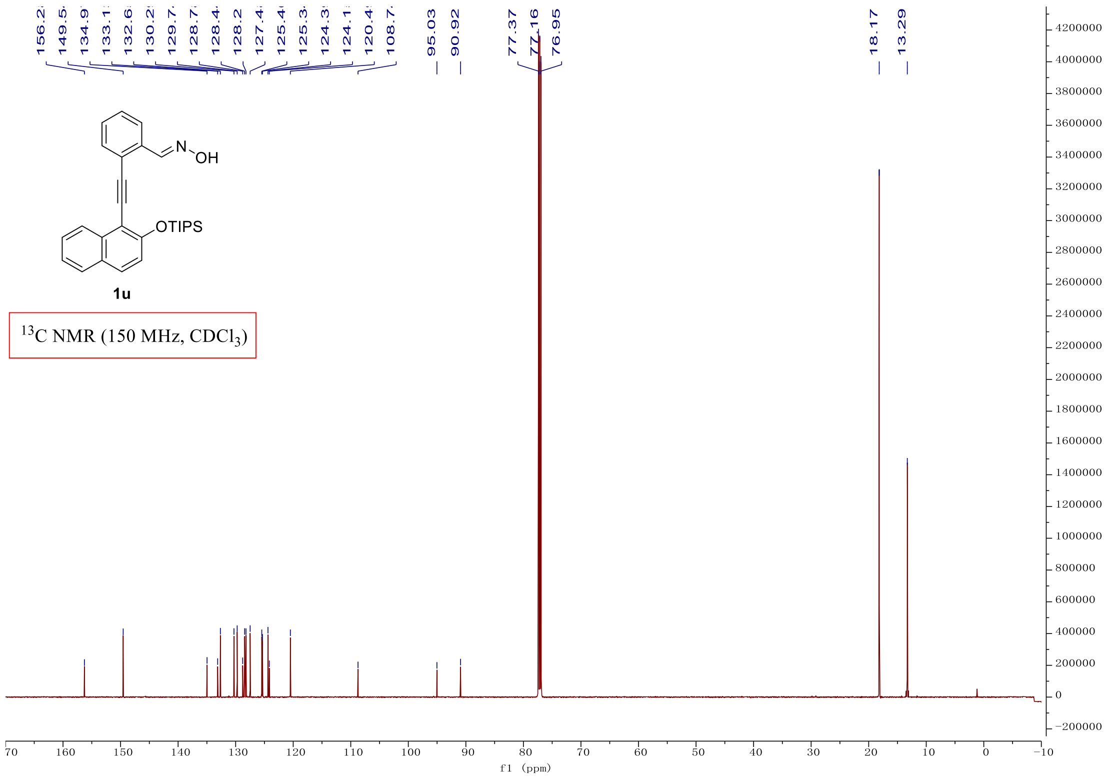

OTIPS
N
OH
1u
13C NMR (150 MHz, CDCl3)
95.03
90.92
77.37
77.16
76.95
18.17
13.29
f1 (ppm)

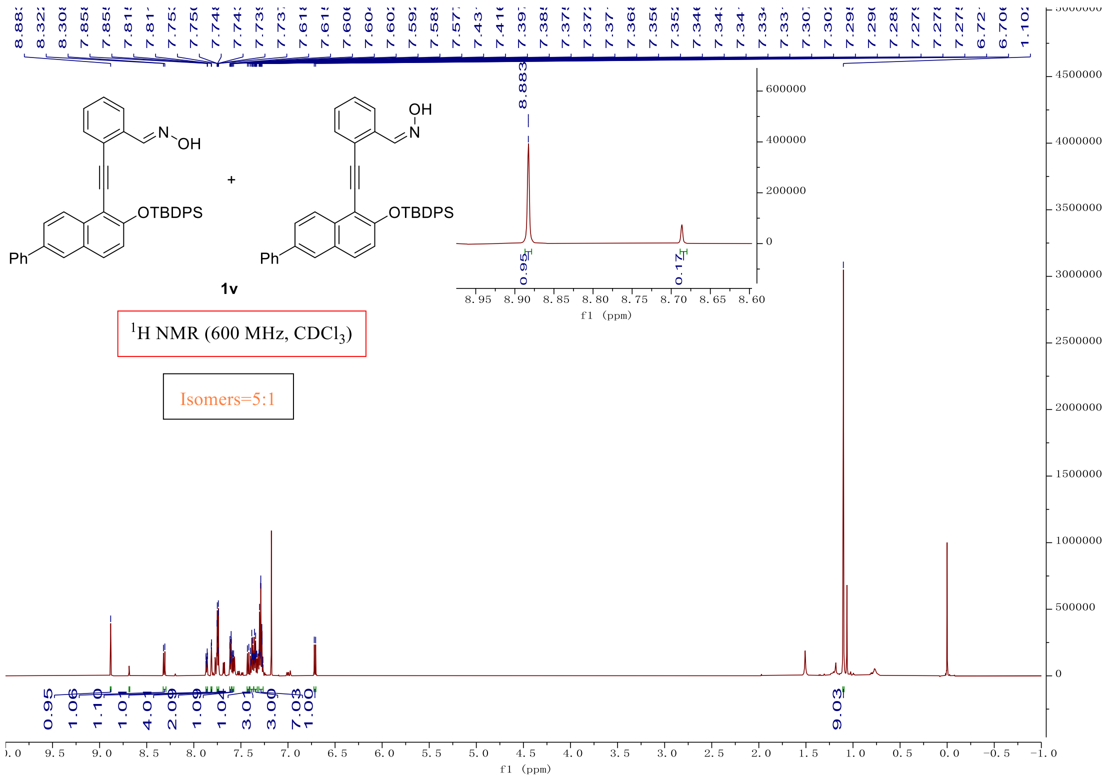

1v
^{1}H NMR (600 MHz, CDCl$_3$)
Isomers=5:1
f1 (ppm)

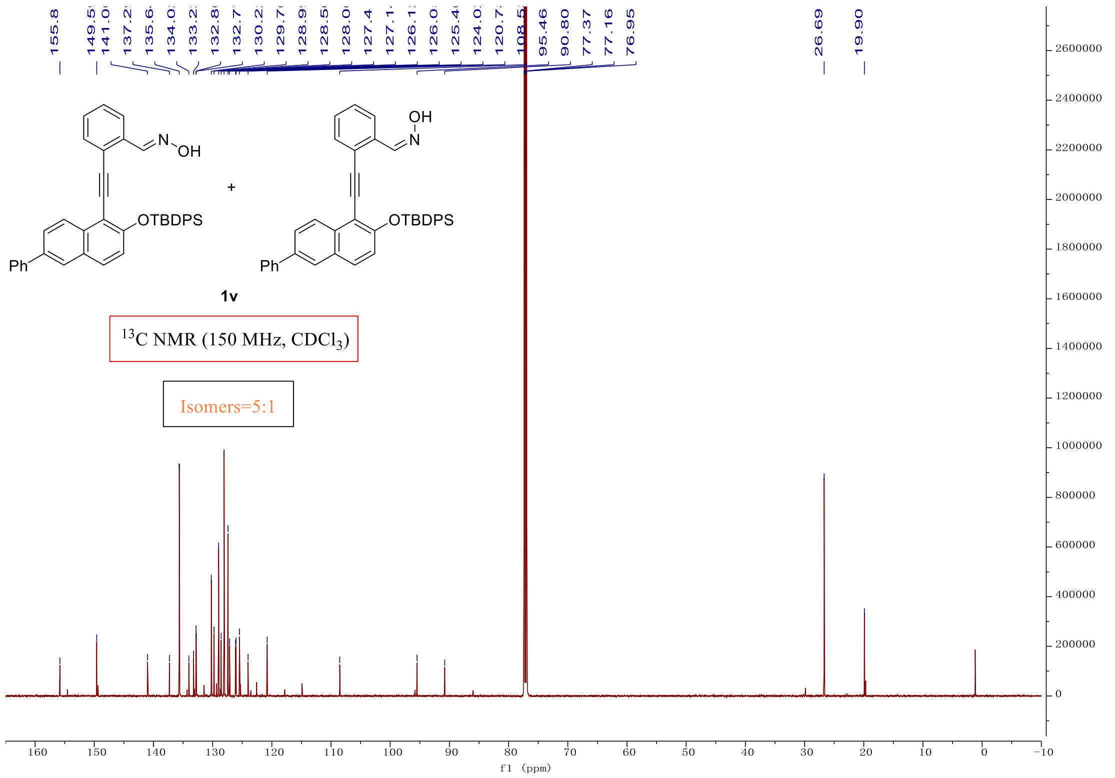

1v
^{13}C NMR (150 MHz, CDCl$_3$)
Isomers=5:1
f1 (ppm)

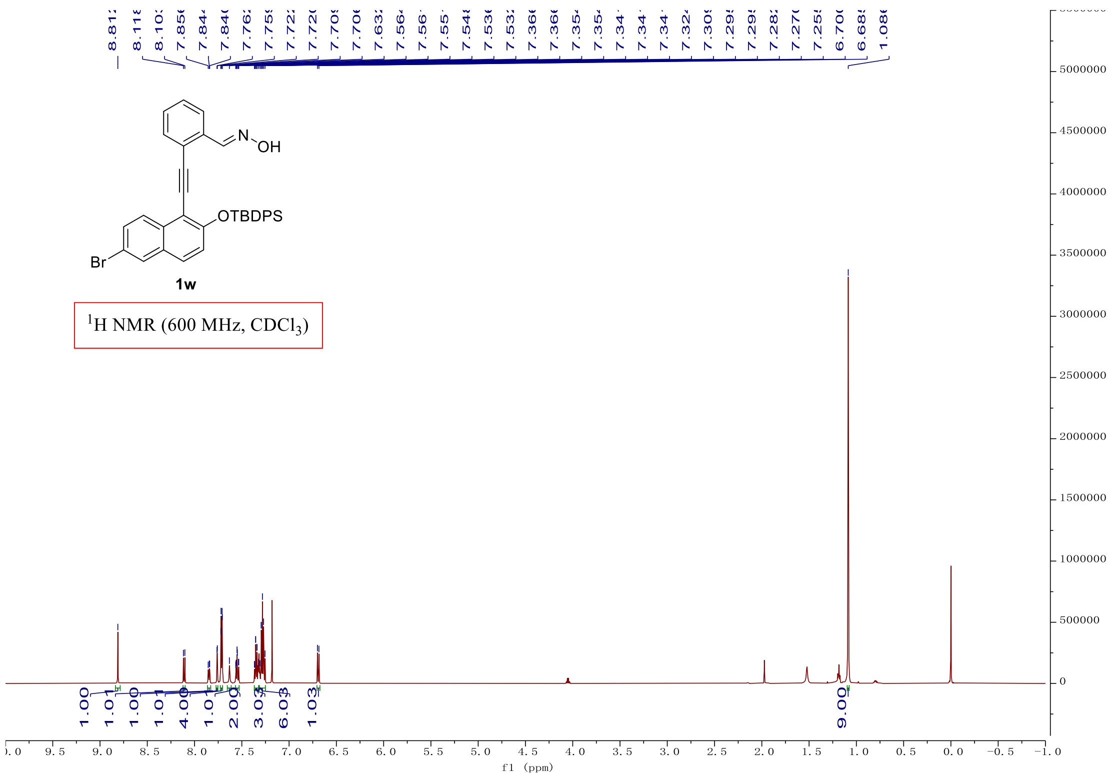
OTBDPS
Br
N
OH
1w
1H NMR (600 MHz, CDCl3)
f1 (ppm)

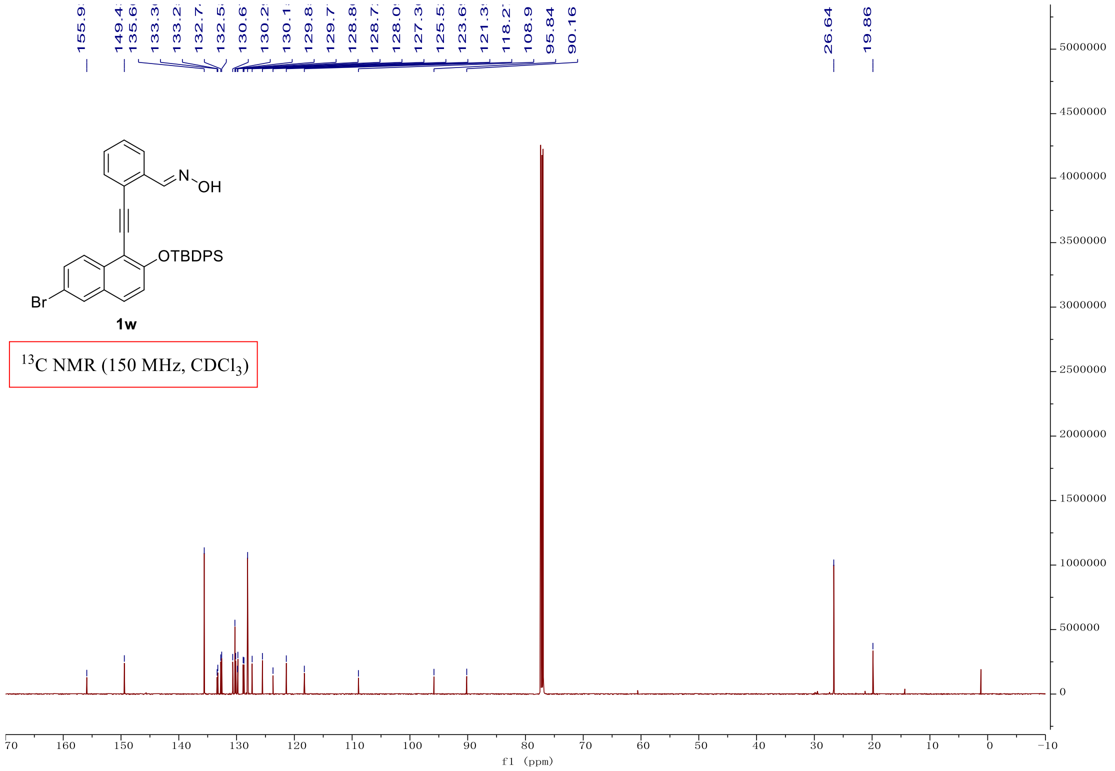
OTBDPS
Br
N
OH
1w
13C NMR (150 MHz, CDCl3)
f1 (ppm)

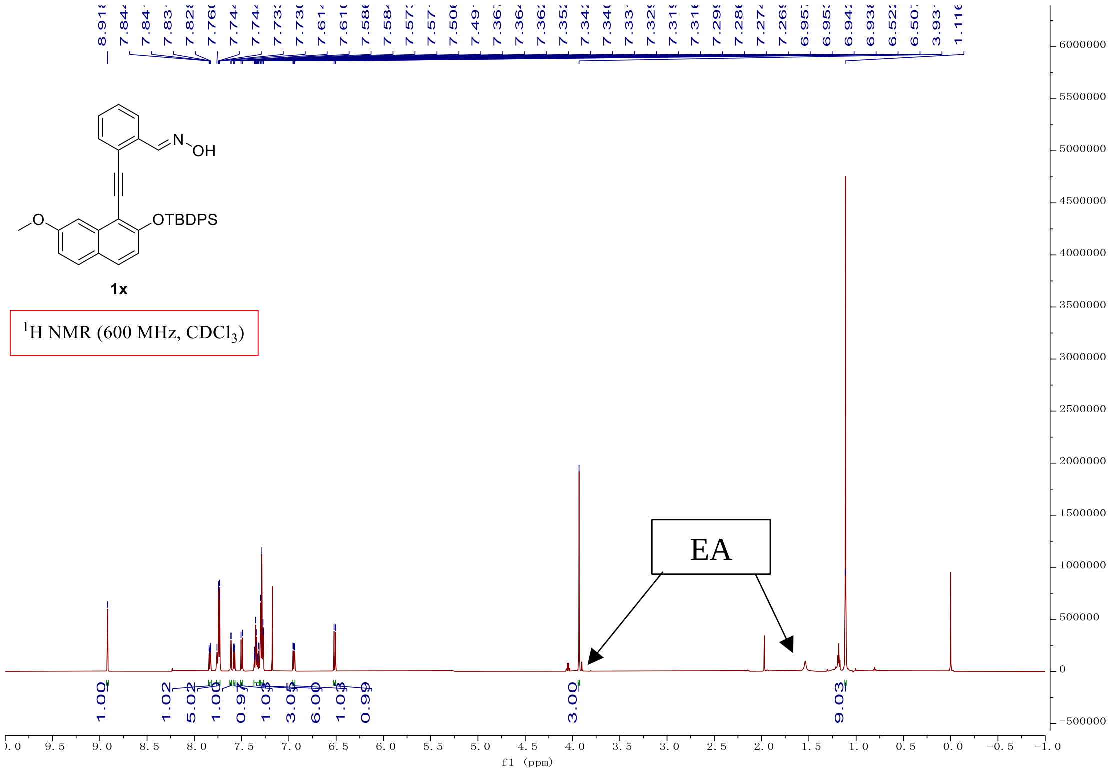

1x
^{1}H NMR (600 MHz, $CDCl_3$)
EA
f1 (ppm)

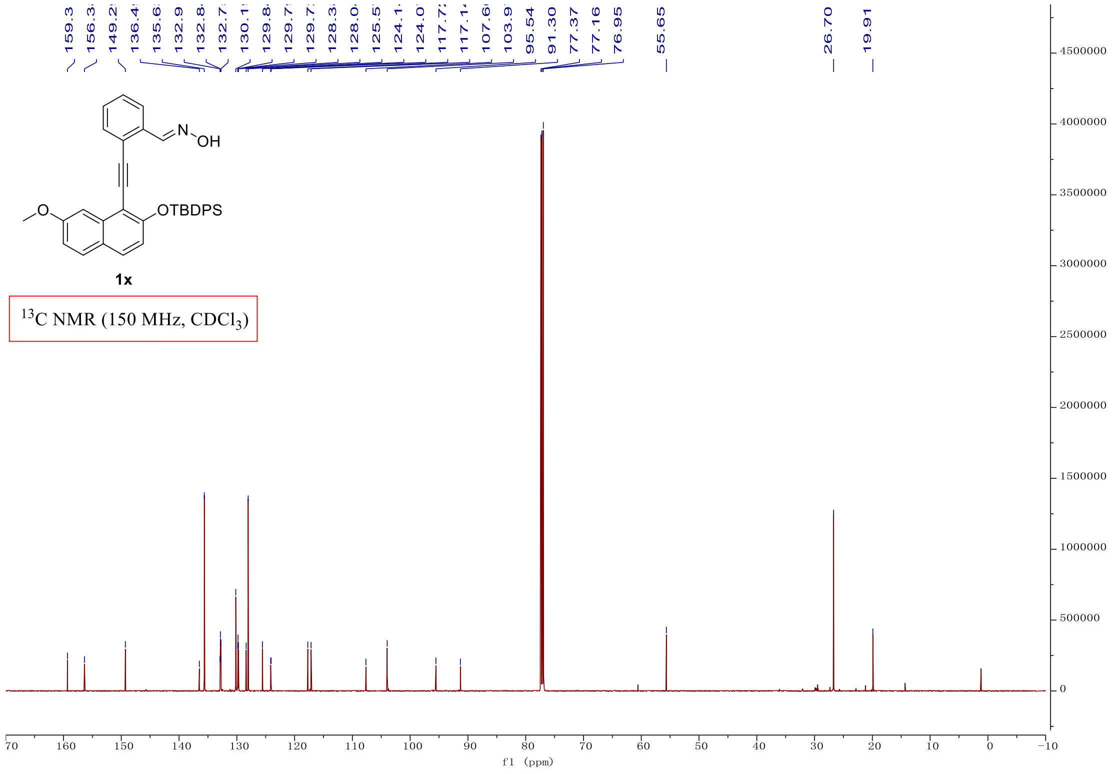

1x
^{13}C NMR (150 MHz, $CDCl_3$)
f1 (ppm)

1aa

^{1}H NMR (600 MHz, $CDCl_3$)

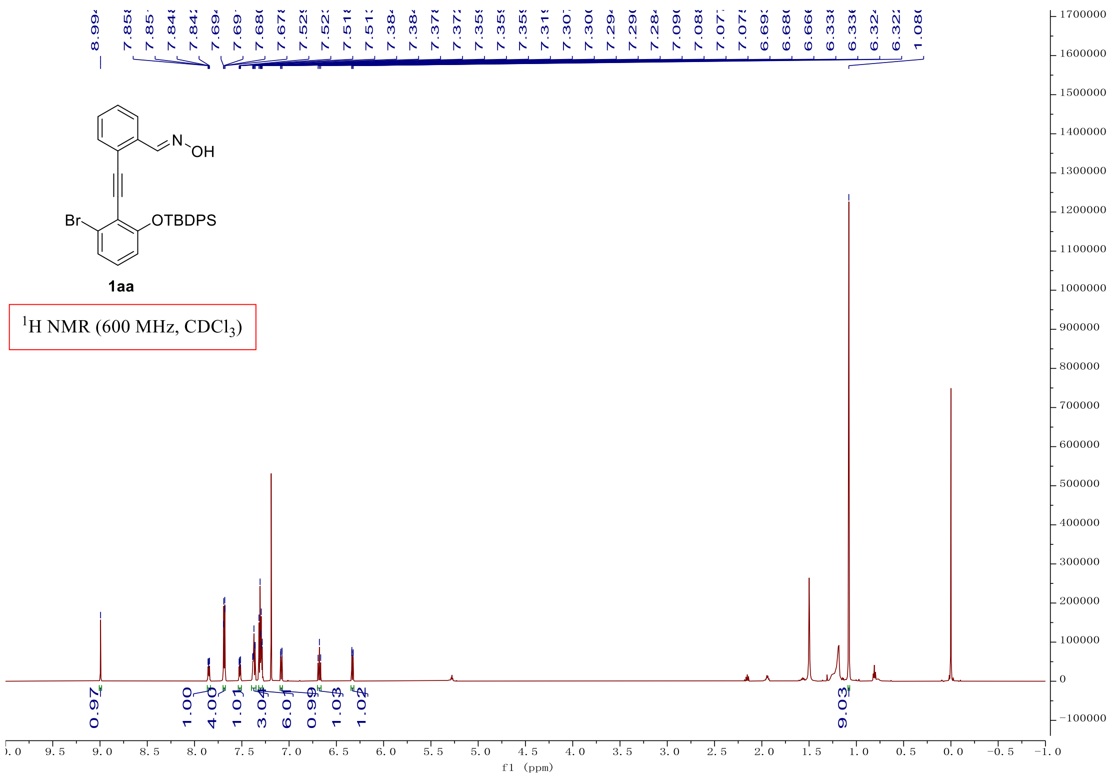

1aa

^{13}C NMR (150 MHz, $CDCl_3$)

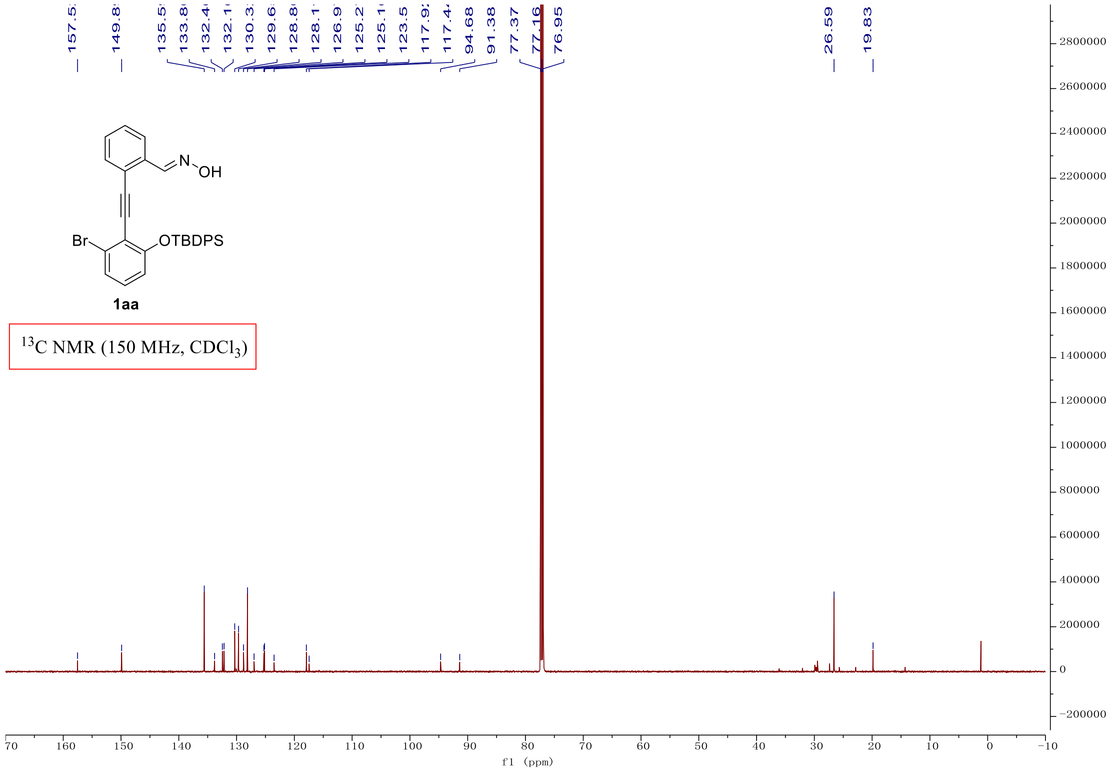

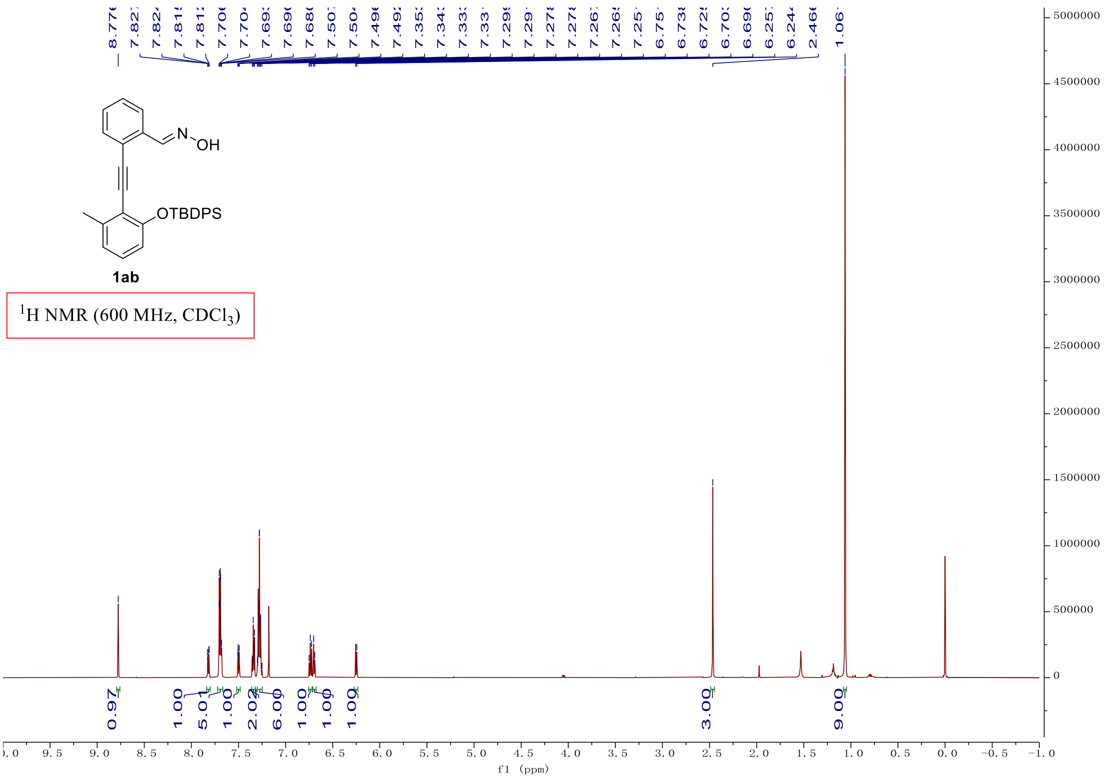

1ab
¹H NMR (600 MHz, CDCl3)
f1 (ppm)

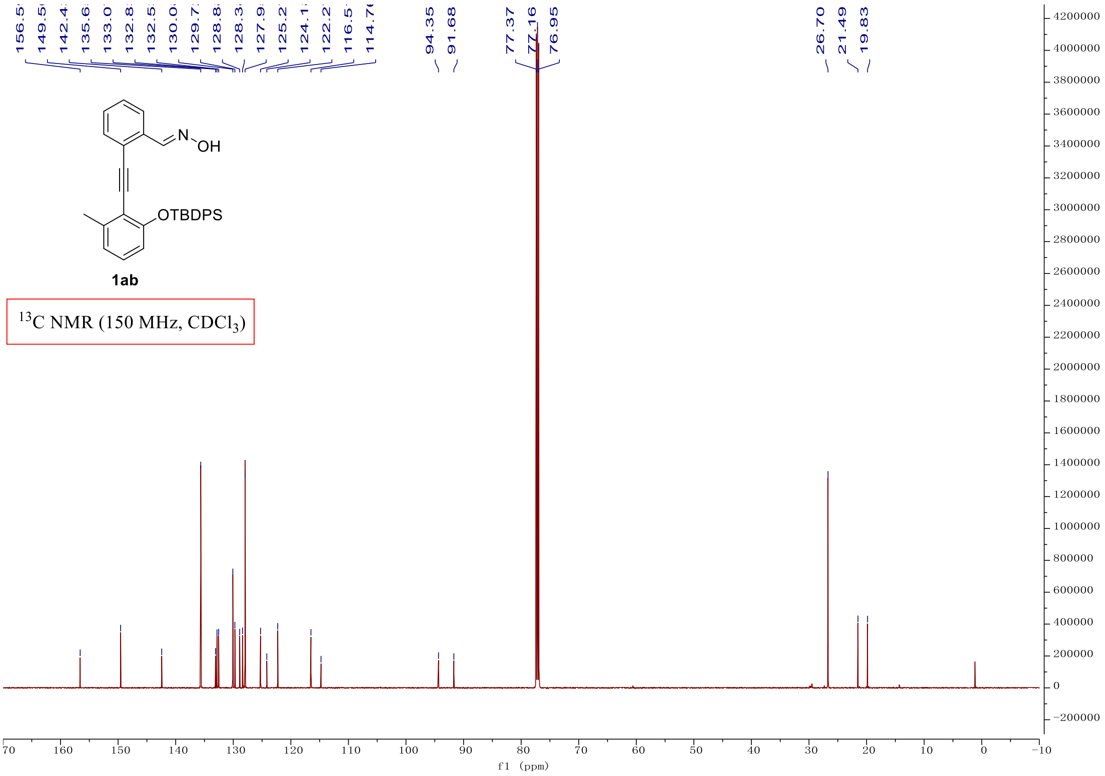

1ab
¹³C NMR (150 MHz, CDCl3)
f1 (ppm)

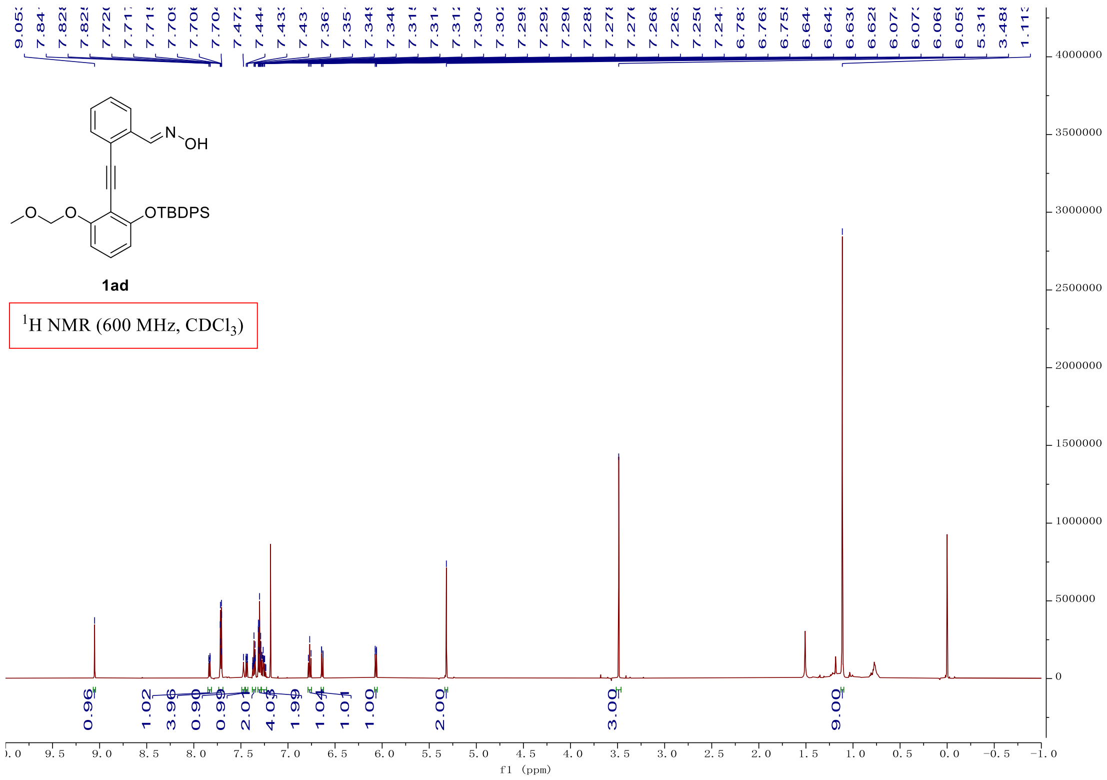

1ad
1H NMR (600 MHz, CDCl3)
OTBDPS
f1 (ppm)

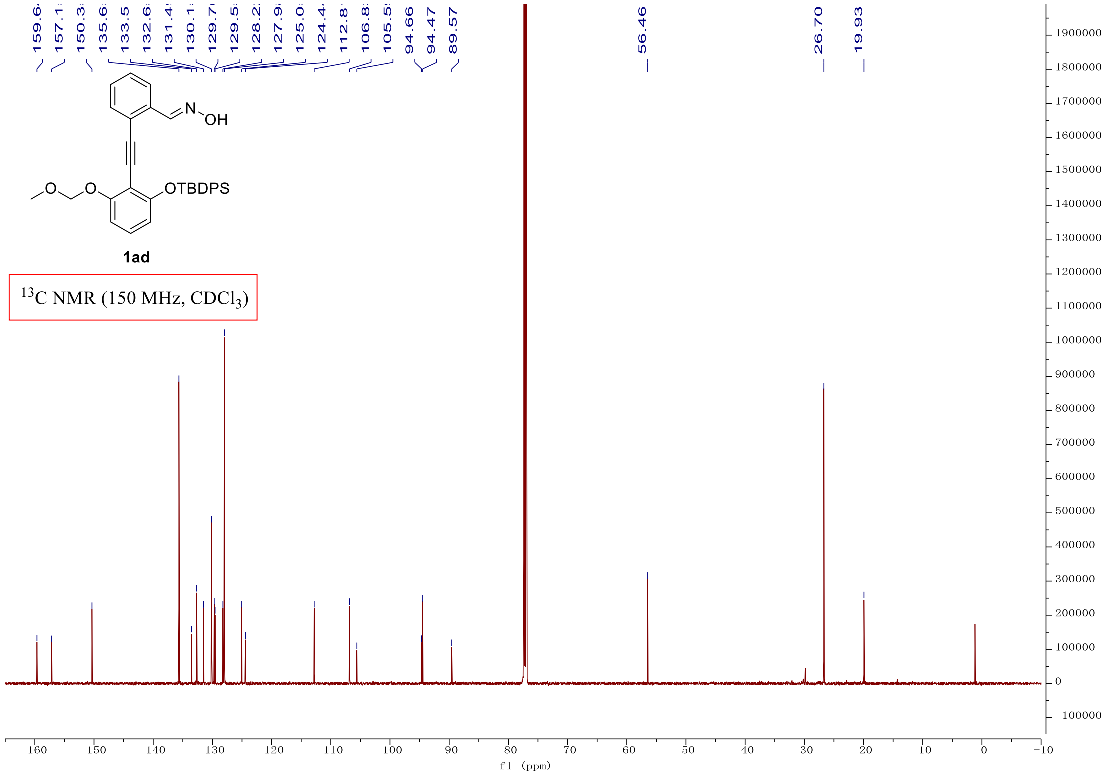

1ad
13C NMR (150 MHz, CDCl3)
OTBDPS
f1 (ppm)

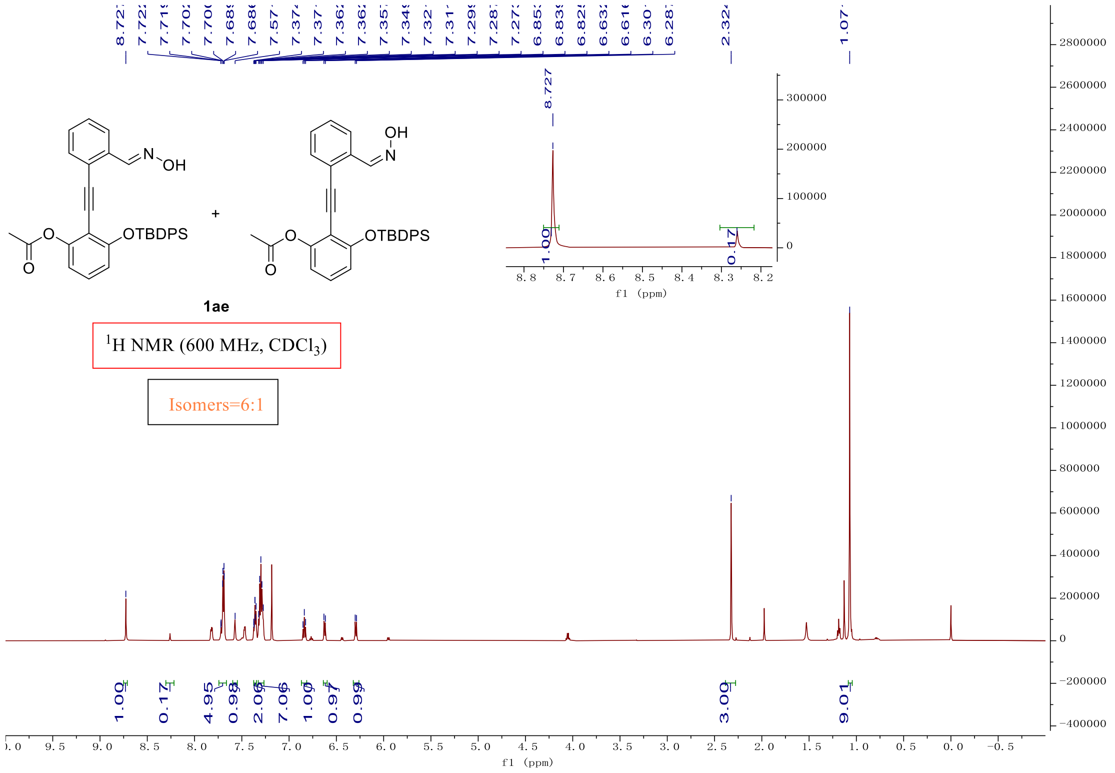

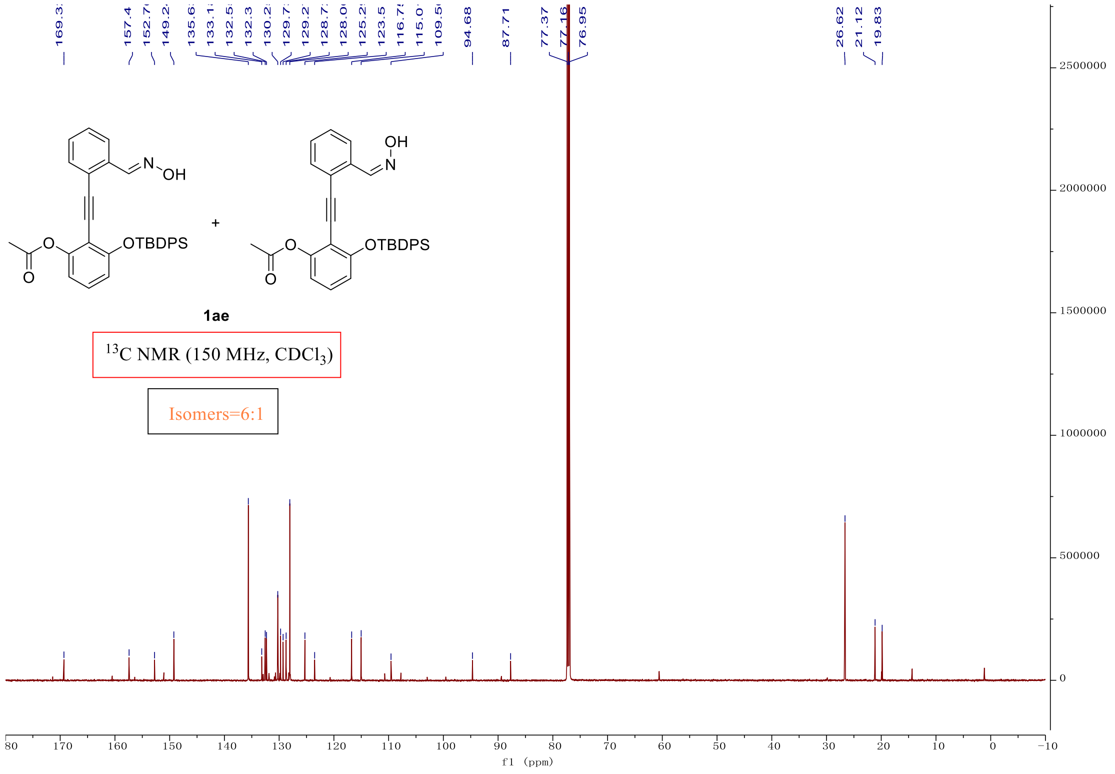

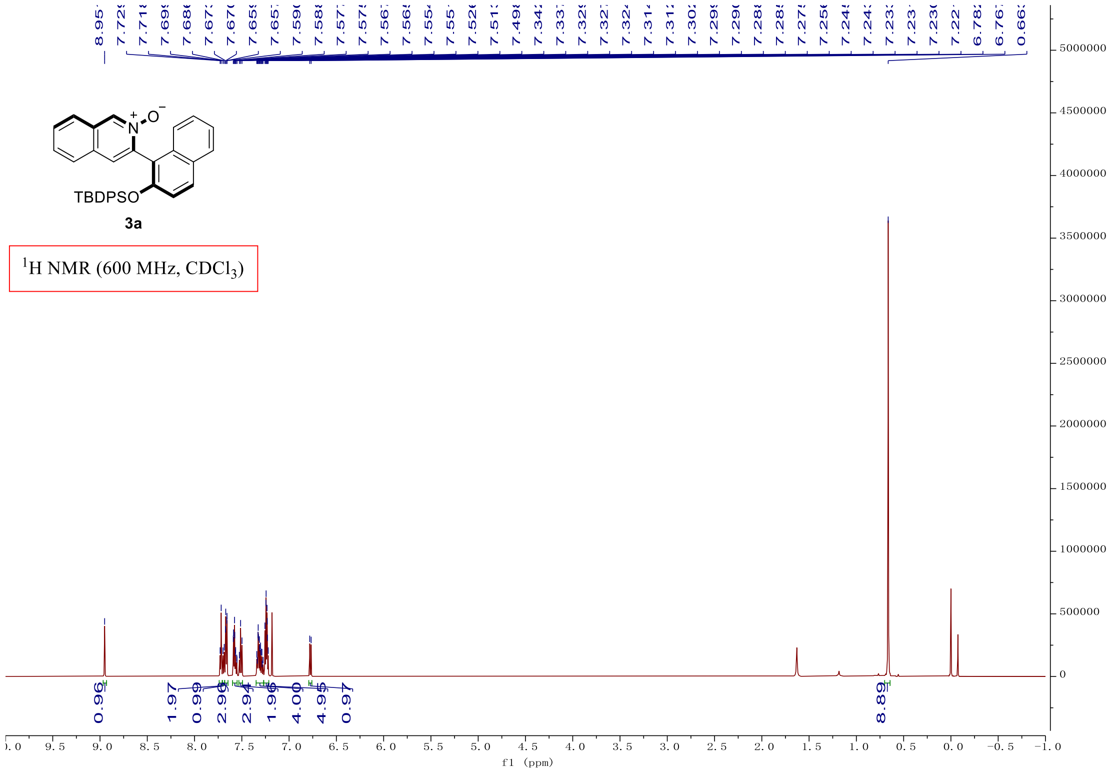
TBDPSO
3a
^{1}H NMR (600 MHz, $CDCl_3$)
f1 (ppm)

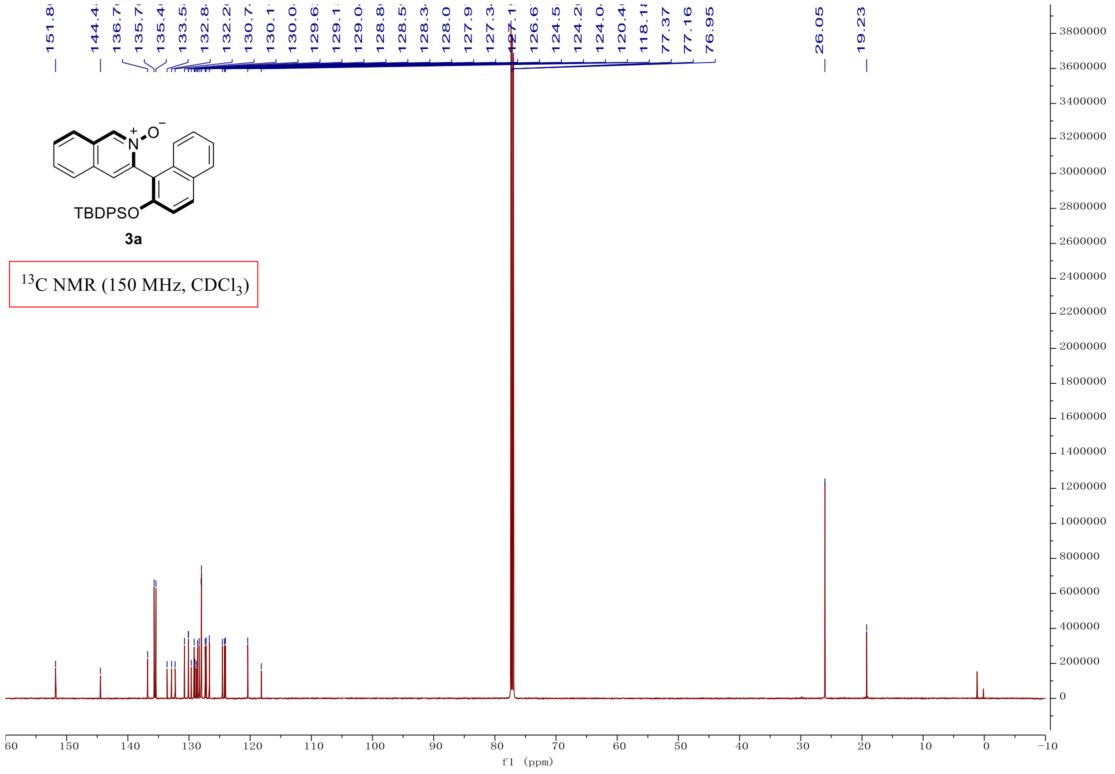
TBDPSO
3a
^{13}C NMR (150 MHz, $CDCl_3$)
f1 (ppm)

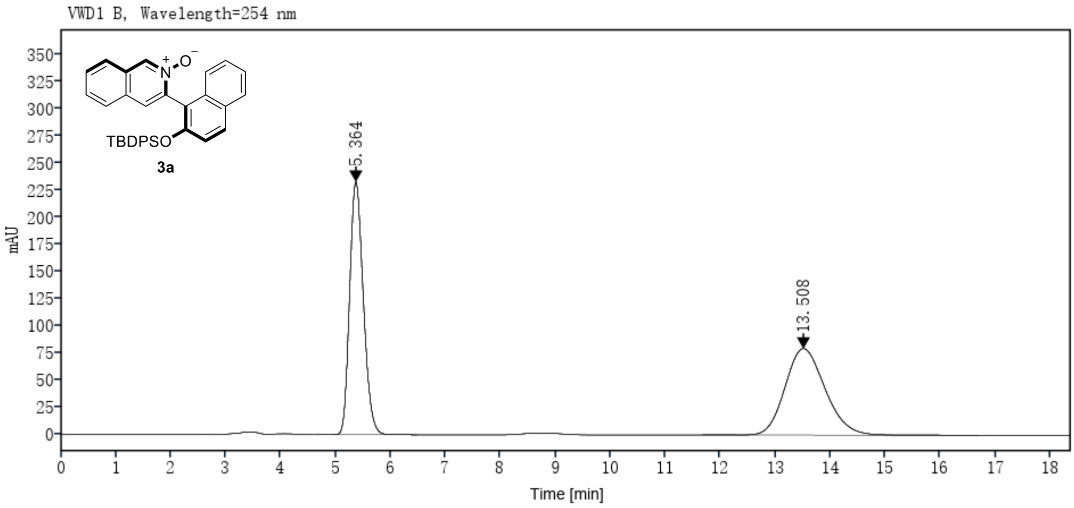

Signal: VWD1 B, Wavelength=254 nm

RT [min]Type	Area	Width[min]	Area%
5.364	4024.3220	0.268	49.7284
13.508	4068.2878	0.774	50.2716
Sum	8092.6099		

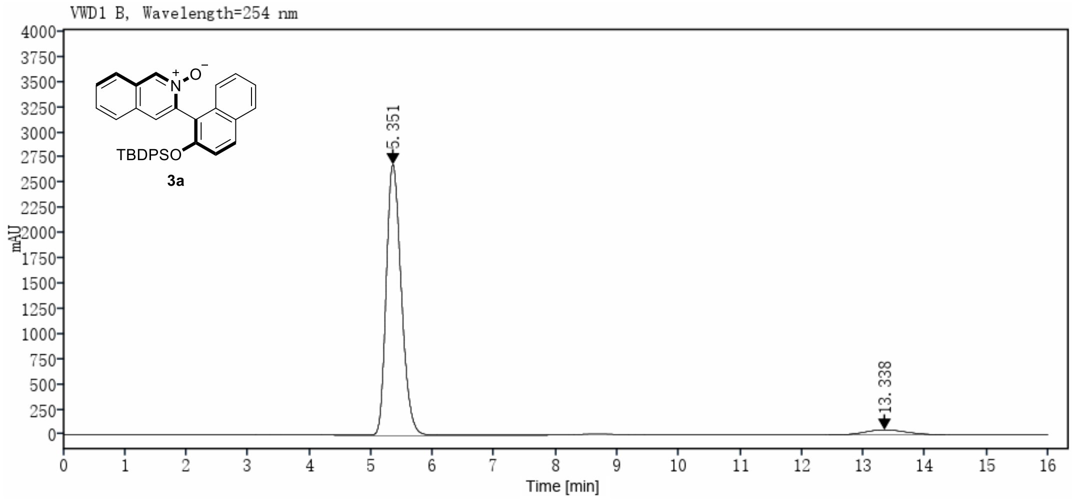

Signal: VWD1 B, Wavelength=254 nm

RT [min]Type	Area	Width[min]	Area%
5.351	46295.8906	0.261	95.4634
13.338	2200.0750	0.720	4.5366
Sum	48495.9656		

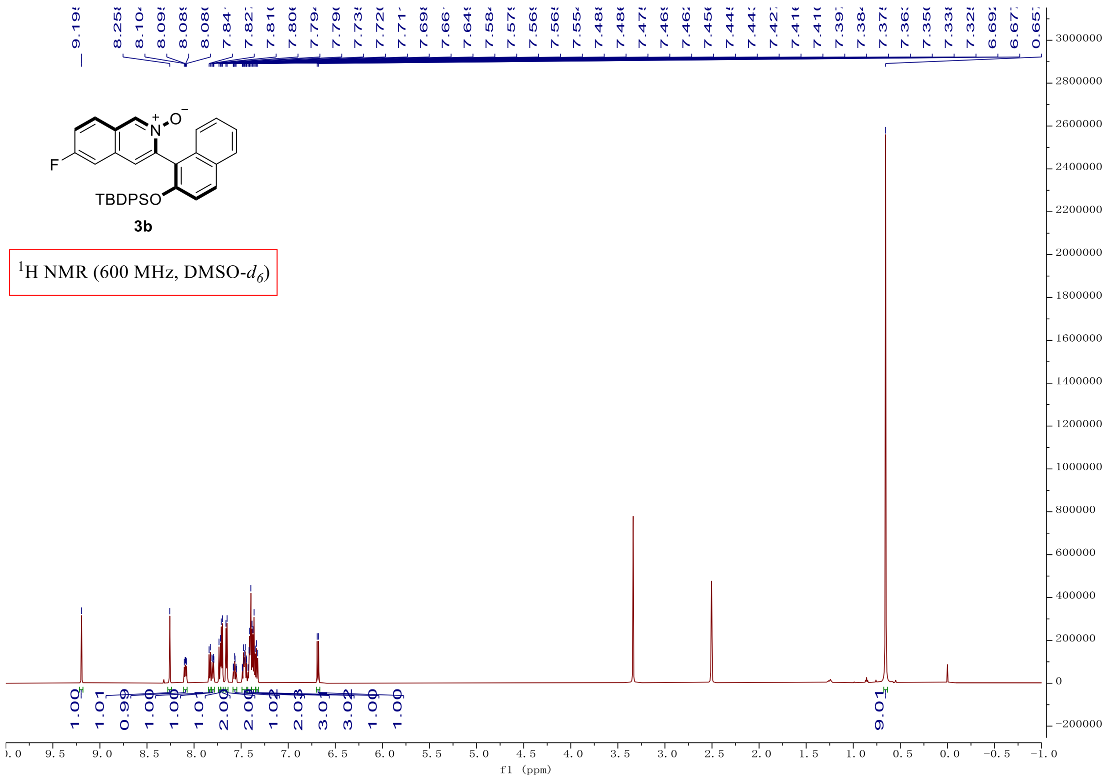
3b
^{1}H NMR (600 MHz, DMSO-d_6)
f1 (ppm)

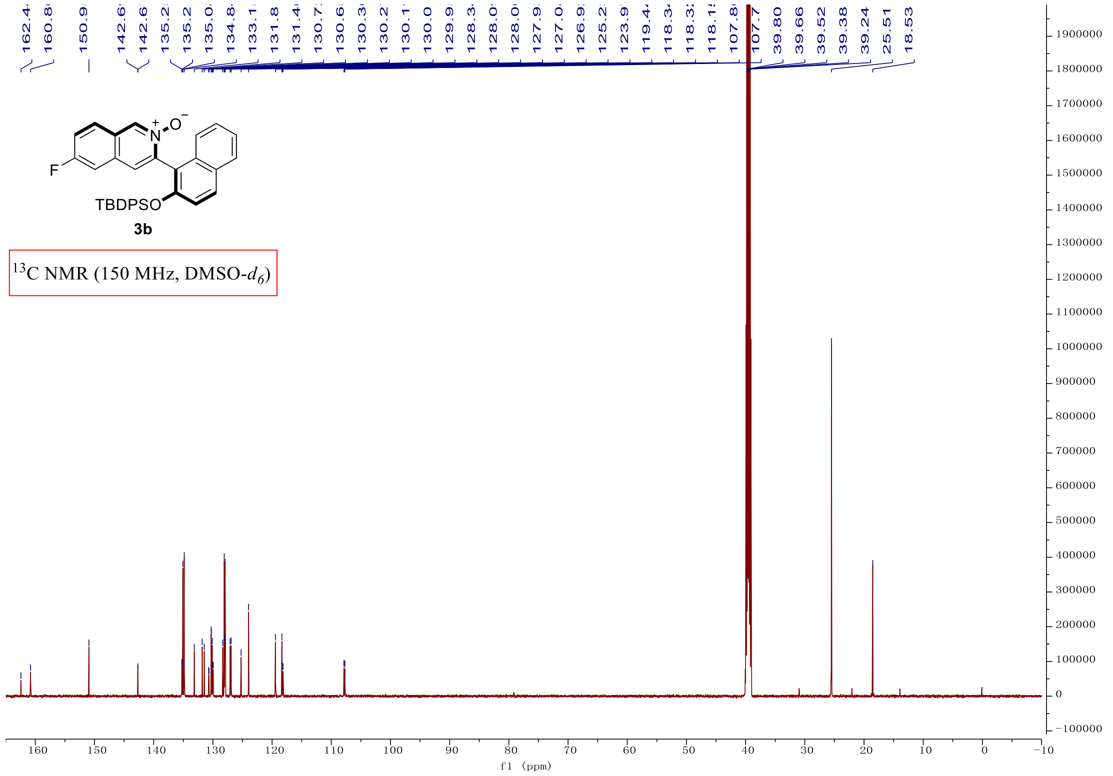
3b
^{13}C NMR (150 MHz, DMSO-d_6)
f1 (ppm)

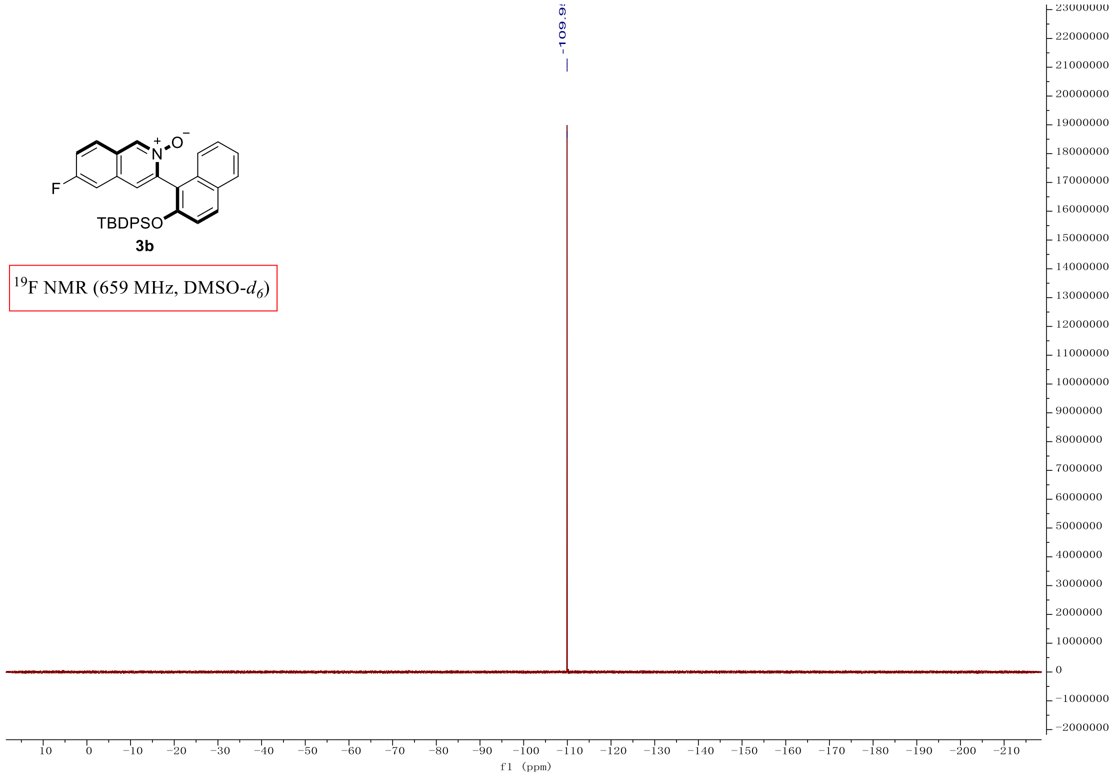

^{19}F NMR (659 MHz, DMSO-d_6)

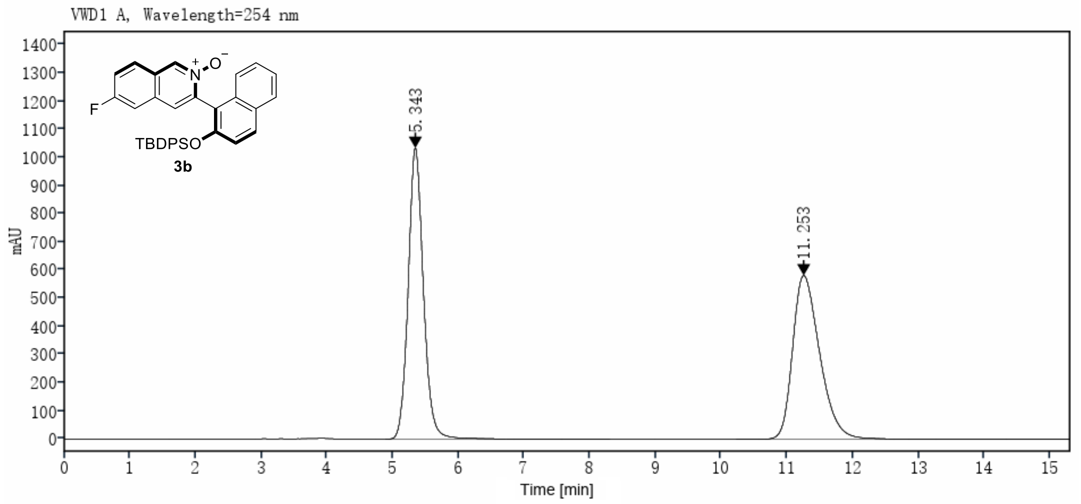

Signal: VWD1 A, Wavelength=254 nm

RT [min]Type	Area	Width[min]	Area%
5.343	16650.0547	0.239	49.8250
11.253	16766.9902	0.434	50.1750
Sum	33417.0449		

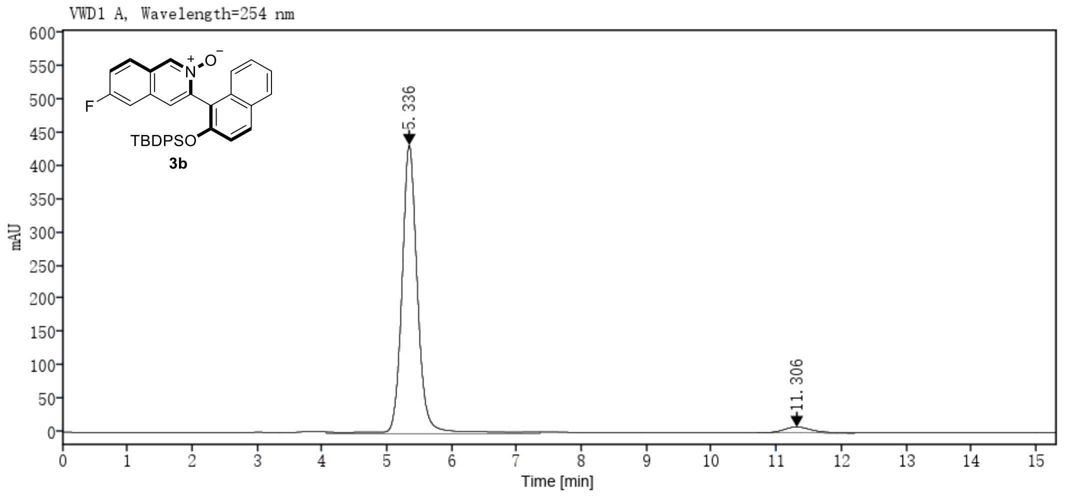

Signal: VWD1 A, Wavelength=254 nm

RT [min]Type	Area	Width[min]	Area%
5.336	7303.0542	0.238	96.4136
11.306	271.6592	0.433	3.5864
Sum	7574.7134		

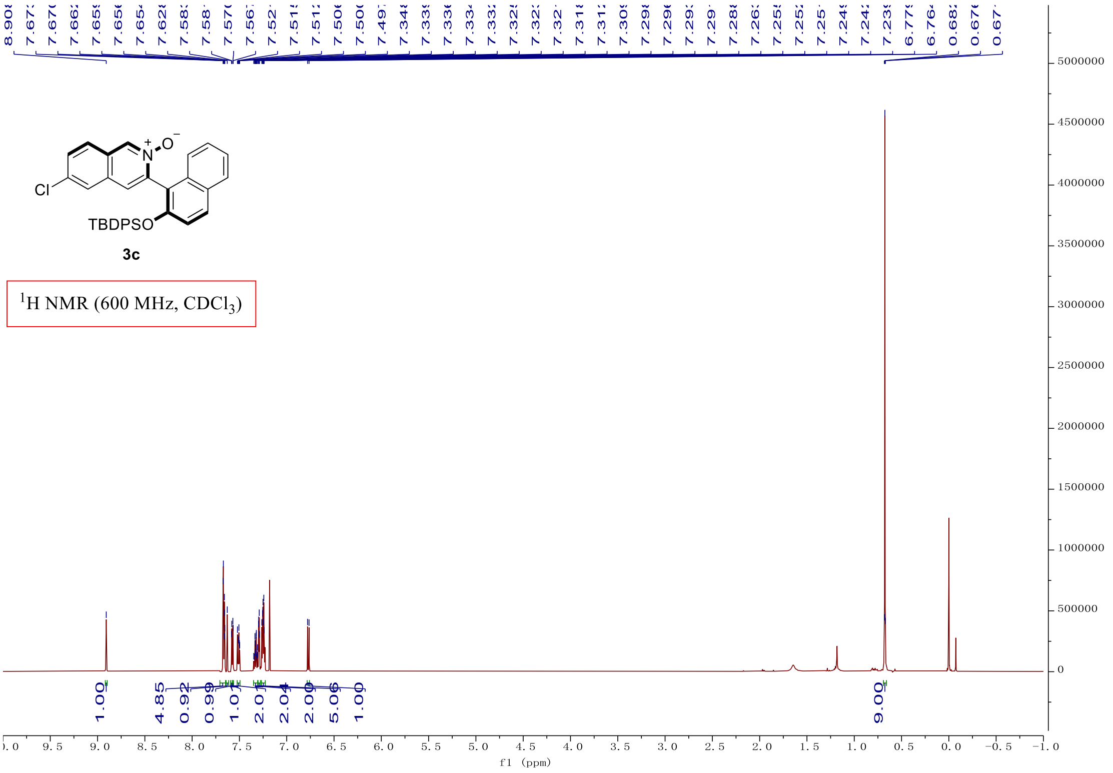
Cl
TBDPSO
3c
1H NMR (600 MHz, CDCl3)
f1 (ppm)

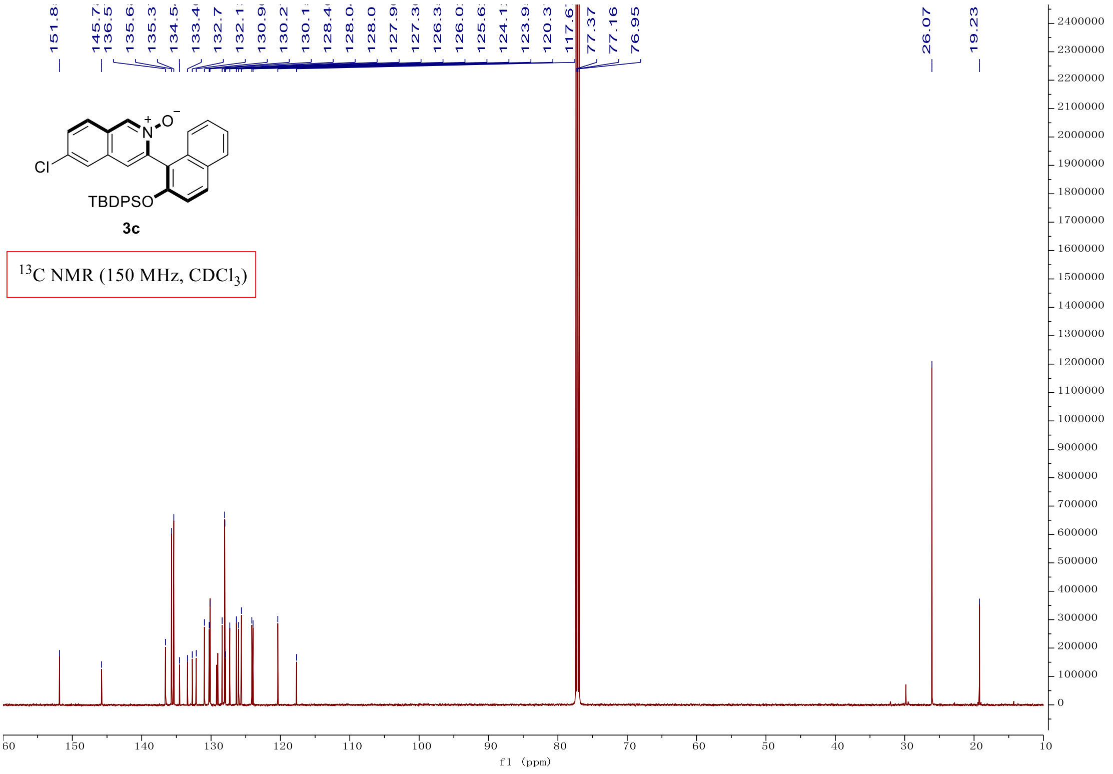
Cl
TBDPSO
3c
13C NMR (150 MHz, CDCl3)
f1 (ppm)

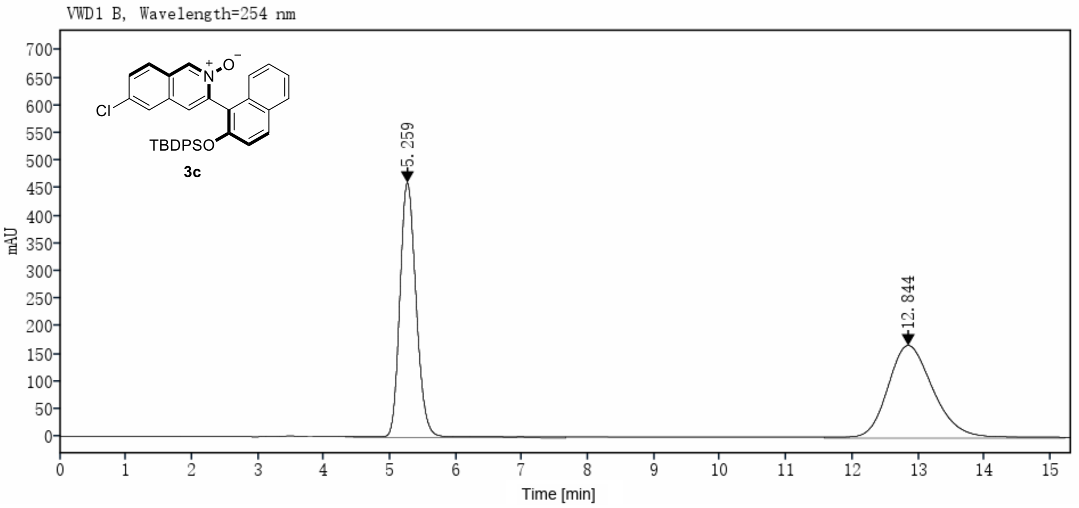

Signal: VWD1 B, Wavelength=254 nm

RT [min]Type	Area	Width[min]	Area%
5.259	8095.6221	0.262	49.8715
12.844	8137.3311	0.713	50.1285
Sum	16232.9531		

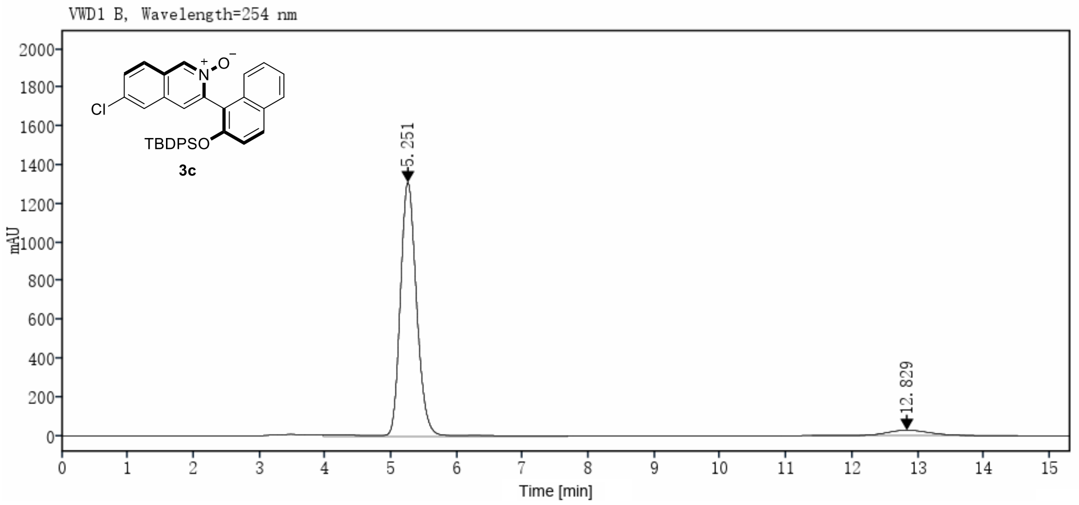

Signal: VWD1 B, Wavelength=254 nm

RT [min]Type	Area	Width[min]	Area%
5.251	23231.6406	0.260	94.9685
12.829	1230.8274	0.700	5.0315
Sum	24462.4680		

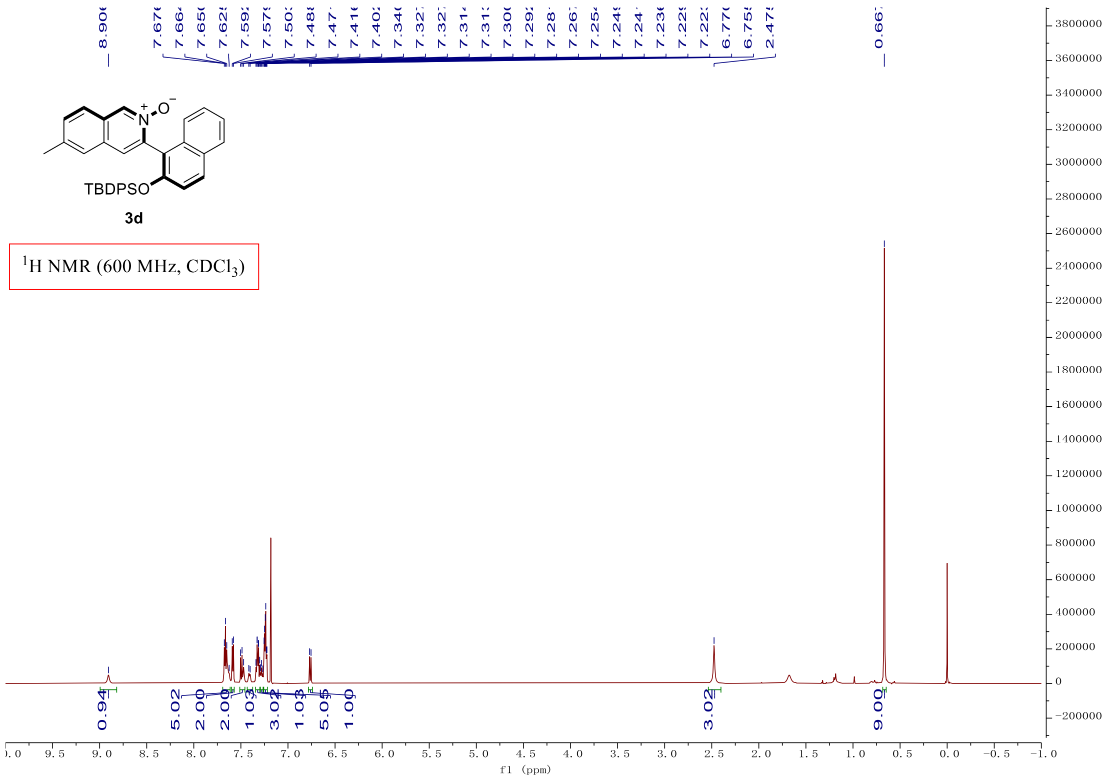
TBDPSO
3d
^{1}H NMR (600 MHz, $CDCl_3$)
f1 (ppm)

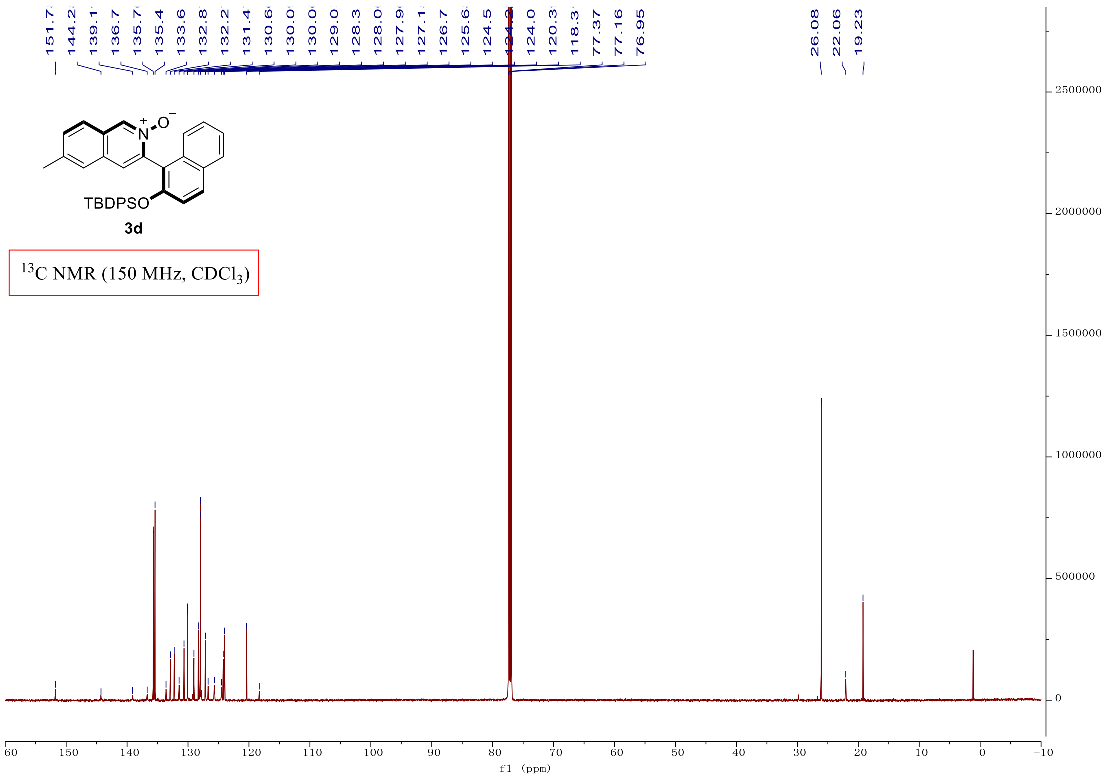
TBDPSO
3d
^{13}C NMR (150 MHz, $CDCl_3$)
f1 (ppm)

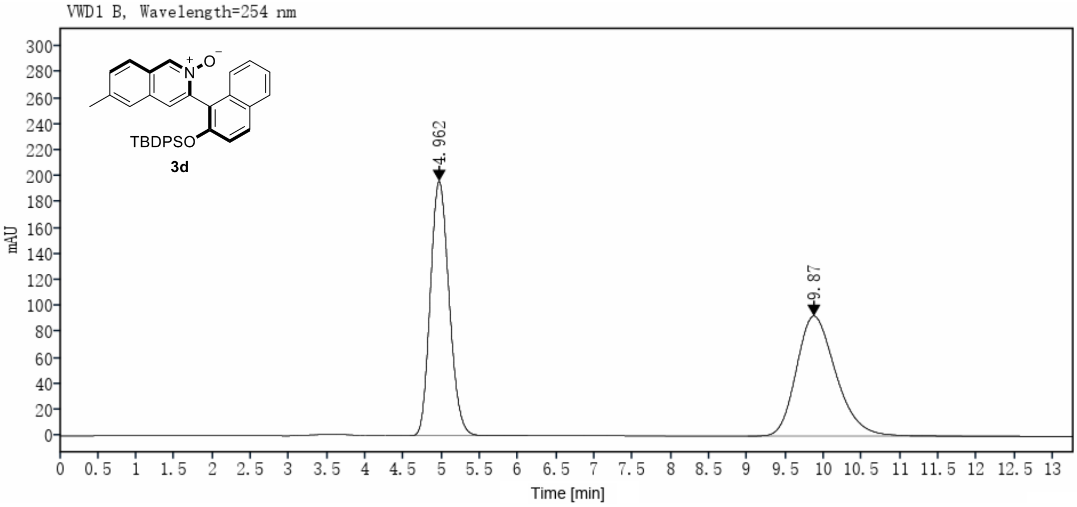

Signal: VWD1 B, Wavelength=254 nm

RT [min]Type	Area	Width[min]	Area%
4.962	3342.0073	0.266	50.0730
9.870	3332.2571	0.546	49.9270
Sum	6674.2644		

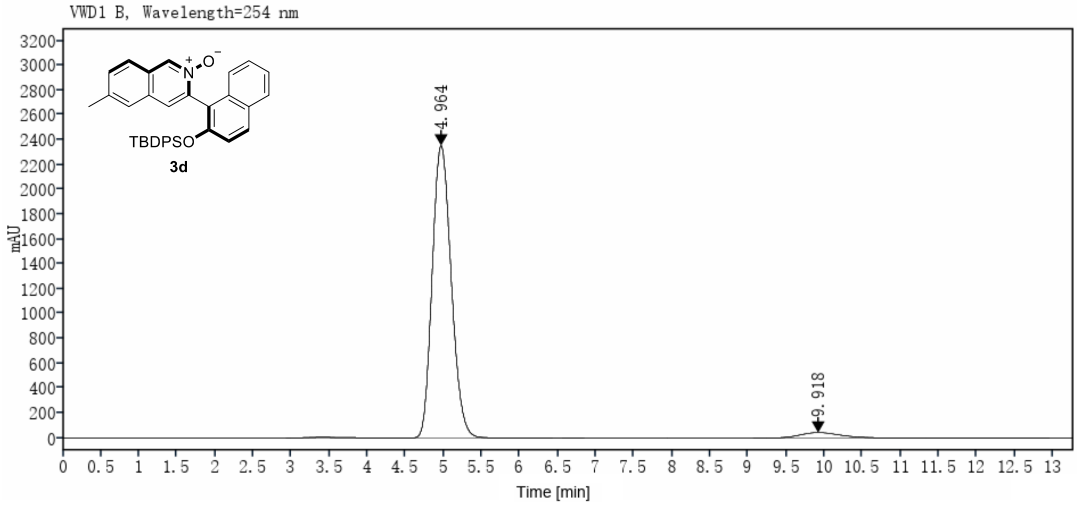

Signal: VWD1 B, Wavelength=254 nm

RT [min]Type	Area	Width[min]	Area%
4.964	41101.6523	0.271	96.2683
9.918	1593.2446	0.559	3.7317
Sum	42694.8970		

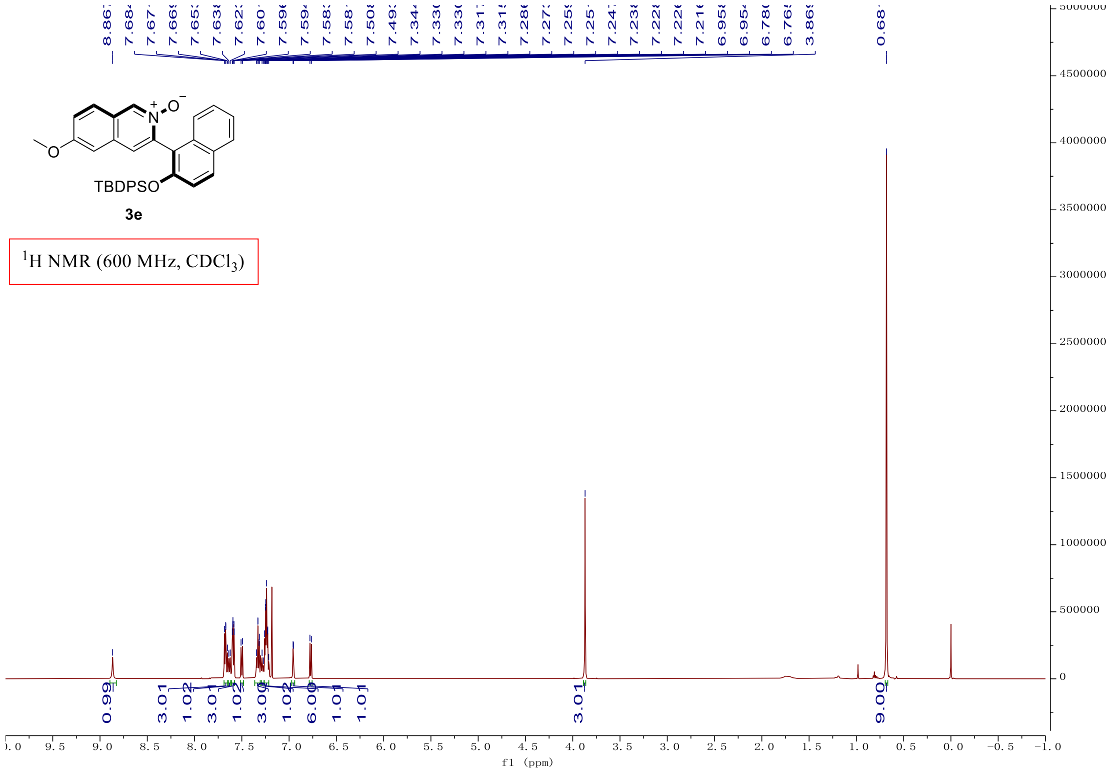
TBDPSO
3e
1H NMR (600 MHz, CDCl3)
f1 (ppm)

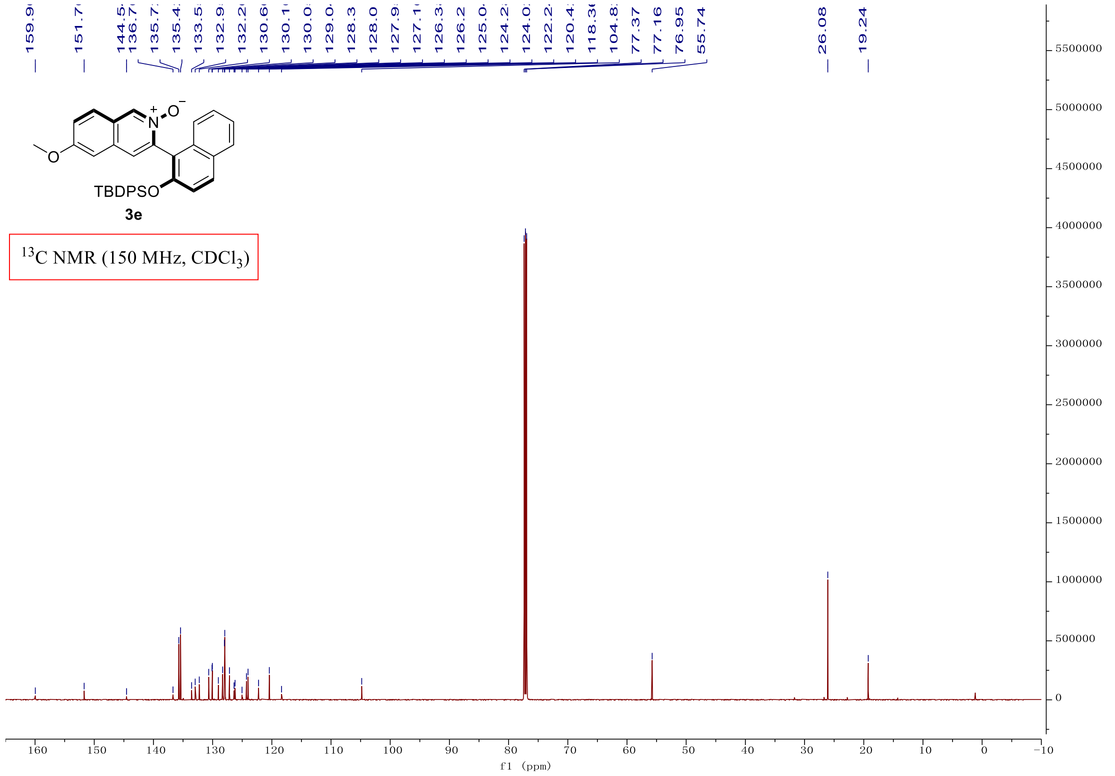
TBDPSO
3e
13C NMR (150 MHz, CDCl3)
f1 (ppm)

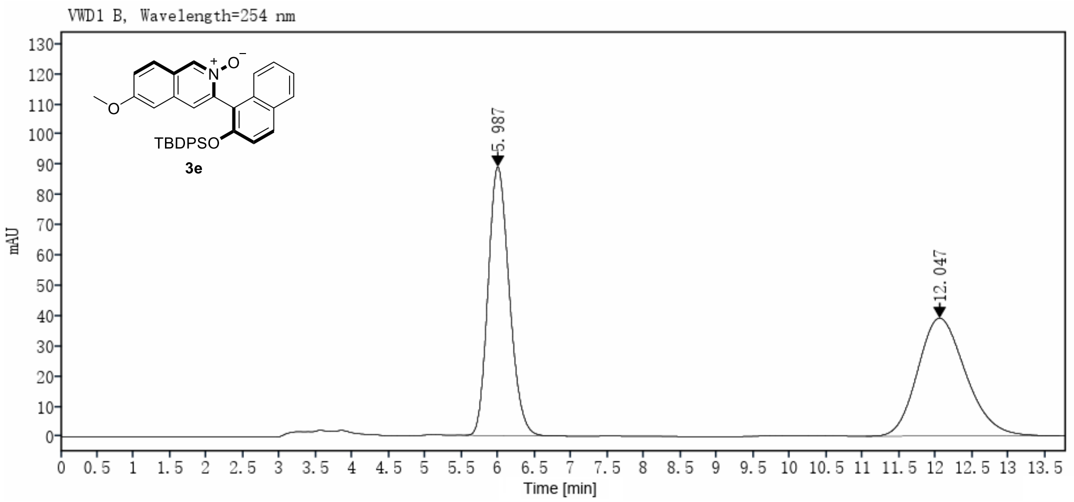

Signal: VWD1 B, Wavelength=254 nm

RT [min]Type	Area	Width[min]	Area%
5.987	1796.3334	0.315	50.1821
12.047	1783.2994	0.712	49.8179
Sum	3579.6328		

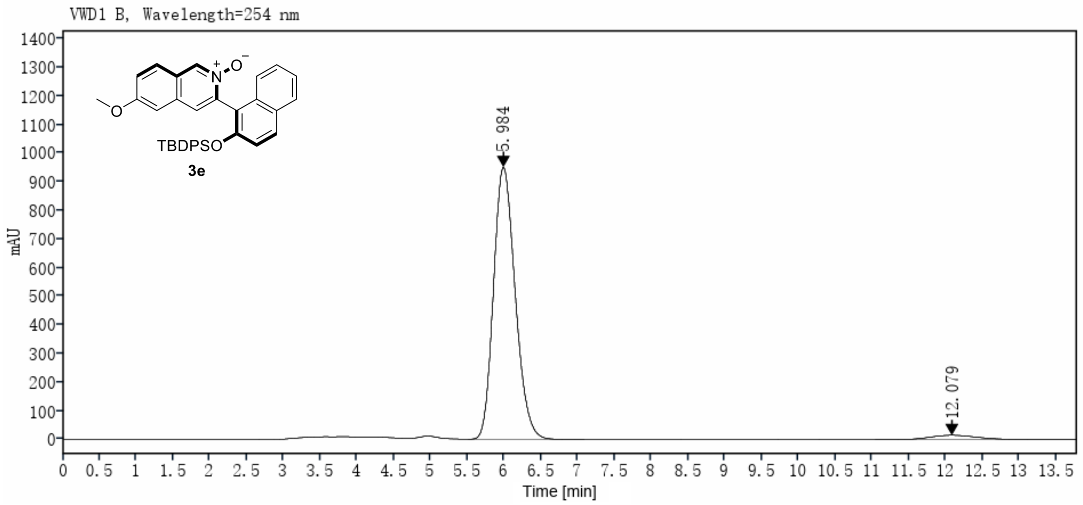

Signal: VWD1 B, Wavelength=254 nm

RT [min]Type	Area	Width[min]	Area%
5.984	19448.5410	0.317	96.4433
12.079	717.2386	0.707	3.5567
Sum	20165.7796		

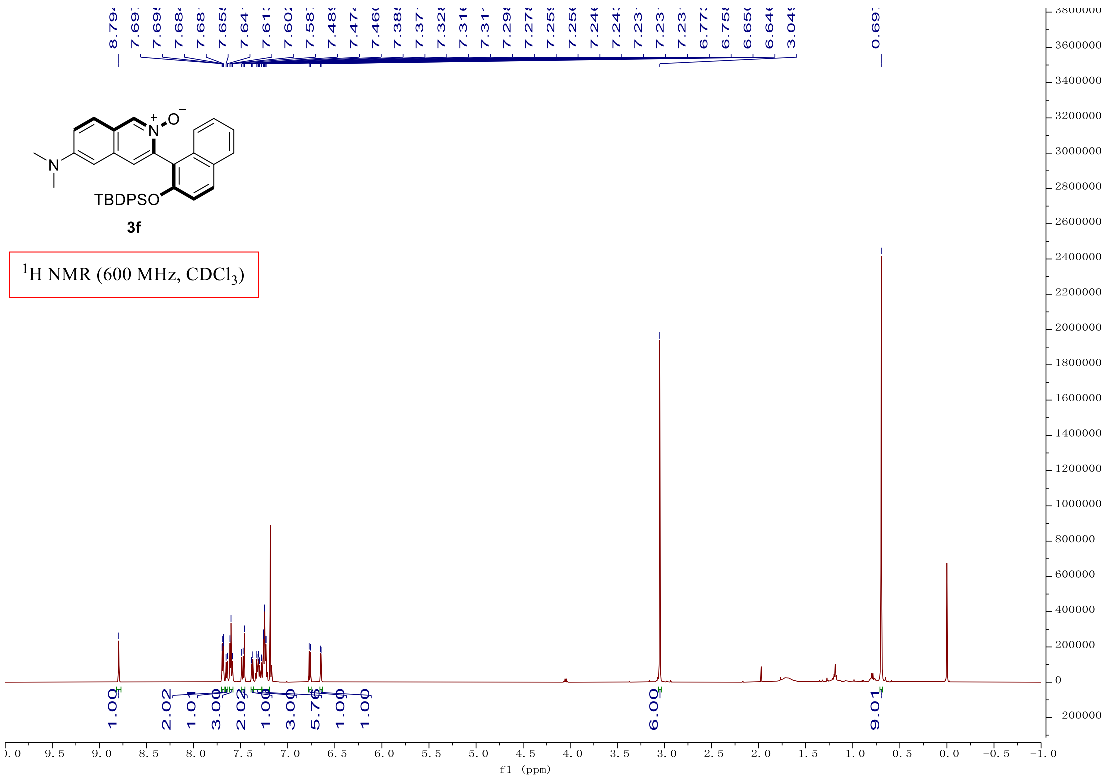

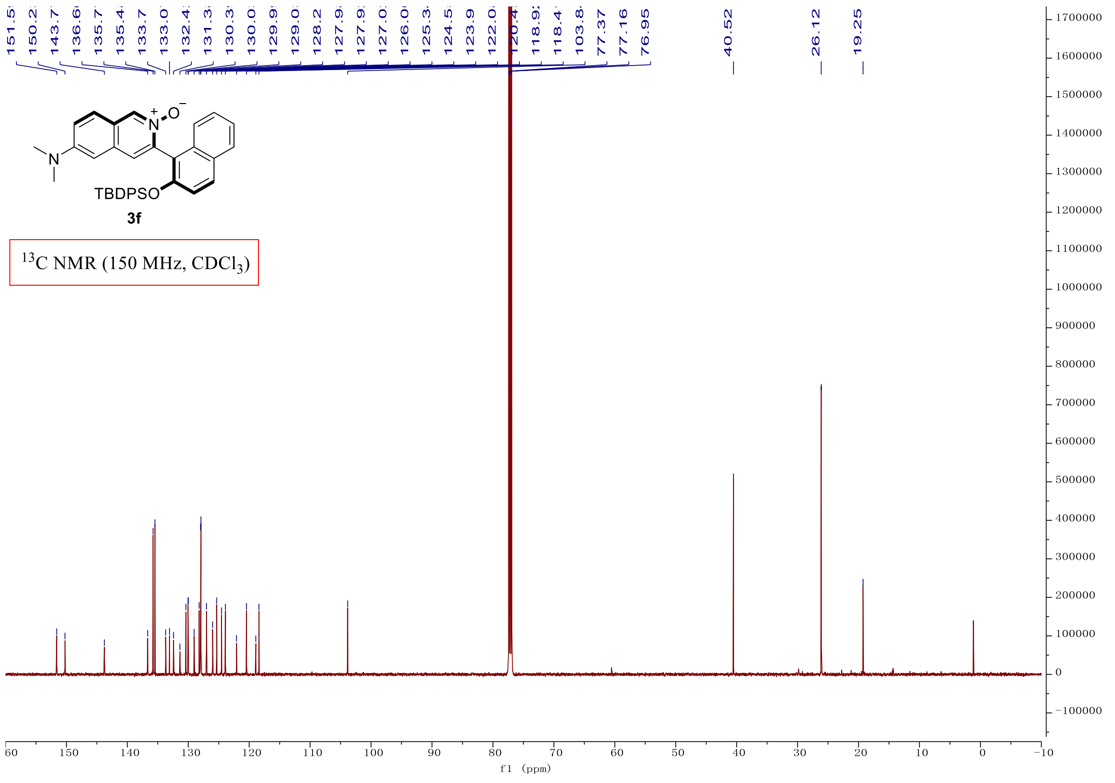

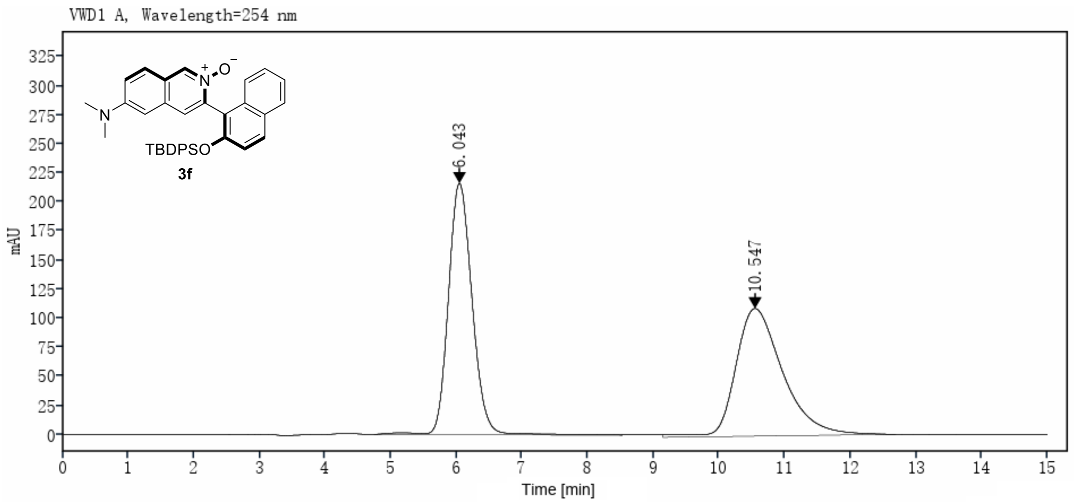

Signal: VWD1 A, Wavelength=254 nm

RT [min]Type	Area	Width[min]	Area%
6.043	5469.5303	0.384	50.0677
10.547	5454.7485	0.727	49.9323
Sum	10924.2788		

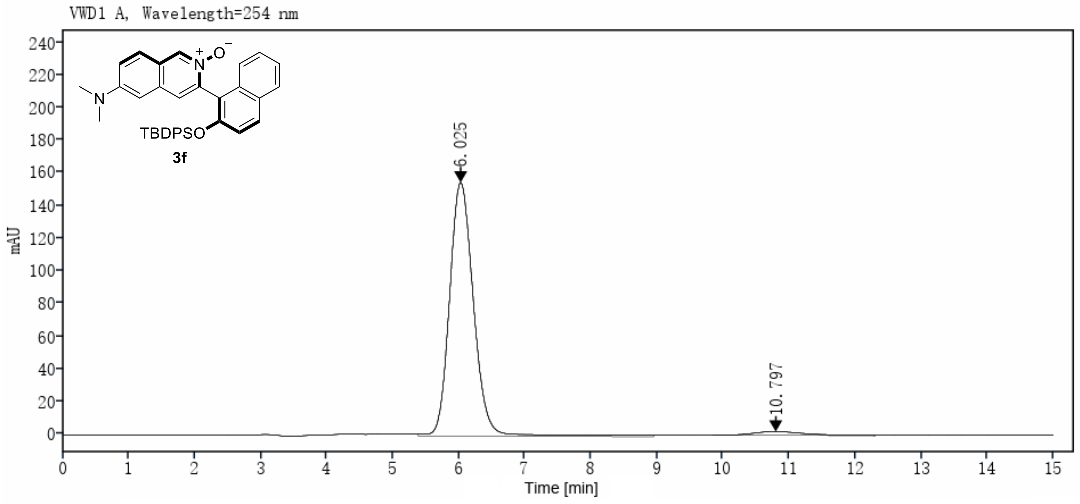

Signal: VWD1 A, Wavelength=254 nm

RT [min]Type	Area	Width[min]	Area%
6.025	3971.4353	0.379	96.9570
10.797	124.6439	0.753	3.0430
Sum	4096.0792		

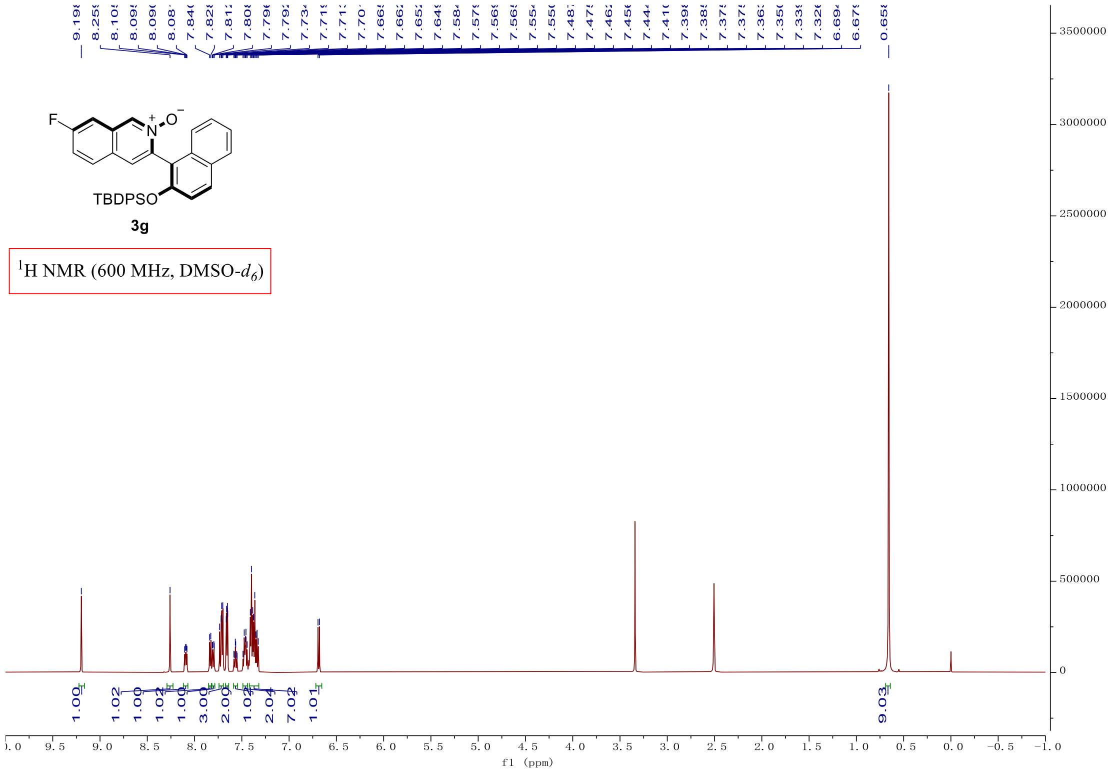
TBDPSO
3g
1H NMR (600 MHz, DMSO-d6)
f1 (ppm)

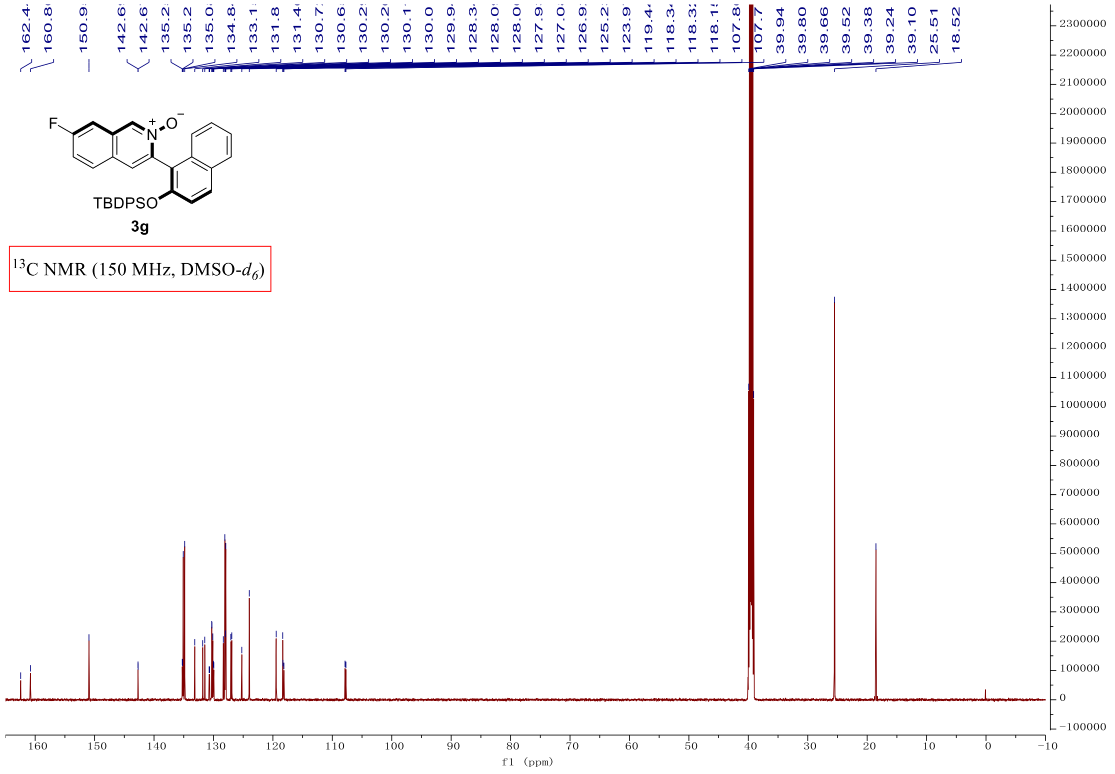
TBDPSO
3g
13C NMR (150 MHz, DMSO-d6)
f1 (ppm)

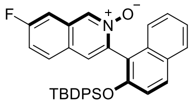

3g

^{19}F NMR (659 MHz, DMSO-d_6)

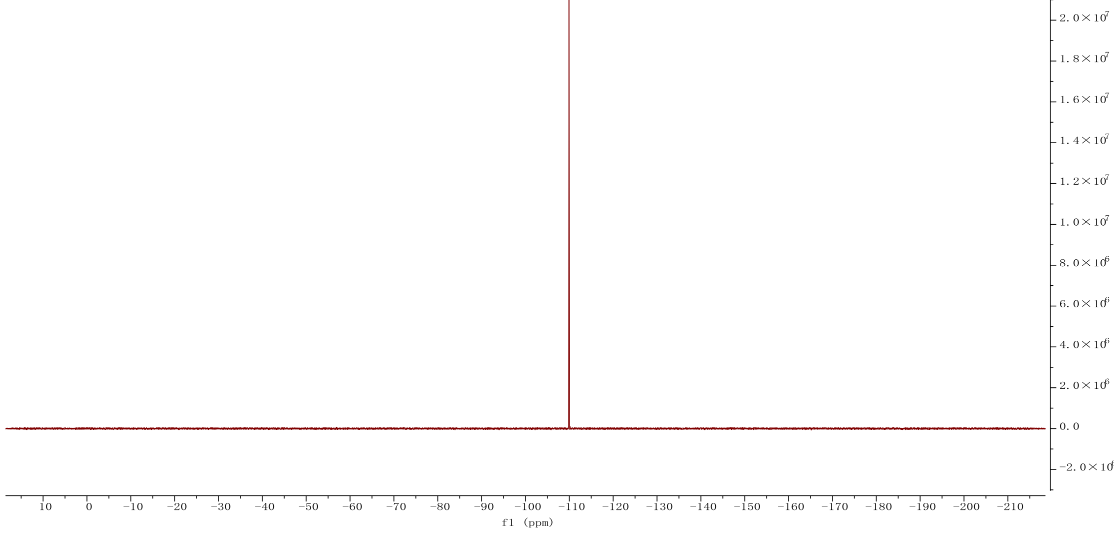

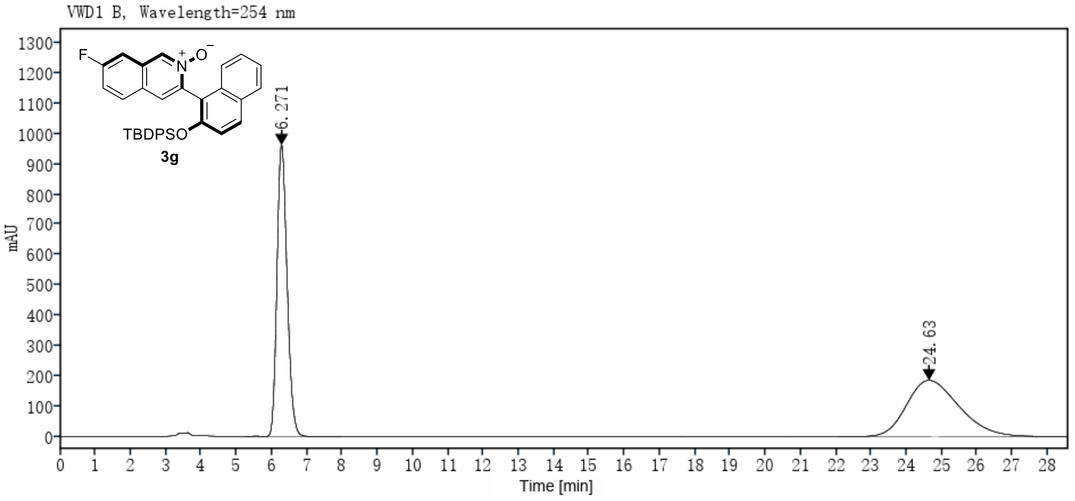

Signal: VWD1 B, Wavelength=254 nm

RT [min]Type	Area	Width[min]	Area%
6.271	19192.8691	0.309	49.9971
24.630	19195.1328	1.600	50.0029
Sum	38388.0020		

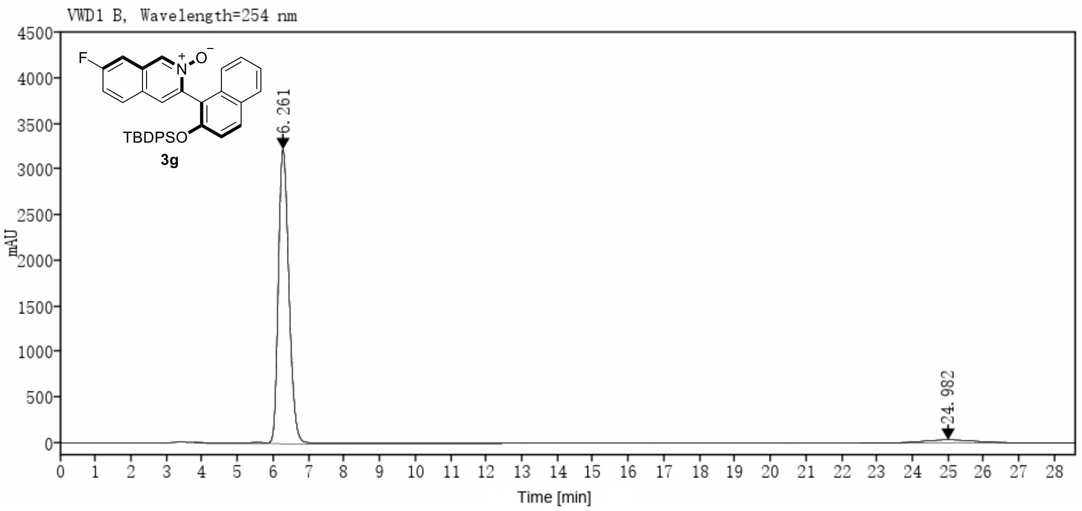

Signal: VWD1 B, Wavelength=254 nm

RT [min]Type	Area	Width[min]	Area%
6.261	72480.9766	0.317	95.5508
24.982	3374.9722	1.614	4.4492
Sum	75855.9487		

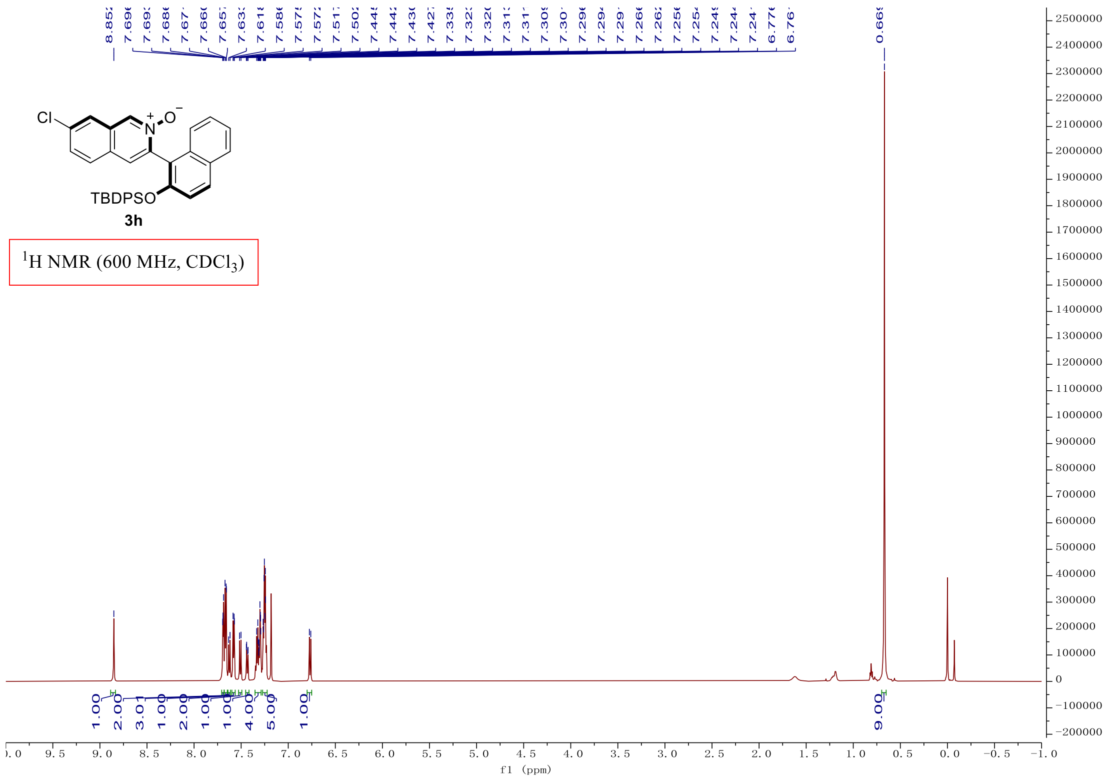
Cl
TBDPSO
3h
^{1}H NMR (600 MHz, CDCl$_3$)
f1 (ppm)

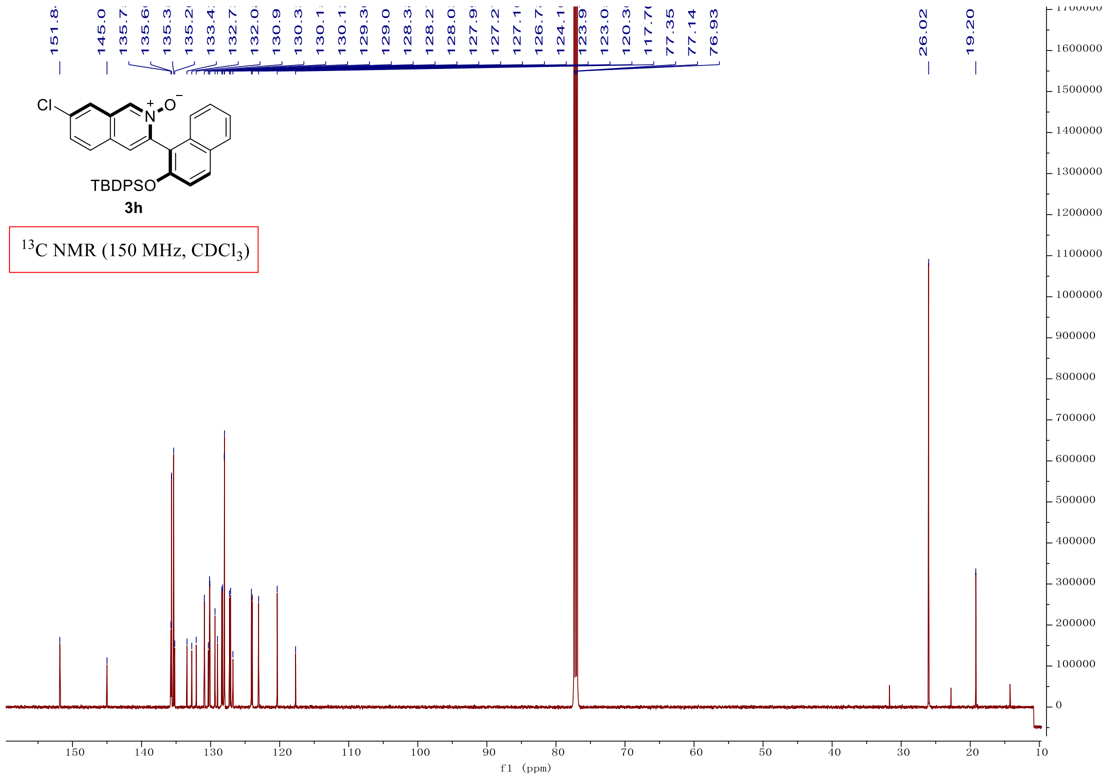
Cl
TBDPSO
3h
^{13}C NMR (150 MHz, CDCl$_3$)
f1 (ppm)

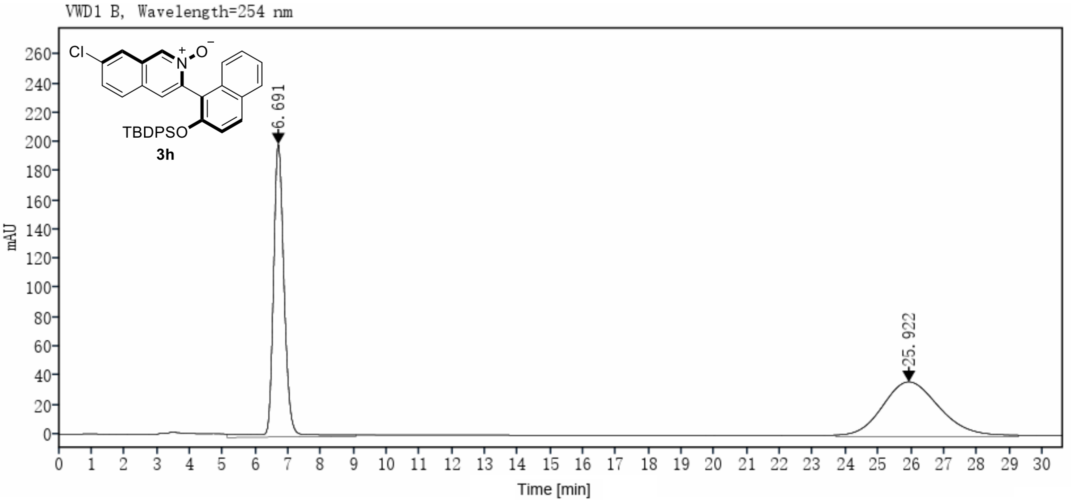

Signal: VWD1 B, Wavelength=254 nm

RT [min]Type	Area	Width[min]	Area%
6.691	4664.0747	0.331	50.7238
25.922	4530.9634	1.840	49.2762
Sum	9195.0381		

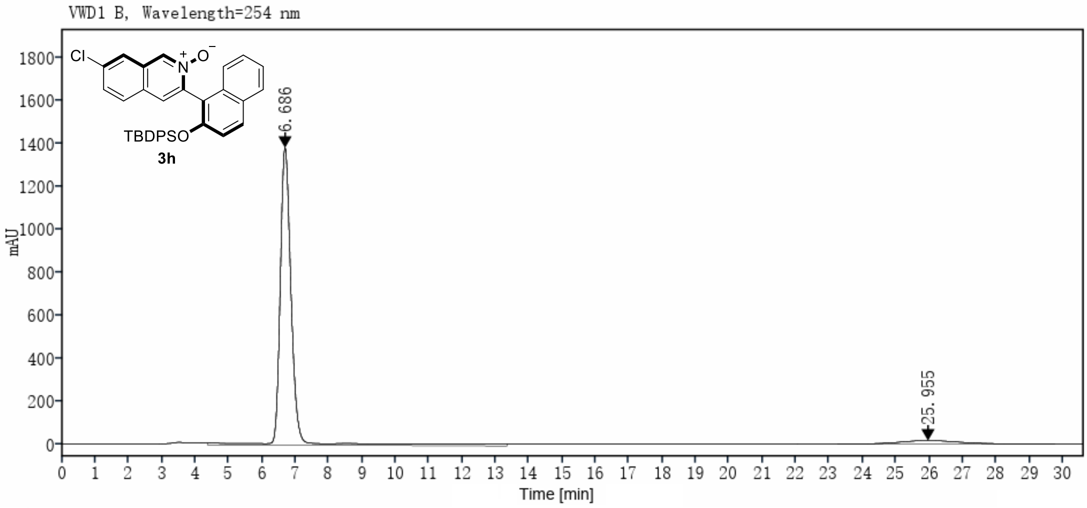

Signal: VWD1 B, Wavelength=254 nm

RT [min]Type	Area	Width[min]	Area%
6.686	34328.5625	0.334	95.0258
25.955	1796.9672	1.820	4.9742
Sum	36125.5297		

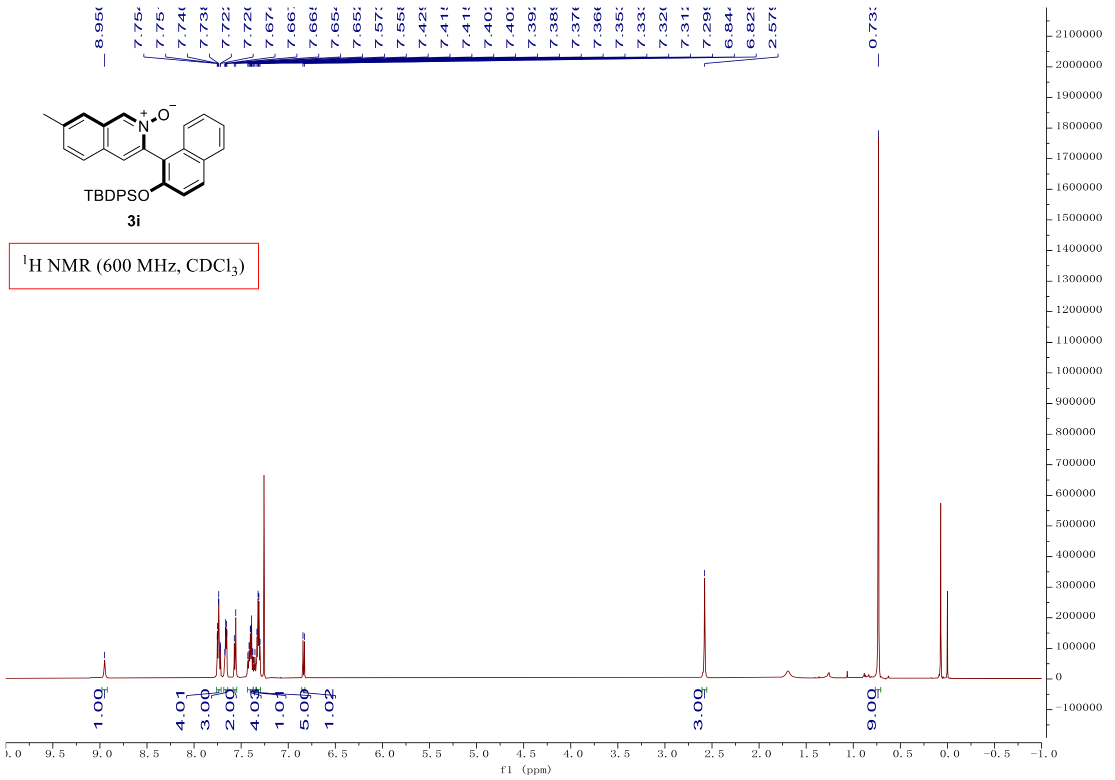
TBDPSO
3i
1H NMR (600 MHz, CDCl3)
f1 (ppm)

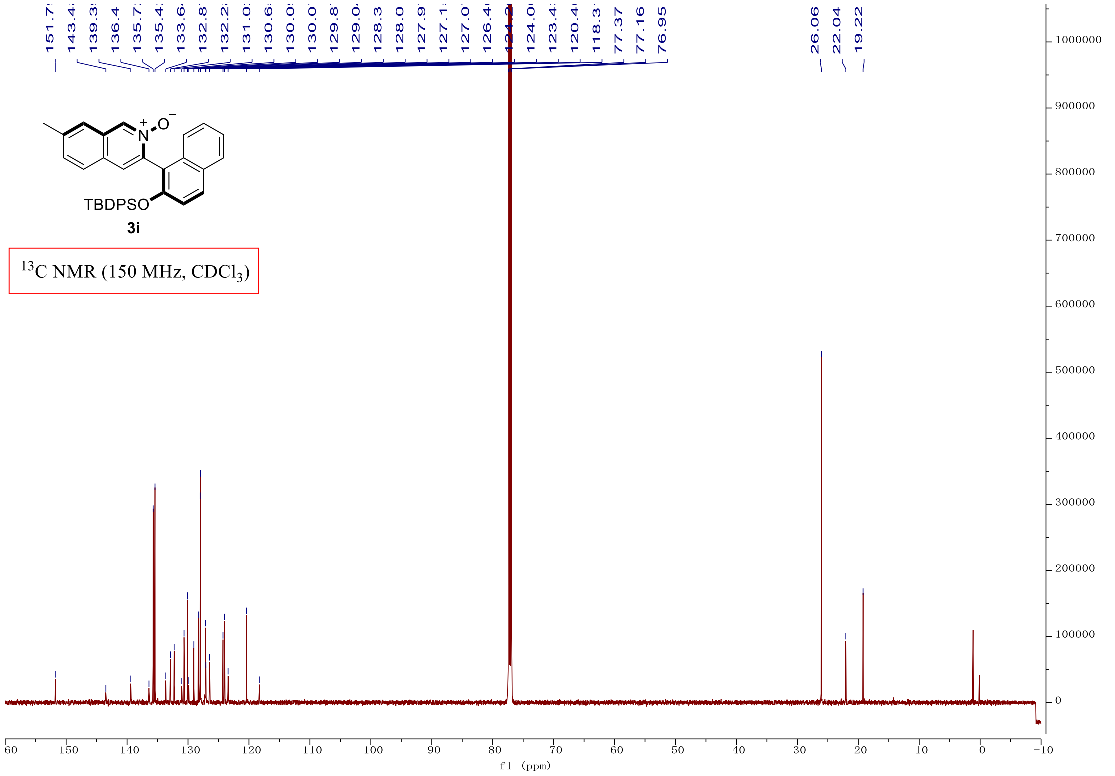
TBDPSO
3i
13C NMR (150 MHz, CDCl3)
f1 (ppm)

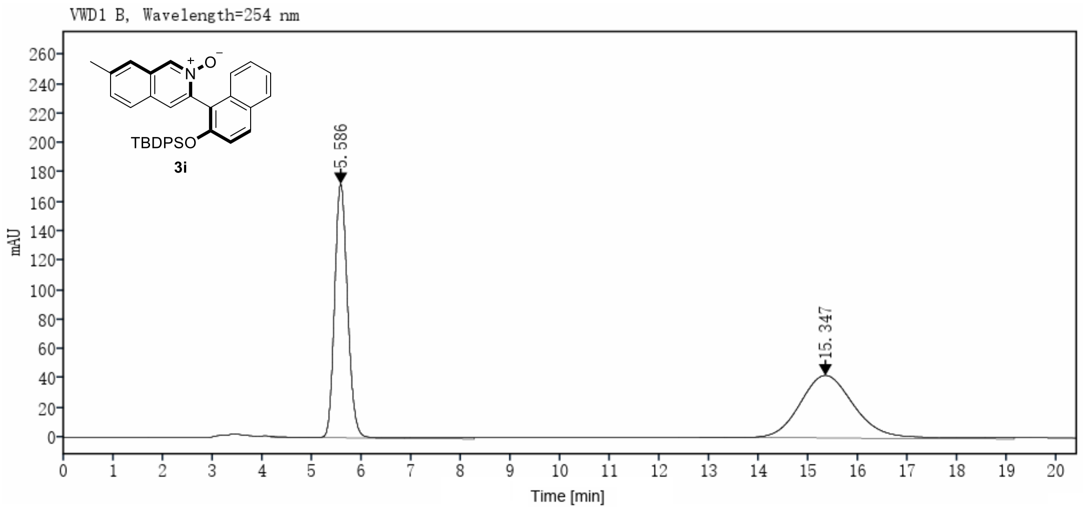

Signal: VWD1 B, Wavelength=254 nm

RT [min]Type	Area	Width[min]	Area%
5.586	3206.2683	0.278	49.4200
15.347	3281.5234	1.140	50.5800
Sum	6487.7917		

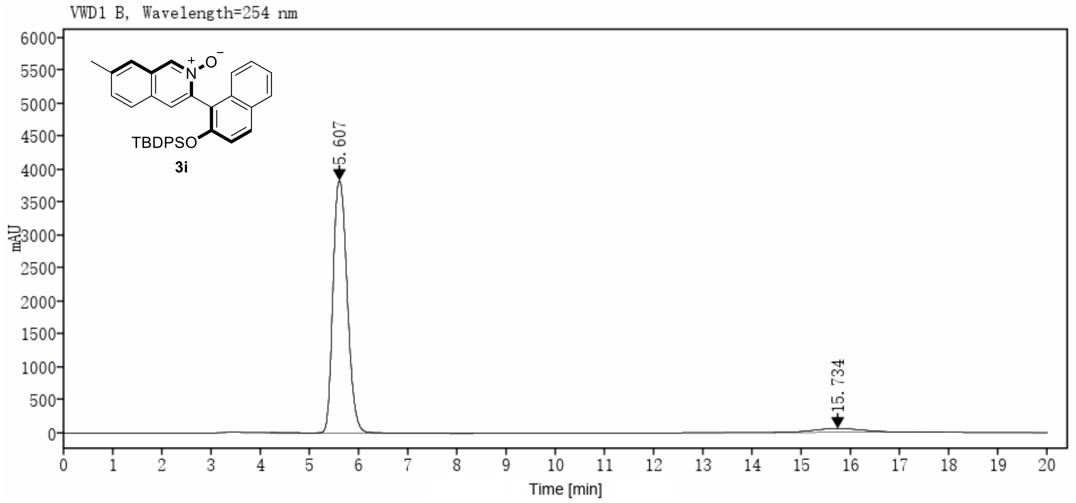

Signal: VWD1 B, Wavelength=254 nm

RT [min]Type	Area	Width[min]	Area%
5.607	78078.8672	0.313	94.9213
15.734	4177.5986	1.147	5.0787
Sum	82256.4658		

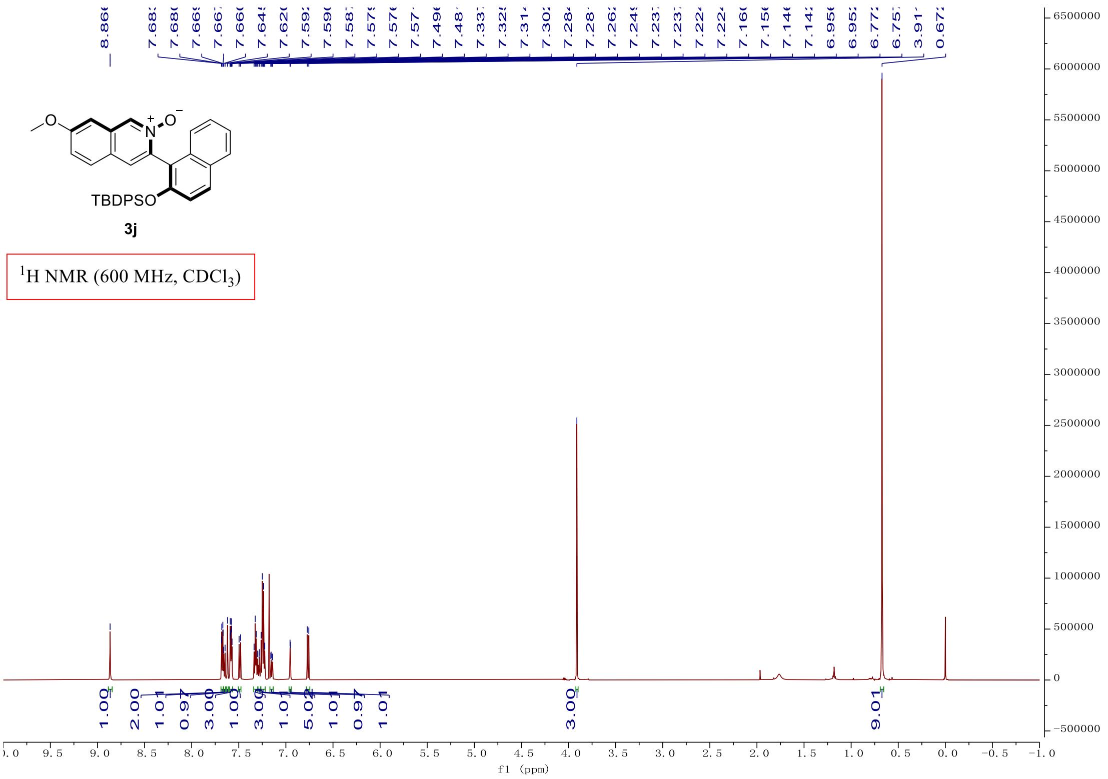
TBDPSO
3j
1H NMR (600 MHz, CDCl3)
3.00
9.01
f1 (ppm)

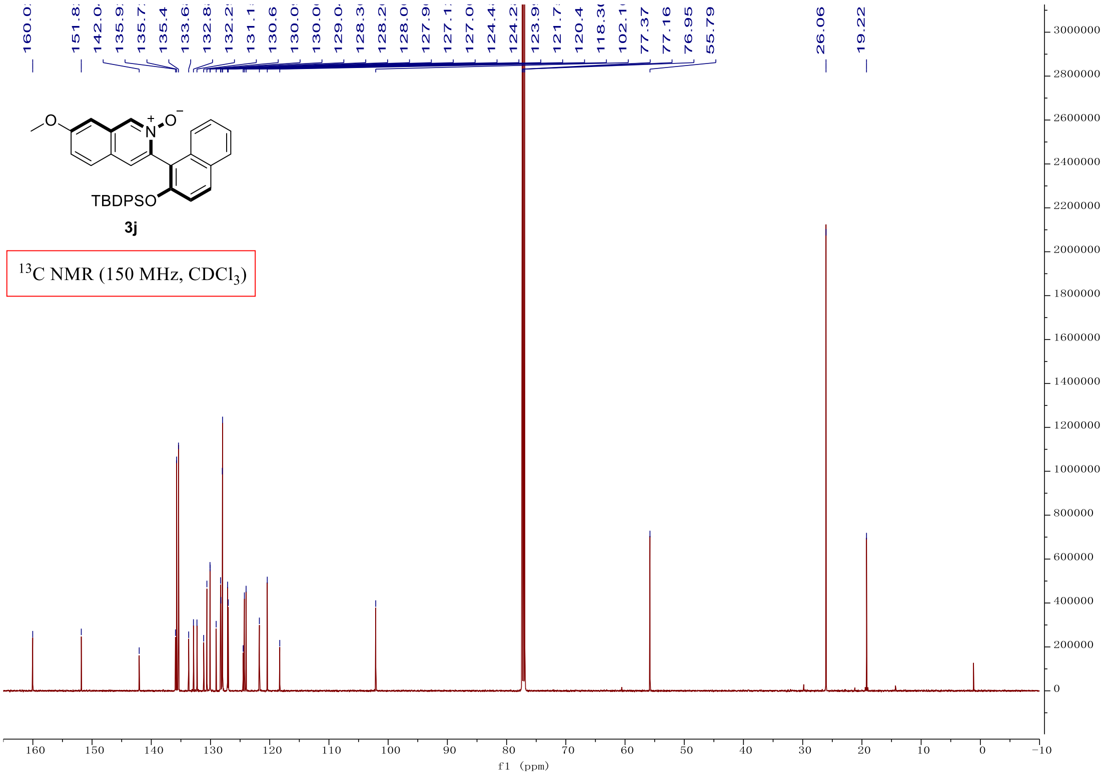
TBDPSO
3j
13C NMR (150 MHz, CDCl3)
77.37
77.16
76.95
55.79
26.06
19.22
f1 (ppm)

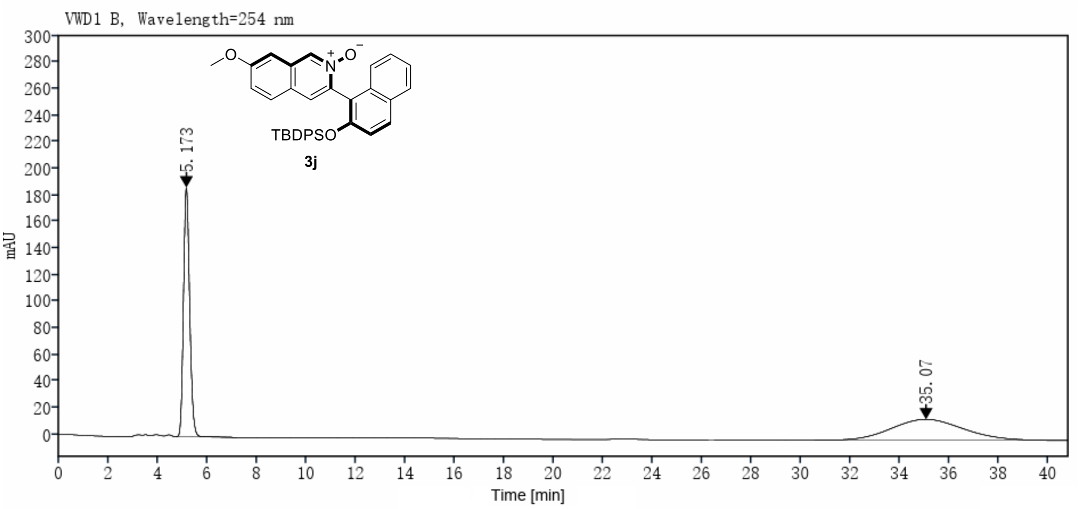

Signal: VWD1 B, Wavelength=254 nm

RT [min]Type	Area	Width[min]	Area%
5.173	3241.8818	0.264	50.1152
35.070	3226.9766	3.167	49.8848
Sum	6468.8584		

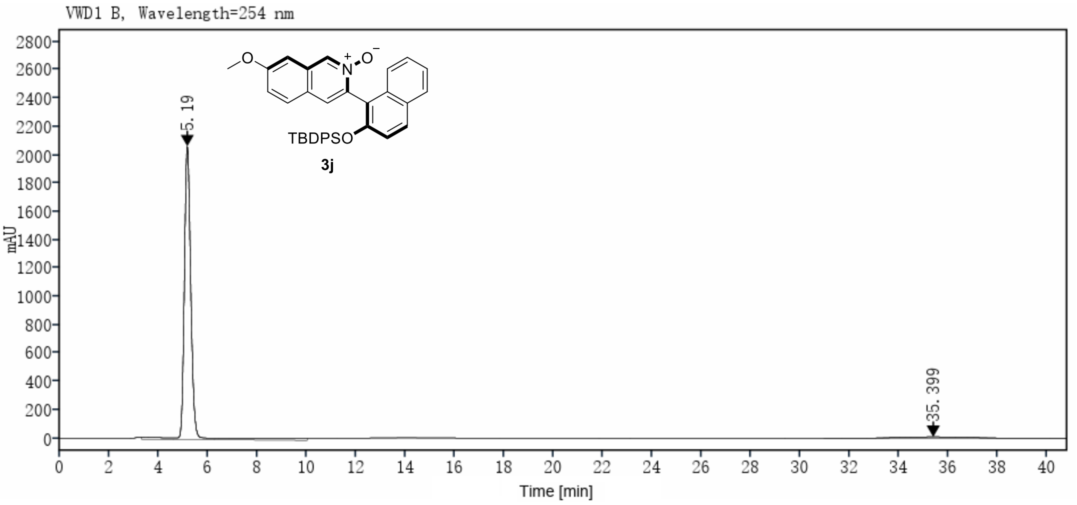

Signal: VWD1 B, Wavelength=254 nm

RT [min]Type	Area	Width[min]	Area%
5.190	41207.6523	0.273	95.0821
35.399	2131.3701	3.167	4.9179
Sum	43339.0225		

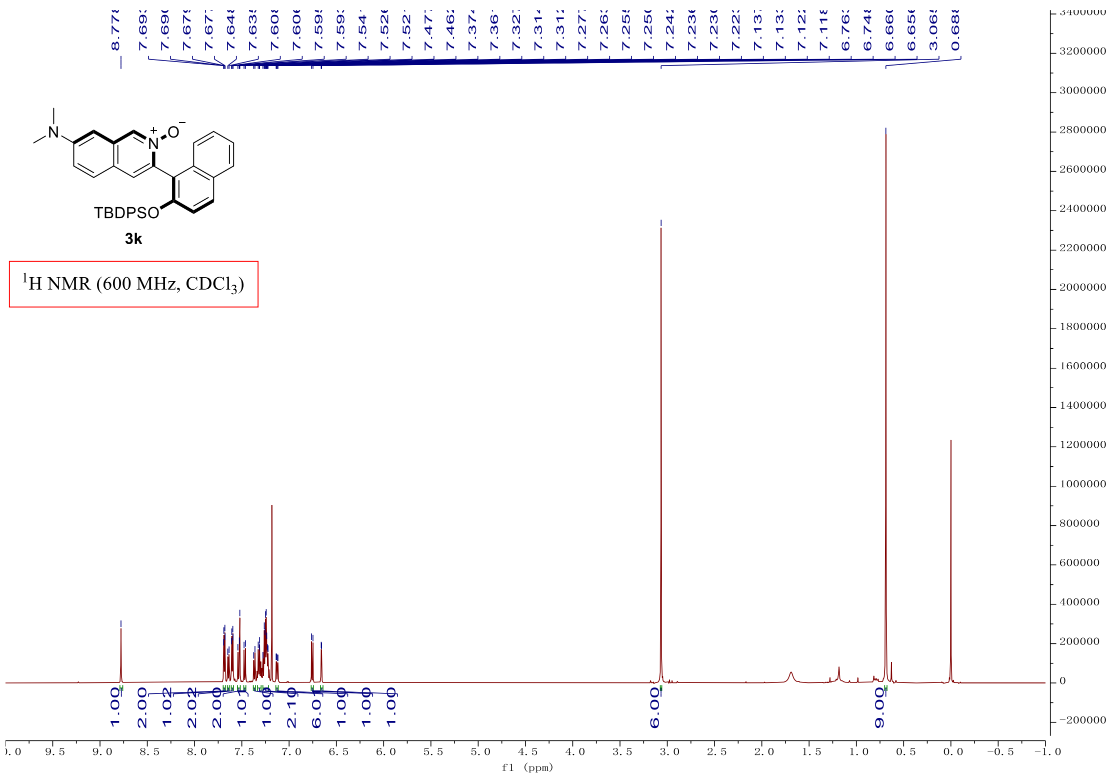
TBDPSO
3k
1H NMR (600 MHz, CDCl3)
f1 (ppm)

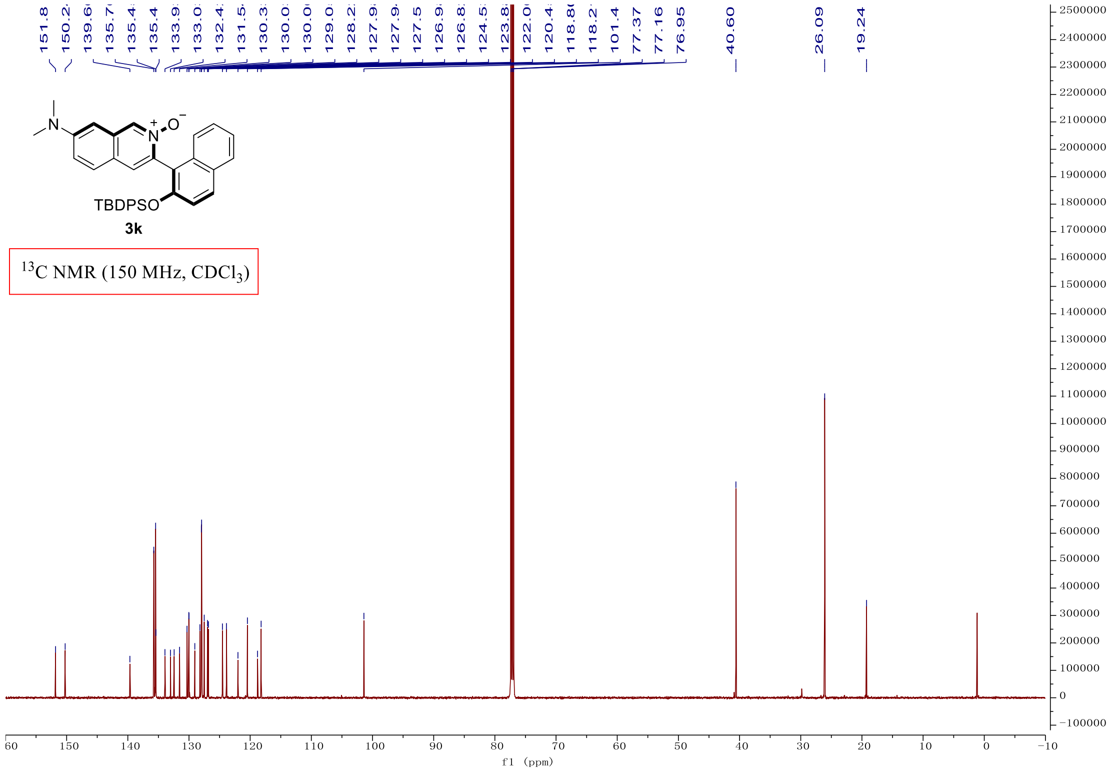
TBDPSO
3k
13C NMR (150 MHz, CDCl3)
f1 (ppm)

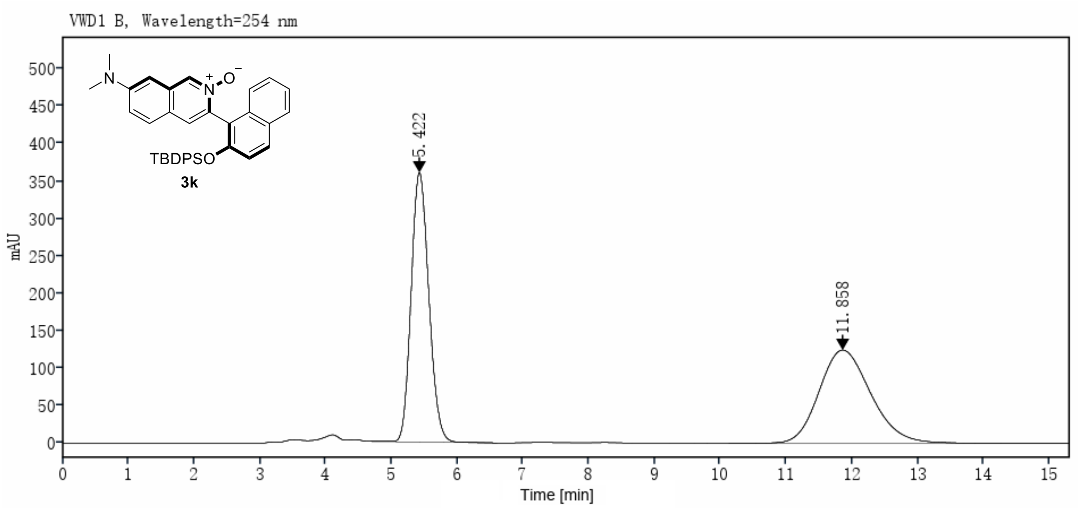

Signal: VWD1 B, Wavelength=254 nm

RT [min]Type	Area	Width[min]	Area%
5.422	6729.2114	0.293	49.6695
11.858	6818.7612	0.843	50.3305
Sum	13547.9727		

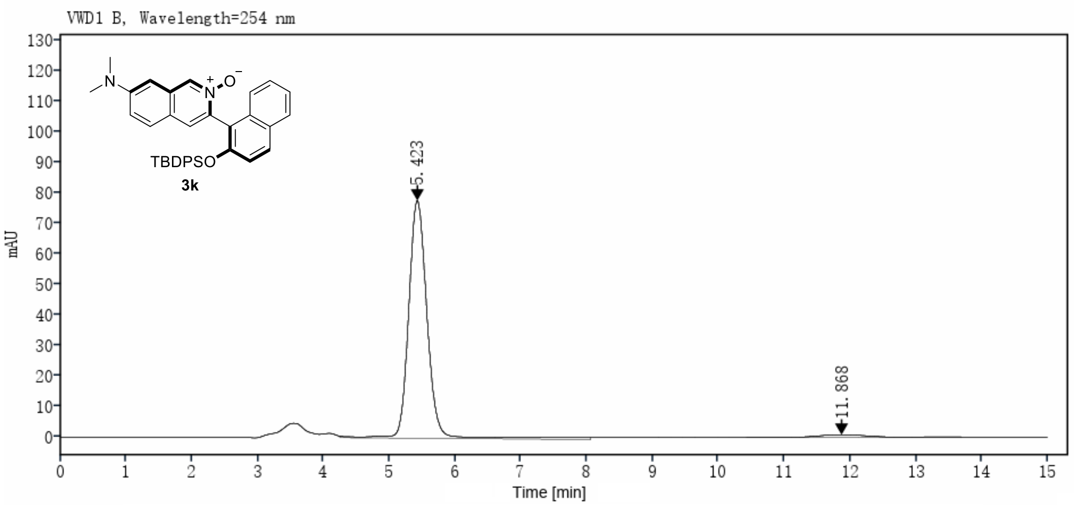

Signal: VWD1 B, Wavelength=254 nm

RT [min]Type	Area	Width[min]	Area%
5.423	1547.6713	0.288	97.5284
11.868	39.2221	0.787	2.4716
Sum	1586.8933		

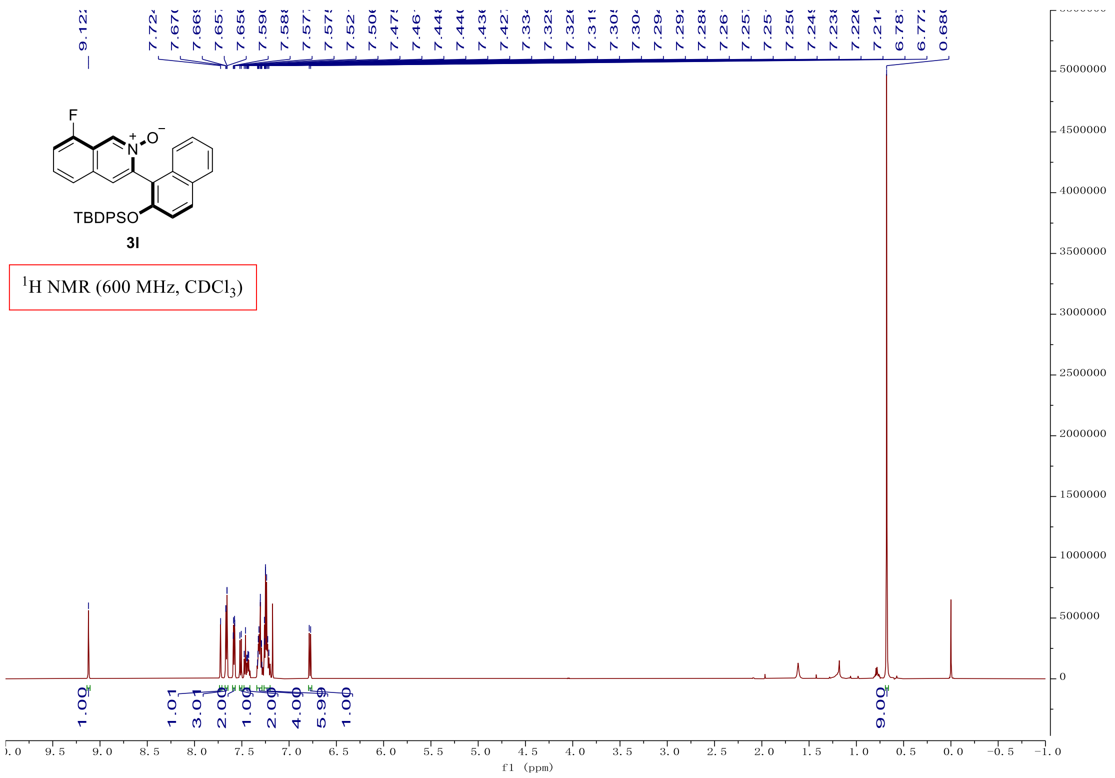
F
TBDPSO
3l
^{1}H NMR (600 MHz, $CDCl_3$)
f1 (ppm)

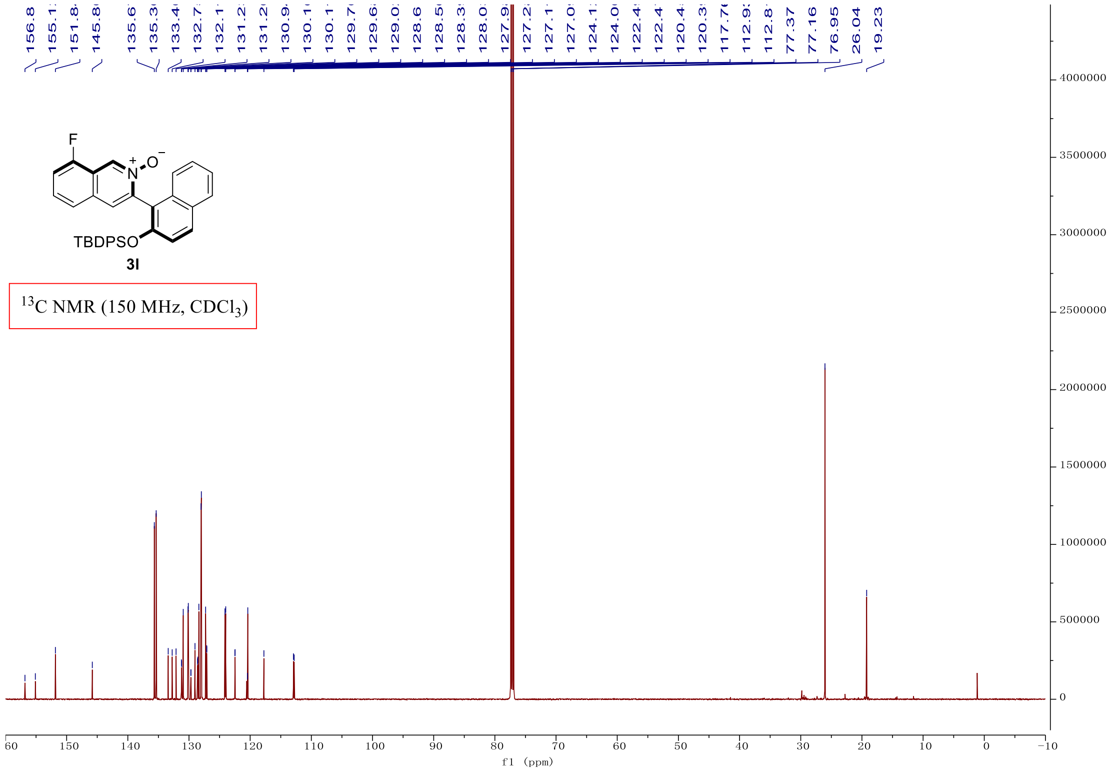
F
TBDPSO
3l
^{13}C NMR (150 MHz, $CDCl_3$)
f1 (ppm)

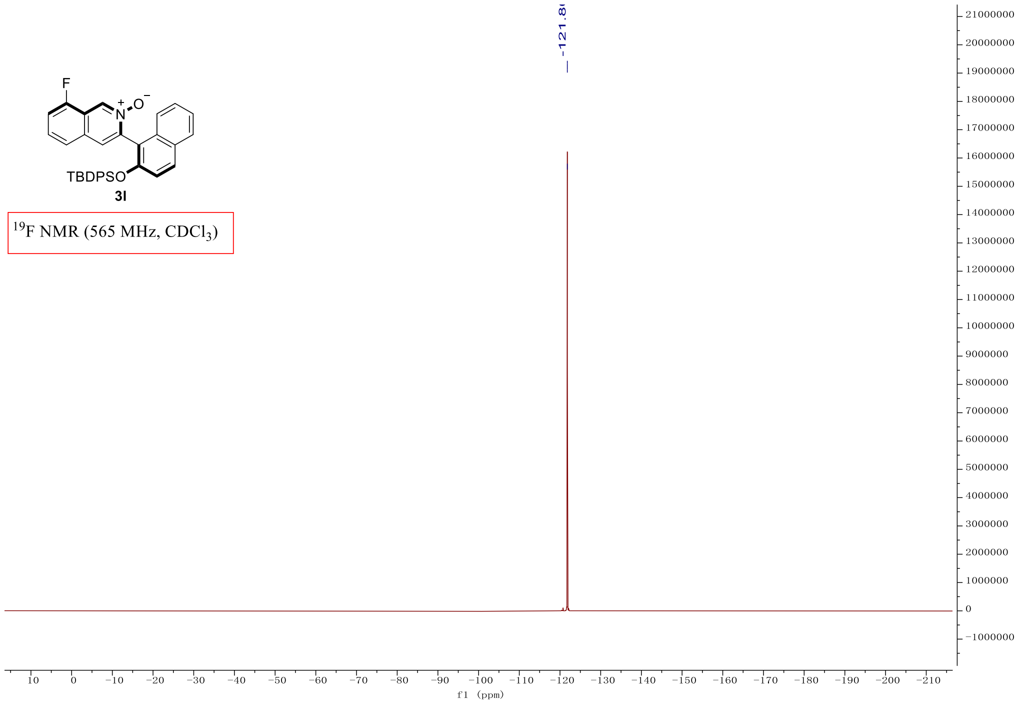

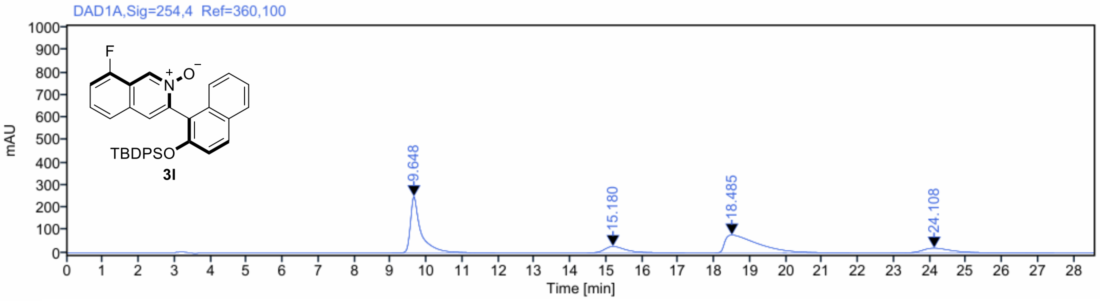

Signal: DAD1A,Sig=254,4 Ref=360,100

RT [min]	Type	Width [min]	Area	Height	Area%	Name
9.648	MM m	2.85	5049.72	249.35	40.91	
15.180	MM m	2.69	1153.47	28.03	9.34	
18.485	MM m	3.74	4974.21	78.59	40.30	
24.108	MM m	4.53	1166.40	20.75	9.45	
		**Sum**	**12343.80**			

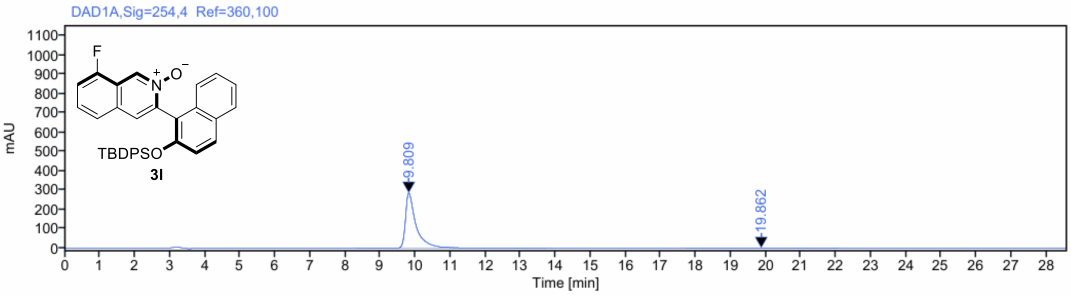

Signal: DAD1A,Sig=254,4 Ref=360,100

RT [min]	Type	Width [min]	Area	Height	Area%	Name
9.809	MM m	5.41	5998.13	286.59	99.68	
19.862	MM m	4.34	19.25	0.47	0.32	
		**Sum**	**6017.39**			

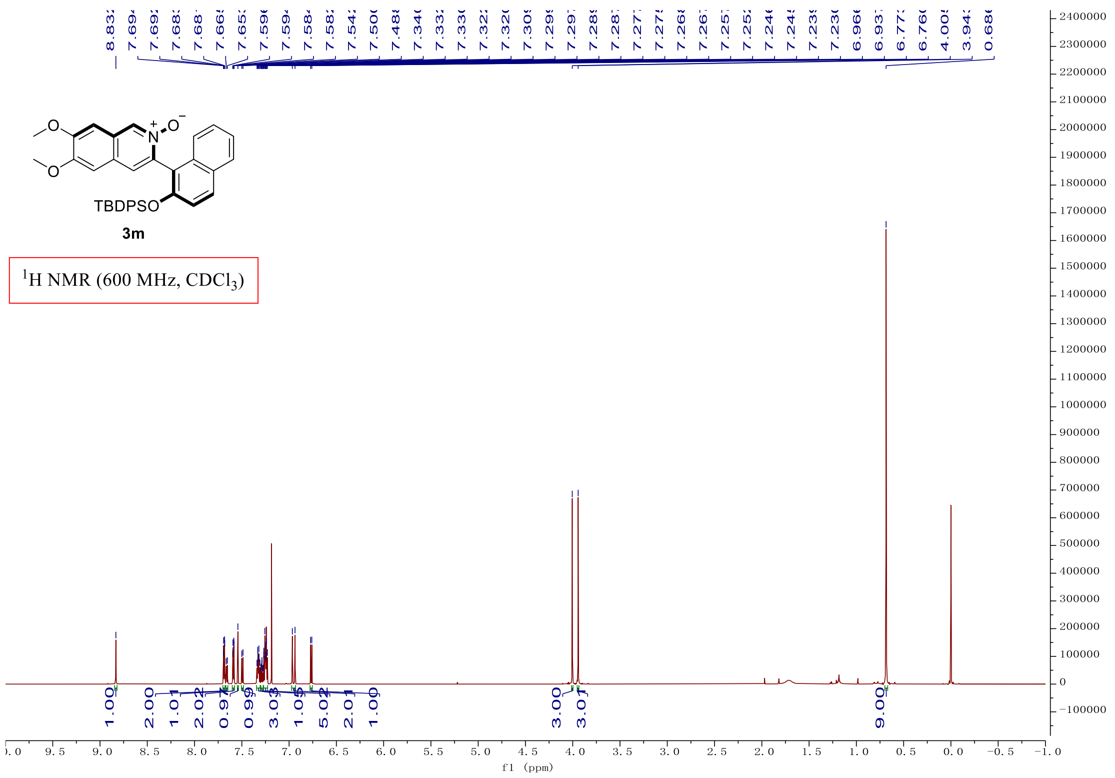
3m
^{1}H NMR (600 MHz, $CDCl_3$)
f1 (ppm)

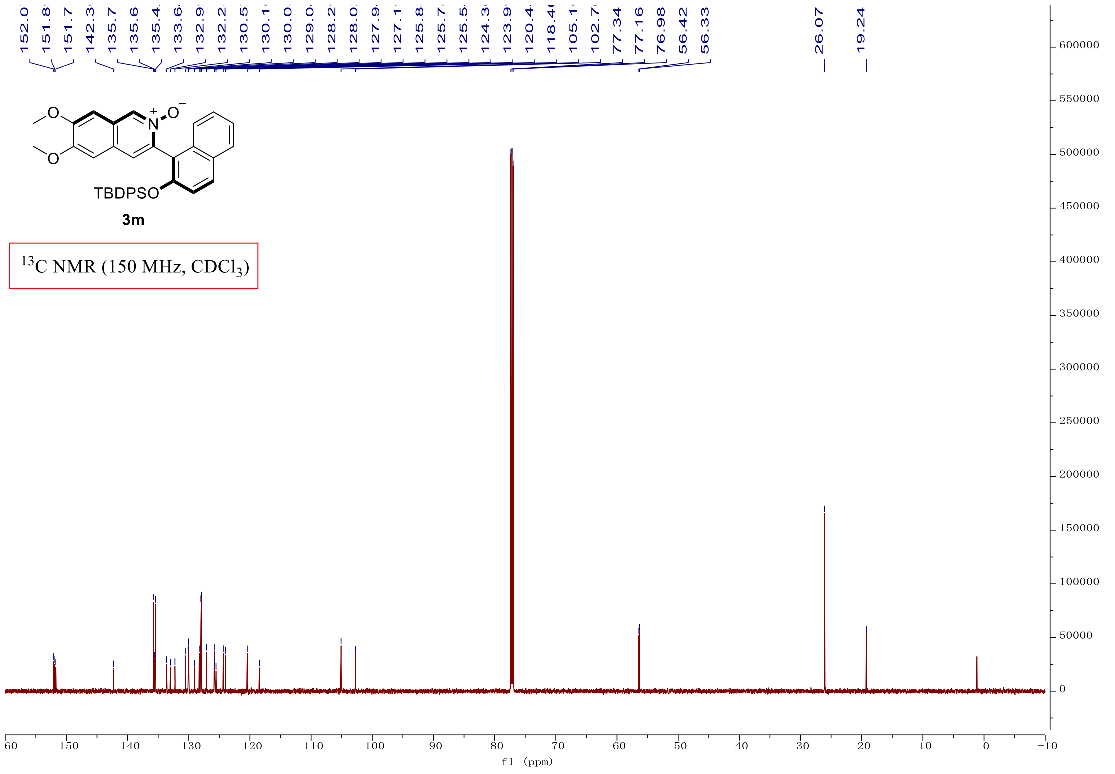
3m
^{13}C NMR (150 MHz, $CDCl_3$)
f1 (ppm)

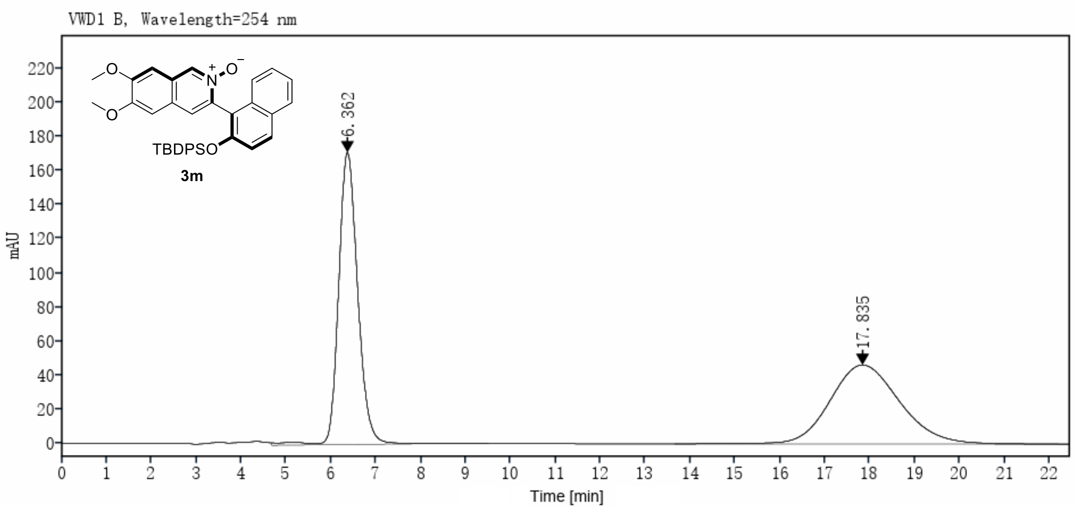

Signal: VWD1 B, Wavelength=254 nm

RT [min]Type	Area	Width[min]	Area%
6.362	5079.0068	0.445	50.1846
17.835	5041.6440	1.647	49.8154
Sum	10120.6509		

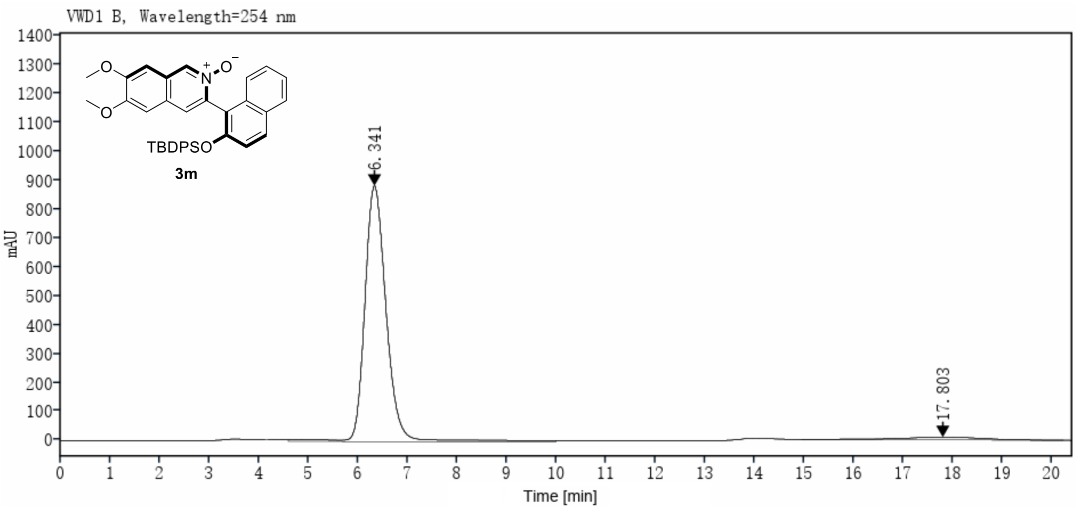

Signal: VWD1 B, Wavelength=254 nm

RT [min]Type	Area	Width[min]	Area%
6.341	27085.6680	0.443	96.1412
17.803	1087.1370	1.667	3.8588
Sum	28172.8049		

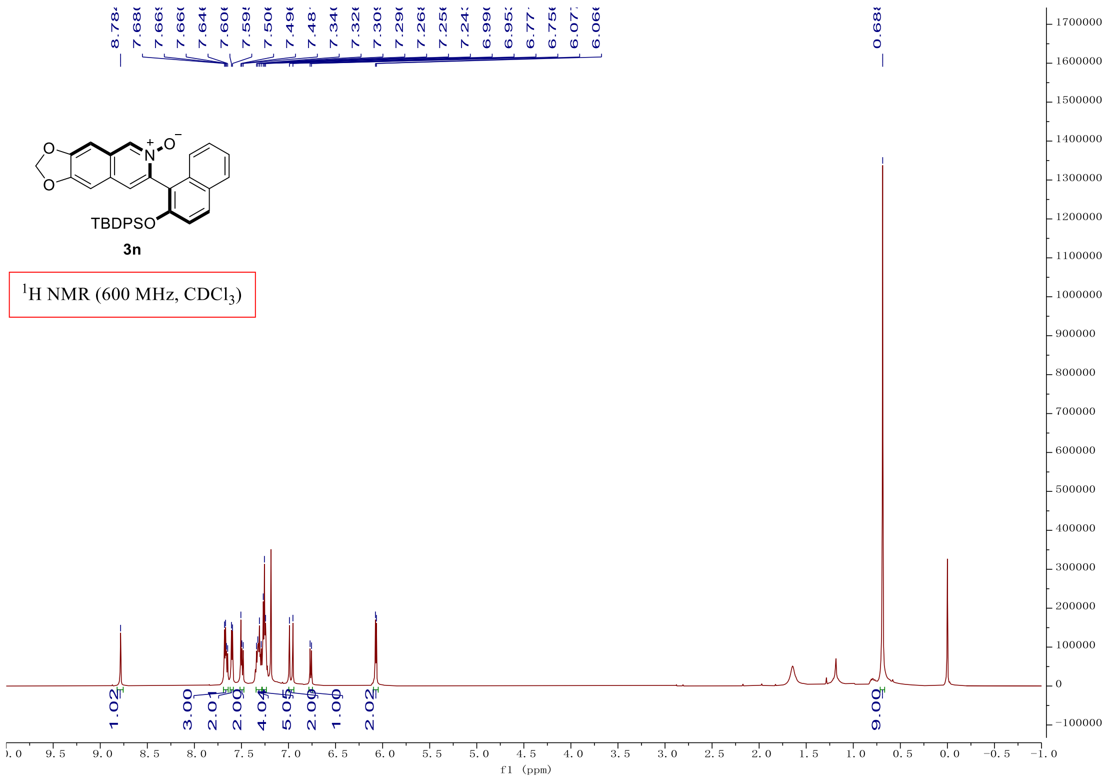
3n
TBDPSO
1H NMR (600 MHz, CDCl3)
f1 (ppm)

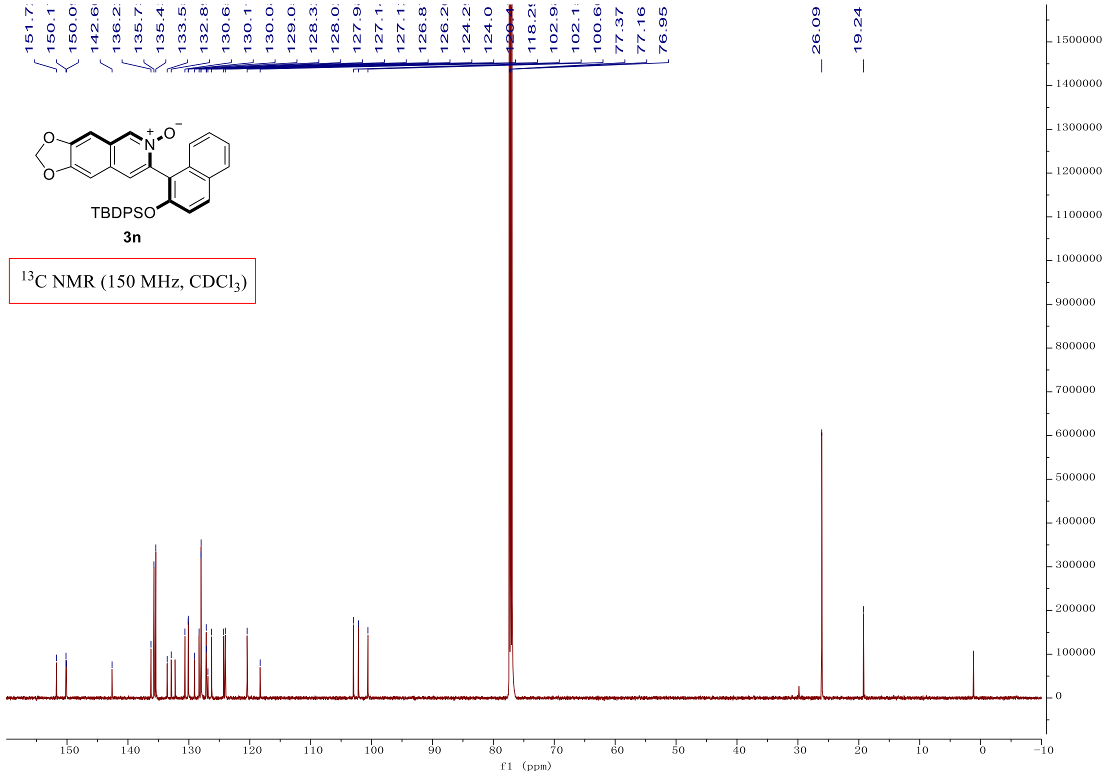
3n
TBDPSO
13C NMR (150 MHz, CDCl3)
f1 (ppm)

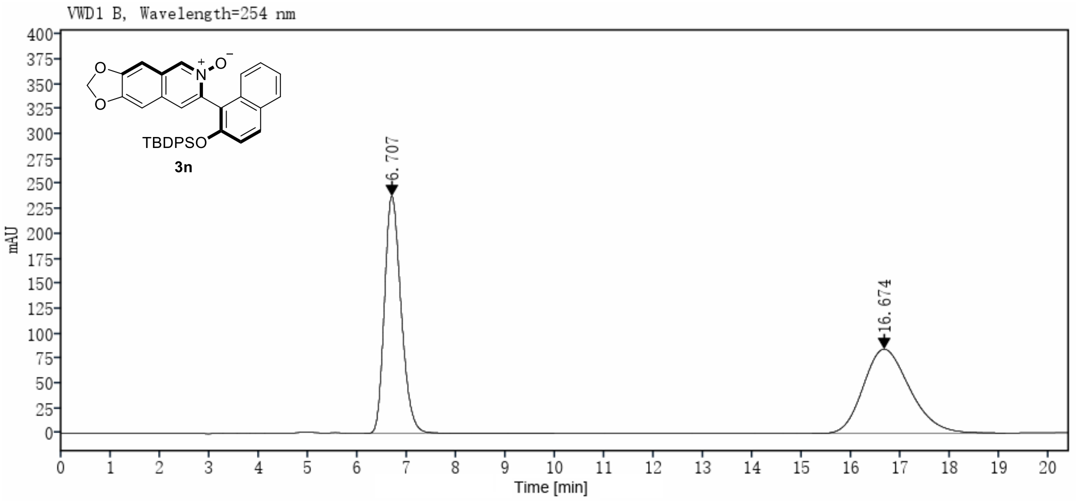

Signal: VWD1 B, Wavelength=254 nm

RT [min]Type	Area	Width[min]	Area%
6.707	5579.5483	0.360	50.3445
16.674	5503.1870	1.007	49.6555
Sum	11082.7354		

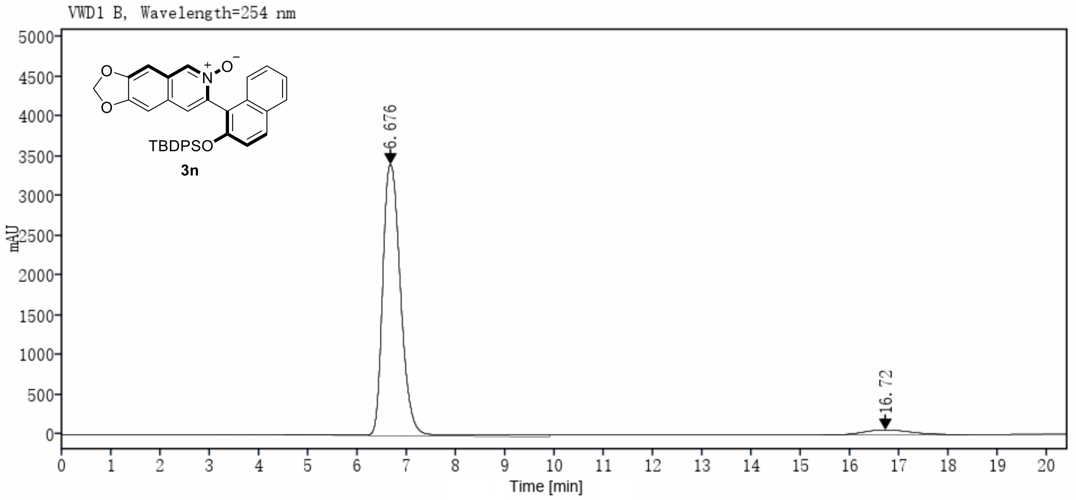

Signal: VWD1 B, Wavelength=254 nm

RT [min]Type	Area	Width[min]	Area%
6.676	88093.1406	0.384	95.6058
16.720	4048.9492	1.047	4.3942
Sum	92142.0898		

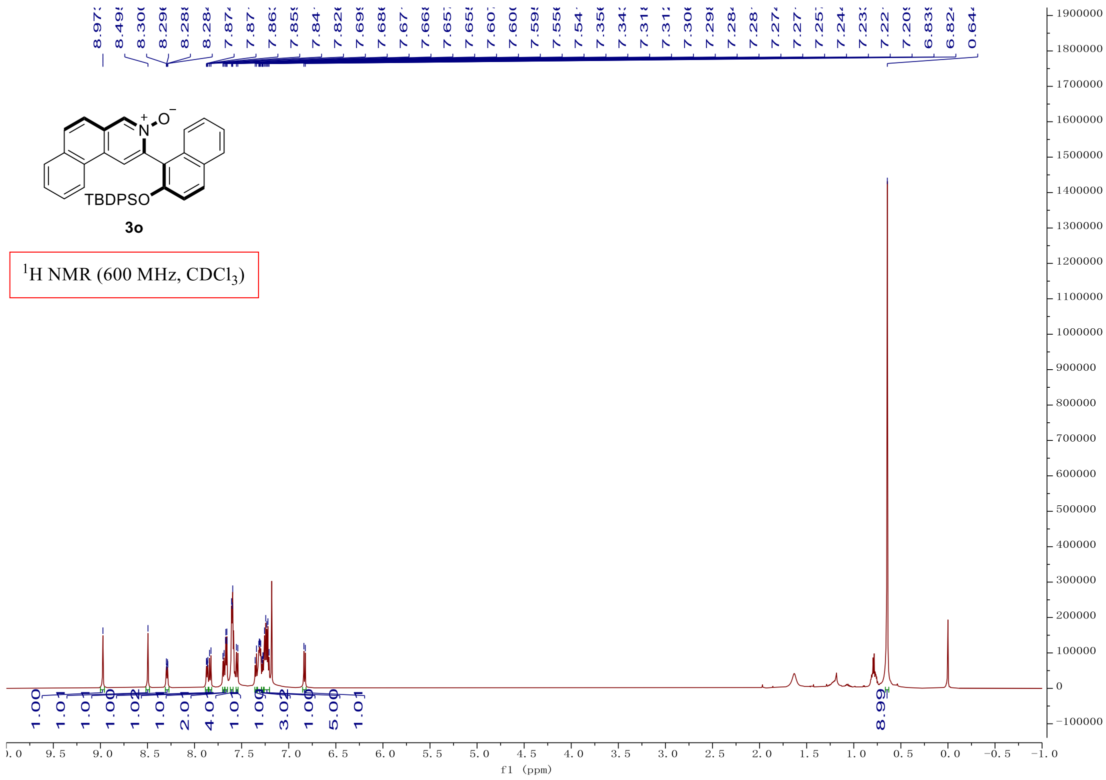
TBDPSO
3o
1H NMR (600 MHz, CDCl3)
f1 (ppm)

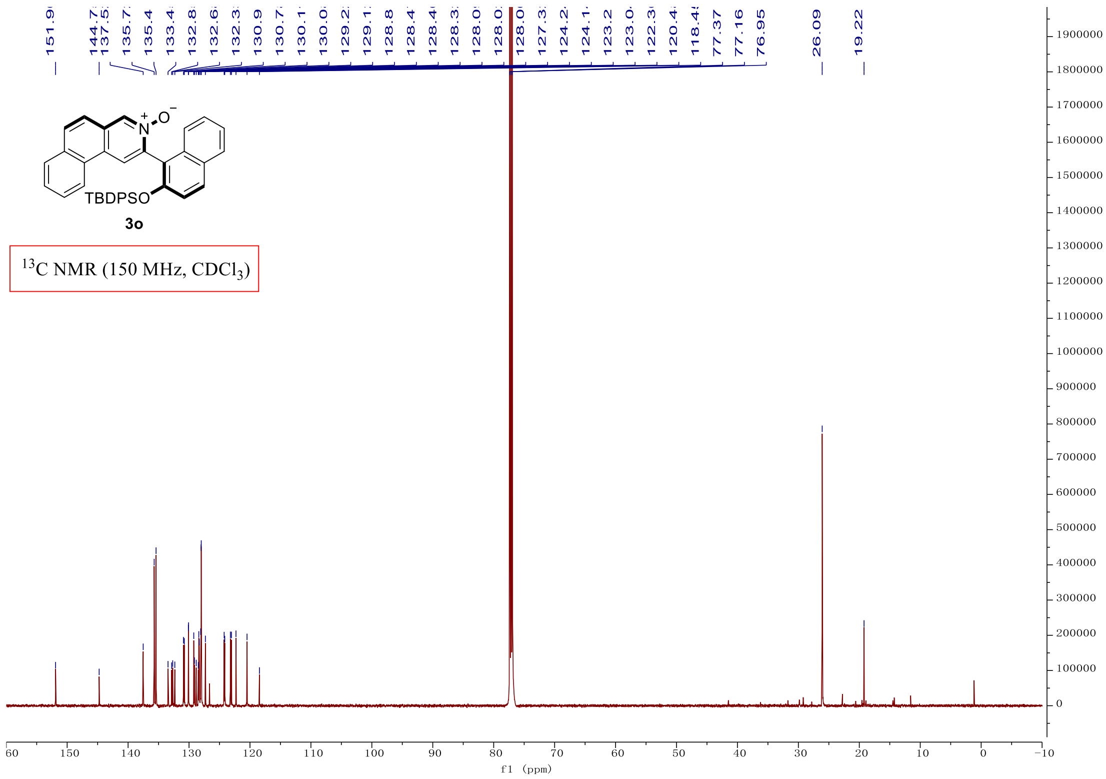
TBDPSO
3o
13C NMR (150 MHz, CDCl3)
f1 (ppm)

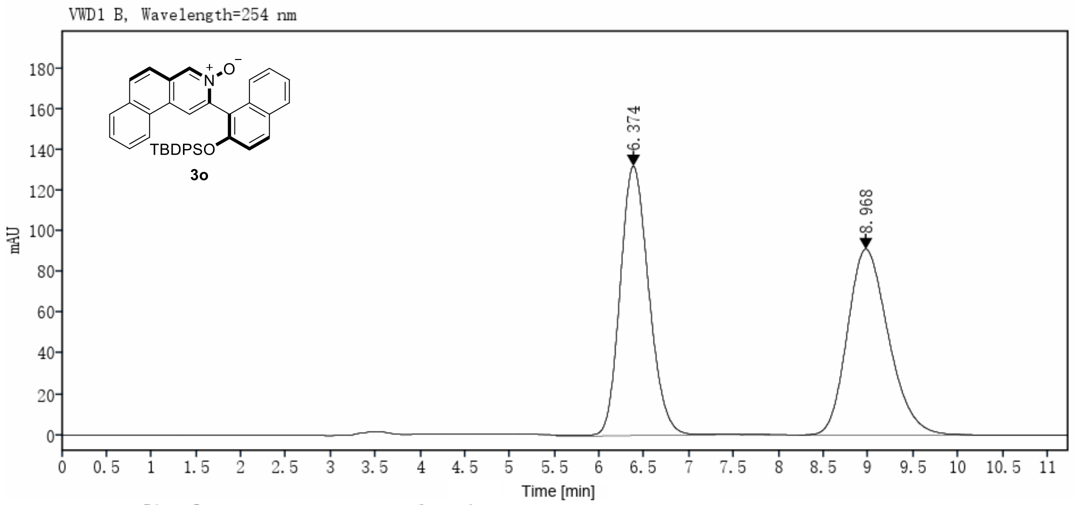

Signal: VWD1 B, Wavelength=254 nm

RT [min]Type	Area	Width[min]	Area%
6.374	2948.2068	0.342	50.3597
8.968	2906.0911	0.490	49.6403
Sum	5854.2979		

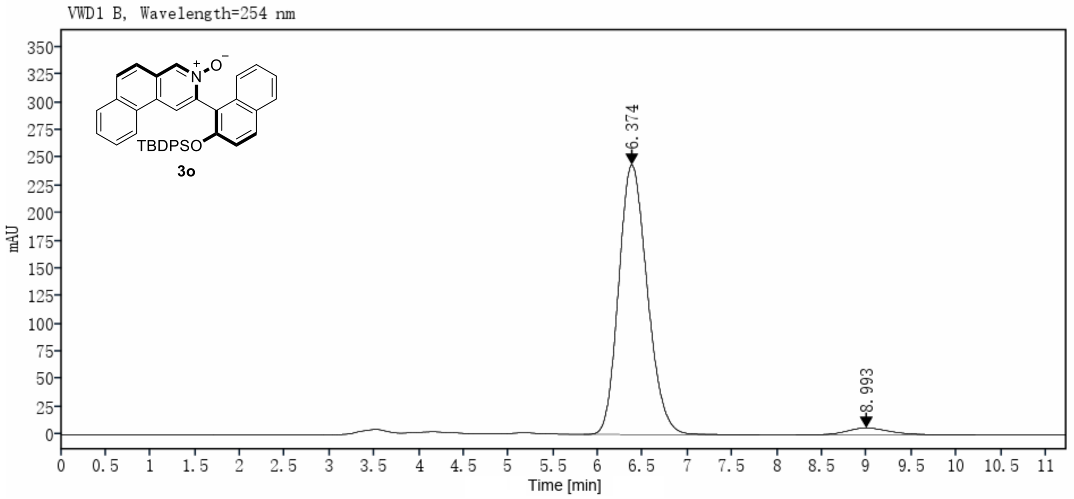

Signal: VWD1 B, Wavelength=254 nm

RT [min]Type	Area	Width[min]	Area%
6.374	5402.3247	0.344	96.5277
8.993	194.3347	0.496	3.4723
Sum	5596.6594		

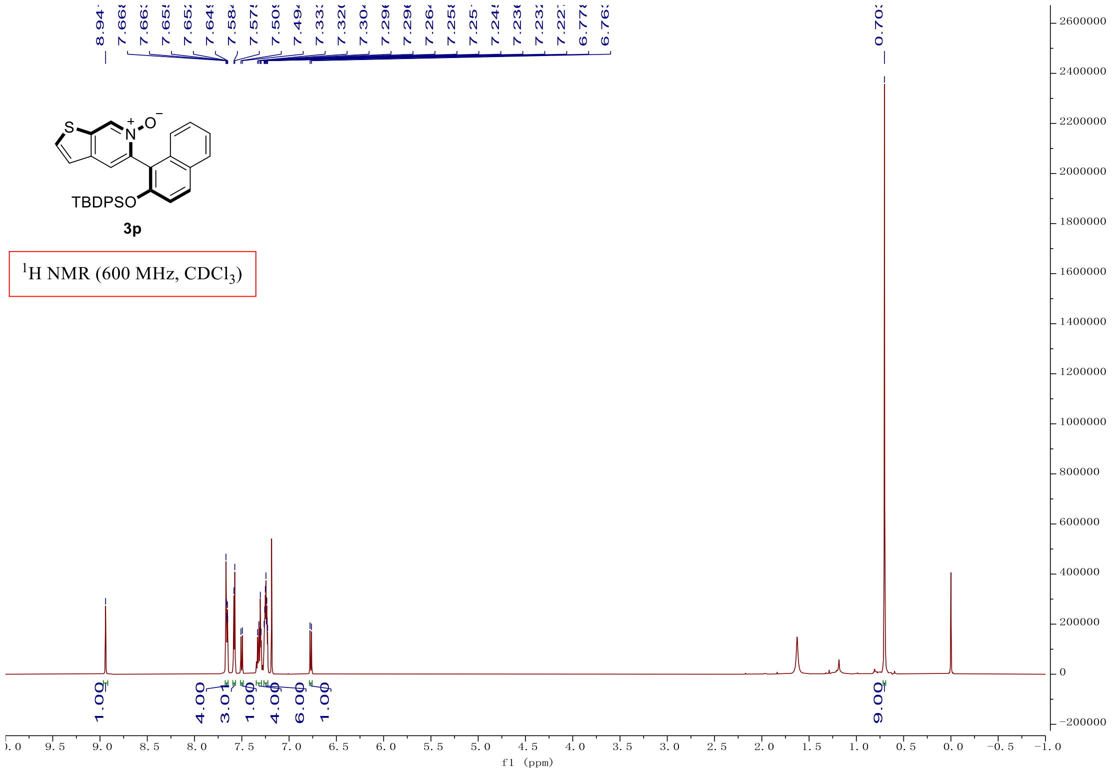

3p

^1H NMR (600 MHz, $CDCl_3$)

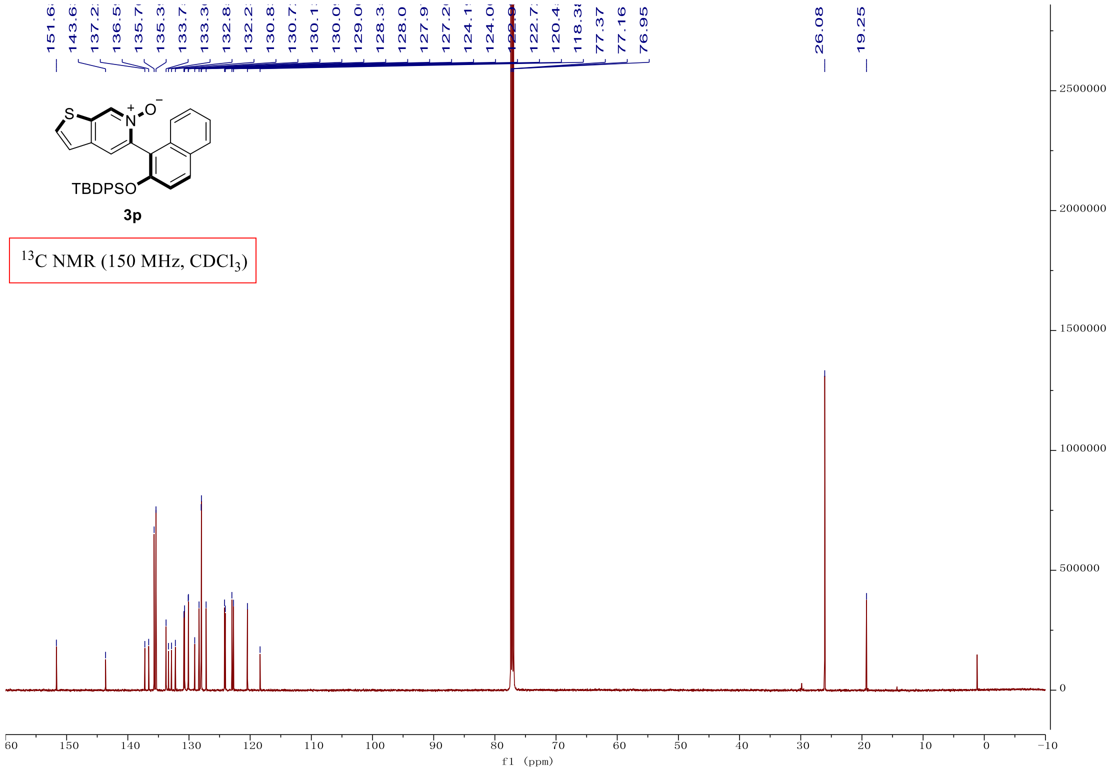

3p

^{13}C NMR (150 MHz, $CDCl_3$)

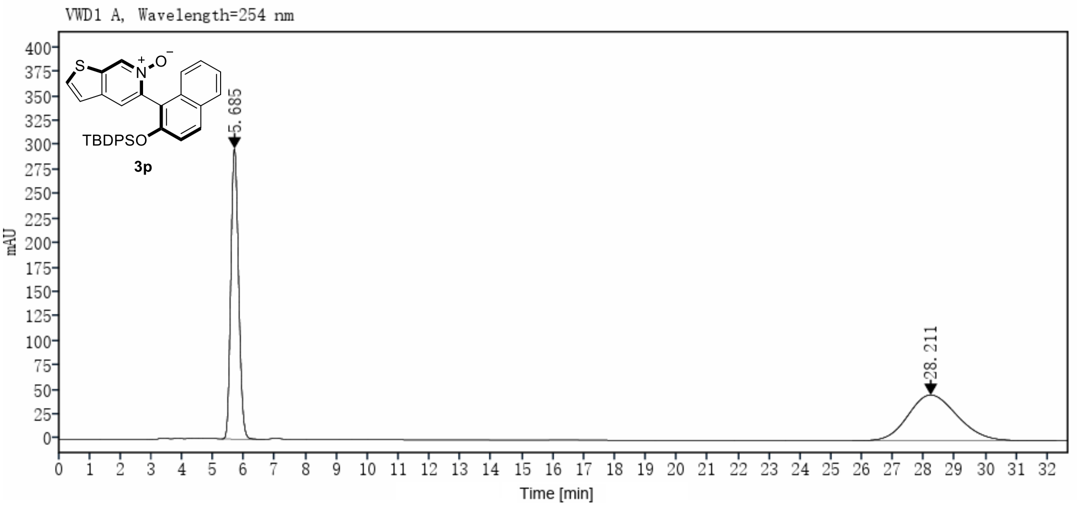

Signal: VWD1 A, Wavelength=254 nm

RT [min]Type	Area	Width[min]	Area%
5.685	5167.2202	0.273	50.1372
28.211	5138.9375	1.713	49.8628
Sum	10306.1577		

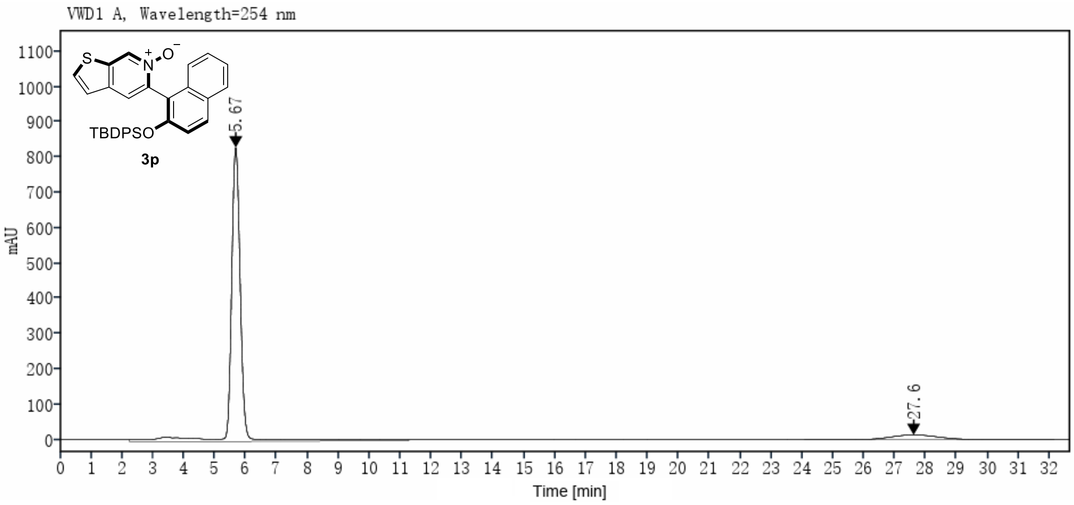

Signal: VWD1 A, Wavelength=254 nm

RT [min]Type	Area	Width[min]	Area%
5.670	18365.2988	0.291	93.1028
27.600	1360.5200	1.660	6.8972
Sum	19725.8188		

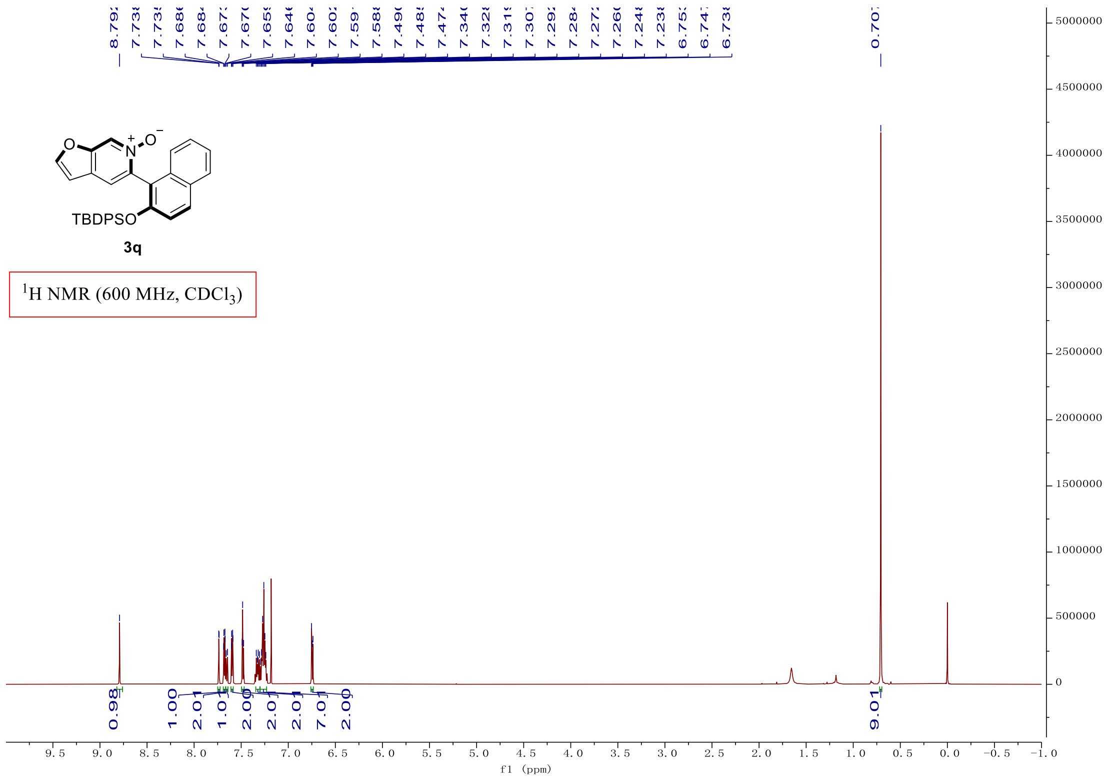

^{1}H NMR (600 MHz, $CDCl_3$)

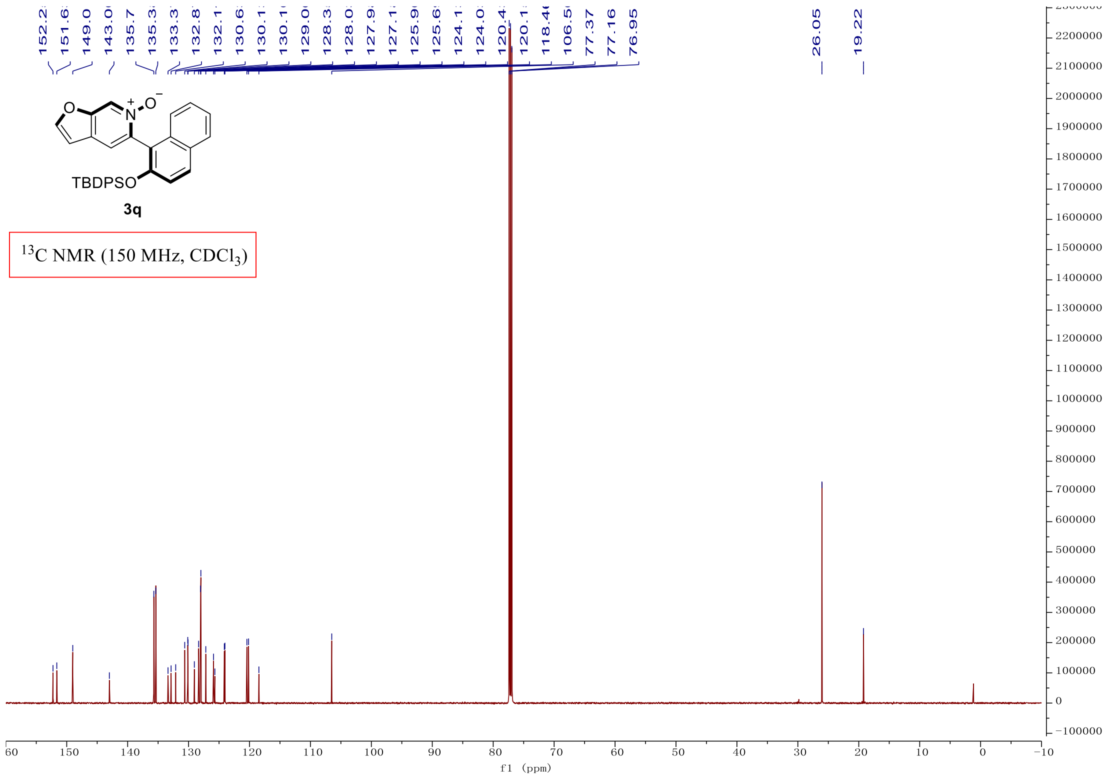

^{13}C NMR (150 MHz, $CDCl_3$)

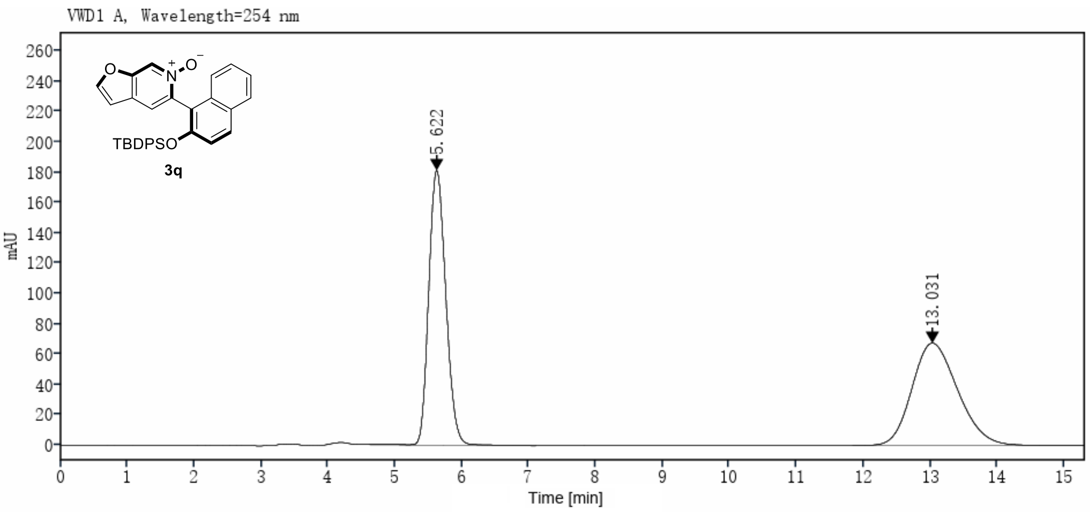

Signal: VWD1 A, Wavelength=254 nm

RT [min]Type	Area	Width[min]	Area%
5.622	3227.2986	0.277	50.2839
13.031	3190.8584	0.737	49.7161
Sum	6418.1570		

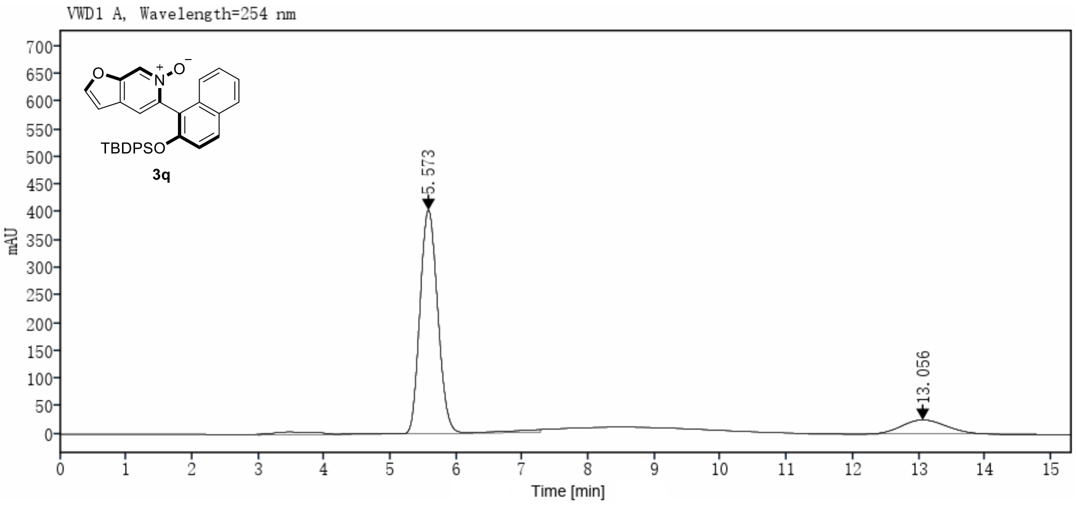

Signal: VWD1 A, Wavelength=254 nm

RT [min]Type	Area	Width[min]	Area%
5.573	7977.2041	0.295	86.9695
13.056	1195.2141	0.747	13.0305
Sum	9172.4182		

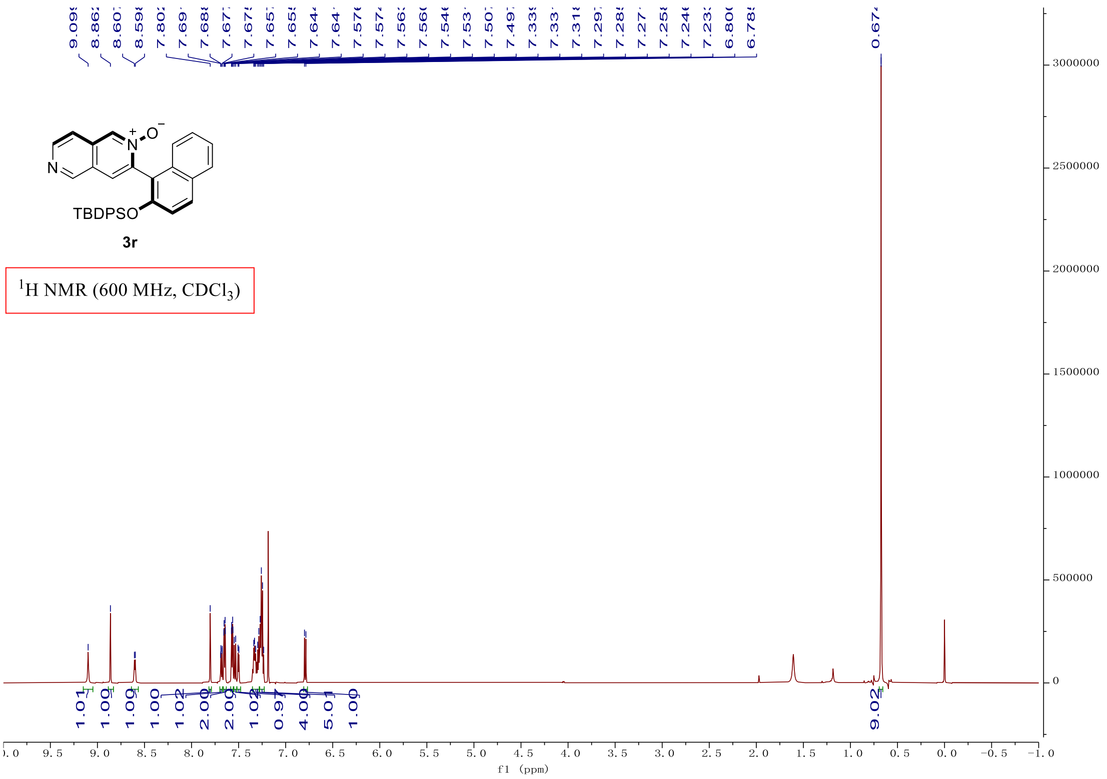
TBDPSO
3r
1H NMR (600 MHz, CDCl3)
f1 (ppm)

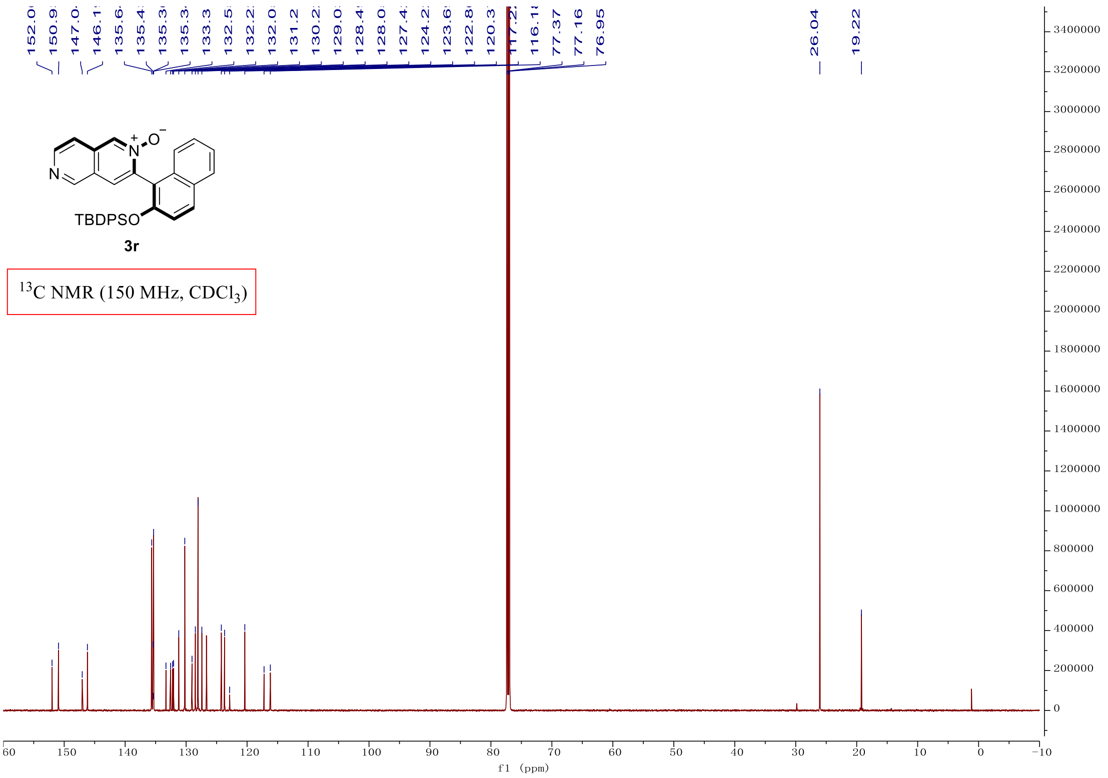
TBDPSO
3r
13C NMR (150 MHz, CDCl3)
f1 (ppm)

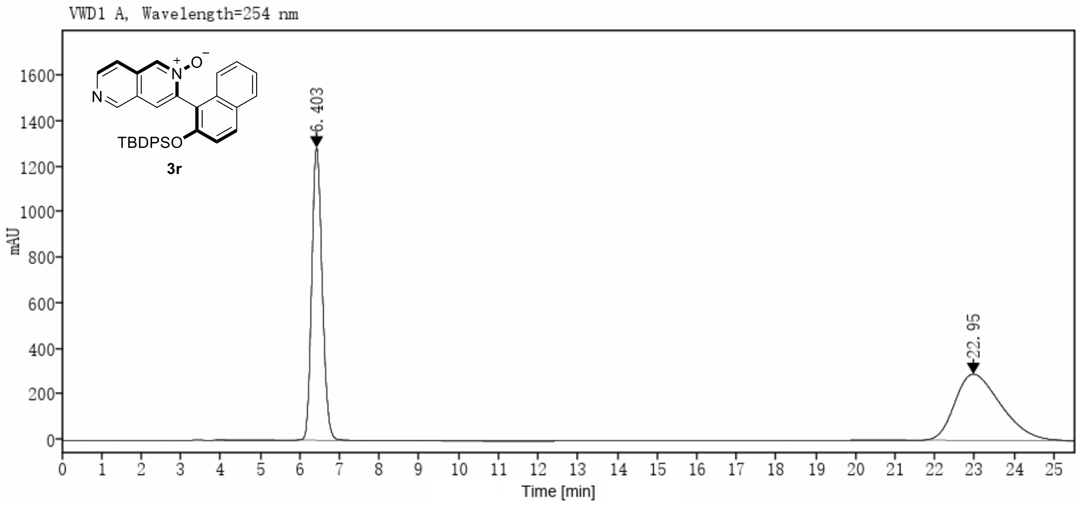

Signal: VWD1 A, Wavelength=254 nm

RT [min]Type	Area	Width[min]	Area%
6.403	24284.0723	0.284	50.4073
22.950	23891.6191	1.248	49.5927
Sum	48175.6914		

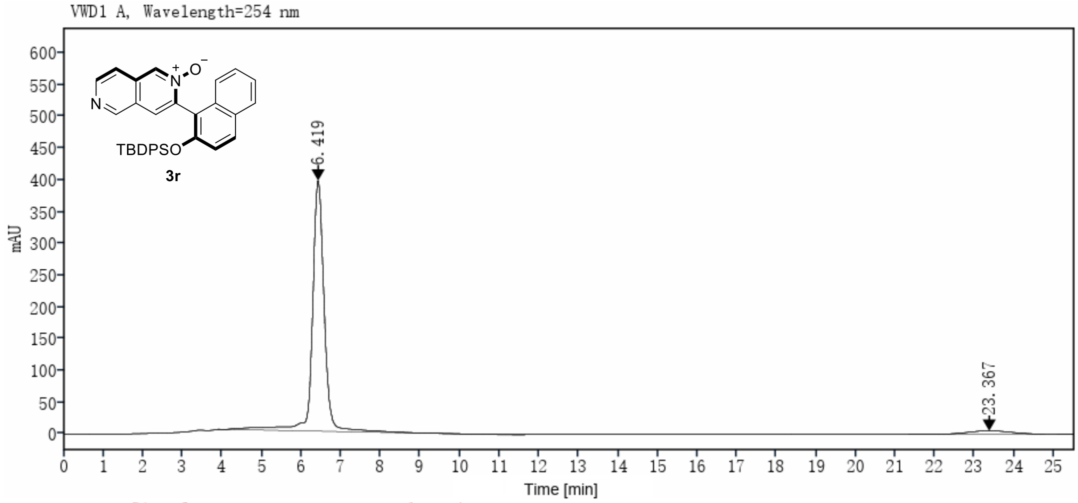

Signal: VWD1 A, Wavelength=254 nm

RT [min]Type	Area	Width[min]	Area%
6.419	8385.8340	0.292	94.9542
23.367	445.6133	1.227	5.0458
Sum	8831.4473		

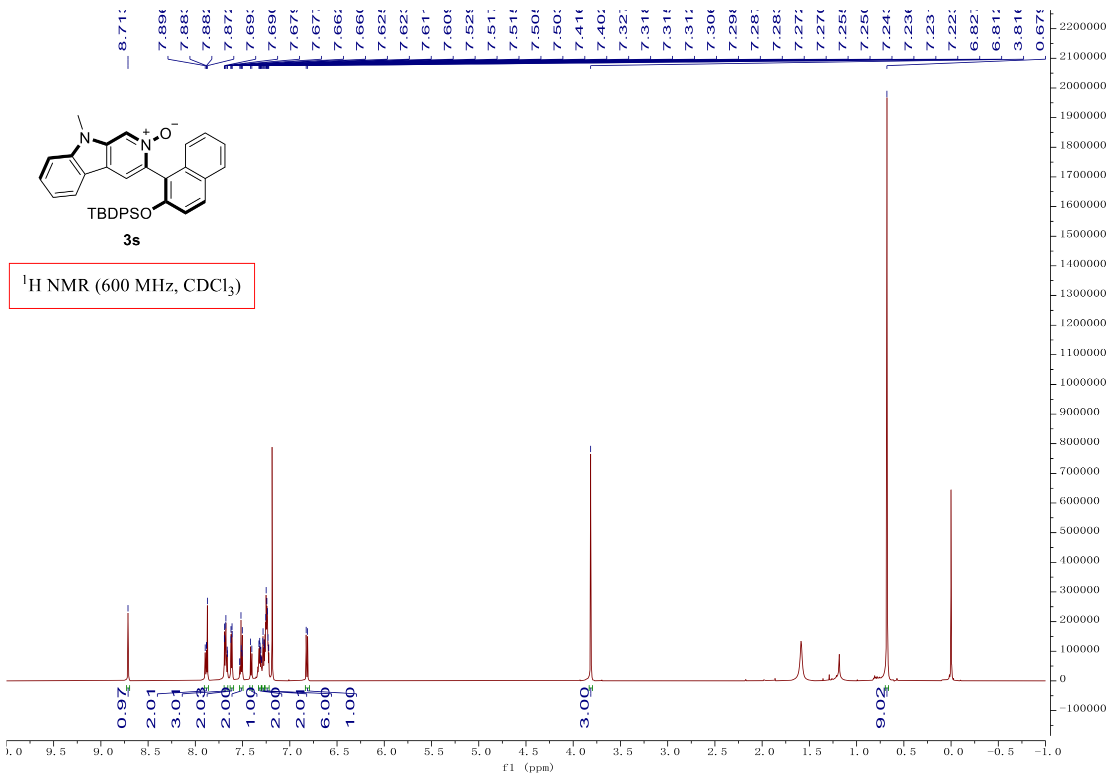

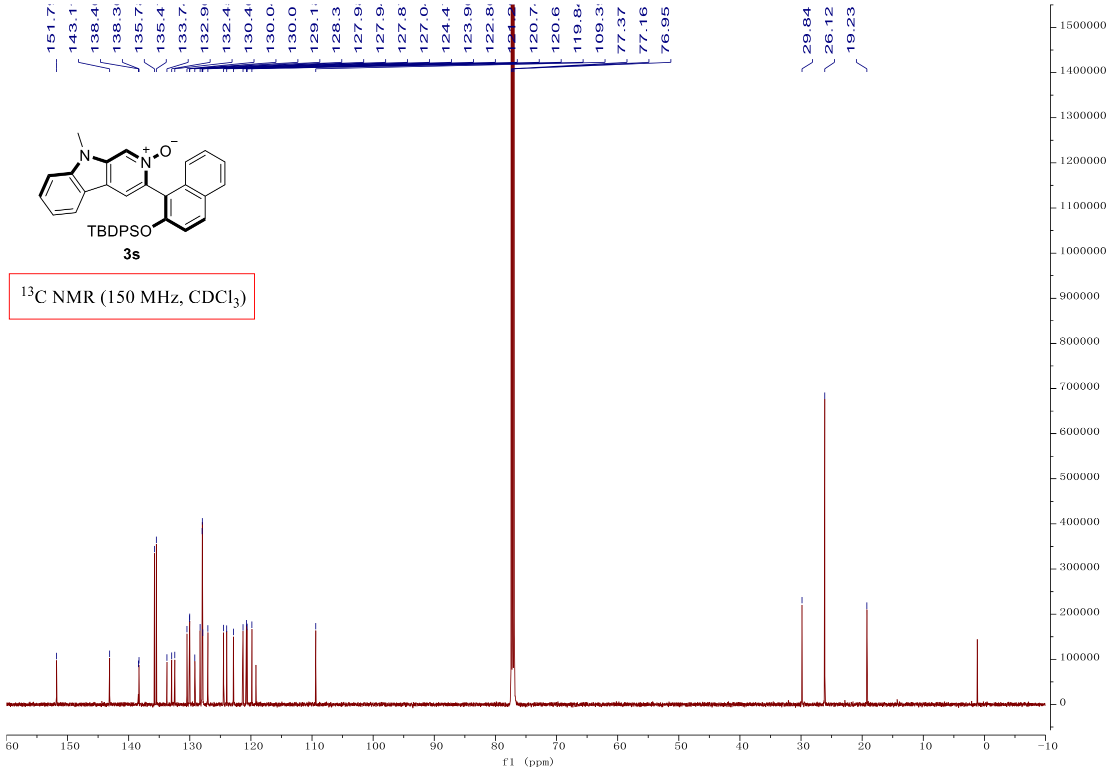

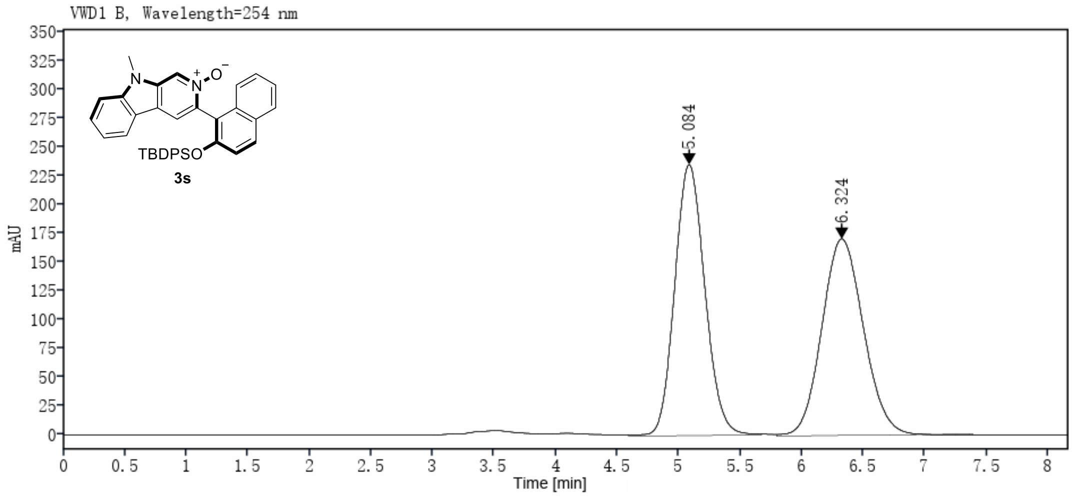

Signal: VWD1 B, Wavelength=254 nm

RT [min]Type	Area	Width[min]	Area%
5.084	4026.0115	0.264	50.1324
6.324	4004.7515	0.363	49.8676
Sum	8030.7629		

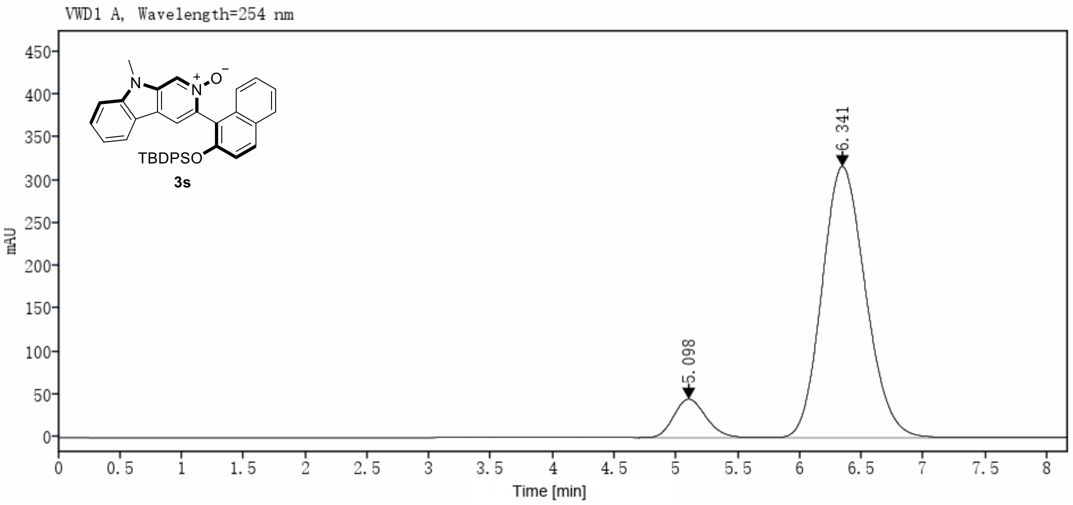

Signal: VWD1 A, Wavelength=254 nm

RT [min]Type	Area	Width[min]	Area%
5.098	815.9349	0.281	9.5326
6.341	7743.4570	0.380	90.4674
Sum	8559.3920		

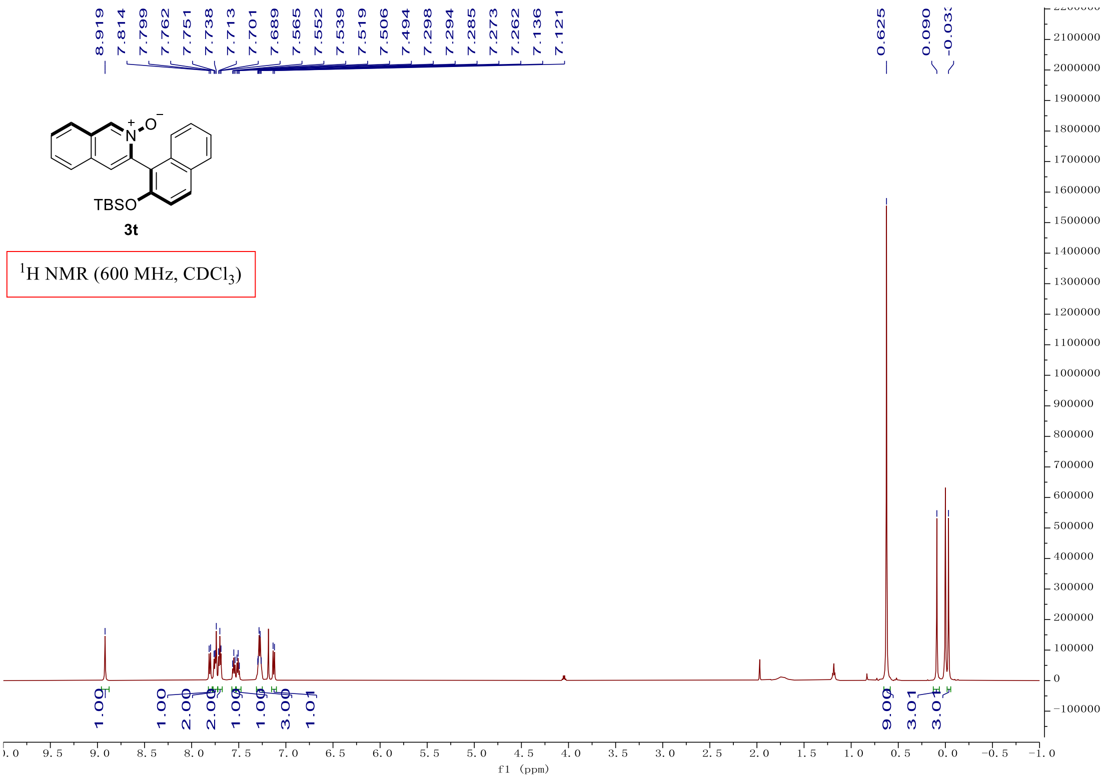

3t

^{1}H NMR (600 MHz, $CDCl_3$)

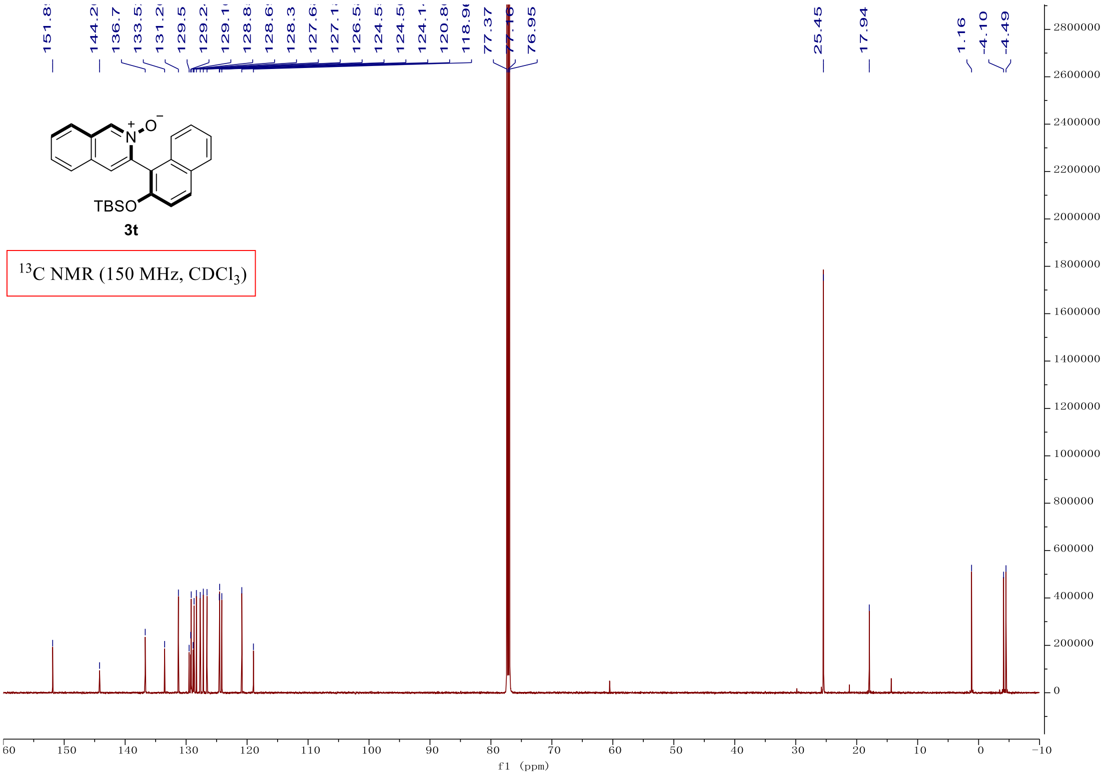

3t

^{13}C NMR (150 MHz, $CDCl_3$)

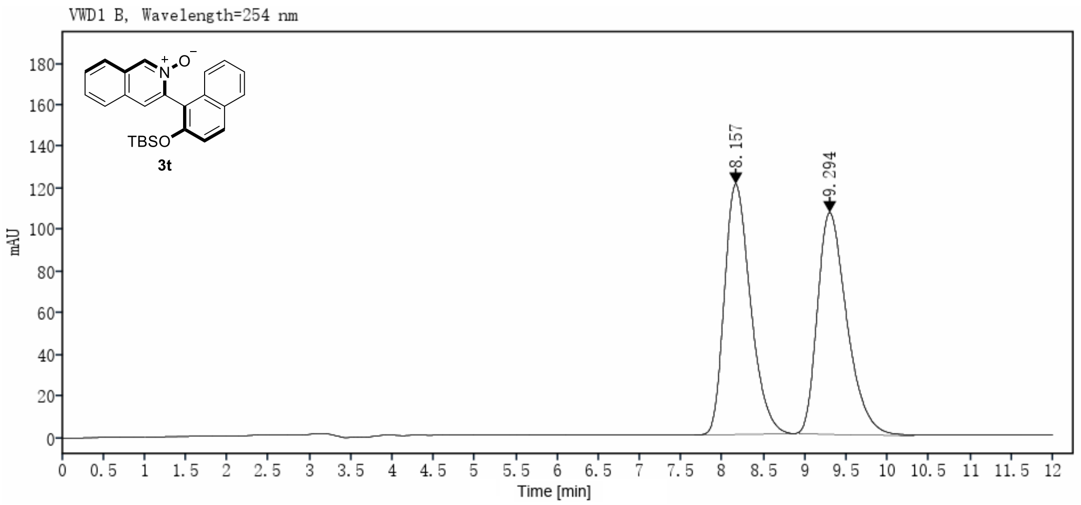

**Signal:**	VWD1 B, Wavelength=254 nm		
RT [min]Type	Area	Width[min]	Area%
8.157	2640.8271	0.340	49.9194
9.294	2649.3577	0.383	50.0806
Sum	5290.1848		

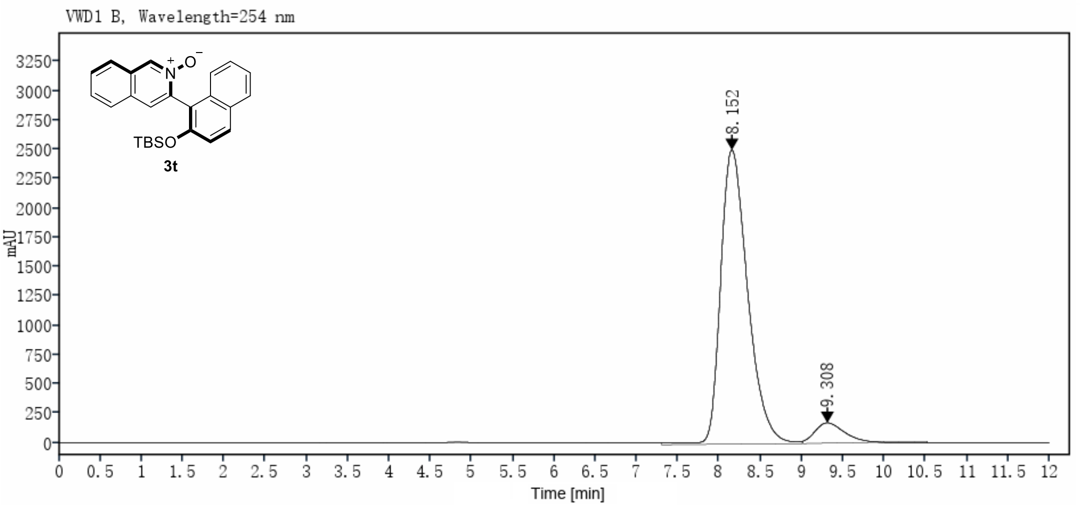

**Signal:**	VWD1 B, Wavelength=254 nm		
RT [min]Type	Area	Width[min]	Area%
8.152	57378.7539	0.344	93.0486
9.308	4286.5781	0.387	6.9514
Sum	61665.3320		

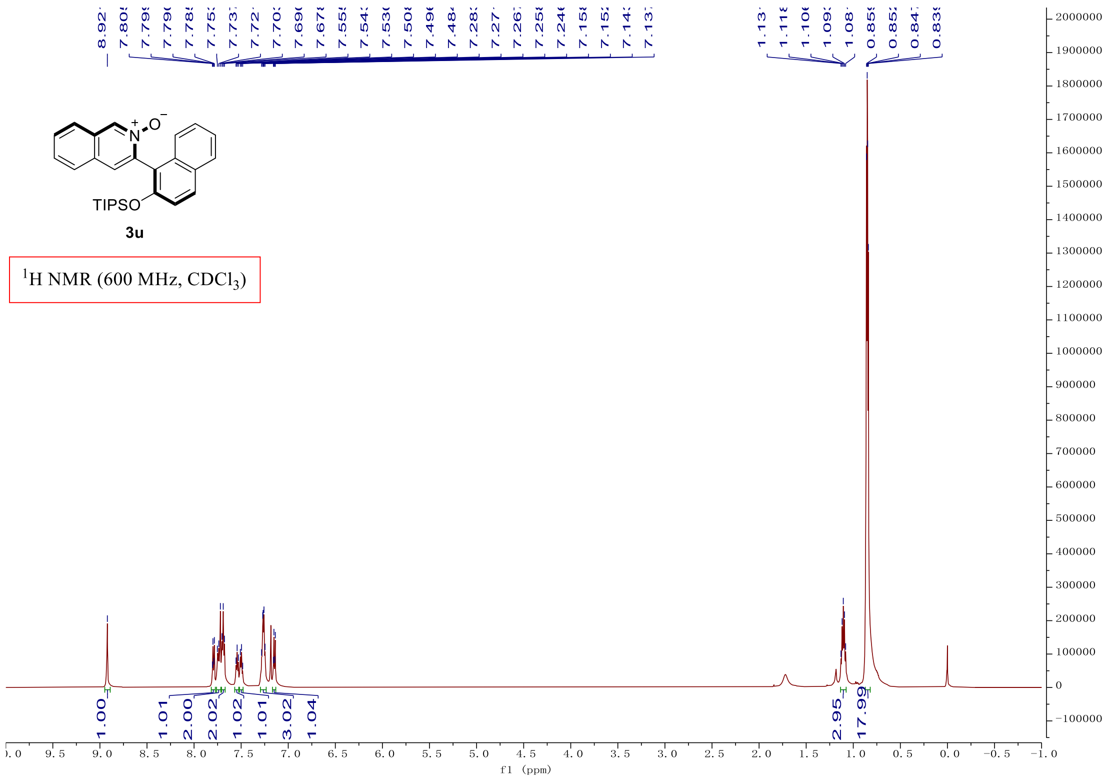

3u

^{1}H NMR (600 MHz, $CDCl_3$)

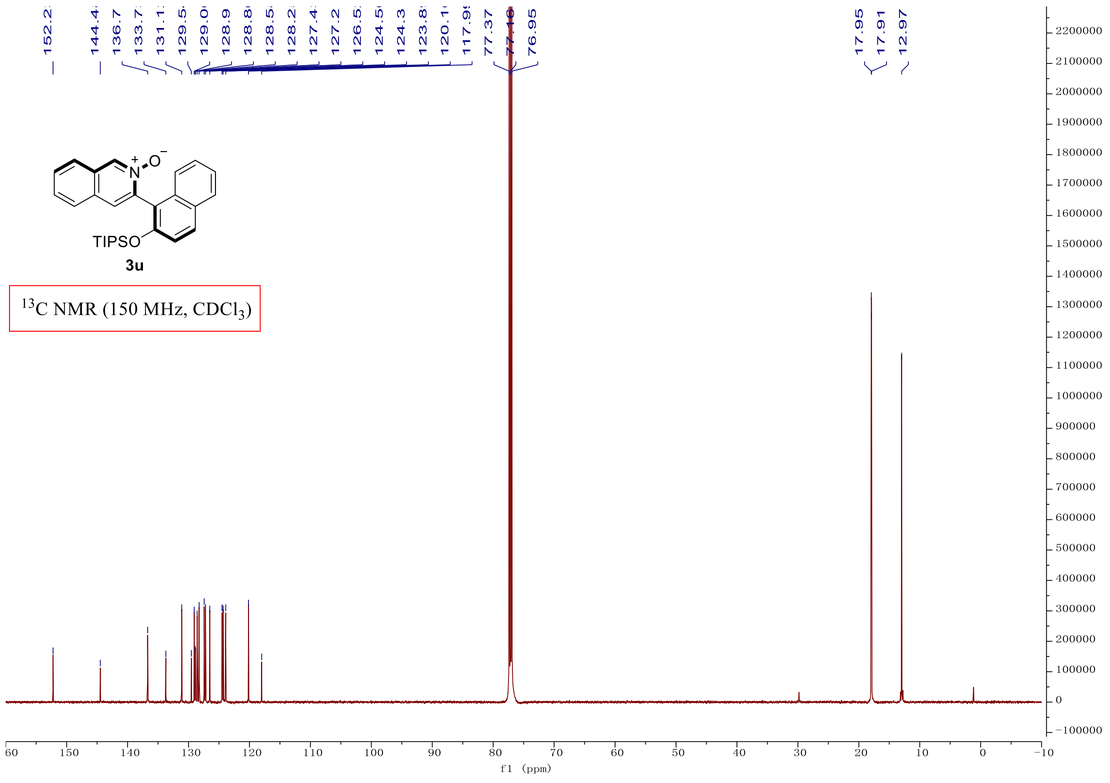

3u

^{13}C NMR (150 MHz, $CDCl_3$)

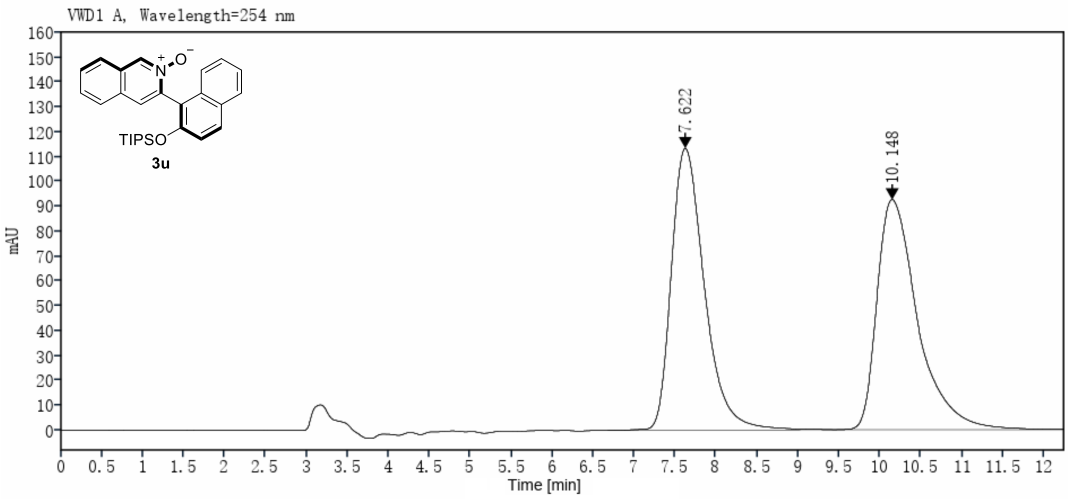

Signal: VWD1 A, Wavelength=254 nm

RT [min]Type	Area	Width[min]	Area%
7.622	3192.2554	0.423	49.9788
10.148	3194.9587	0.502	50.0212
Sum	6387.2141		

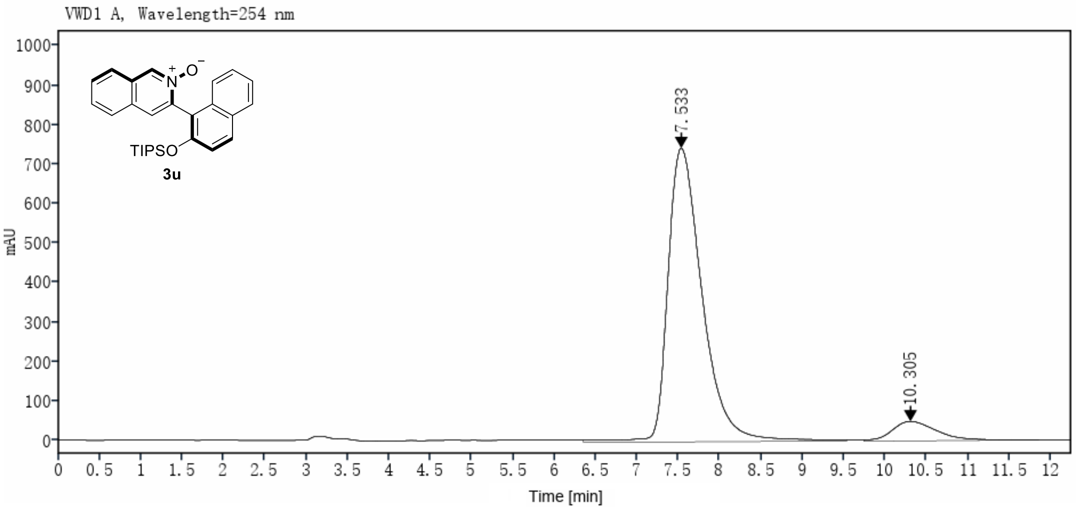

Signal: VWD1 A, Wavelength=254 nm

RT [min]Type	Area	Width[min]	Area%
7.533	21548.5723	0.423	92.0824
10.305	1852.8402	0.563	7.9176
Sum	23401.4125		

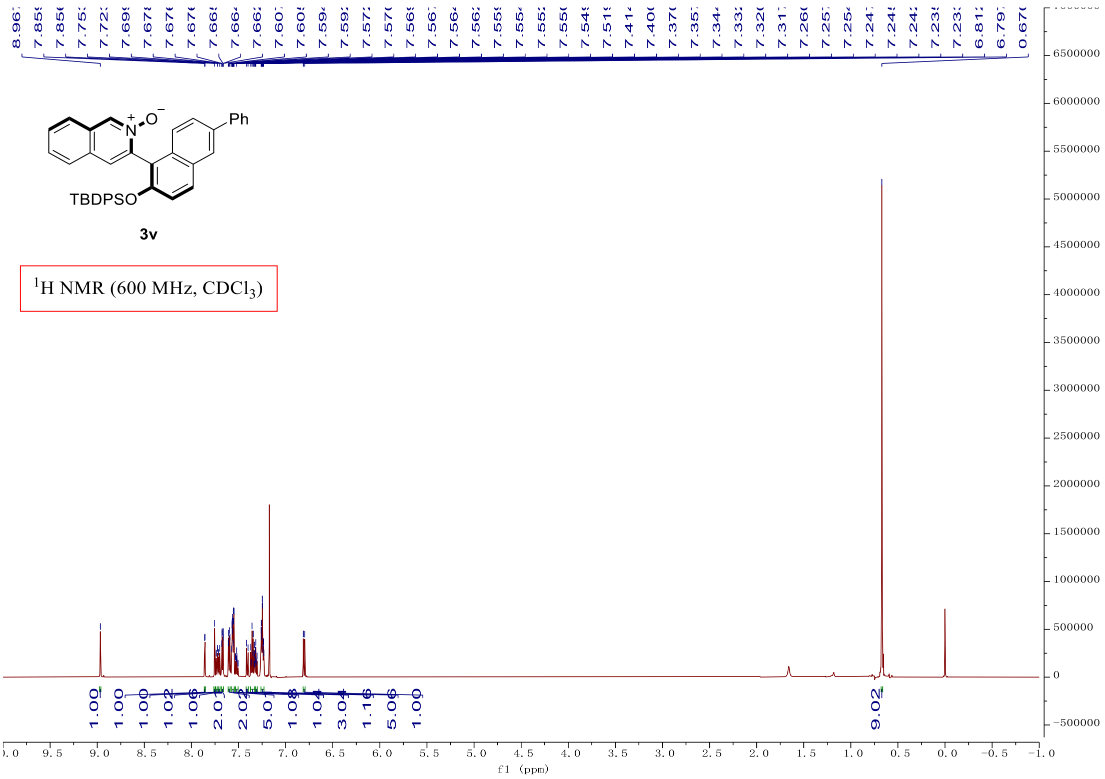

Ph
TBDPSO
3v
1H NMR (600 MHz, CDCl3)
f1 (ppm)

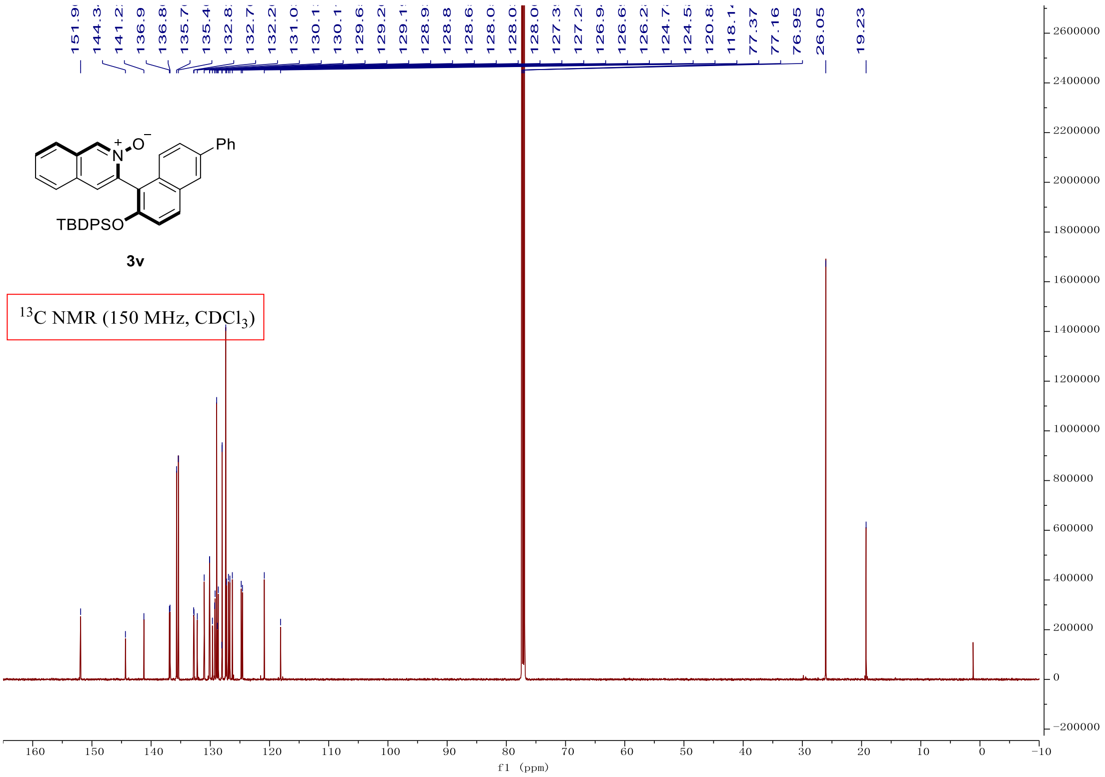

Ph
TBDPSO
3v
13C NMR (150 MHz, CDCl3)
f1 (ppm)

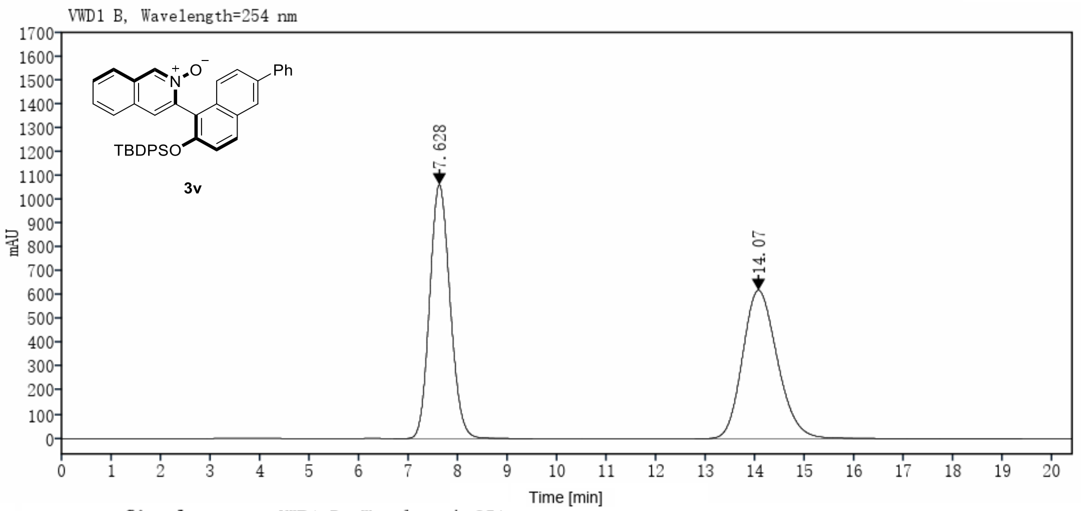

Signal: VWD1 B, Wavelength=254 nm

RT [min]Type	Area	Width[min]	Area%
7.628	30435.4355	0.444	50.0570
14.070	30366.0898	0.752	49.9430
Sum	60801.5254		

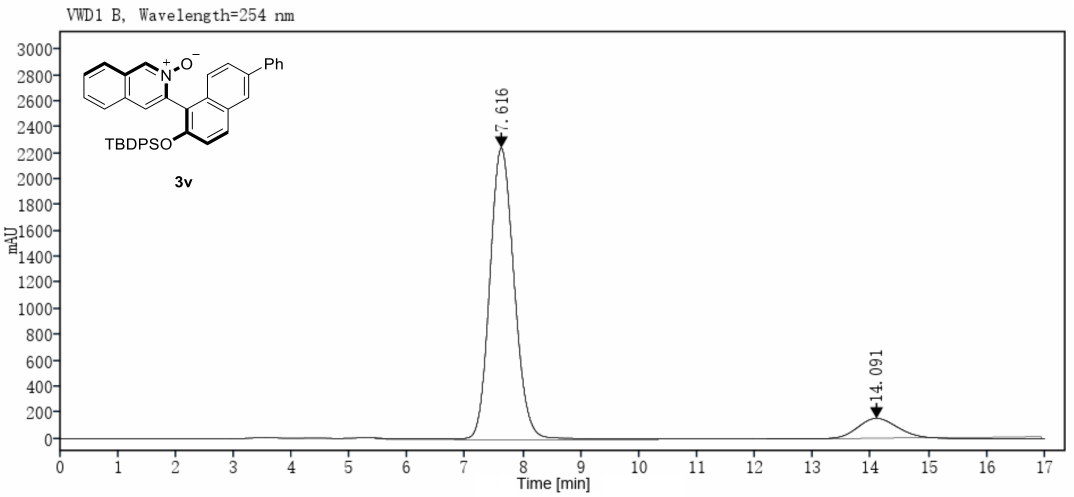

Signal: VWD1 B, Wavelength=254 nm

RT [min]Type	Area	Width[min]	Area%
7.616	67430.2813	0.448	90.2426
14.091	7290.8696	0.747	9.7574
Sum	74721.1509		

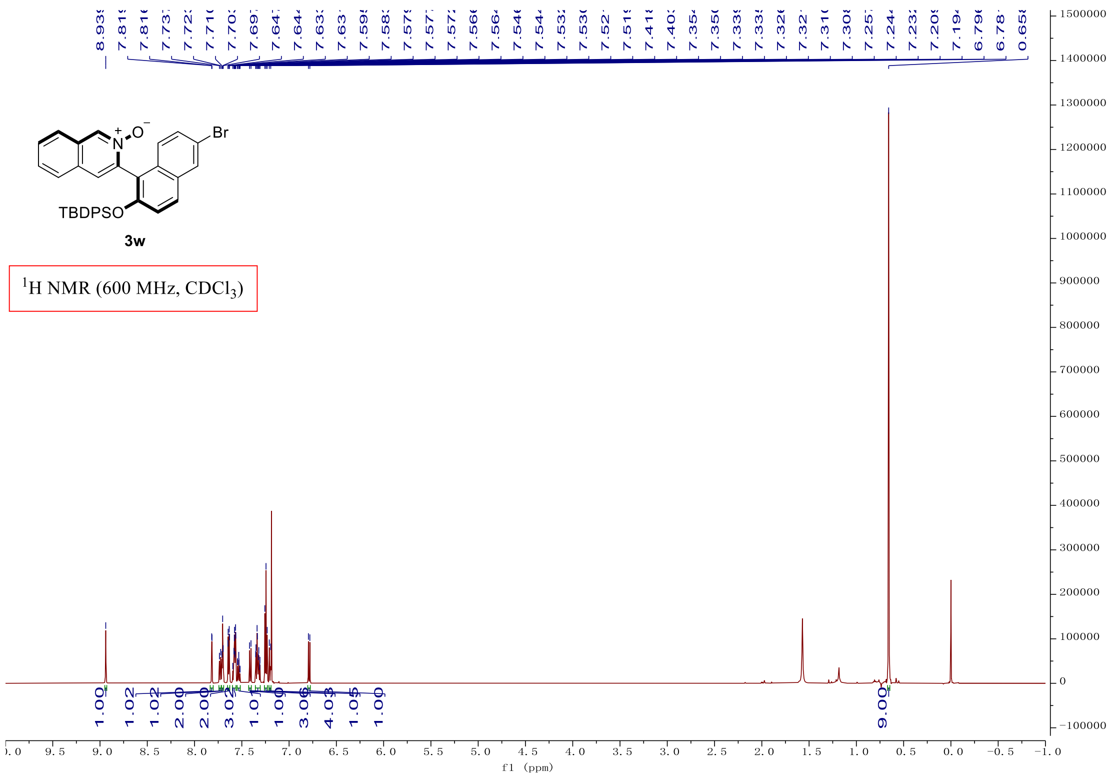

Br
TBDPSO
3w
1H NMR (600 MHz, CDCl3)
f1 (ppm)

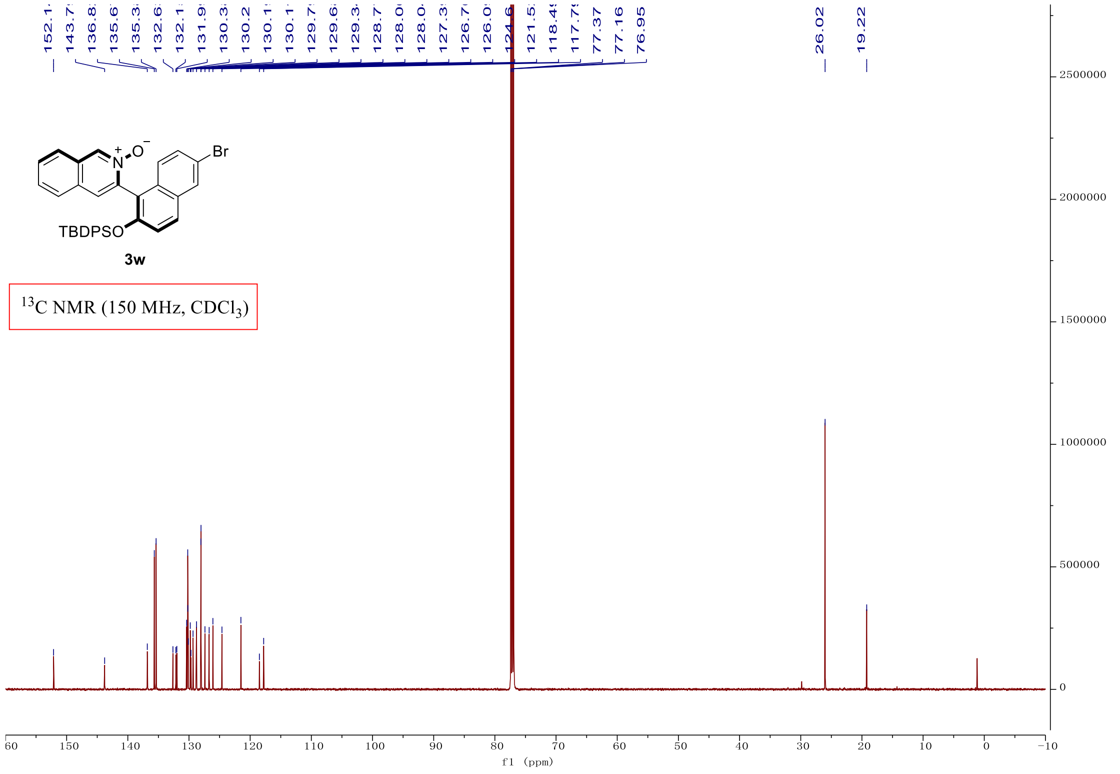

Br
TBDPSO
3w
13C NMR (150 MHz, CDCl3)
f1 (ppm)

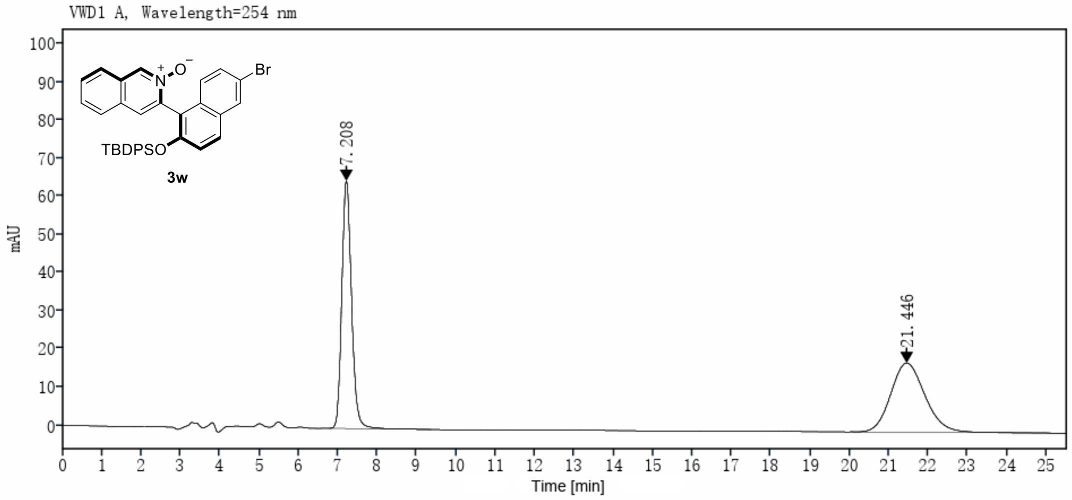

Signal: VWD1 A, Wavelength=254 nm

RT [min]Type	Area	Width[min]	Area%
7.208	1112.7031	0.255	50.3333
21.446	1097.9670	0.920	49.6667
Sum	2210.6702		

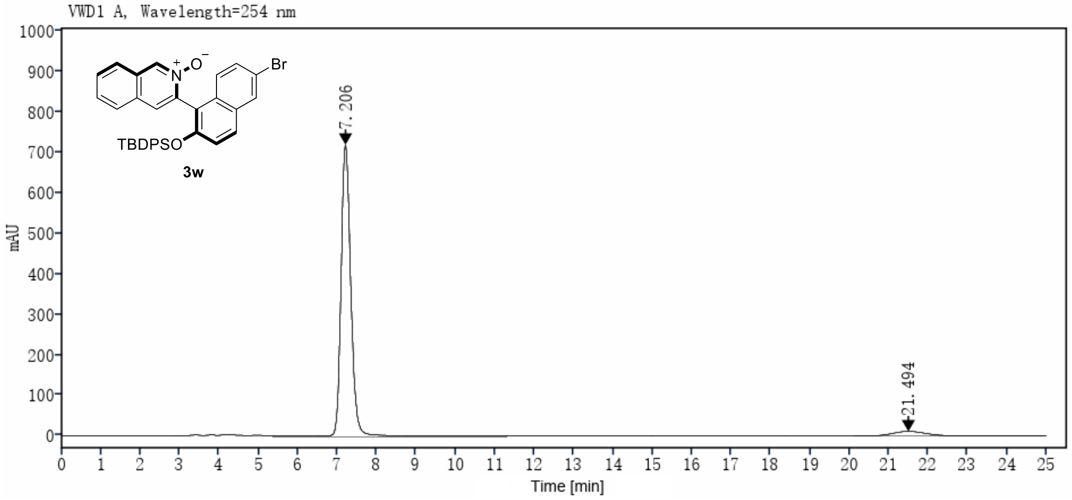

Signal: VWD1 A, Wavelength=254 nm

RT [min]Type	Area	Width[min]	Area%
7.206	13254.0508	0.258	94.9984
21.494	697.8207	0.920	5.0016
Sum	13951.8715		

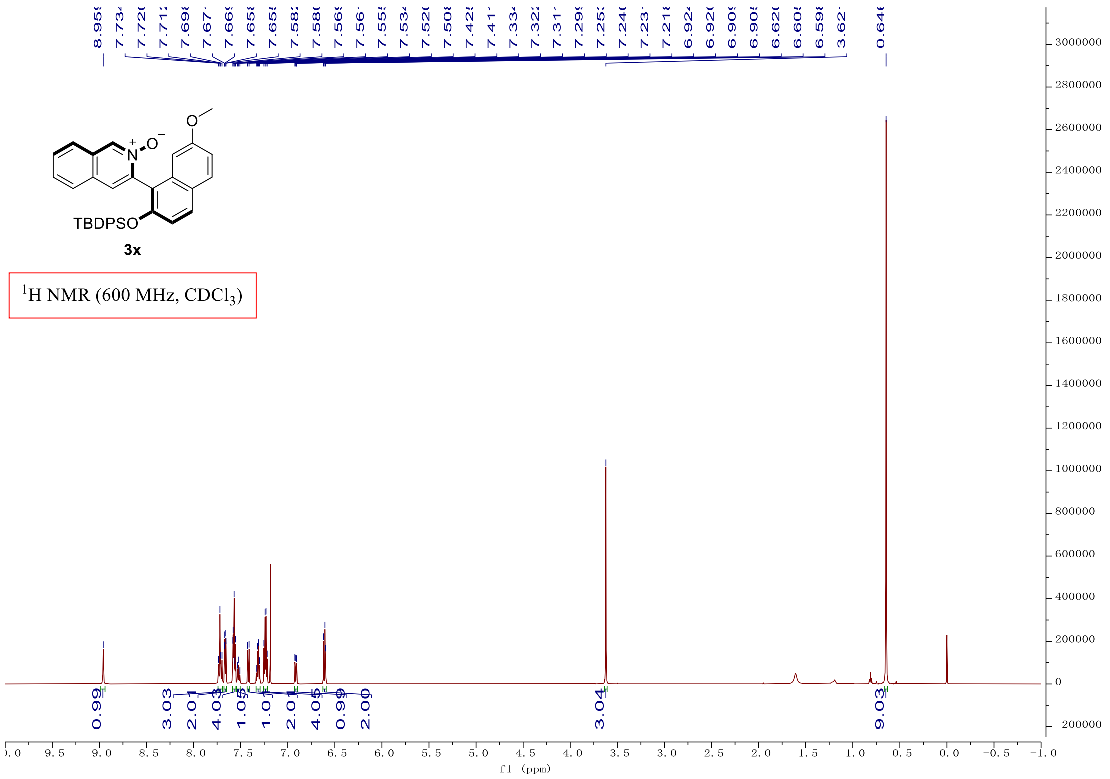
TBDPSO
3x
^{1}H NMR (600 MHz, CDCl$_3$)
f1 (ppm)

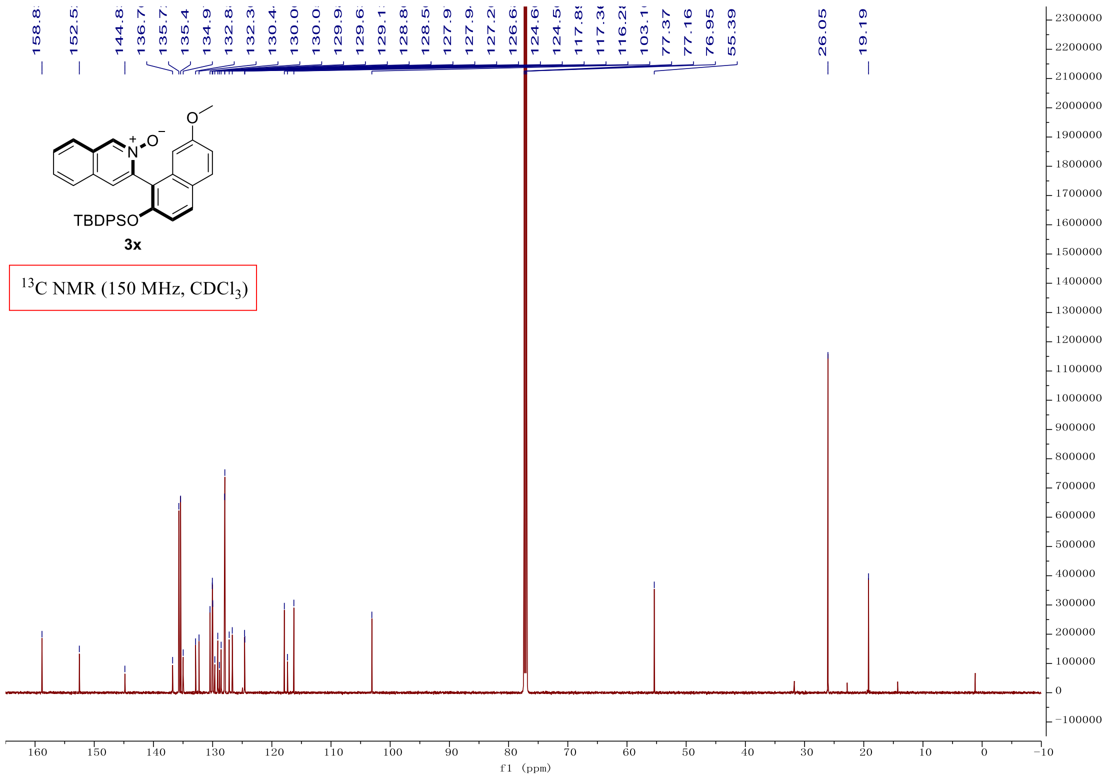
TBDPSO
3x
^{13}C NMR (150 MHz, CDCl$_3$)
f1 (ppm)

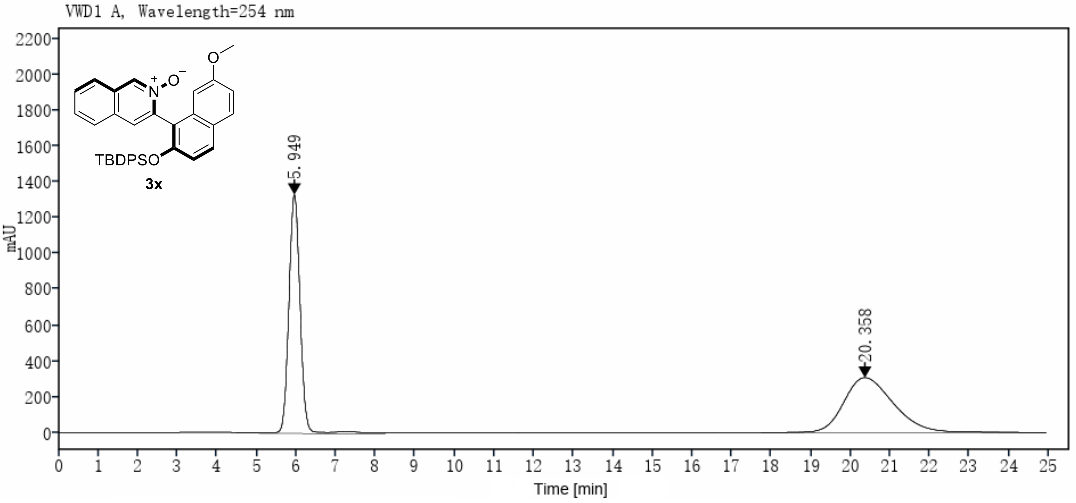

Signal: VWD1 A, Wavelength=254 nm

RT [min]Type	Area	Width[min]	Area%
5.949	27030.3281	0.299	50.2735
20.358	26736.2598	1.331	49.7265
Sum	53766.5879		

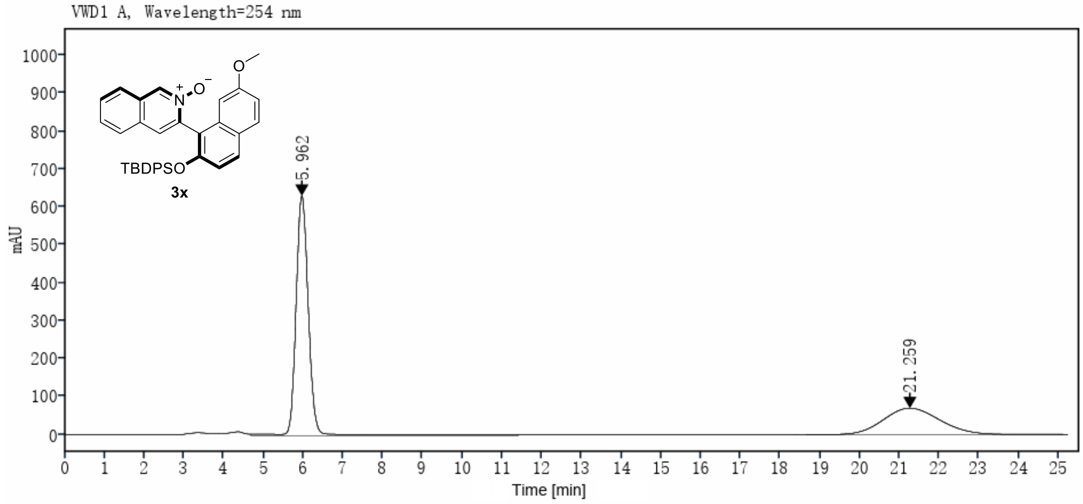

Signal: VWD1 A, Wavelength=254 nm

RT [min]Type	Area	Width[min]	Area%
5.962	14683.6172	0.323	67.9441
21.259	6927.7085	1.542	32.0559
Sum	21611.3257		

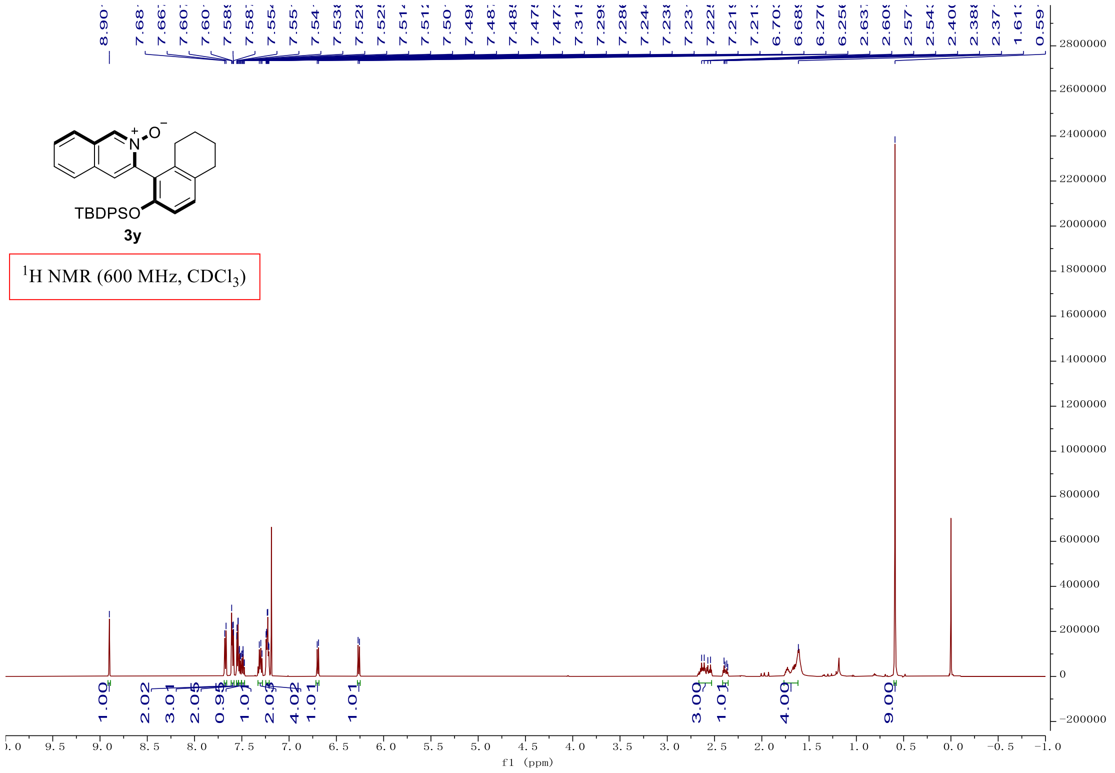
TBDPSO
3y
^{1}H NMR (600 MHz, CDCl$_3$)
f1 (ppm)

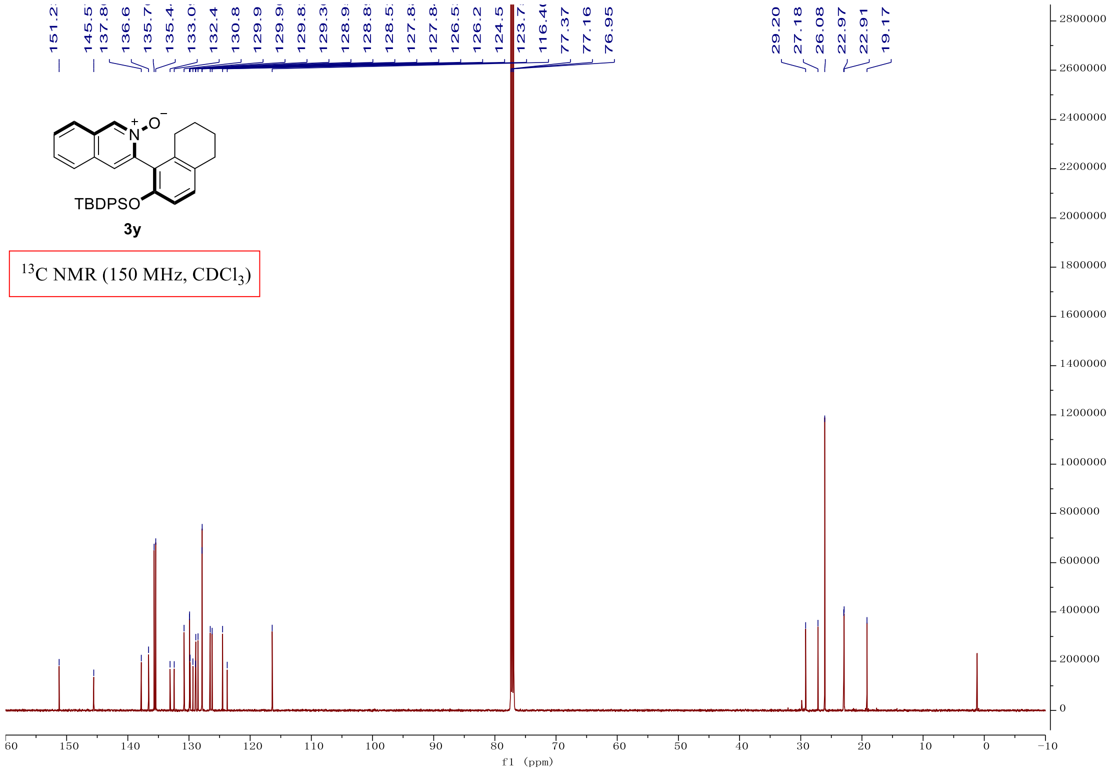
TBDPSO
3y
^{13}C NMR (150 MHz, CDCl$_3$)
f1 (ppm)

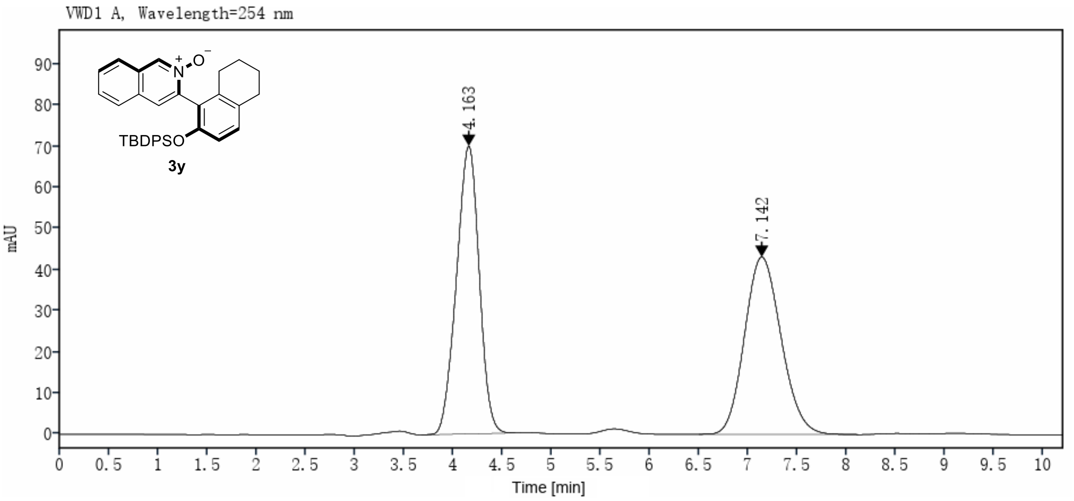

Signal: VWD1 A, Wavelength=254 nm

RT [min]Type	Area	Width[min]	Area%
4.163	1125.4368	0.251	50.2315
7.142	1115.0634	0.402	49.7685
Sum	2240.5001		

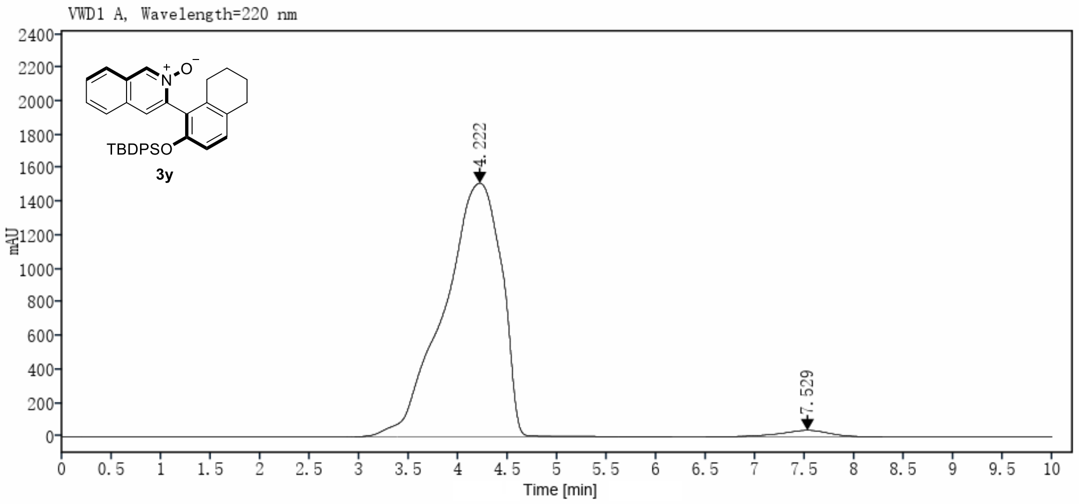

Signal: VWD1 A, Wavelength=220 nm

RT [min]Type	Area	Width[min]	Area%
4.222	61293.5664	0.632	97.9793
7.529	1264.1189	0.500	2.0207
Sum	62557.6853		

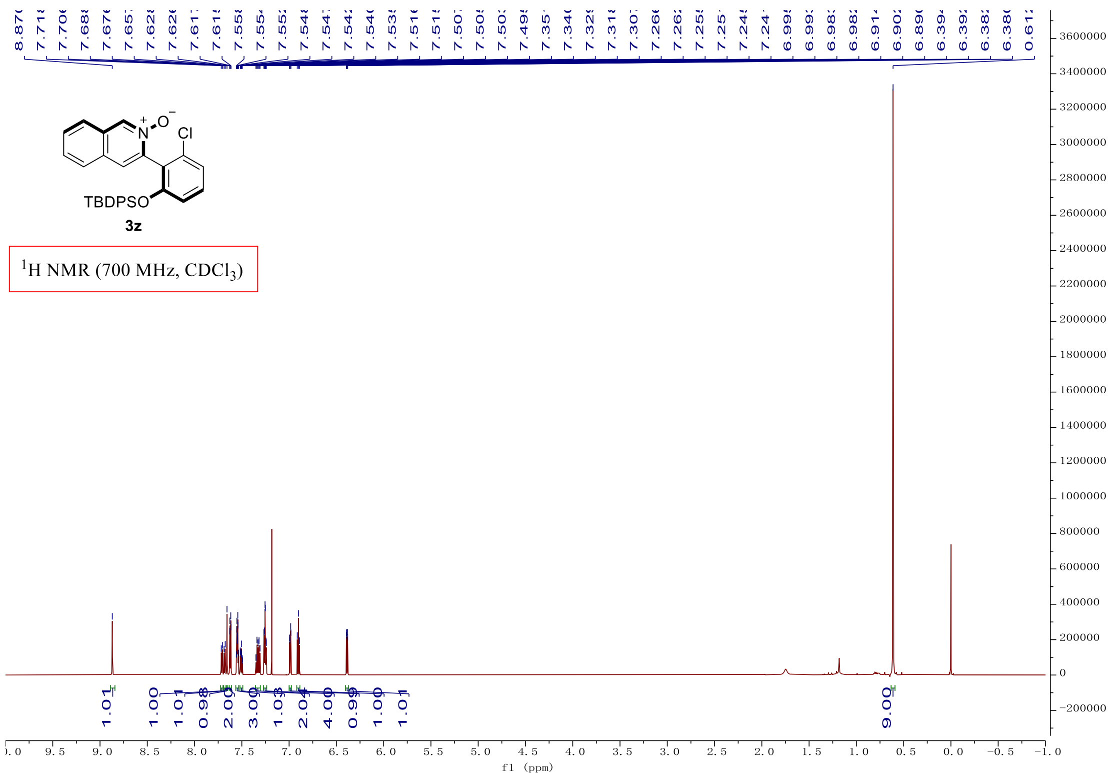

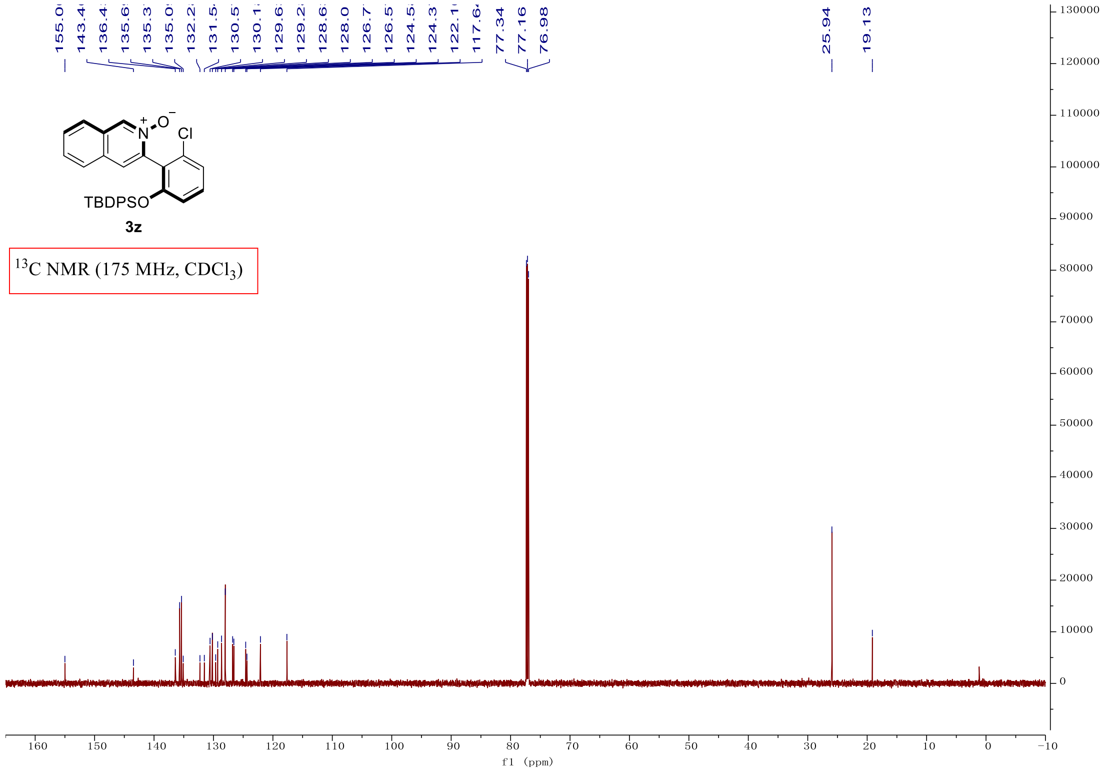

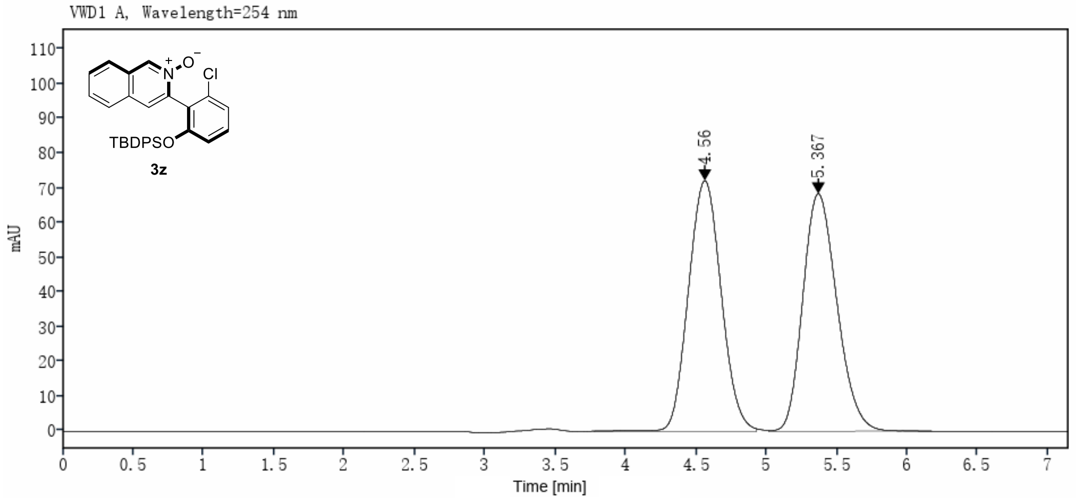

Signal: VWD1 A, Wavelength=254 nm

RT [min]Type	Area	Width[min]	Area%
4.560	1178.1783	0.254	50.5383
5.367	1153.0790	0.262	49.4617
Sum	2331.2573		

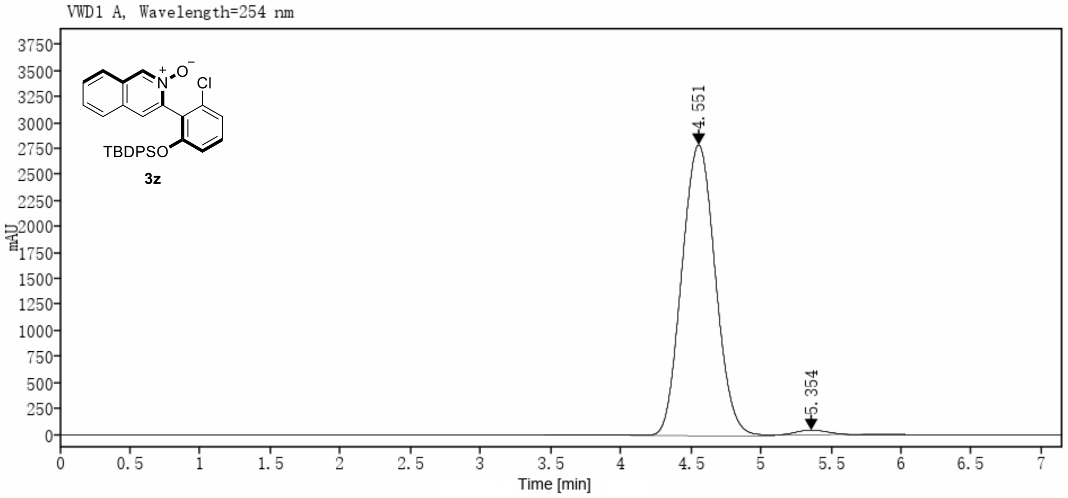

Signal: VWD1 A, Wavelength=254 nm

RT [min]Type	Area	Width[min]	Area%
4.551	46461.3711	0.261	98.5930
5.354	663.0375	0.251	1.4070
Sum	47124.4086		

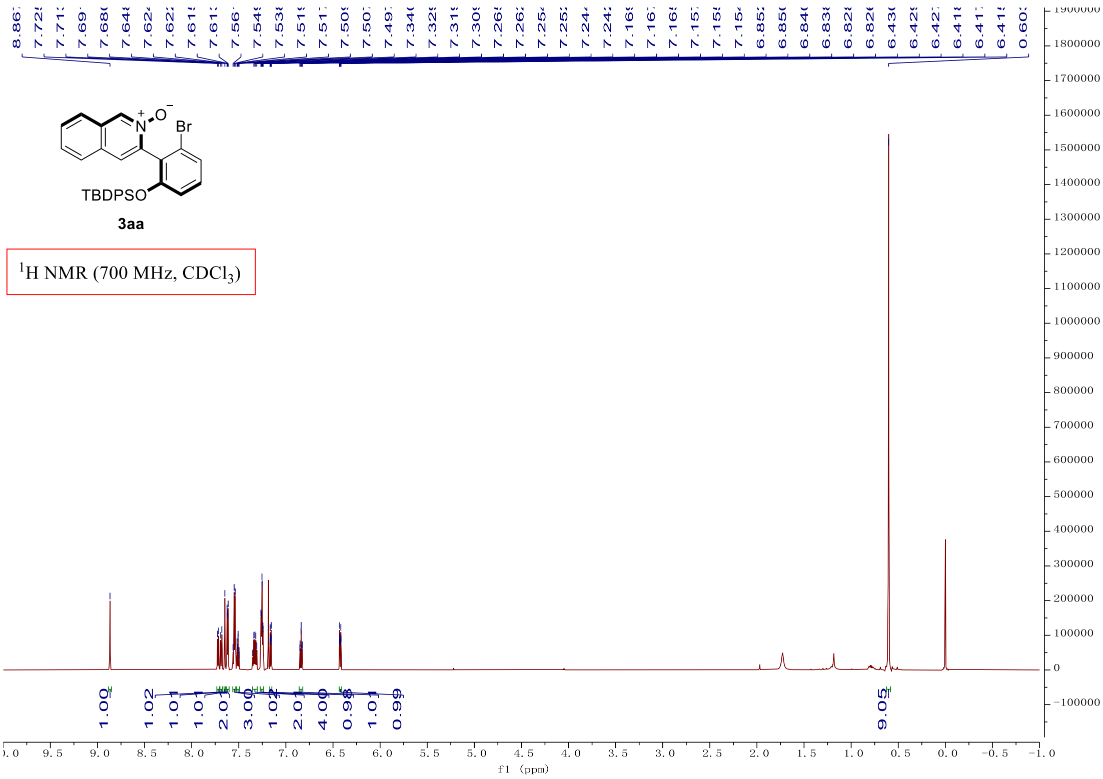
TBDPSO
Br
3aa
^{1}H NMR (700 MHz, $CDCl_3$)
f1 (ppm)

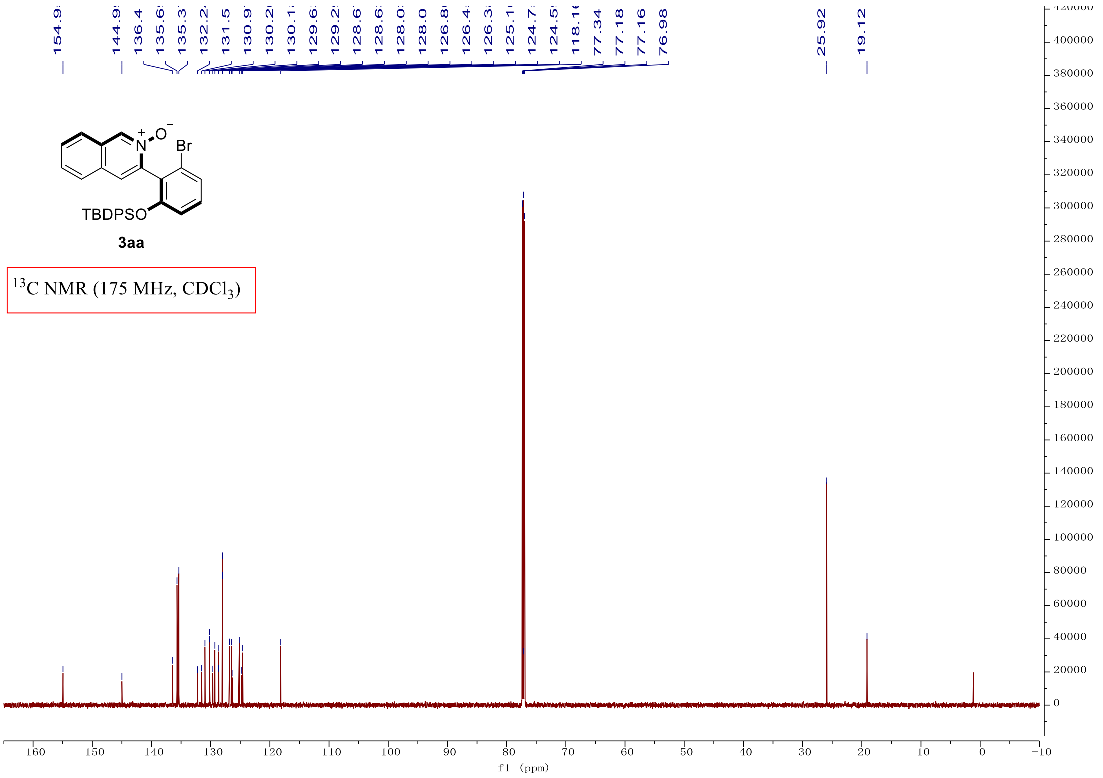
TBDPSO
Br
3aa
^{13}C NMR (175 MHz, $CDCl_3$)
f1 (ppm)

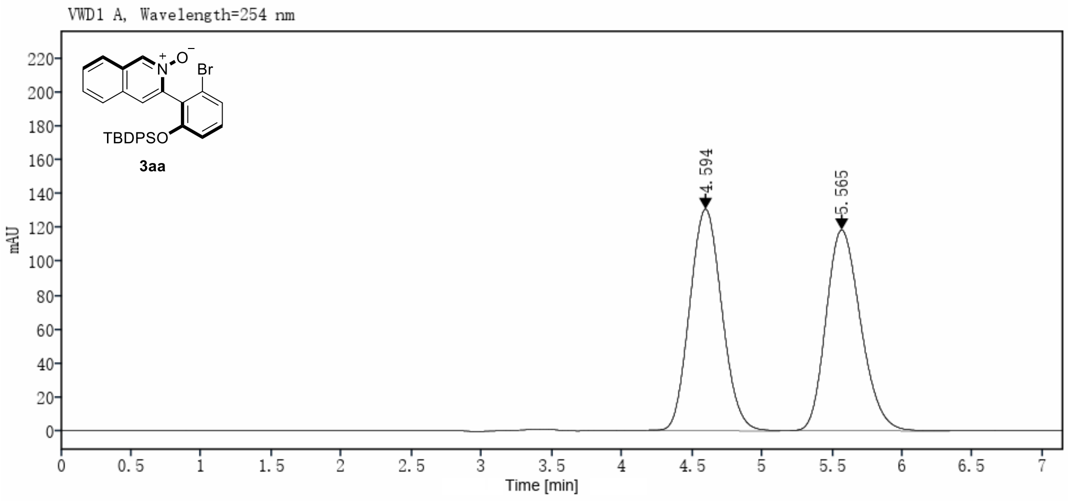

Signal: VWD1 A, Wavelength=254 nm

RT [min]Type	Area	Width[min]	Area%
4.594	2101.3330	0.250	50.6451
5.565	2047.7988	0.267	49.3549
Sum	4149.1318		

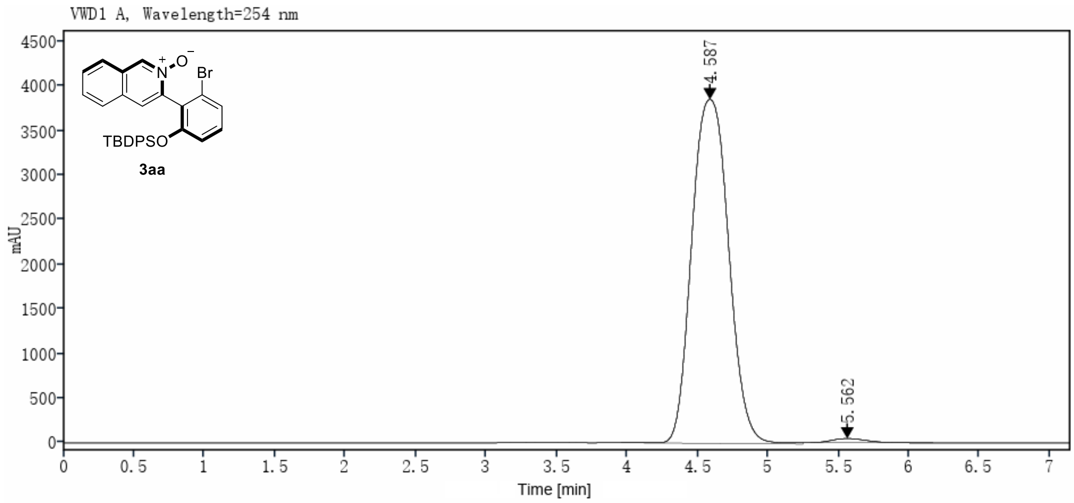

Signal: VWD1 A, Wavelength=254 nm

RT [min]Type	Area	Width[min]	Area%
4.587	70180.1719	0.289	98.9042
5.562	777.5527	0.267	1.0958
Sum	70957.7246		

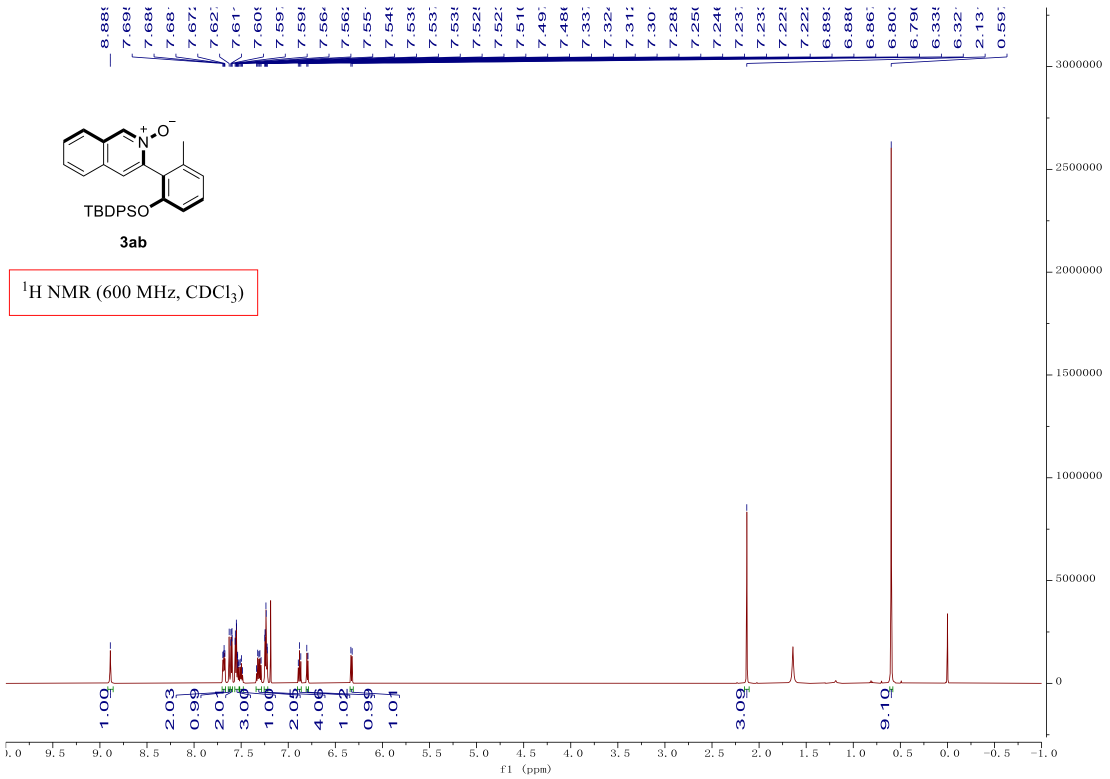
TBDPSO
3ab
1H NMR (600 MHz, CDCl3)
f1 (ppm)

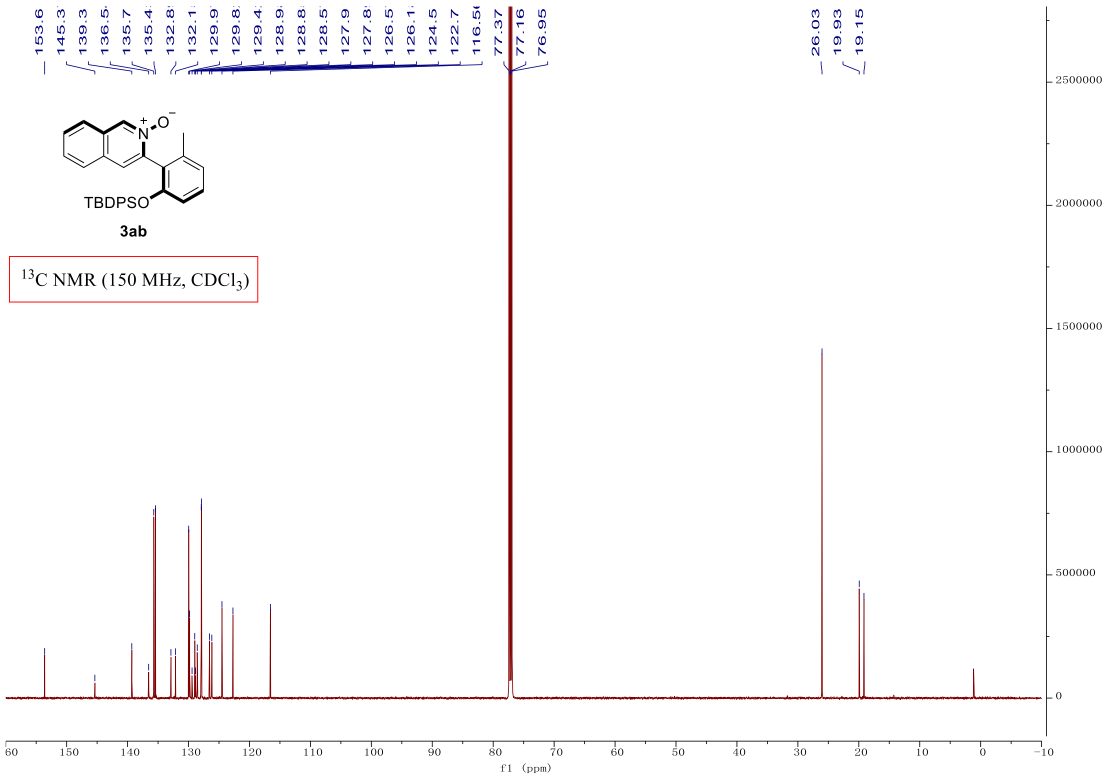
TBDPSO
3ab
13C NMR (150 MHz, CDCl3)
f1 (ppm)

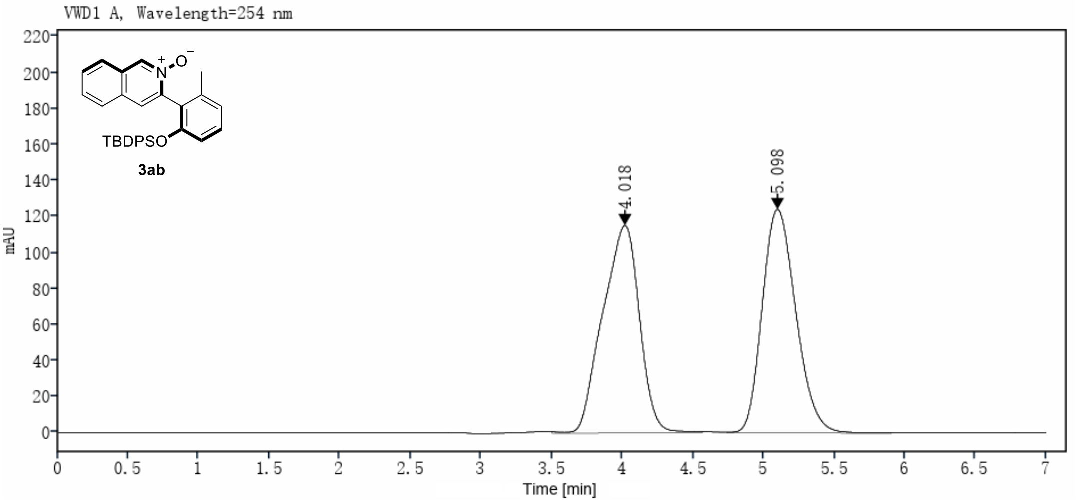

Signal: VWD1 A, Wavelength=254 nm

RT [min]Type	Area	Width[min]	Area%
4.018	2084.4114	0.295	50.3147
5.098	2058.3337	0.258	49.6853
Sum	4142.7451		

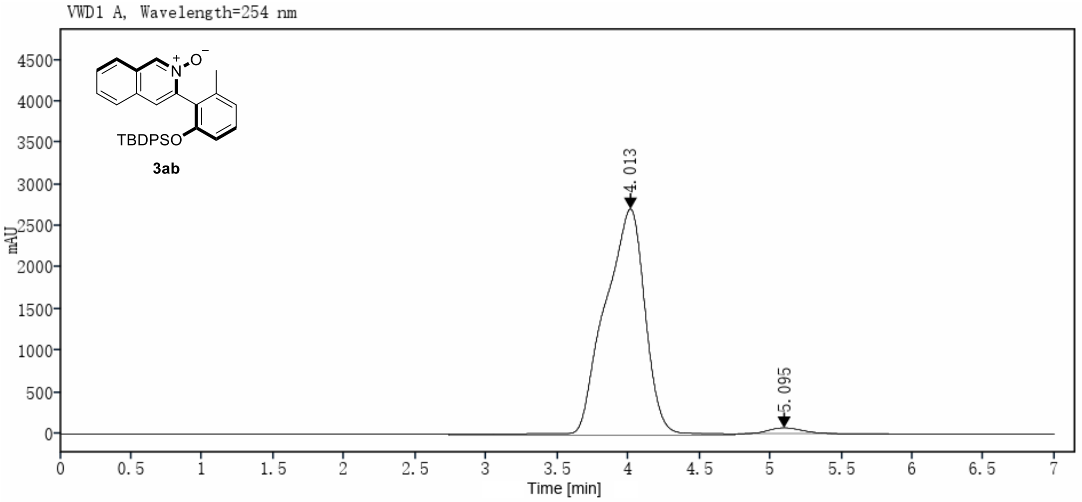

Signal: VWD1 A, Wavelength=254 nm

RT [min]Type	Area	Width[min]	Area%
4.013	54295.3516	0.322	97.9719
5.095	1123.9479	0.260	2.0281
Sum	55419.2994		

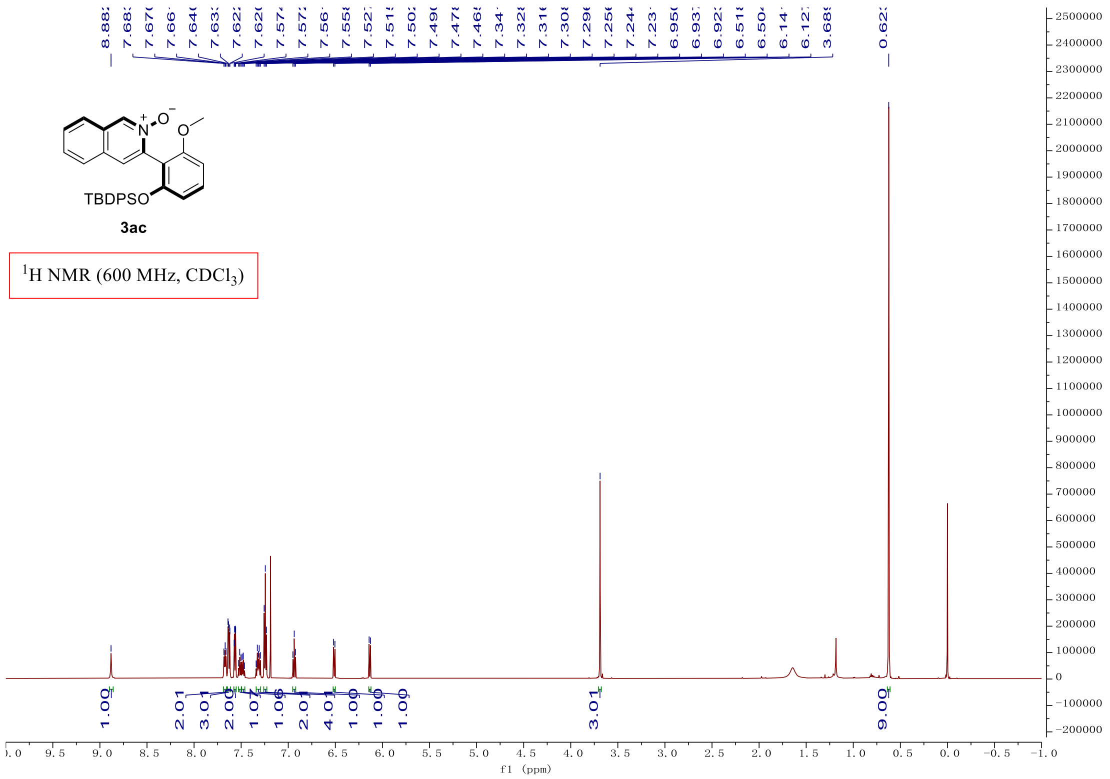
TBDPSO
3ac
^{1}H NMR (600 MHz, $CDCl_3$)
f1 (ppm)

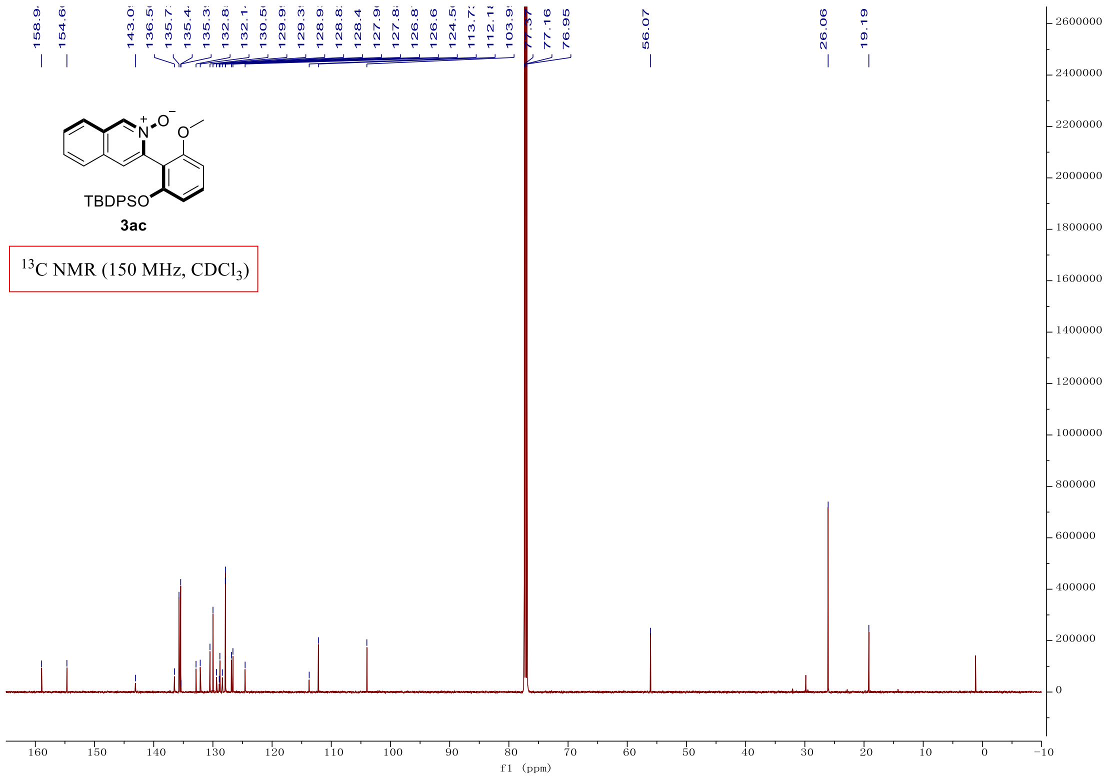
TBDPSO
3ac
^{13}C NMR (150 MHz, $CDCl_3$)
f1 (ppm)

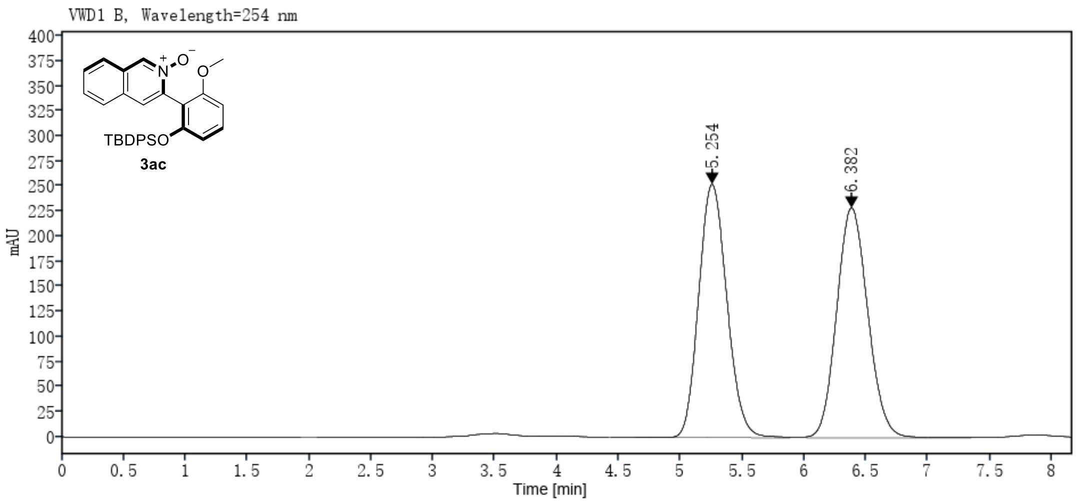

Signal: VWD1 B, Wavelength=254 nm

RT [min]Type	Area	Width[min]	Area%
5.254	4001.3689	0.245	49.7610
6.382	4039.8064	0.271	50.2390
Sum	8041.1753		

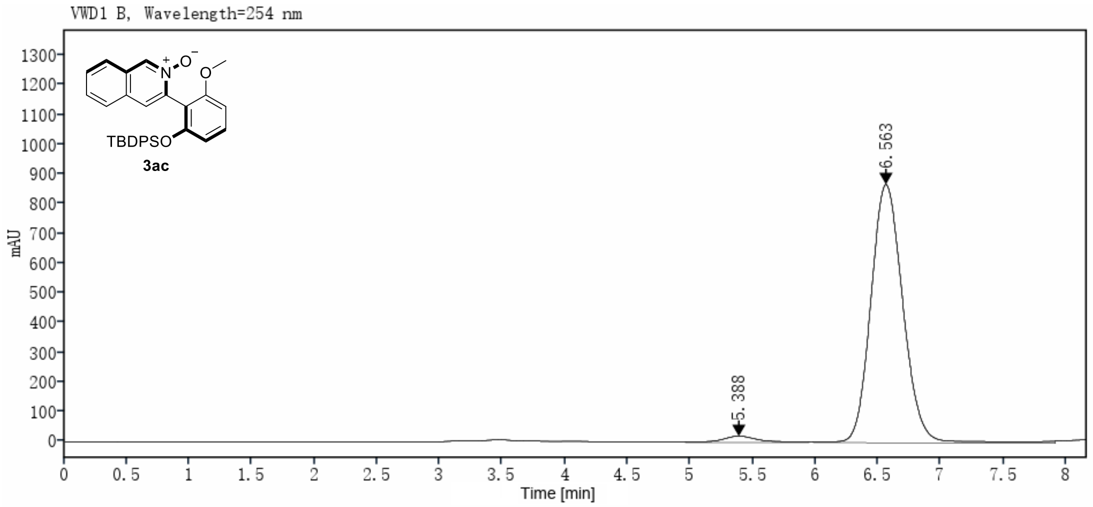

Signal: VWD1 B, Wavelength=254 nm

RT [min]Type	Area	Width[min]	Area%
5.388	451.9762	0.271	2.8099
6.563	15633.0225	0.272	97.1901
Sum	16084.9987		

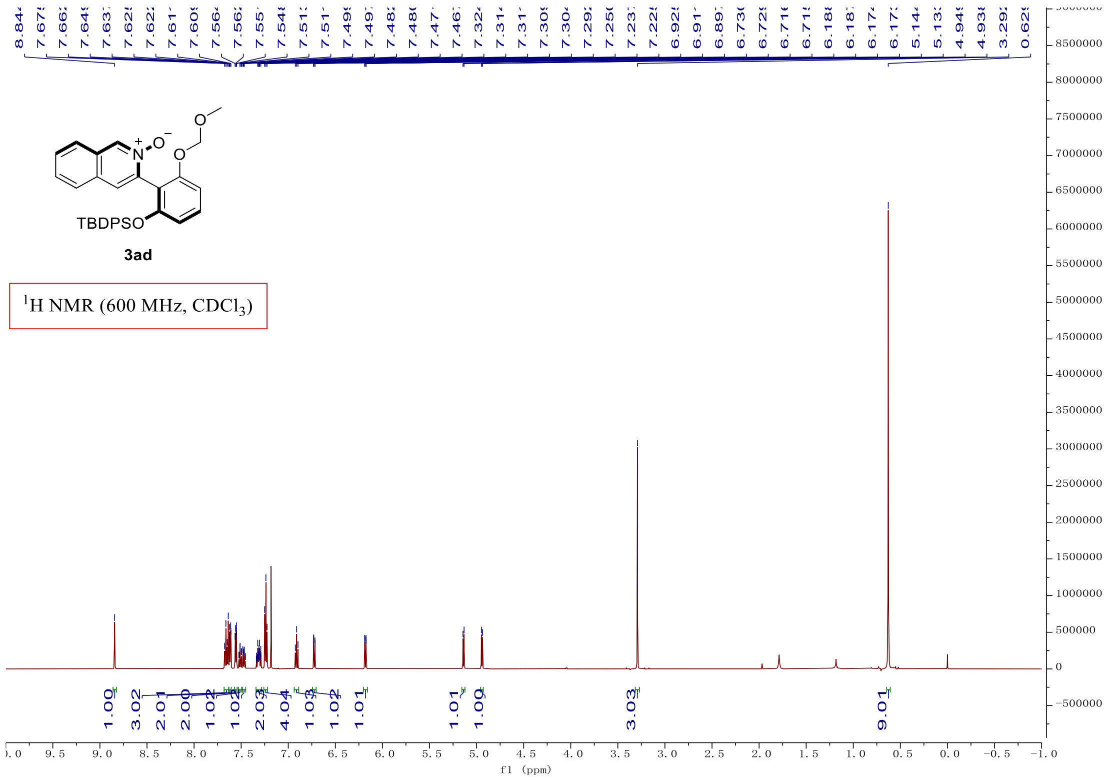

TBDPSO
3ad
1H NMR (600 MHz, CDCl3)
f1 (ppm)

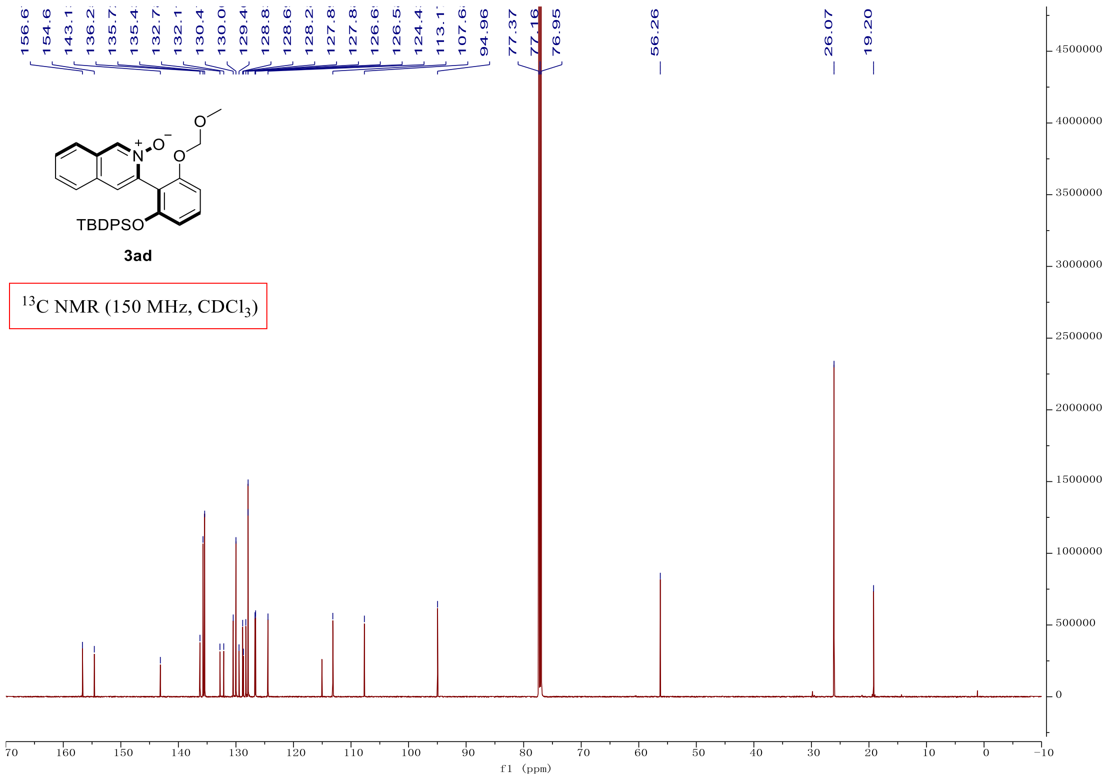

156.6
154.6
143.1
136.2
135.7
135.4
132.7
132.1
130.4
130.0
129.4
128.8
128.6
128.2
127.8
127.8
126.6
126.5
124.4
113.1
107.6
94.96
77.37
77.16
76.95
56.26
26.07
19.20
TBDPSO
3ad
13C NMR (150 MHz, CDCl3)
f1 (ppm)

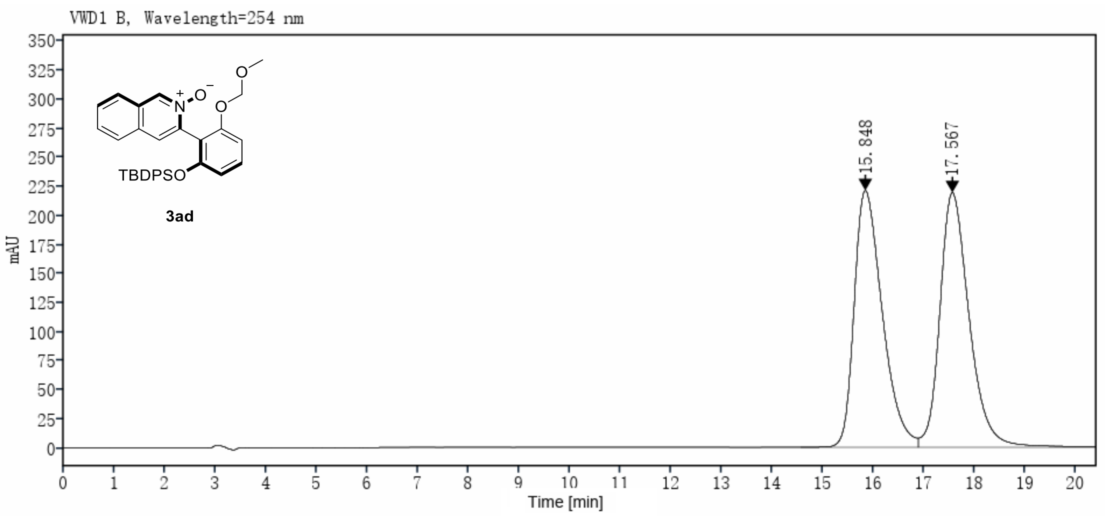

Signal: VWD1 B, Wavelength=254 nm

RT [min]Type	Area	Width[min]	Area%
15.848	8525.5557	0.591	49.0921
17.567	8840.8877	0.598	50.9079
Sum	17366.4434		

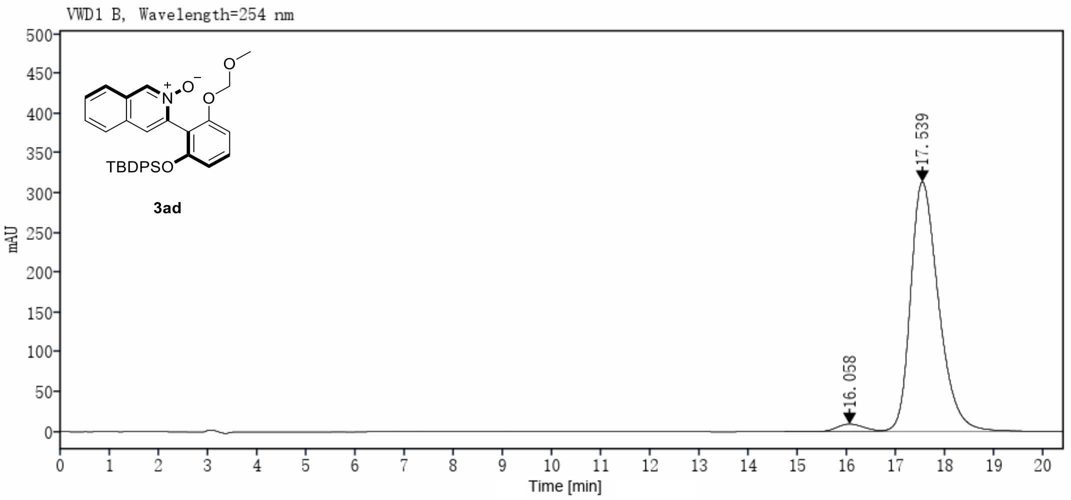

Signal: VWD1 B, Wavelength=254 nm

RT [min]Type	Area	Width[min]	Area%
16.058	384.0240	0.620	2.9735
17.539	12530.9570	0.599	97.0265
Sum	12914.9811		

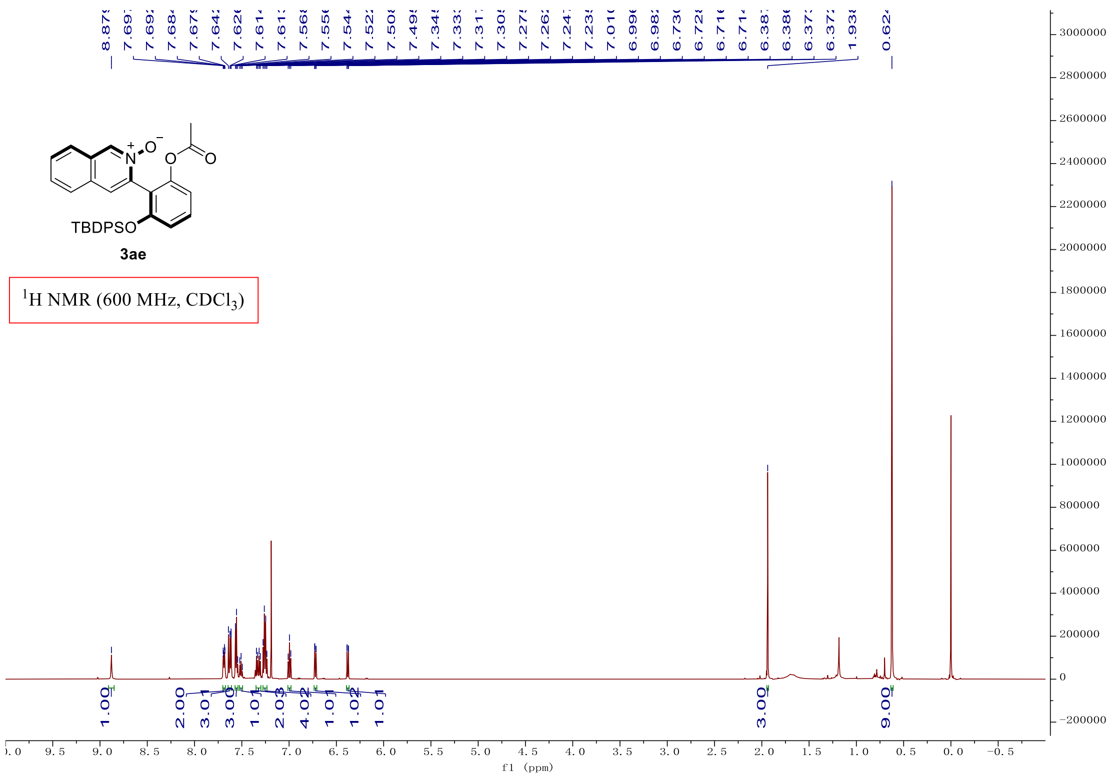
TBDPSO
3ae
1H NMR (600 MHz, CDCl3)
f1 (ppm)

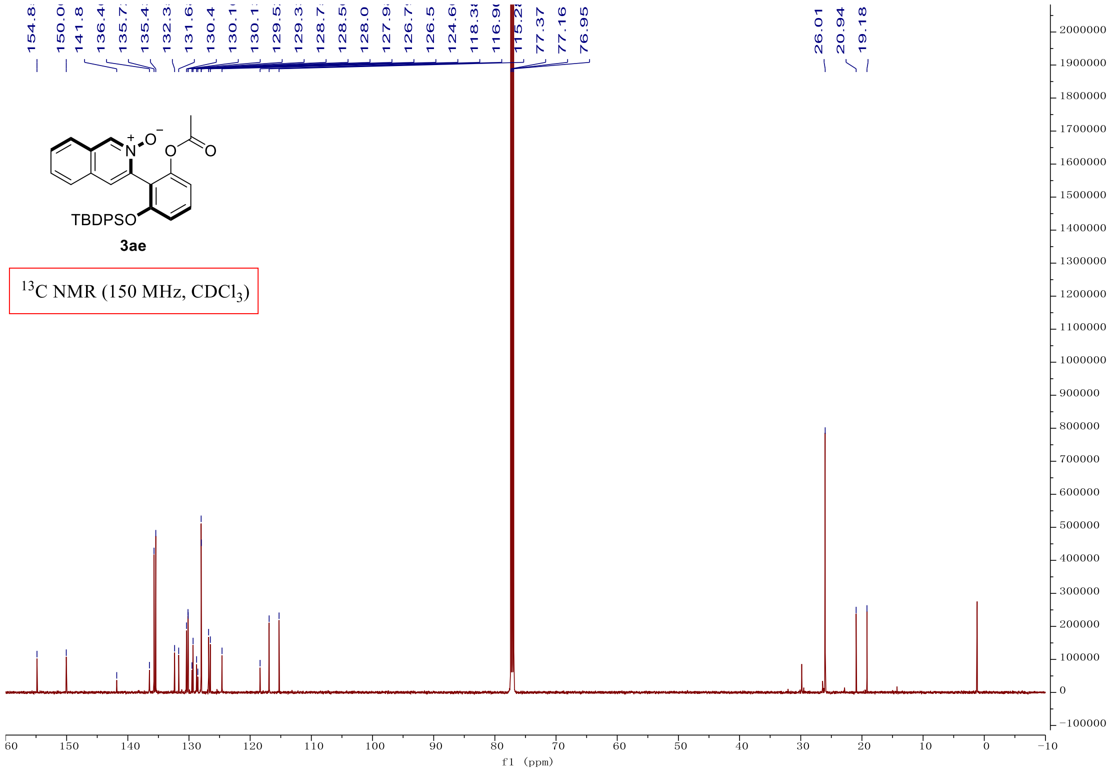
TBDPSO
3ae
13C NMR (150 MHz, CDCl3)
f1 (ppm)

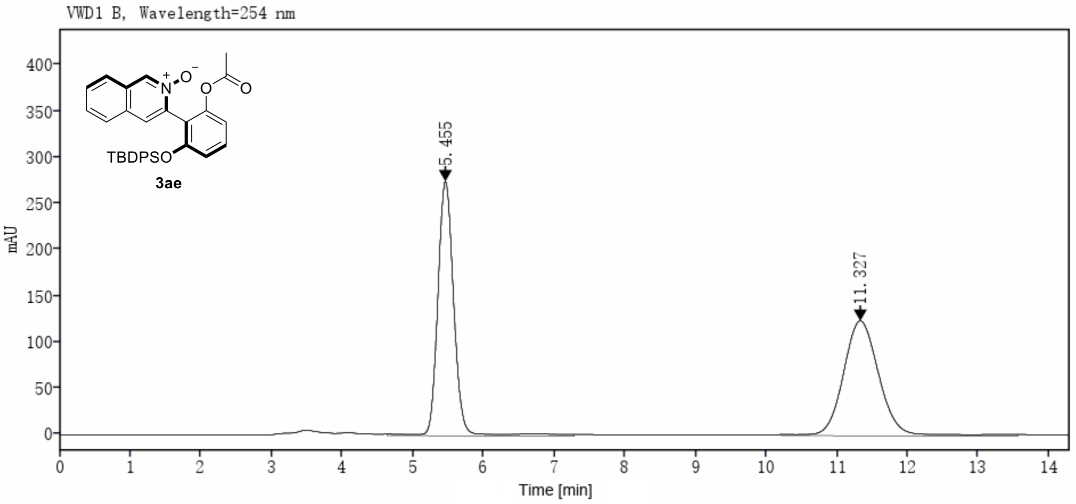

Signal: VWD1 B, Wavelength=254 nm

RT [min]Type	Area	Width[min]	Area%
5.455	4402.5889	0.235	49.8011
11.327	4437.7480	0.522	50.1989
Sum	8840.3369		

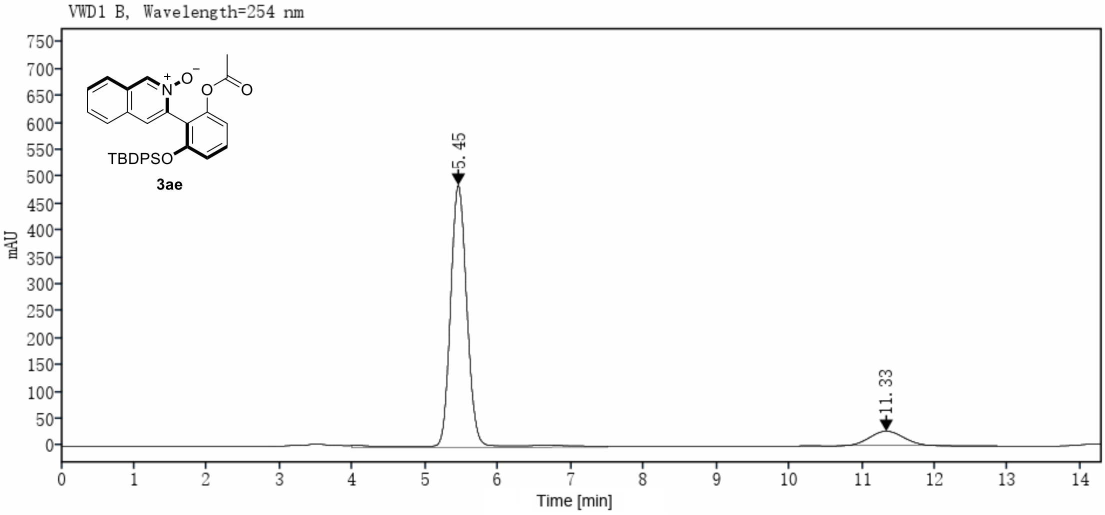

Signal: VWD1 B, Wavelength=254 nm

RT [min]Type	Area	Width[min]	Area%
5.450	7991.5972	0.237	90.4632
11.330	842.4907	0.520	9.5368
Sum	8834.0879		

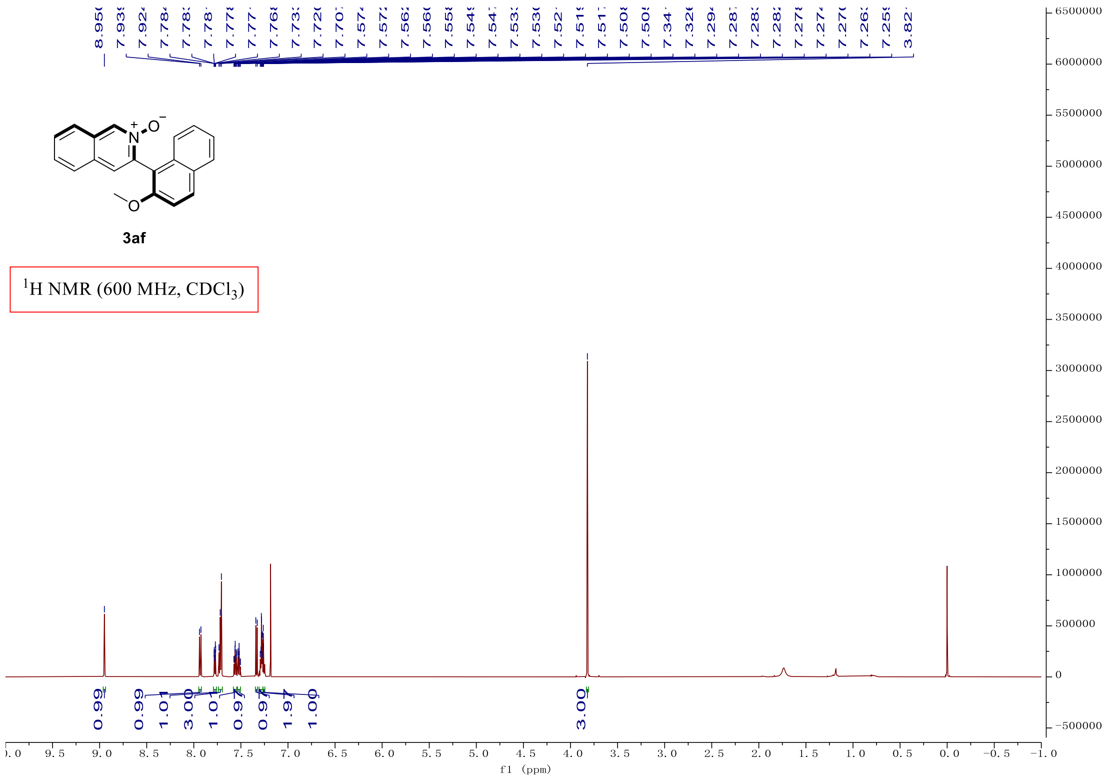
3af
^{1}H NMR (600 MHz, CDCl$_3$)
f1 (ppm)

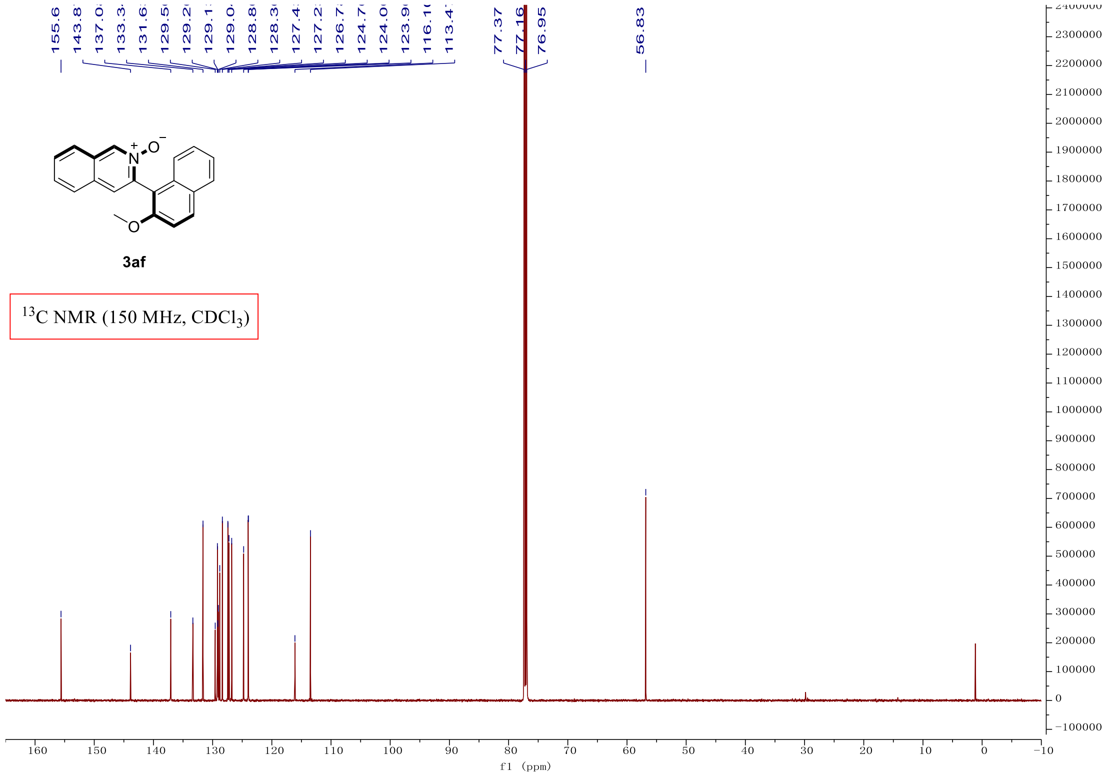
3af
^{13}C NMR (150 MHz, CDCl$_3$)
f1 (ppm)

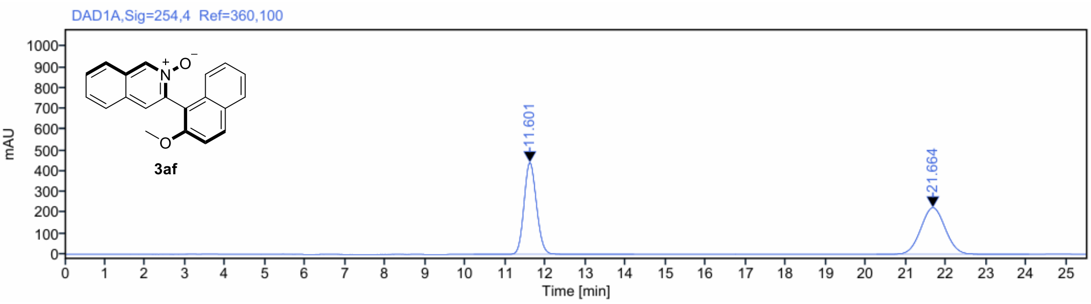

Signal: DAD1A,Sig=254,4 Ref=360,100

RT [min]	Type	Width [min]	Area	Height	Area%	Name
11.601	MM m	3.99	9530.93	441.77	50.04	
21.664	MM m	3.81	9514.72	224.47	49.96	
		**Sum**	**19045.64**			

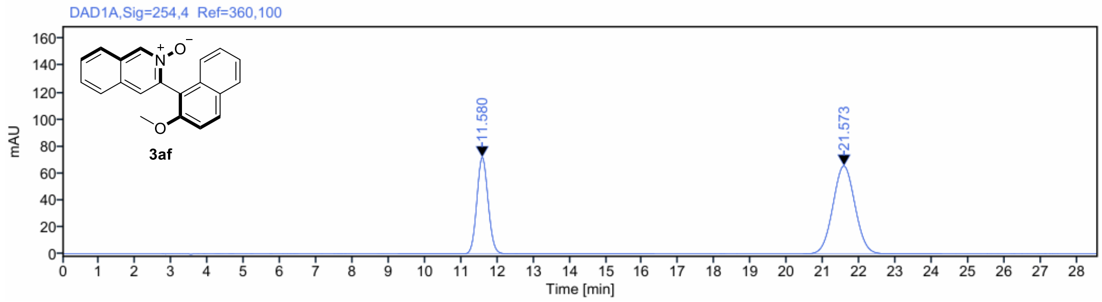

Signal: DAD1A,Sig=254,4 Ref=360,100

RT [min]	Type	Width [min]	Area	Height	Area%	Name
11.580	MM m	7.08	1552.99	71.81	35.98	
21.573	MM m	3.84	2763.52	65.34	64.02	
		**Sum**	**4316.51**			

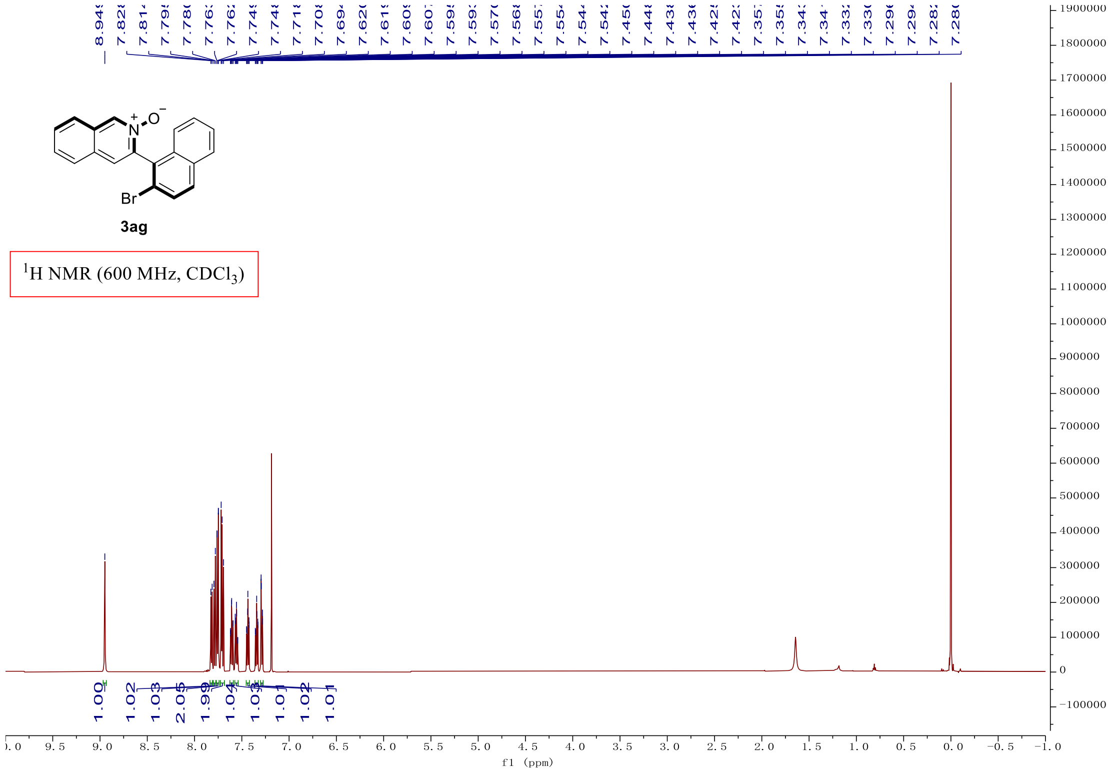
3ag
1H NMR (600 MHz, CDCl3)
f1 (ppm)

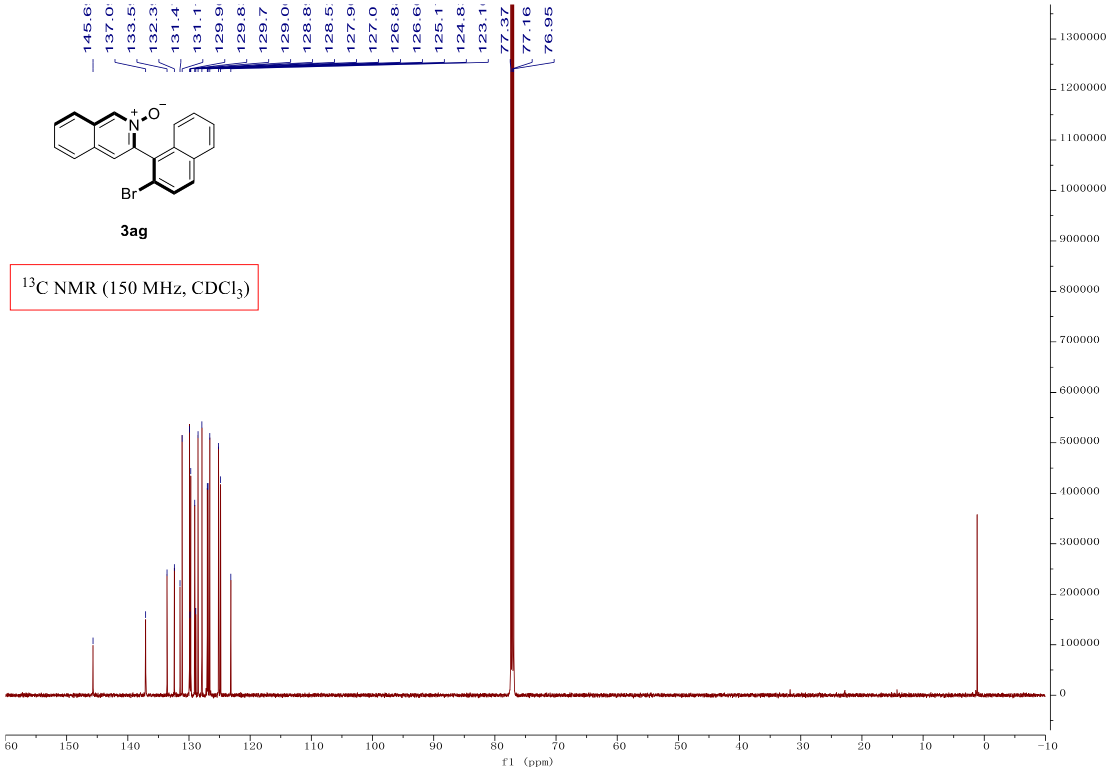
3ag
13C NMR (150 MHz, CDCl3)
f1 (ppm)

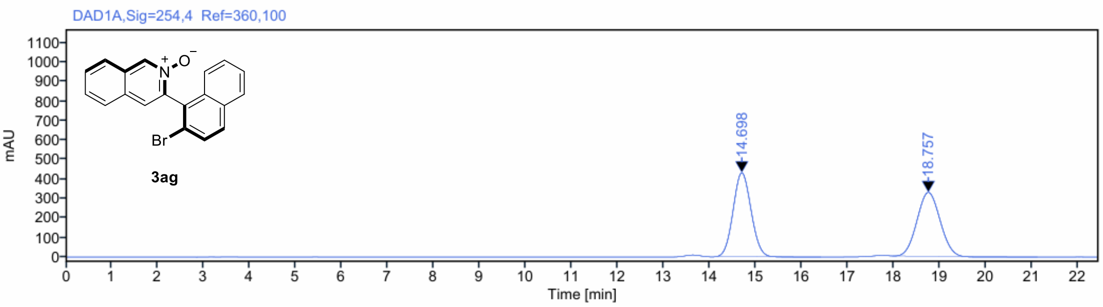

Signal:	DAD1A,Sig=254,4 Ref=360,100					
**RT [min]**	**Type**	**Width [min]**	**Area**	**Height**	**Area%**	**Name**
14.698	MM m	2.25	11563.41	430.00	50.50	
18.757	MM m	3.15	11333.60	327.84	49.50	
		**Sum**	**22897.01**			

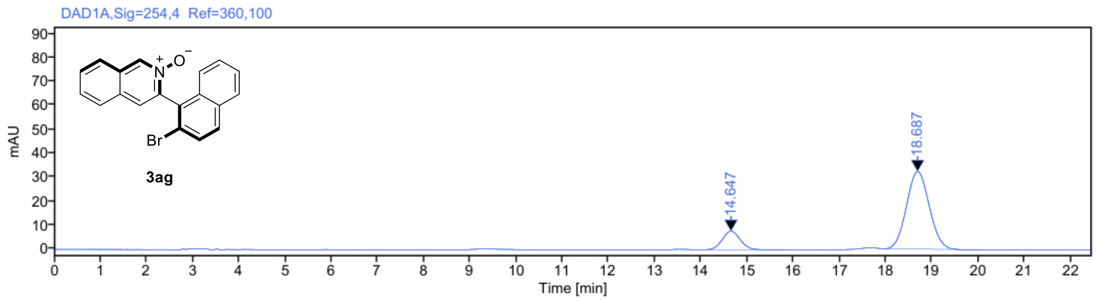

Signal:	DAD1A,Sig=254,4 Ref=360,100					
**RT [min]**	**Type**	**Width [min]**	**Area**	**Height**	**Area%**	**Name**
14.647	MM m	1.80	205.40	7.71	15.64	
18.687	MM m	2.14	1107.90	32.42	84.36	
		**Sum**	**1313.30**			

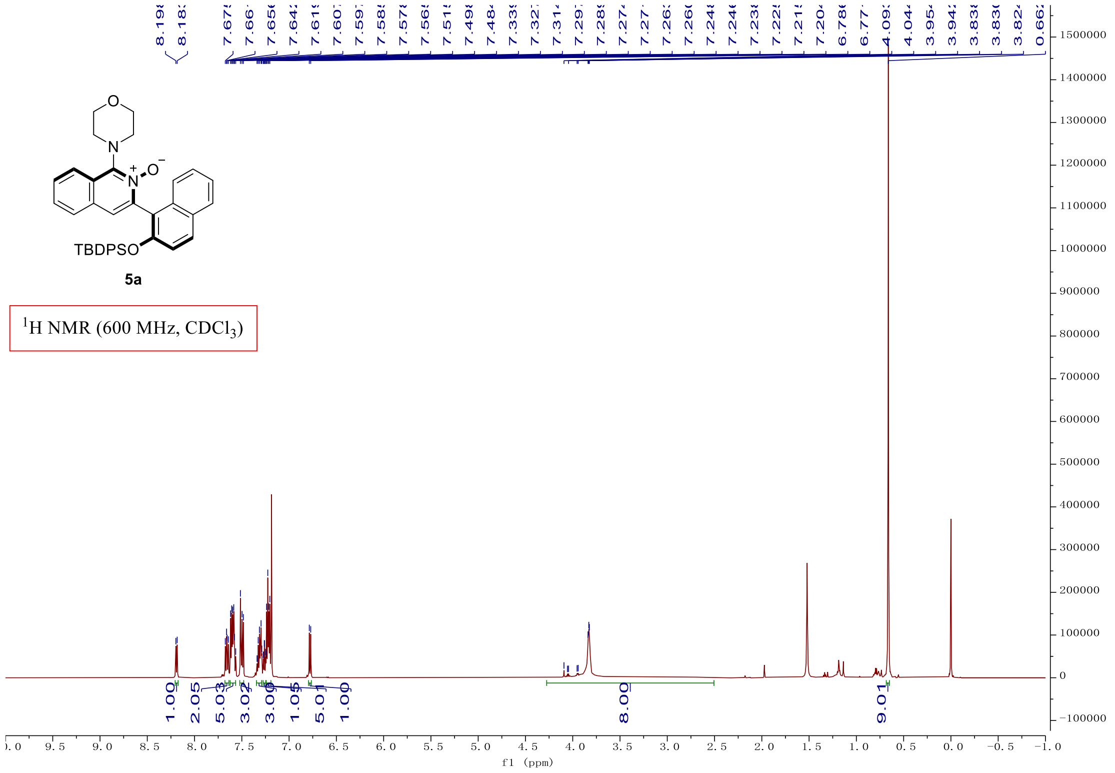
TBDPSO
5a
1H NMR (600 MHz, CDCl3)
f1 (ppm)

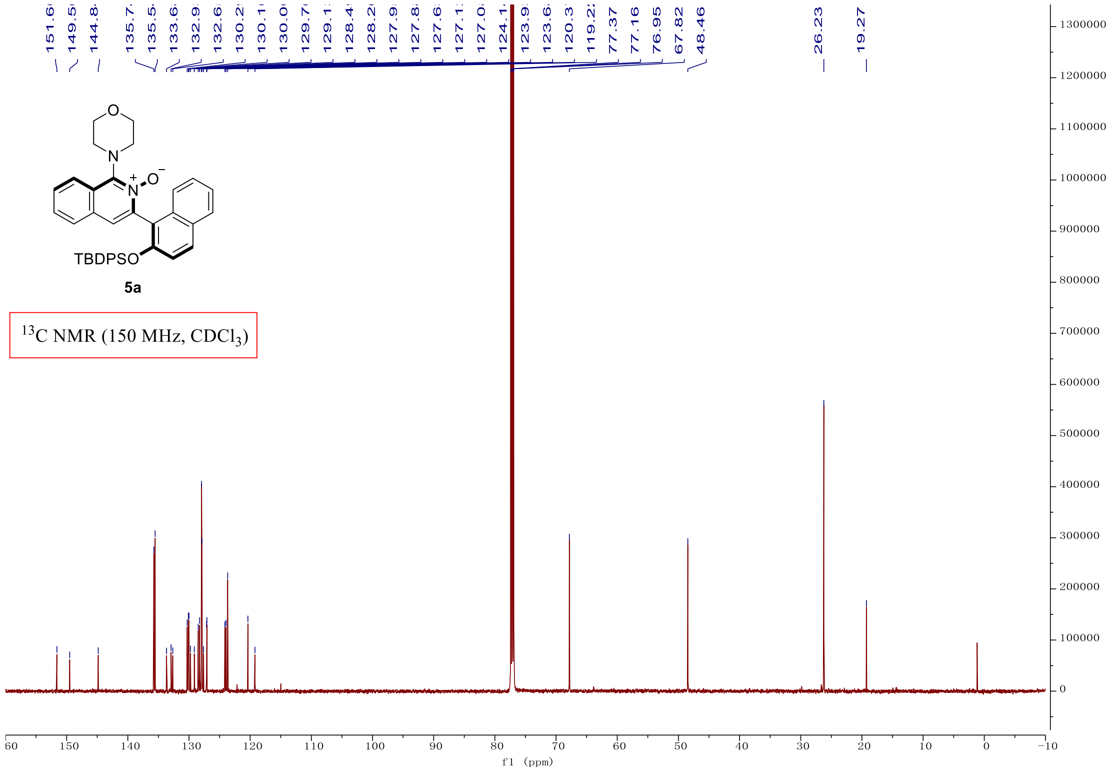
TBDPSO
5a
13C NMR (150 MHz, CDCl3)
f1 (ppm)

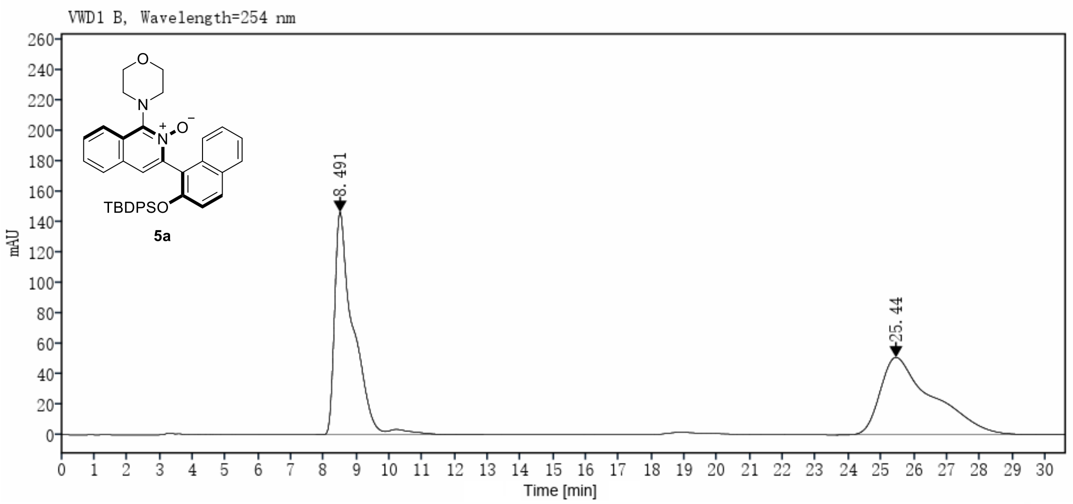

Signal: VWD1 B, Wavelength=254 nm

RT [min]Type	Area	Width[min]	Area%
8.491	5860.2153	0.537	50.7717
25.440	5682.0811	1.588	49.2283
Sum	11542.2964		

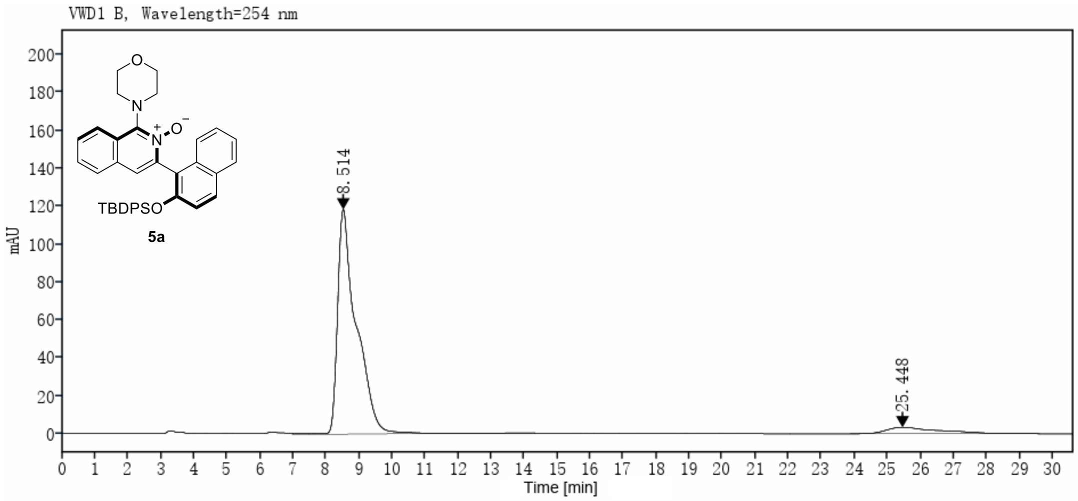

Signal: VWD1 B, Wavelength=254 nm

RT [min]Type	Area	Width[min]	Area%
8.514	4691.3125	0.537	92.9598
25.448	355.2897	1.567	7.0402
Sum	5046.6022		

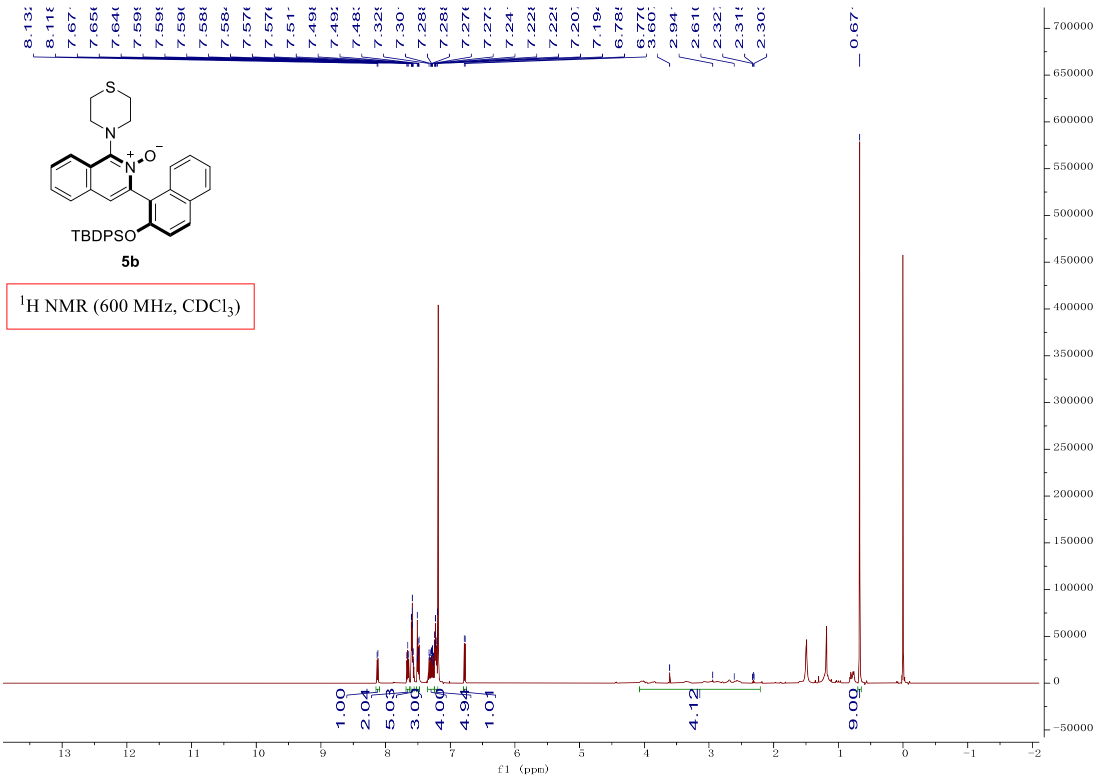

S
N
N
O
TBDPSO
5b
^{1}H NMR (600 MHz, CDCl$_3$)
f1 (ppm)

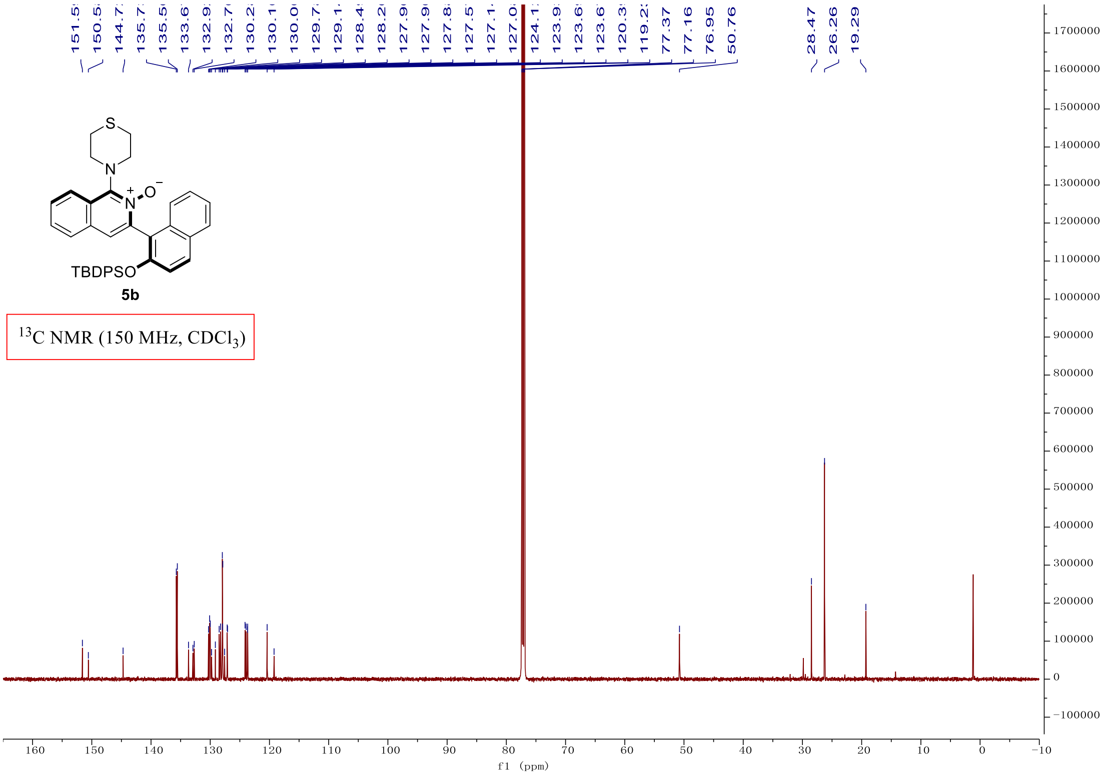

S
N
N
O
TBDPSO
5b
^{13}C NMR (150 MHz, CDCl$_3$)
f1 (ppm)

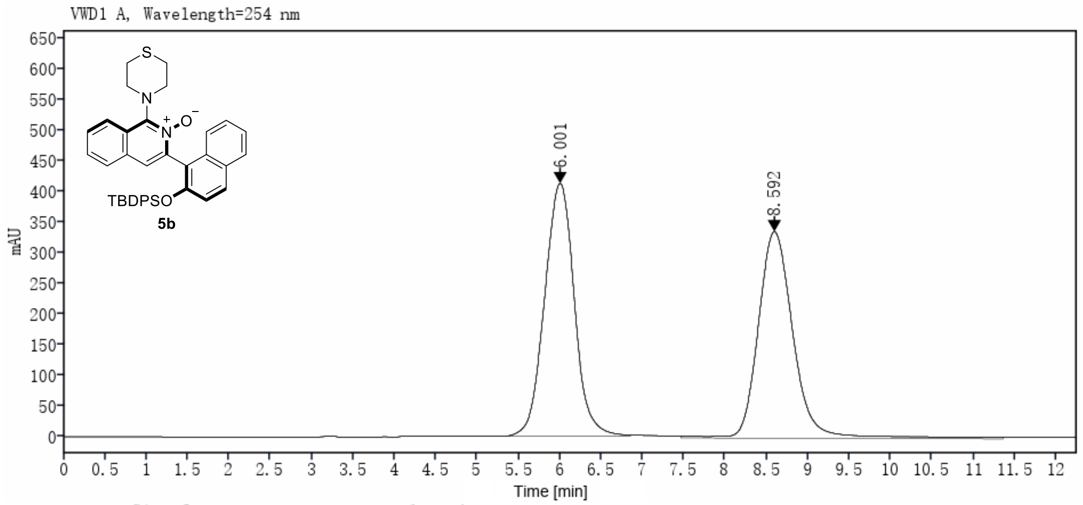

Signal:	VWD1 A, Wavelength=254 nm		
RT [min]Type	Area	Width[min]	Area%
6.001	10272.3818	0.386	50.5772
8.592	10037.9063	0.431	49.4228
Sum	20310.2881		

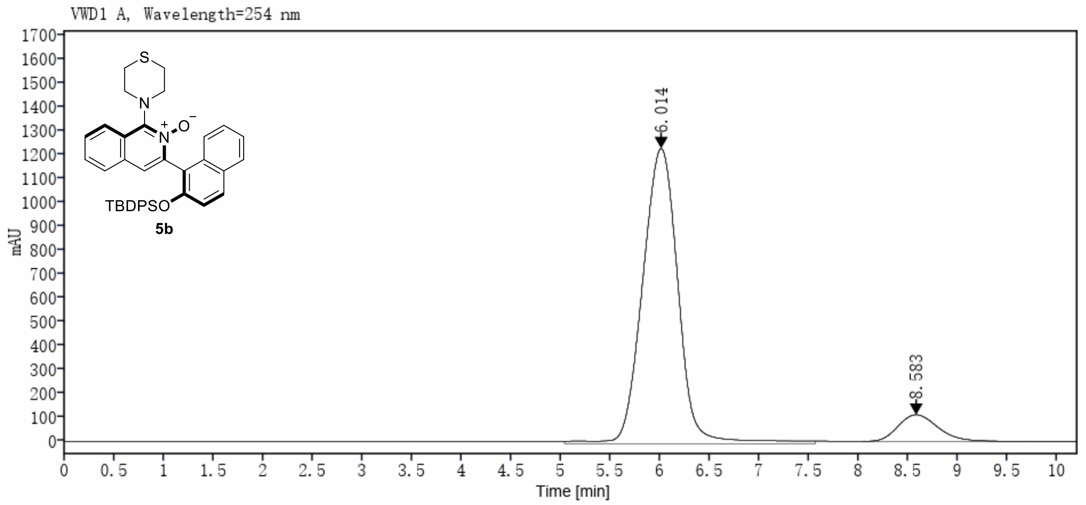

Signal:	VWD1 A, Wavelength=254 nm		
RT [min]Type	Area	Width[min]	Area%
6.014	30767.0371	0.378	91.0171
8.583	3036.5259	0.437	8.9829
Sum	33803.5630		

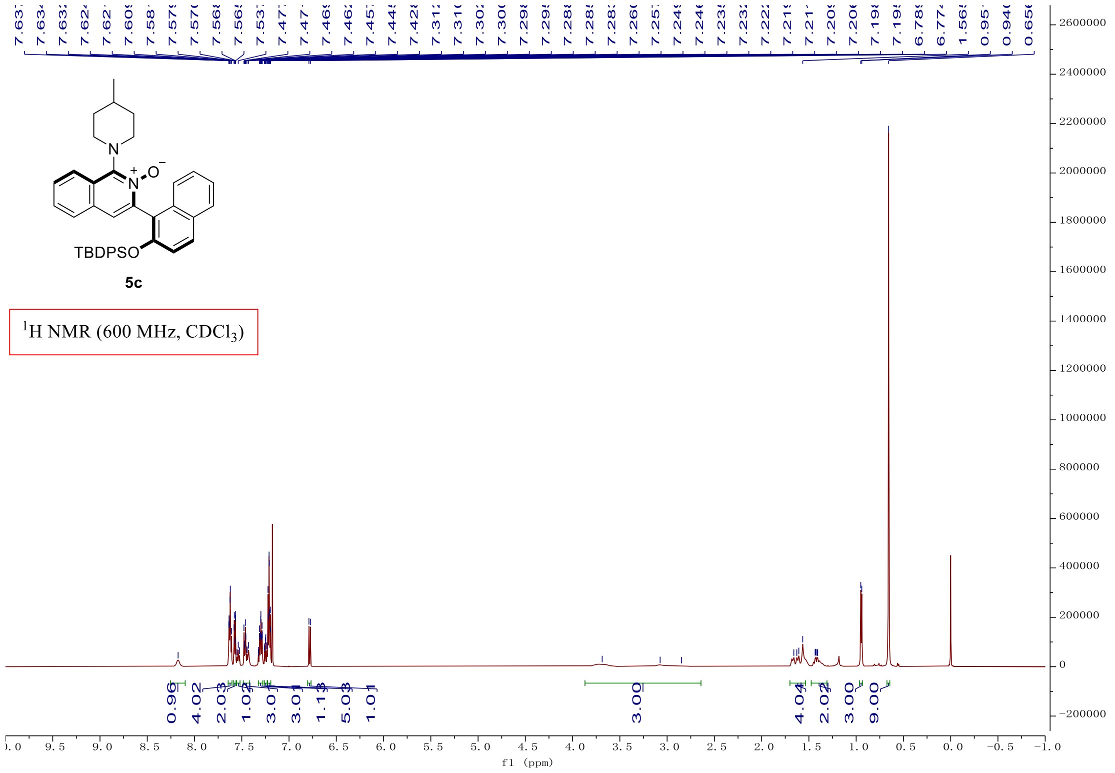
TBDPSO
5c
^{1}H NMR (600 MHz, $CDCl_3$)
f1 (ppm)

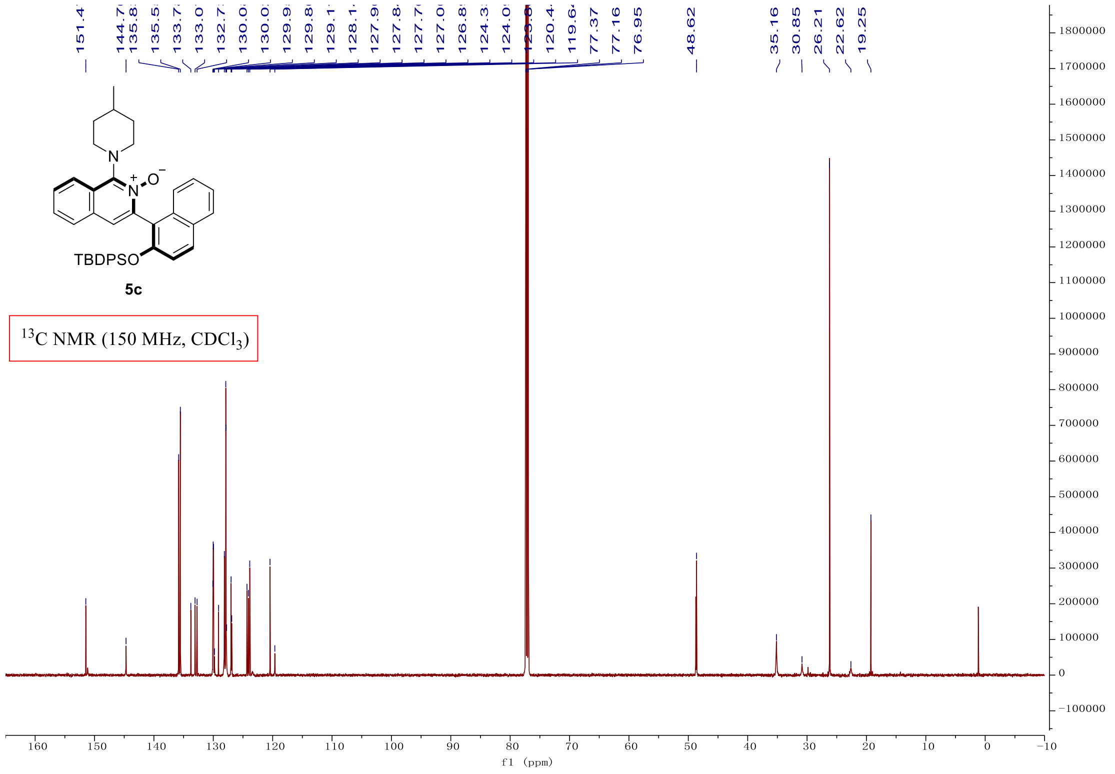
TBDPSO
5c
^{13}C NMR (150 MHz, $CDCl_3$)
151.4
144.7
135.8
48.62
35.16
30.85
26.21
22.62
19.25
77.37
77.16
76.95
f1 (ppm)

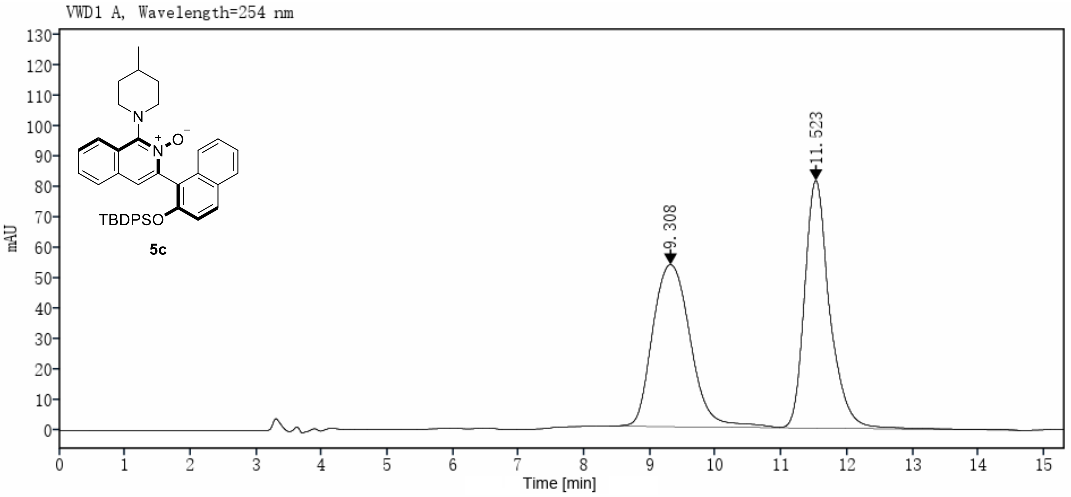

Signal: VWD1 A, Wavelength=254 nm

RT [min]Type	Area	Width[min]	Area%
9.308	2136.4814	0.633	49.5809
11.523	2172.5996	0.387	50.4191
Sum	4309.0811		

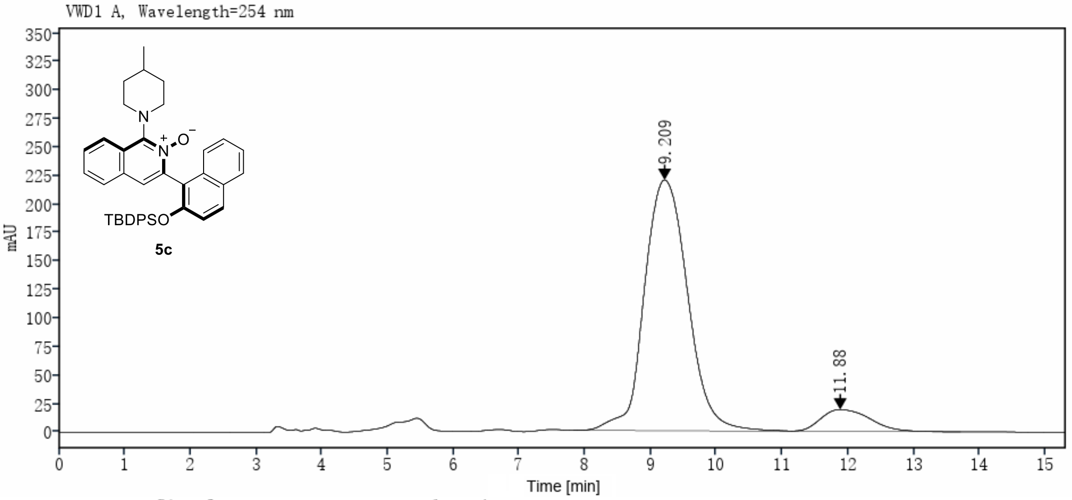

Signal: VWD1 A, Wavelength=254 nm

RT [min]Type	Area	Width[min]	Area%
9.209	10082.2607	0.713	90.9993
11.880	997.2325	0.833	9.0007
Sum	11079.4933		

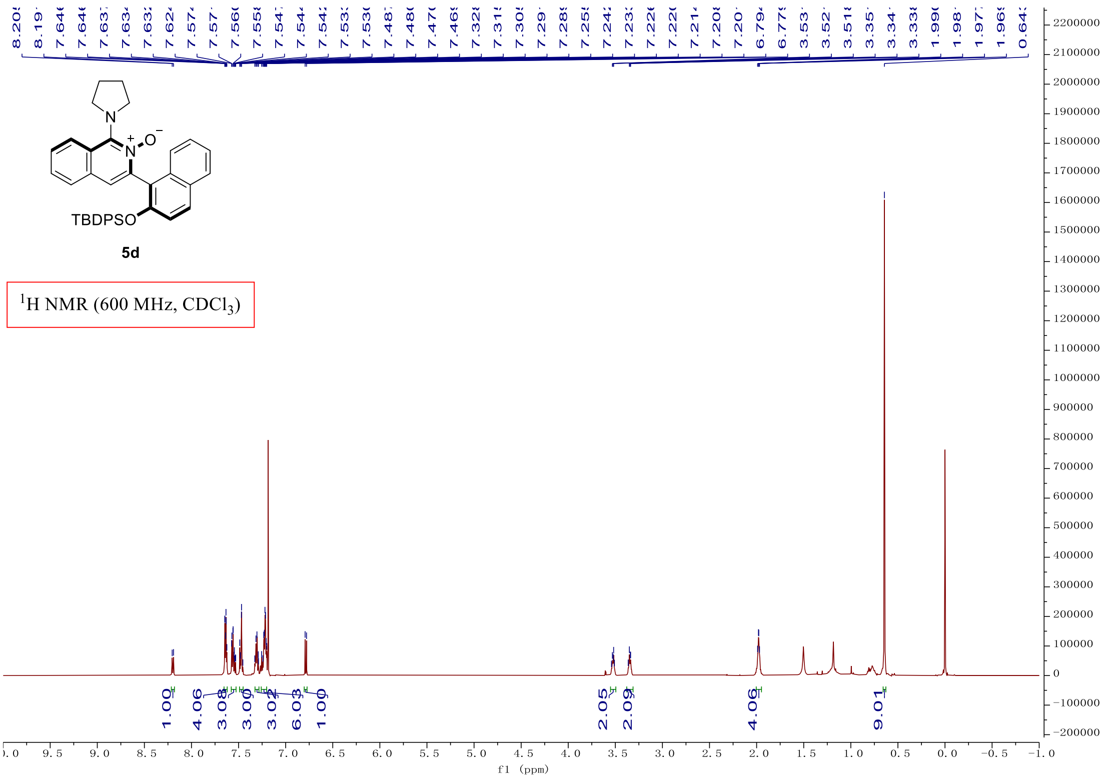

5d

^{1}H NMR (600 MHz, $CDCl_3$)

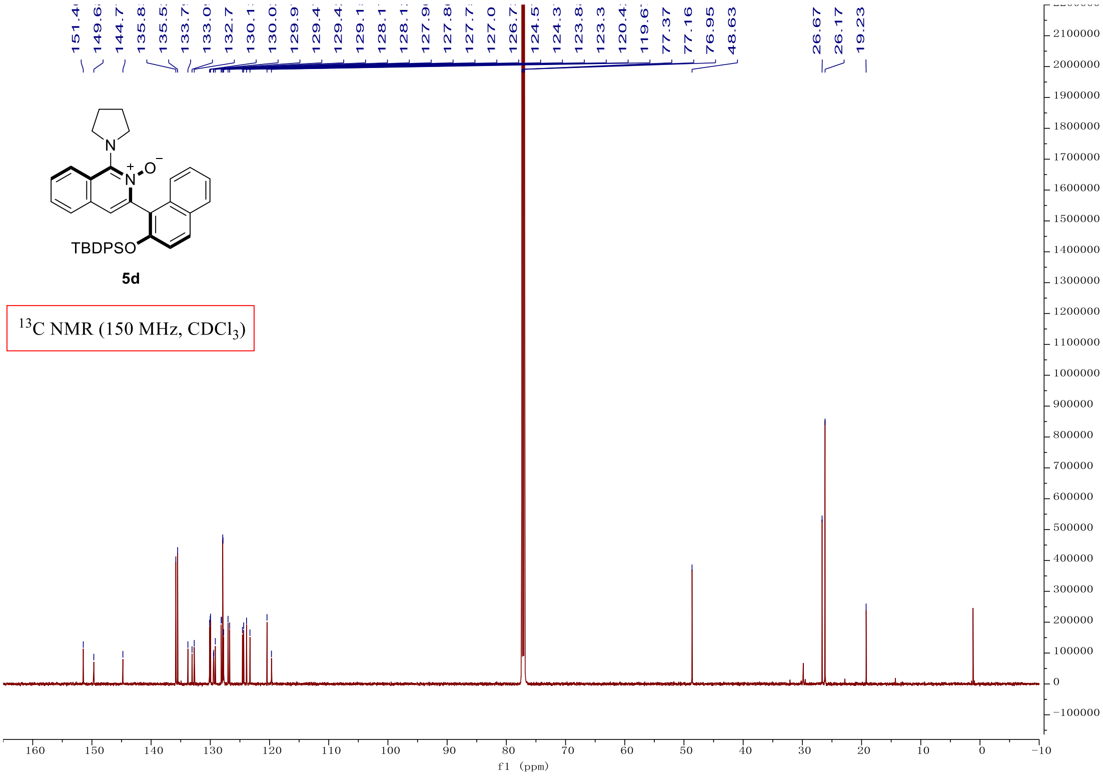

5d

^{13}C NMR (150 MHz, $CDCl_3$)

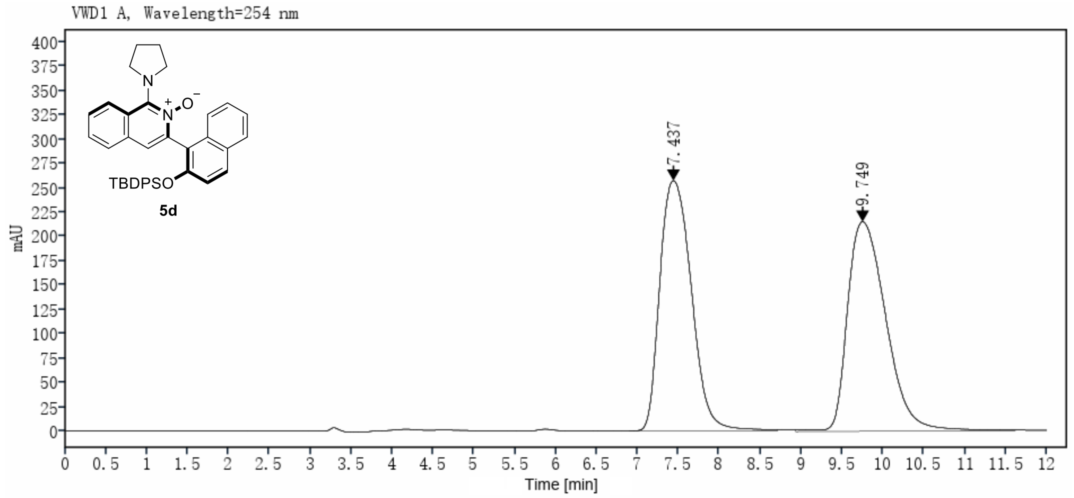

Signal: VWD1 A, Wavelength=254 nm

RT [min]Type	Area	Width[min]	Area%
7.437	6896.8208	0.428	49.4695
9.749	7044.7285	0.506	50.5305
Sum	13941.5493		

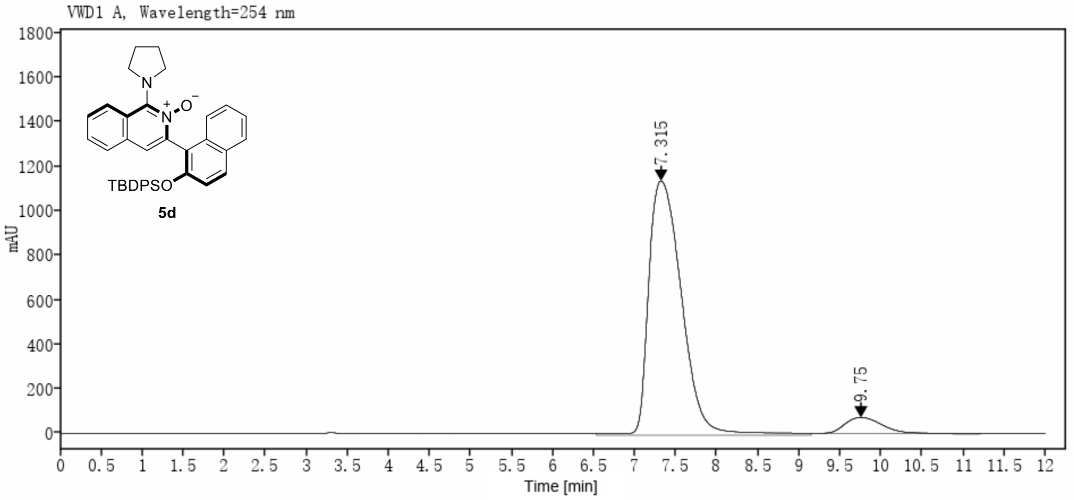

Signal: VWD1 A, Wavelength=254 nm

RT [min]Type	Area	Width[min]	Area%
7.315	32050.1738	0.431	93.1546
9.750	2355.1787	0.495	6.8454
Sum	34405.3525		

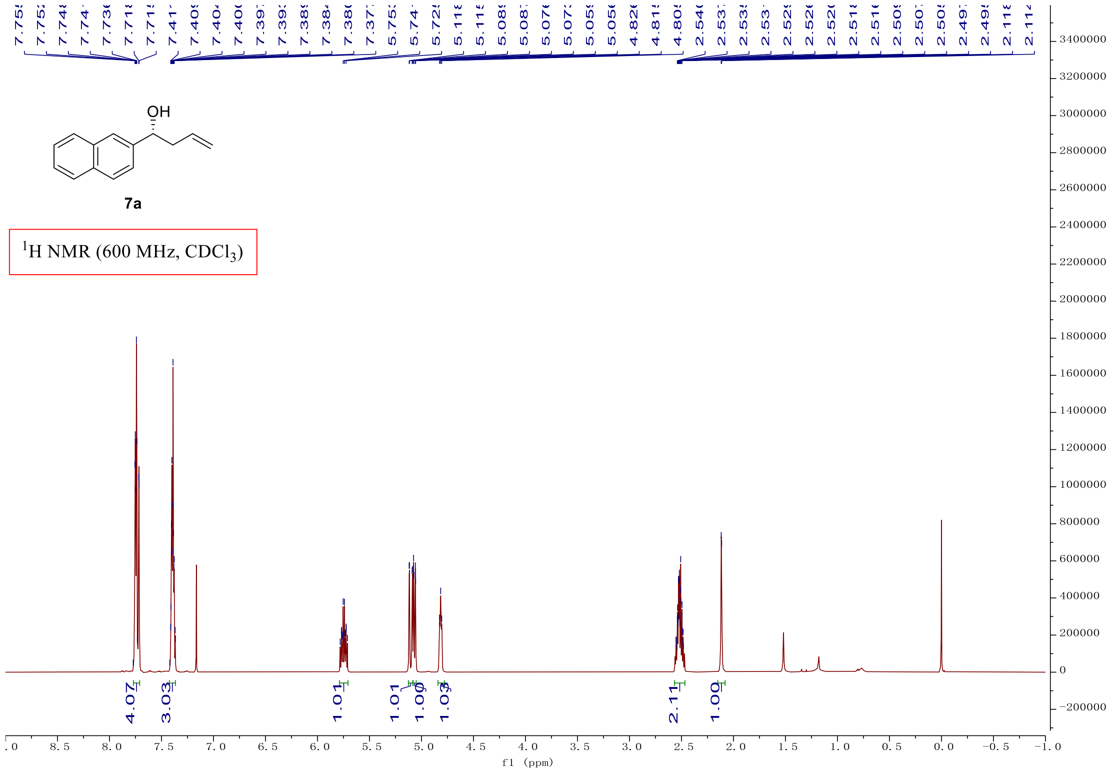
OH
7a
^{1}H NMR (600 MHz, CDCl$_3$)
f1 (ppm)

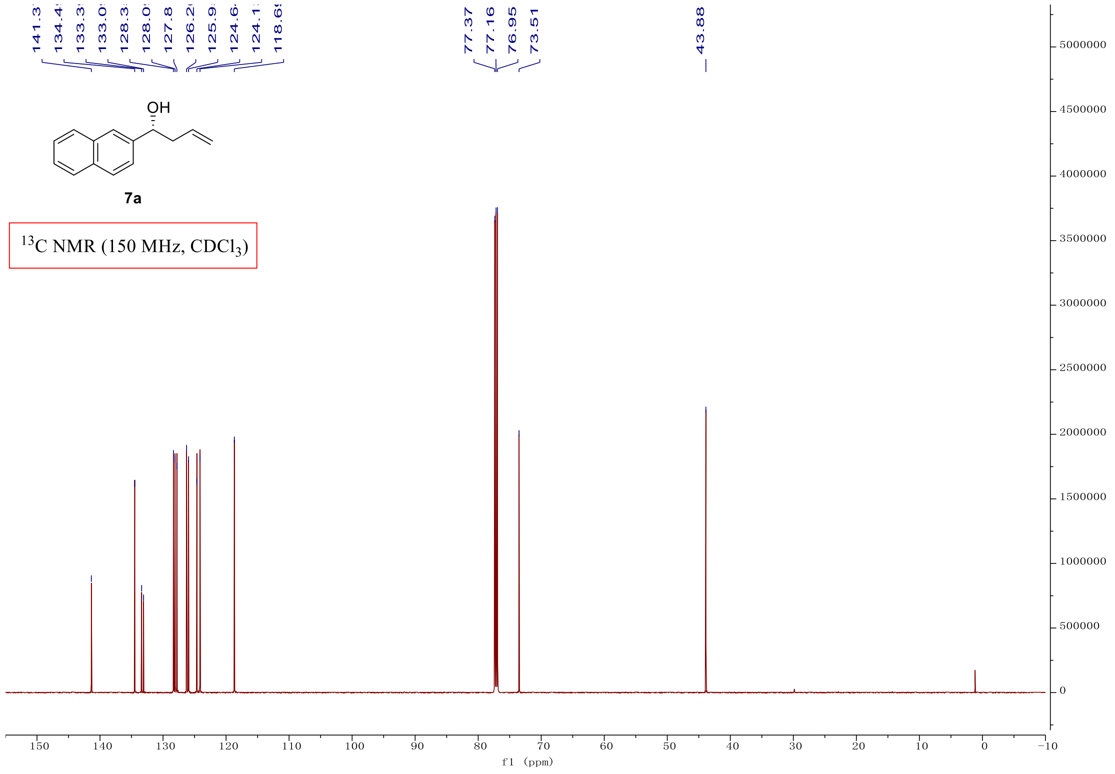
77.37
77.16
76.95
73.51
43.88
OH
7a
^{13}C NMR (150 MHz, CDCl$_3$)
f1 (ppm)

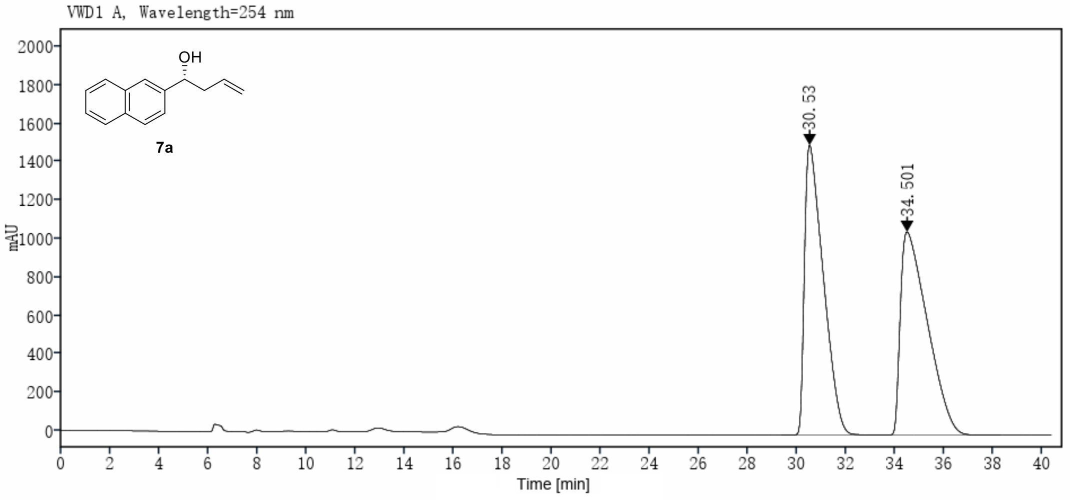

**Signal:**	VWD1 A, Wavelength=254 nm		
RT [min]Type	Area	Width[min]	Area%
30.530	80286.5703	0.840	49.9124
34.501	80568.3281	1.220	50.0876
Sum	160854.8984		

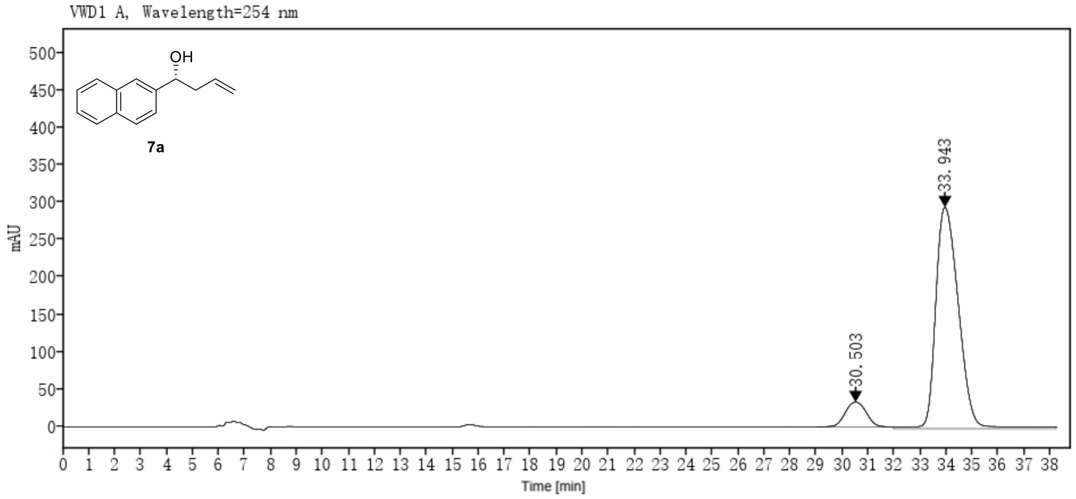

**Signal:**	VWD1 A, Wavelength=254 nm		
RT [min]Type	Area	Width[min]	Area%
30.503	1868.6622	0.893	9.2630
33.943	18304.6816	0.947	90.7370
Sum	20173.3439		

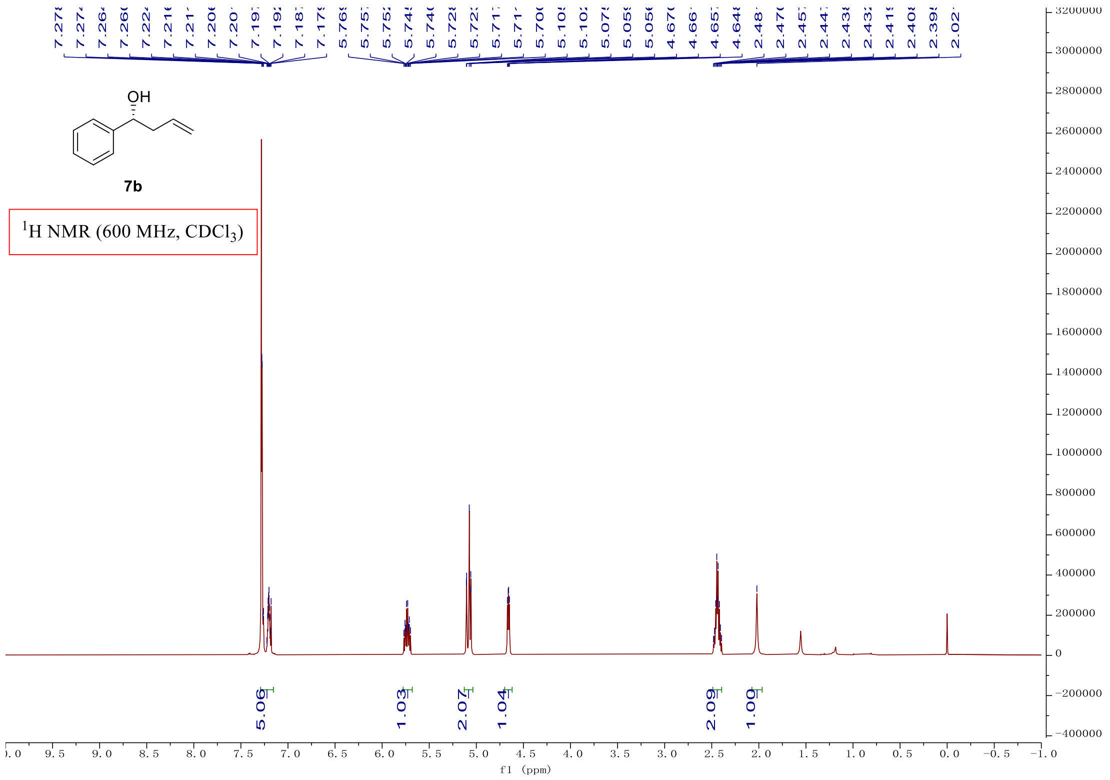
OH
7b
1H NMR (600 MHz, CDCl3)
5.06
1.03
2.07
1.04
2.09
1.00
f1 (ppm)

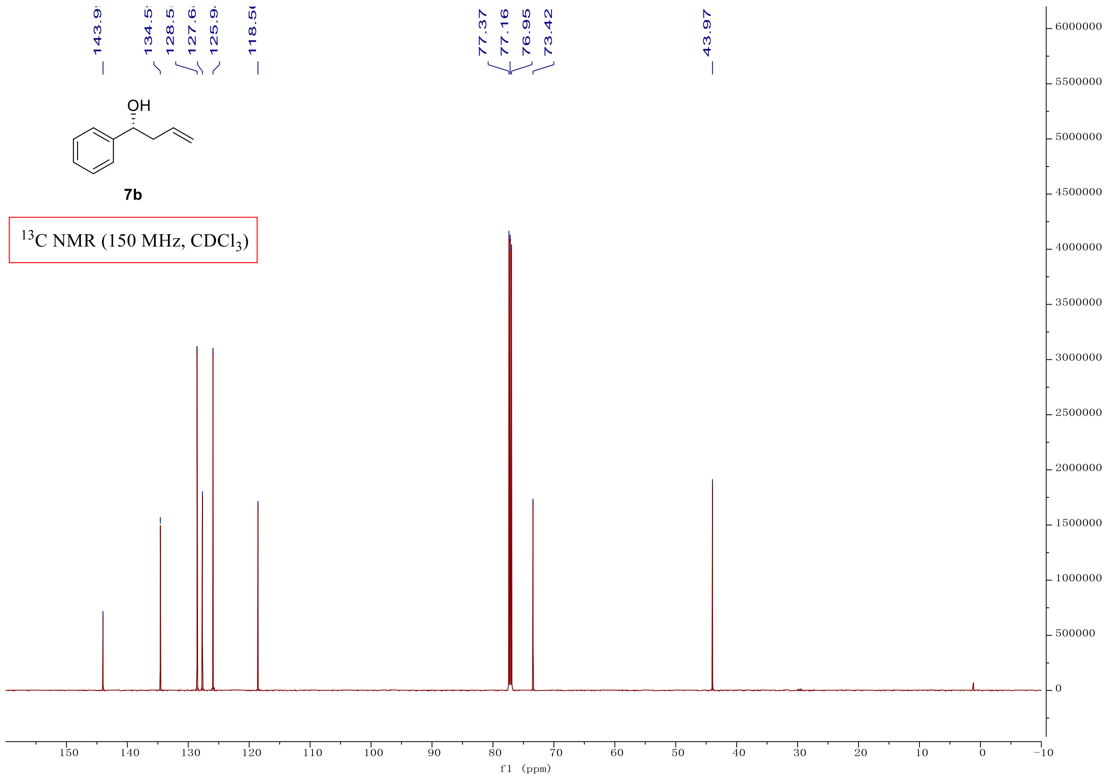
77.37
77.16
76.95
73.42
43.97
OH
7b
13C NMR (150 MHz, CDCl3)
f1 (ppm)

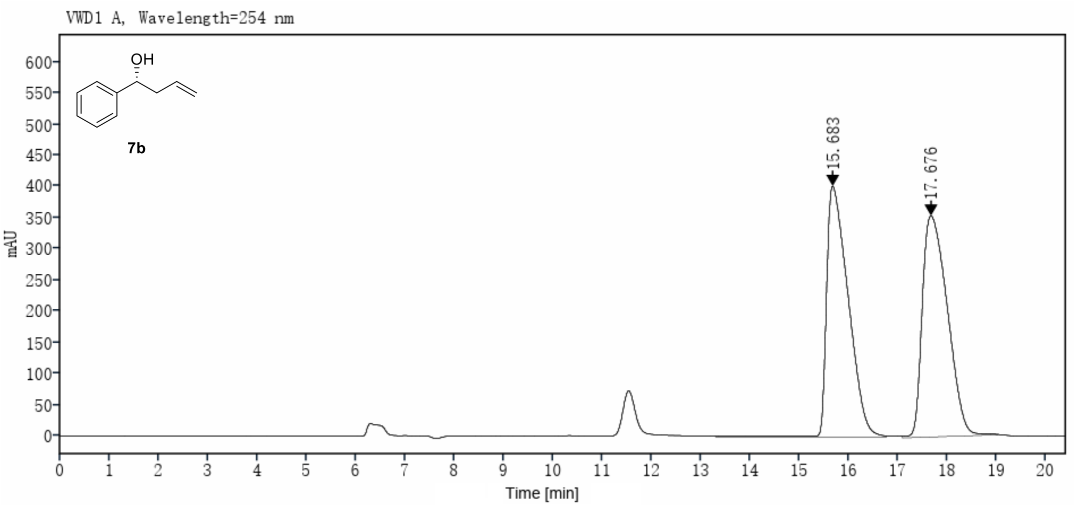

Signal: VWD1 A, Wavelength=254 nm

RT [min]Type	Area	Width[min]	Area%
15.683	12262.7129	0.481	49.4334
17.676	12543.8203	0.572	50.5666
Sum	24806.5332		

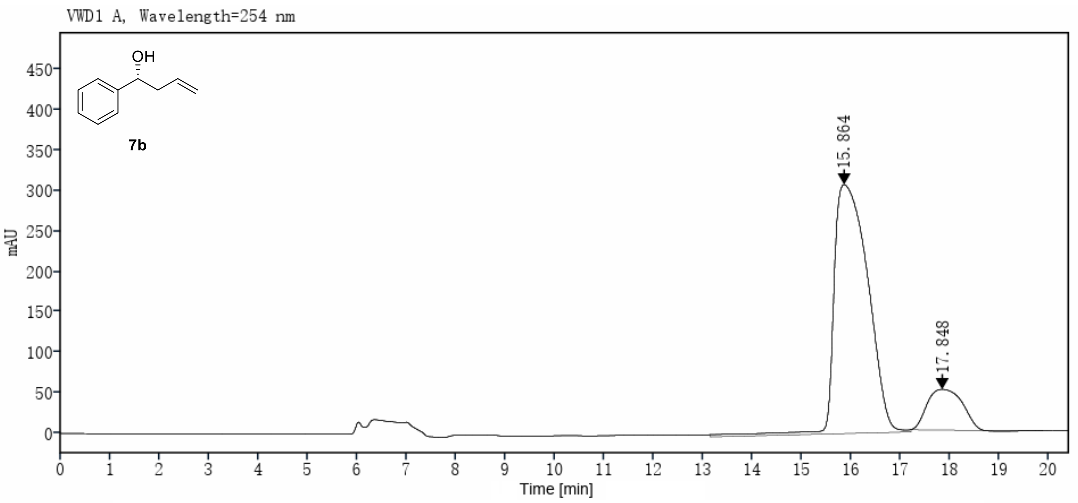

Signal: VWD1 A, Wavelength=254 nm

RT [min]Type	Area	Width[min]	Area%
15.864	14763.1279	0.779	85.4422
17.848	2515.3772	0.847	14.5578
Sum	17278.5051		

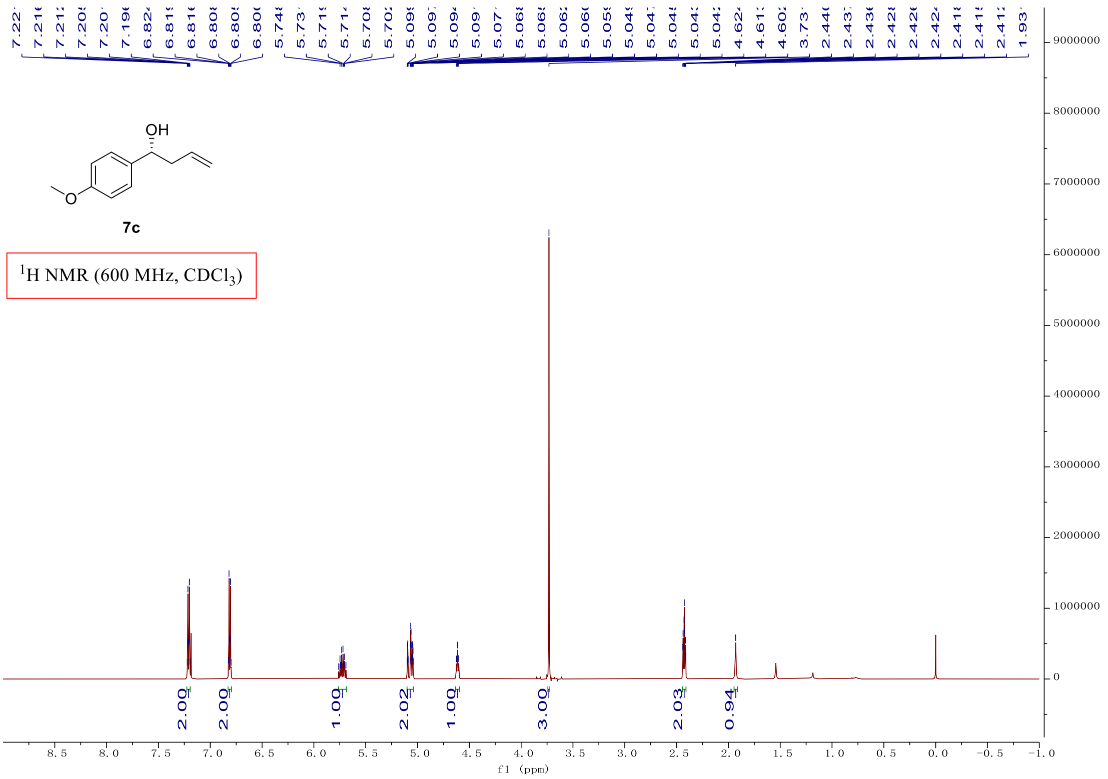
OH
O
7c
1H NMR (600 MHz, CDCl3)
2.00
2.00
1.00
2.02
1.00
3.00
2.03
0.94
f1 (ppm)

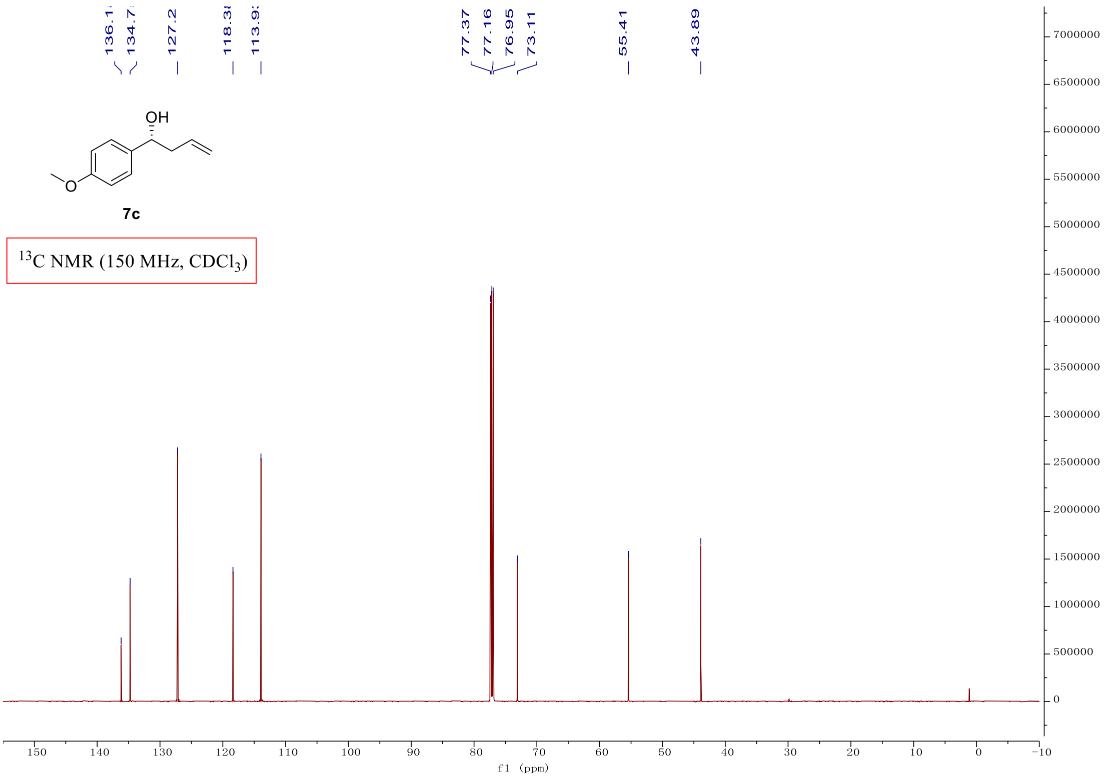
127.2
77.37
77.16
76.95
73.11
55.41
43.89
OH
O
7c
13C NMR (150 MHz, CDCl3)
f1 (ppm)

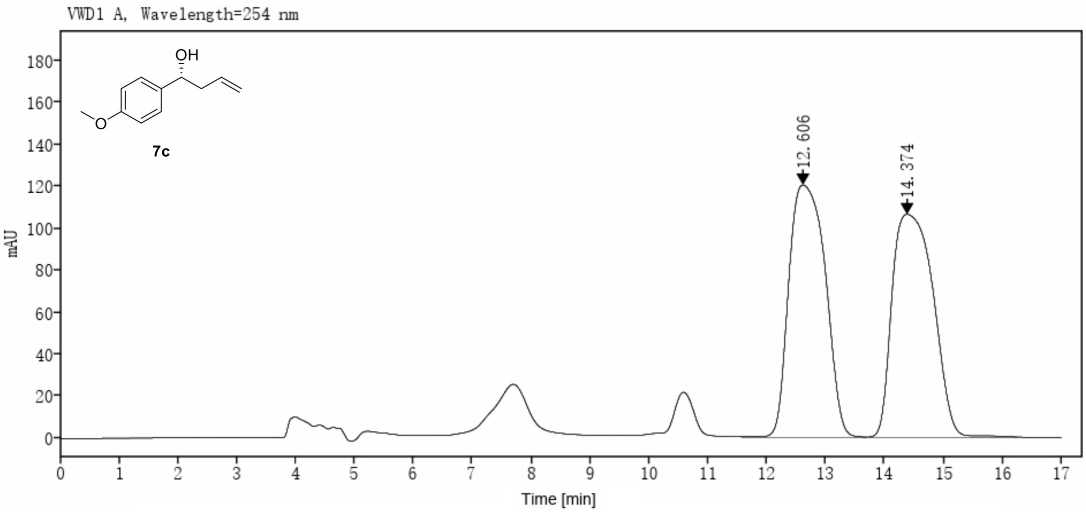

Signal: VWD1 A, Wavelength=254 nm

RT [min]Type	Area	Width[min]	Area%
12.606	5192.3379	0.721	50.0286
14.374	5186.3999	0.811	49.9714
Sum	10378.7378		

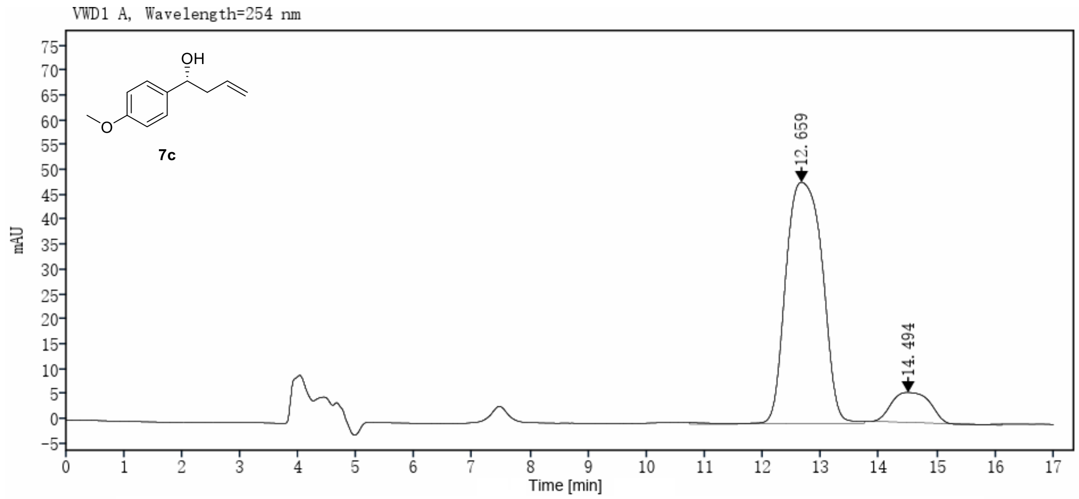

Signal: VWD1 A, Wavelength=254 nm

RT [min]Type	Area	Width[min]	Area%
12.659	2128.0513	0.720	88.4012
14.494	279.2150	0.760	11.5988
Sum	2407.2662		

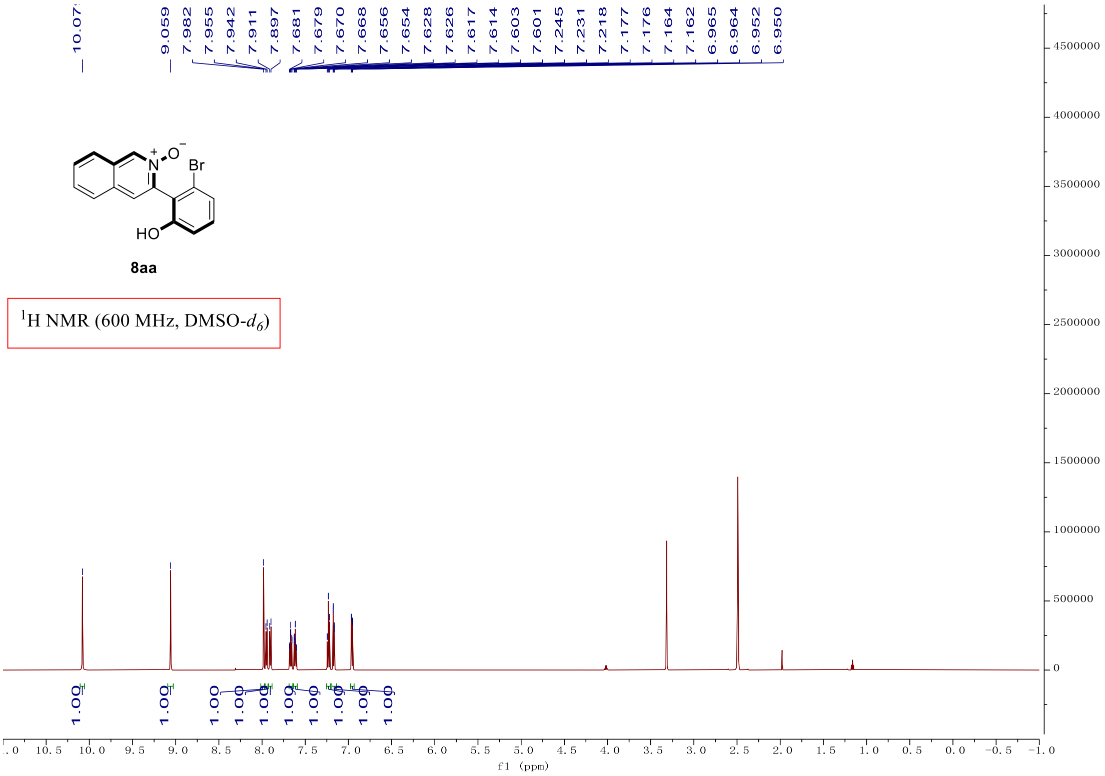
10.07
9.059
7.982
7.955
7.942
7.911
7.897
7.681
7.679
7.670
7.668
7.656
7.654
7.628
7.626
7.617
7.614
7.603
7.601
7.245
7.231
7.218
7.177
7.176
7.164
7.162
6.965
6.964
6.952
6.950
Br
HO
8aa
1H NMR (600 MHz, DMSO-d6)
1.00
1.00
1.00
1.00
1.00
1.09
1.09
1.06
1.06
1.00
f1 (ppm)

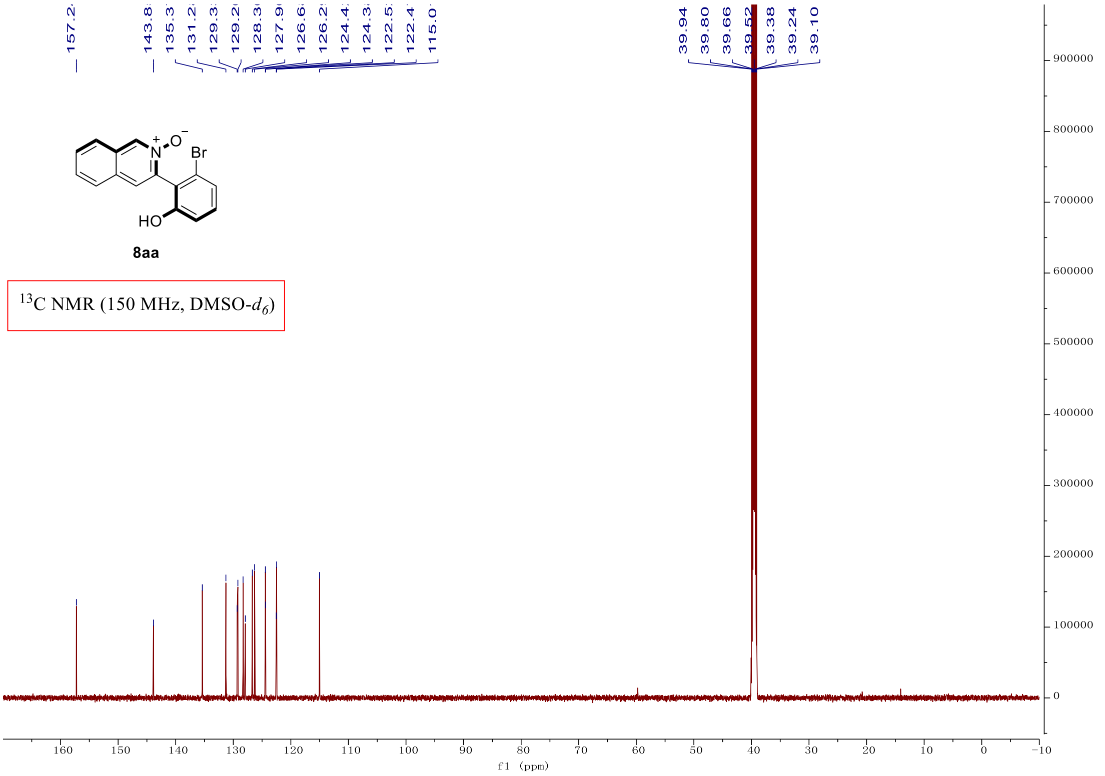
157.2
143.8
135.3
131.2
129.3
129.2
128.3
127.9
126.6
126.2
124.4
124.3
122.5
122.4
115.0
39.94
39.80
39.66
39.52
39.38
39.24
39.10
Br
HO
8aa
13C NMR (150 MHz, DMSO-d6)
f1 (ppm)

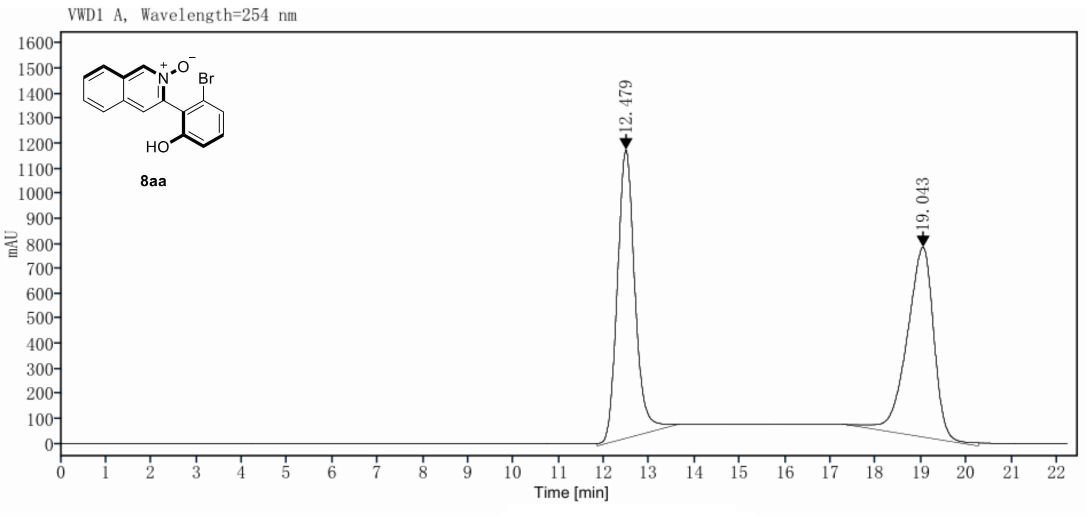

Signal:	VWD1 A, Wavelength=254 nm		
RT [min]Type	Area	Width[min]	Area%
12.479	31042.6797	0.403	49.8771
19.043	31195.6035	0.627	50.1229
**Sum**	62238.2832		

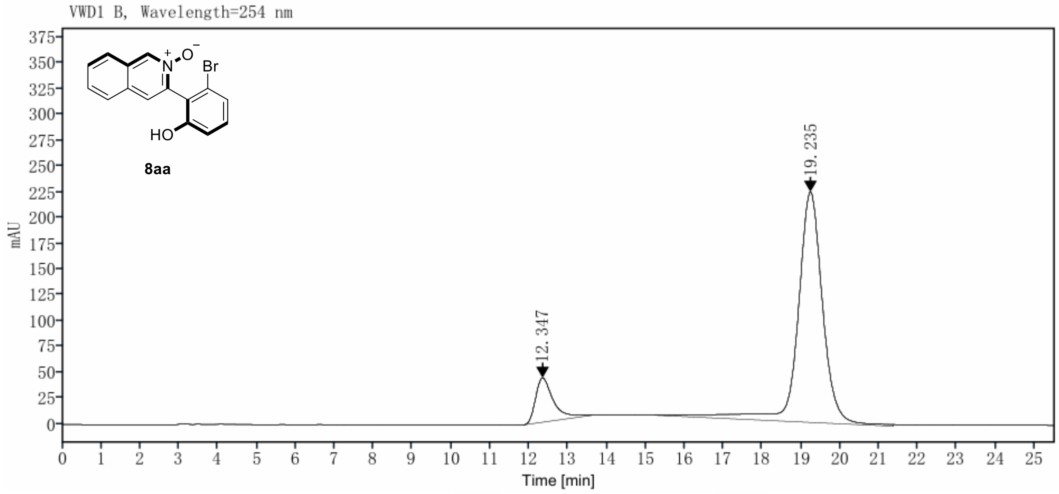

Signal:	VWD1 B, Wavelength=254 nm		
RT [min]Type	Area	Width[min]	Area%
12.347	1354.6488	0.467	11.9796
19.235	9953.2754	0.625	88.0204
**Sum**	11307.9242		

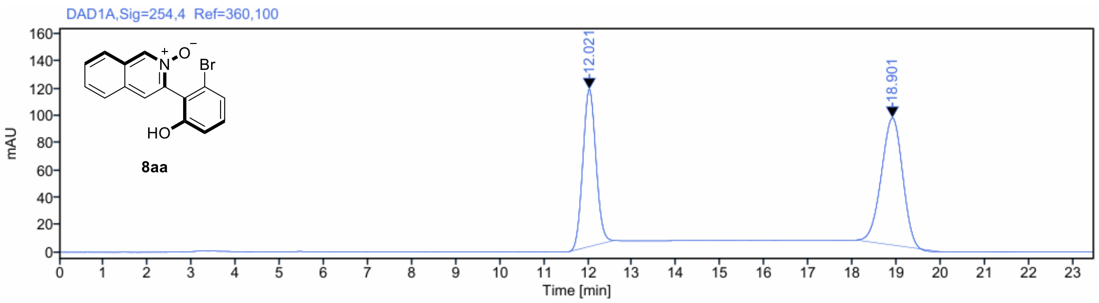

Signal: DAD1A,Sig=254,4 Ref=360,100

RT [min]	Type	Width [min]	Area	Height	Area%	Name
12.021	MM m	1.01	2390.39	115.45	43.45	
18.901	MM m	1.95	3110.98	93.30	56.55	
		**Sum**	**5501.37**			

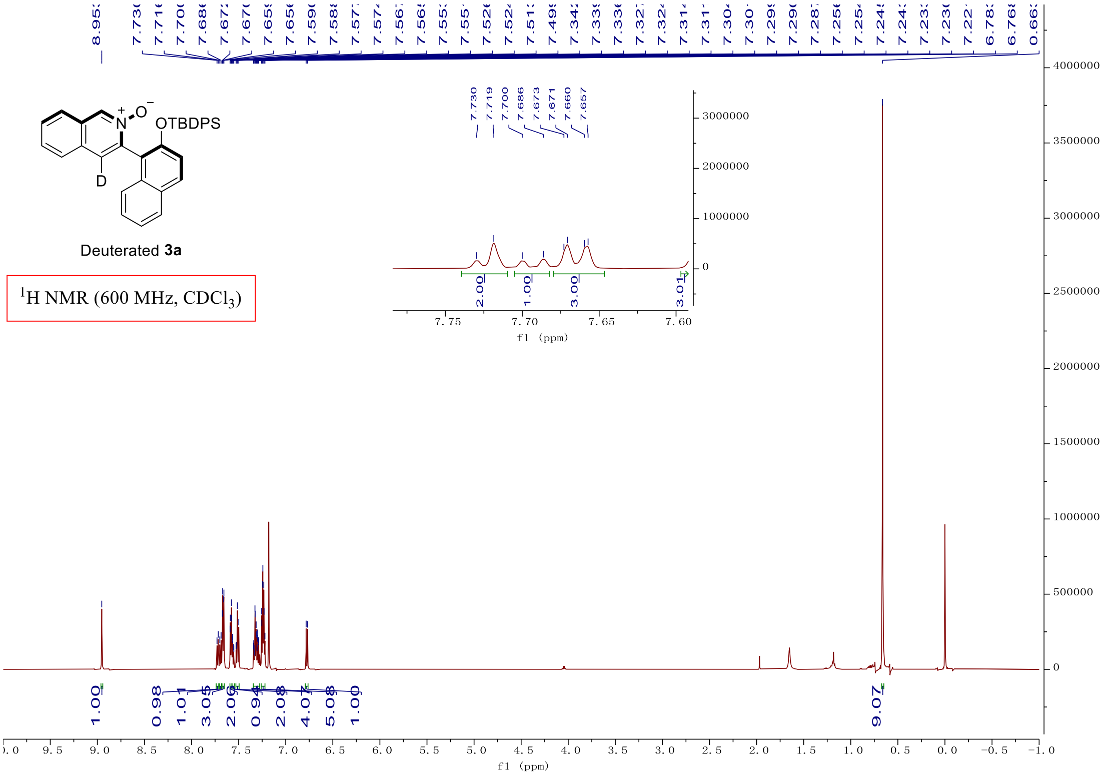

11. References

[1] a) Q.-Q. Yang, C. Chen, D. Yao, W. Liu, B. Liu, J. Zhou, D. Pan, C. Peng, G. Zhan, B. Han, *Angew. Chem. Int. Ed.* **2024**, *63*, e202312663; b) R. J. B. Schäfer, M. R. Monaco, M. Li, A. Tirla, P. Rivera-Fuentes, H. Wennemers, *J. Am. Chem. Soc.* **2019**, *141*, 18644.

[2] G. Li, C. Jia, K. Sun, Y. Lv, F. Zhao, K. Zhou, H. Wu, *Org. Biomol. Chem.* **2015**, *13*, 3207.

[3] a) A. V. Malkov, M. Orsini, D. Pernazza, K. W. Muir, V. Langer, P. Meghani, P. Kocovský, *Org. Lett.* **2002**, *4*, 1047; b) D.-W. Gao, Q. Gu, S.-L. You, *ACS Catal.* **2014**, *4*, 2741.

[4] a) S. Staniland, R. W. Adams, J. J. McDouall, I. Maffucci, A. Contini, D. M. Grainger, N. J. Turner, J. Clayden, *Angew. Chem. Int. Ed.* **2016**, *55*, 10755; b) Y. Huang, L. Yang, P. Shao, Y. Zhao, *Chem. Sci.* **2013**, *4*, 3275.

[5] a) A. Ahmed, R. A. Bragg, J. Clayden, L. W. Lai, C. McCarthy, J. H. Pink, N. Westlund, S. A. Yasin, *Tetrahedron* **1998**, *54*, 13277; b) K. T. Barrett, A. J. Metrano, P. R. Rablen, S. J. Miller, *Nature* **2014**, *509*, 71.

[6] M. J. Frisch, G. W. Trucks, H. B. Schlegel, G. E. Scuseria, M. A. Robb, J. R. Cheeseman, G. Scalmani, V. Barone, G. A. Petersson, H. Nakatsuji, X. Li, M. Caricato, A. V. Marenich, J. Bloino, B. G. Janesko, R. Gomperts, B. Mennucci, H. P. Hratchian, J. V. Ortiz, A. F. Izmaylov, J. L. Sonnenberg, D. Williams-Young, F. Ding, F. Lipparini, F. Egidi, J. Goings, B. Peng, A. Petrone, T. Henderson, D. Ranasinghe, V. G. Zakrzewski, J. Gao, N. Rega, G. Zheng, W. Liang, M. Hada, M. Ehara, K. Toyota, R. Fukuda, J. Hasegawa, M. Ishida, T. Nakajima, Y. Honda, O. Kitao, H. Nakai, T. Vreven, K. Throssell, J. A. Montgomery, Jr., J. E. Peralta, F. Ogliaro, M. J. Bearpark, J. J. Heyd, E. N. Brothers, K. N. Kudin, V. N. Staroverov, T. A. Keith, R. Kobayashi, J. Normand, K. Raghavachari, A. P. Rendell, J. C. Burant, S. S. Iyengar, J. Tomasi, M. Cossi, J. M. Millam, M. Klene, C. Adamo, R. Cammi, J. W. Ochterski, R. L. Martin, K. Morokuma, O. Farkas, J. B. Foresman, and D. J. Fox, *Gaussian 16, Revision C.01*, Gaussian, Inc., Wallingford CT, 2019.

[7] A. V. Marenich, C. J. Cramer, D. G. Truhlar, *J. Phys. Chem. B* **2009**, *113*, 6378.

[8] a) Y. Zhao, D. G. Truhlar, *Theor. Chem. Acc*. **2008**, *120*, 215; b) Y. Zhao, D. G. Truhlar, *Acc. Chem. Res.* **2008**, *41*, 157; c) W. R. Wadt, P. J. Hay, *J. Chem. Phys*. **1985**, *82*, 284; d) P. J. Hay, W. R. Wadt, *J. Chem. Phys*. **1985**, *82*, 270; e) R. Krishnan, J. S. Binkley, R. Seeger, J. A. Pople, *J. Phys. Chem.* **1980**, *72*, 650; f) A. D. McLean, G. S. Chandler, *J. Chem. Phys*. **1980**, *72*, 5639; g) T. H. Dunning, Jr., *J. Chem. Phys*. **1989**, *90*, 1007.

[9] R. Bauernschmitt, R. Ahlrichs, *J. Chem. Phys*. **1996**, *104*, 9047.

[10] a) C. Gonzalez, H. B. Schlegel, *J. Phys. Chem.* **1989**, *90*, 2154; b) C. Gonzalez, H. B.

Schlegel, *J. Phys. Chem.* **1990**, *94*, 5523.

[11] a) F. Weigend, R. Ahlrichs, *Phys. Chem. Chem. Phys.* **2005**, *7*, 3297; b)F. Weigend, *Phys. Chem. Chem. Phys.* **2006**, *8*, 1057.
